# A phase I study of autologous mesenchymal stromal cells for severe steroid-dependent nephrotic syndrome

**DOI:** 10.1172/jci.insight.169424

**Published:** 2023-09-22

**Authors:** Marina Vivarelli, Manuela Colucci, Mattia Algeri, Federica Zotta, Francesco Emma, Ines L’Erario, Marco Busutti, Stefano Rota, Chiara Capelli, Martino Introna, Marta Todeschini, Federica Casiraghi, Annalisa Perna, Tobia Peracchi, Andrea De Salvo, Nadia Rubis, Franco Locatelli, Giuseppe Remuzzi, Piero Ruggenenti

**Affiliations:** 1 Division of Nephrology, and; 2 Laboratory of Nephrology, Bambino Gesù Children’s Hospital IRCCS, Rome, Italy.; 3 Department of Pediatric Hematology/Oncology and Cell and Gene Therapy, Bambino Gesù Children’s Hospital IRCCS, Rome, Italy.; 4 Unit of Nephrology and Dialysis, Azienda Socio Sanitaria Territoriale (ASST), Bergamo, Italy.; 5 Center of Cellular Therapy “G. Lanzani,” Haematology Department, ASST Papa Giovanni XXIII, Bergamo, Italy.; 6 Istituto di Ricerche Farmacologiche Mario Negri IRCCS, Bergamo, Italy.; 7 Psychology Unit, Bambino Gesù Children’s Hospital IRCCS, Rome, Italy.; 8 Catholic University of the Sacred Heart, Rome, Italy.

**Keywords:** Clinical Trials, Nephrology, Immunotherapy

## Abstract

**BACKGROUND:**

Severe forms of idiopathic nephrotic syndrome (INS) require prolonged immunosuppressive therapies and repeated courses of high-dose glucocorticoids. Mesenchymal stromal cells (MSCs) have promising immunomodulatory properties that may be employed therapeutically to reduce patient exposure to medications and their side effects.

**METHODS:**

We performed a phase I open-label trial assessing safety and feasibility of autologous bone marrow–derived MSCs (BM-MSCs) in children and young adults with severe forms of steroid-dependent nephrotic syndrome. Following autologous BM-MSC preparation and infusion, oral immunosuppression was tapered. Safety, efficacy, and immunomodulatory effects in vivo were monitored for 12 months.

**RESULTS:**

Sixteen patients (10 children, 6 adults) were treated. Adverse events were limited and not related to BM-MSC infusions. All patients relapsed during follow-up, but in the 10 treated children, time to first relapse was delayed (*P* = 0.02) and number of relapses was reduced (*P* = 0.002) after BM-MSC infusion, compared with the previous 12 months. Cumulative prednisone dose was also reduced at 12 months compared with baseline (*P* < 0.05). No treatment benefit was observed in adults.

**In children, despite tapering of immunosuppression, clinical benefit was mirrored by a significant reduction in total CD19+, mature, and memory B cells and an increase in regulatory T cells in vivo up to 3–6 months following BM-MSC infusion:**

**CONCLUSION:**

Treatment with autologous BM-MSCs is feasible and safely reduces relapses and immunosuppression at 12 months in children with severe steroid-dependent INS. Immunomodulatory studies suggest that repeating MSC infusions at 3–6 months may sustain benefit.

**TRIAL REGISTRATION:**

EudraCT 2016-004804-77.

**FUNDING:**

AIFA Ricerca Indipendente 2016-02364623.

## Graphical abstract

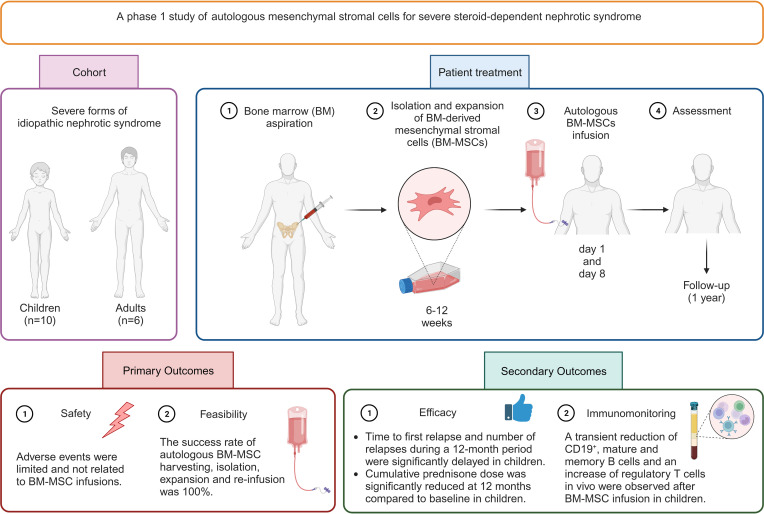

## Introduction

Idiopathic nephrotic syndrome (INS) is the most frequent form of NS of childhood, affecting 1.4–6.1 per 100,000 children (1). Children with a classic presentation (edema, hypoalbuminemia, and nephrotic-range proteinuria) receive a standard course of oral prednisone, and if they respond to this treatment a renal biopsy is not performed. When performed, usually “minimal-change disease” is observed, characterized by minimal mesangial inflammation or no changes on light microscopy, negative immunofluorescence in terms of immunoglobulin or complement glomerular staining, and foot-process effacement detected by electron microscopy. For this reason, the disease is considered an idiopathic podocytopathy, whose etiology is not clearly known (2). However, in 1974, Shalhoub proposed that T cells were involved in the disease pathogenesis based on favorable response to glucocorticoids, association with T cell malignancies and atopy, and lack of renal immunocomplex deposition (3). Over subsequent decades, many studies have attempted to further elucidate the underlying immunological mechanism leading to disruption of the glomerular filtration barrier and massive protein loss in the urine. In addition to downregulation of T regulatory (Treg) cells (2), the observation of the efficacy of B cell–depleting anti-CD20 monoclonal antibodies, primarily rituximab, in treating children with INS led to studies on B cell phenotype (reviewed in ref. 4). These have revealed B cell changes, mainly an increase in memory B cells, both at disease onset, prior to glucocorticoids, and following rituximab or ofatumumab infusion, where the reappearance of this B cell subset precedes relapse in children with INS (5, 6).

From a clinical standpoint, INS is very heterogeneous. Although steroid-sensitive forms never lead to kidney failure, 30% of patients experience a frequently relapsing or steroid-dependent evolution, which requires prolonged glucocorticoid use and the introduction of glucocorticoid-sparing immunosuppressive agents, such as calcineurin inhibitors, alkylating agents, and/or mycophenolate mofetil and rituximab. These immunosuppressive regimes are burdened by poor tolerability and substantial morbidity, including nephrotoxicity, myelotoxicity, and increased risk of cancer, lymphoproliferative disorders, and serious infections (7–9). Poor tolerability may even result in poor adherence to treatment (1). Approximately 15% of children, moreover, continue to relapse as adults, posing a major challenge in terms of long-term requirement for immunosuppression (1). There is therefore an unmet clinical need for safe and effective therapies for severe forms of INS, affecting children and young adults.

In this context, treatment with mesenchymal stromal cells (MSCs) could be a valuable therapeutic option to prevent relapses and spare glucocorticoid and immunosuppressive therapy. MSCs are multipotent progenitor cells resident in the bone marrow (BM) and other tissues (10). They can be obtained from BM cells after in vitro culture and expansion and can differentiate into several mesenchymal tissues, as well as home to inflamed sites, contributing to tissue repair and mediating potent immunomodulatory effects both in vitro and in vivo (11). The capacity to regulate immune responses leading to regeneration of damaged tissues, as well as in vitro observations that MSCs inhibit T cell activation and proliferation induced by specific stimuli provide a strong rationale for studies testing MSCs to modulate immune responses in diseases characterized by an immune dysregulation leading to tissue damage (10). A combination of in vitro coculture studies and animal studies demonstrated that MSCs can also induce the expansion of Treg cells and inhibit the expansion and/or the activation of B cells, NK cells, and myeloid-derived dendritic cells, also inducing the skewing of monocytes and macrophages toward an antiinflammatory phenotype (12, 13). Together, these data indicate that MSCs can suppress immune system activation by regulating cells of the adaptive and innate immune system. Therefore, the potential therapeutic role of MSCs has been tested in a variety of clinical settings, and several phase I and phase II studies have confirmed their safety with no adverse effects up to 1 year after treatment exposure (14–16) and suggested their effectiveness in a number of immune-mediated diseases, including glomerulopathies (12, 17–19).

It has been debated whether autologous patient-derived MSCs maintain the full spectrum of regenerative and immunomodulatory properties of MSCs obtained by third-party healthy donors (reviewed in ref. 11). To address this issue, we performed a proof-of-principle study in 5 children diagnosed with INS at the Bambino Gesù Children’s Hospital (OPBG). As controls, we used MSCs isolated from residual cells of 8 healthy donors (HDs) who donated BM for transplantation. BM-derived MSCs (BM-MSCs) were isolated and expanded ex vivo from both patients with INS and HDs, as previously described (20). Proliferative capacity, osteogenic and adipogenic differentiation, and senescence assays were performed as described by Starc et al. (21). More relevantly, immunomodulatory properties were assessed in terms of T and B cell proliferation and in terms of cytokine production. Notably, BM-MSCs from INS patients displayed a capacity to inhibit T and B cell proliferation and cytokine production similarly to that of BM-MSCs derived from HDs. Moreover, supernatant from BM-MSCs derived from INS patients was protective against adriamycin-induced podocyte damage, with reduced glomerular barrier permeability to bovine serum albumin (21).

Based on these encouraging preclinical results, we designed an open-label phase I study to primarily explore the safety and feasibility of autologous BM-MSC treatment in children and young adults with severe steroid-dependent, multi-relapsing forms of INS.

## Results

### Patients.

Following regulatory approval in July 2018, between September 2018 and February 2020 18 patients (10 children and 8 adults) were enrolled. Of these, 16 (all 10 children and 6 adults) received the autologous BM-MSC infusions according to protocol. Two adult patients did not receive autologous BM-MSCs, because following enrolment, their disease changed and they were considered “screening failures” due to lack of eligibility.

**Figure 1:**
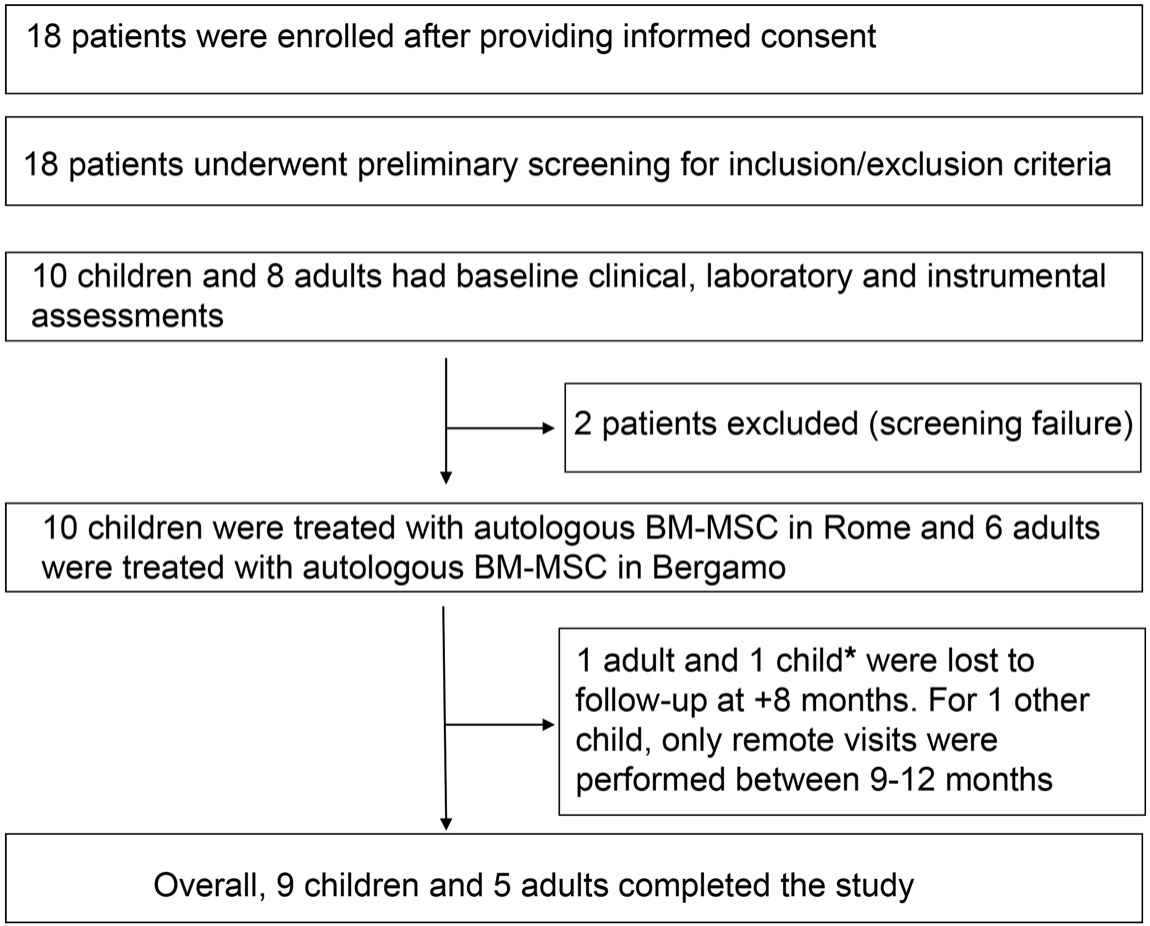
Study design. *For this child, a subsequent telephone call gave information relating to safety, relapses, and immunosuppression at these time points.

When the COVID-19 pandemic occurred, due to logistical difficulties and for the safety of patients, we had to modify our modality of study assessments, as face-to-face visits were not possible. We obtained complete clinical and laboratory data for 8 out of 10 children, while for 1 child we obtained complete data until 8 months after BM-MSC infusion and subsequently remote clinical and safety data until study completion, and for 1 other we obtained complete data until 8 months after MSC infusion, and then only retrospective clinical information (relating to relapses and immunosuppression) and safety data at 9 and 12 months. Of the 6 adult patients who received the BM-MSC infusions according to study protocol, 5 completed the study. One patient was obliged to skip visits after 8 months from BM-MSC infusion due to the COVID-19 pandemic. The last patient visit occurred on July 14, 2021 (Figure 1). Baseline patient characteristics and study drug production data for all treated patients are summarized in Table 1.

### Safety.

All patients who received the study drug (autologous BM-MSCs) were included in the safety analysis. All treated patients (10 children, 6 adults) were exposed to 2 infusions (on day 1 and day 8) of 1 × 10^6^ cells (range 0.75–1.25 × 10^6^)/kg of body weight of autologous BM-MSCs, according to study protocol. Pediatric patients were hospitalized for 1–2 nights both for BM harvesting and for BM-MSC infusions. Adults were hospitalized for 1 night for BM harvesting and followed for 4 hours after BM-MSC infusions. No infusion reactions occurred. All reported adverse events (AEs) were mild or moderate. All the 10 children and 5 of the 6 adults had at least one AE. Table 2 summarizes the number of events and the number of patients with events, categorized by MedDRA code (https://www.meddra.org/), in the study group as a whole (overall) and in children and adults considered separately. Infections were the most frequent AE and the respiratory tract was the organ system most frequently involved both in children and in adults. Rhinitis and cough were the most commonly reported symptoms, followed by pyrexia. As expected, these AEs were more frequent in children than in adults. No patient died during the study or discontinued the study prematurely because of treatment-emergent AEs. No AEs were considered related to the study drug. The frequency of treatment-emergent AEs of interest (infections) was consistent with the known incidence of infectious episodes per year in the specific patient population, i.e., children and young adults with severe forms of NS (22). No clinically meaningful changes in the safety laboratory parameters or vital signs were attributable to treatment. Seven serious AEs (SAEs), detailed in Table 3, were observed in 6 patients (37.5%). One SAE (malaise and vomiting in a child) occurred prior to the MSC infusions. Following MSC infusions, 1 child experienced 2 SAEs, both subsequent to a prolonged disease relapse, while other 3 patients, 2 children and 1 adult, experienced edema and oliguria related to a disease relapse, requiring hospitalization. Finally, 1 adult woman became pregnant after MSC infusions; the baby was born at term in good health, while the patient experienced 2 relapses after pregnancy, the first treated with glucocorticoids and the second with rituximab after remission. She is currently in remission and well. No SAEs were judged to be related to study medication, or unexpected, by the study investigators. A Safety Committee received periodic reports every 6 months and reviewed all AEs. In addition, as per protocol, an interim analysis was conducted after the first 10 patients had received BM-MSC infusions. Based on its results, the Safety Committee concluded that treatment was safe and the study could be continued up to completion of patient inclusion and planned follow-up.

### Efficacy.

**Figure 2:**
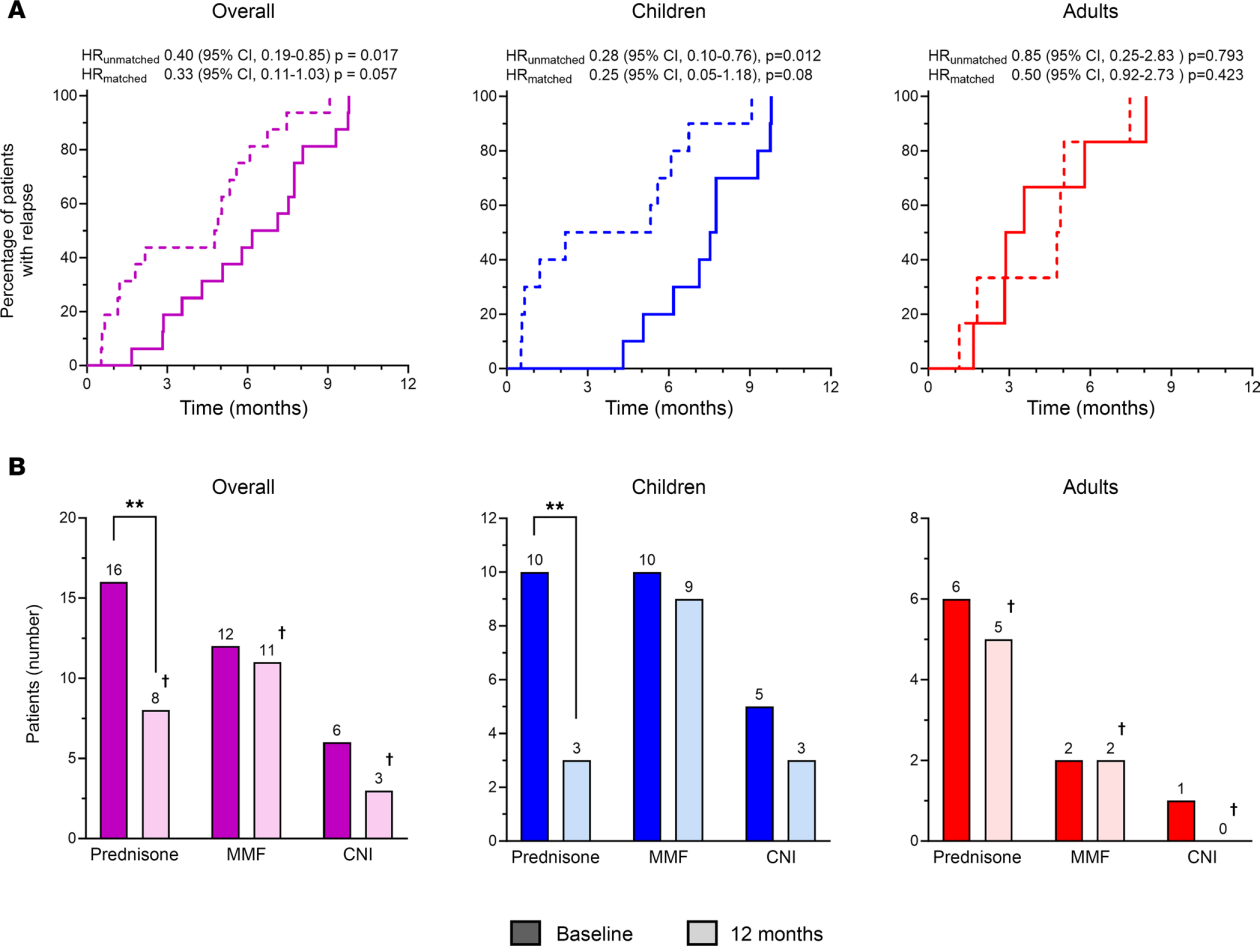
Autologous BM-MSC treatment efficacy in relapse-free survival and withdrawal of immunosuppressive drugs. (**A**) Relapse-free survival curve in the 12 months before (dotted line) and after (solid line) BM-MSC infusion shown overall (purple, left panel), in children (blue, central panel), and in adults (red, right panel). Unmatched Cox’s regression and a stratified Cox’s regression for matched pairs between the year before versus the year after of first MSC infusion was carried out. HR, hazard ratio. (**B**) Number of patients on immunosuppressive agents (prednisone, mycophenolate mofetil, calcineurin inhibitors) at baseline (full plots) and at +12 months (shaded plots) from BM-MSC infusion, shown overall (purple, left panel), in children (blue, central panel), and in adults (red, right panel). Bars represent the sum of patients in each group. Data were compared by Fisher’s exact test. **P < 0.01. ^†^One adult patient was lost to follow-up at +8 months, as detailed in Supplemental Table 2. MMF, mycophenolate mofetil; CNI, calcineurin inhibitors (cyclosporine or tacrolimus).

**Figure 3:**
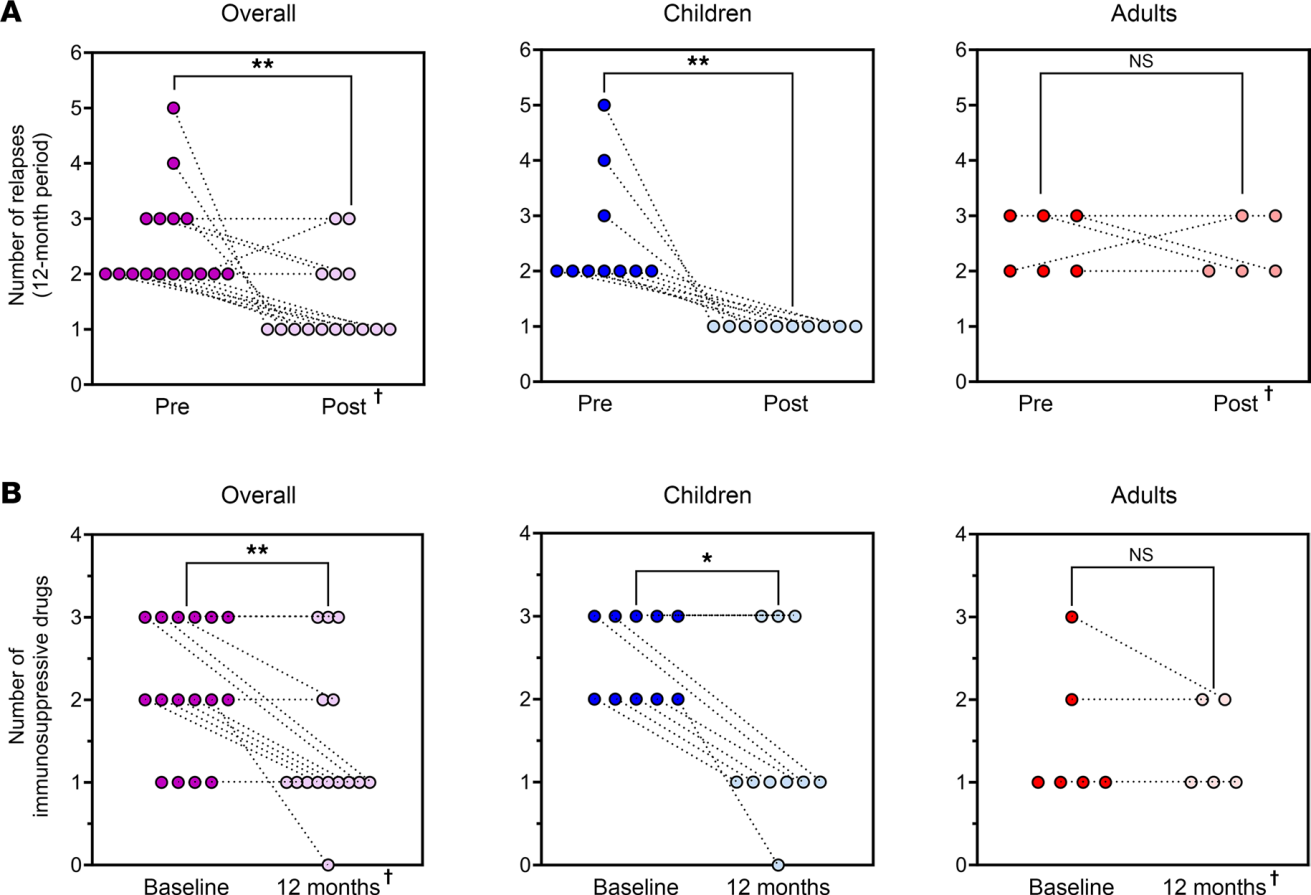
Autologous BM-MSC treatment efficacy in number of relapses and number of immunosuppressive drugs needed to maintain remission. (**A**) Single-patient representation of number of relapses in the 12 previous months (full dots, *n*_tot_ = 16) and in the 12 months after BM-MSC infusions (shaded dots, *n*_tot_ = 15) shown overall (purple, left panel), in children (blue, central panel), and in adults (red, right panel). Data were compared by Wilcoxon’s test. **P < 0.01. NS, not significant. ^†^One adult patient was lost to follow-up at +8 months, as detailed in Supplemental Table 2. (**B**) Single-patient representation of number of immunosuppressive drugs at baseline (full dots, *n*_tot_ = 16) and at +12 months after BM-MSC infusions (shaded dots, *n*_tot_ = 15) shown overall (purple, left panel), in children (blue, central panel), and in adults (red, right panel). Data were compared by Wilcoxon’s test. *P < 0.05, **P < 0.01. NS, not significant. ^†^One adult patient was lost to follow-up at +8 months, as detailed in Supplemental Table 2.

As shown in Figure 2A, all patients relapsed during the 12 months following BM-MSC administration. However, the median time to first relapse was delayed during the 12 months following BM-MSC infusion compared with the 12 months preceding BM-MSC infusion (6.7 vs. 4.8 months, respectively, *P* = 0.05) (Figure 2A). However, BM-MSC treatment significantly delayed median time to first relapse during a 12-month period in children (7.6 in the 12 months following BM-MSC infusions vs. 3.7 months in the previous 12 months, *P* = 0.02) but had no effect in adults (3.2 vs. 4.8 months, respectively, *P* = 0.99) (Figure 2A). Consistently, the median number of relapses was significantly reduced during the 12 months following BM-MSC infusion, compared with the previous 12 months in the study group considered as a whole (1 [IQR 1–2] vs. 2 [IQR 2–3], *P* = 0.003) (Figure 3A). Again, treatment effect was fully driven by the event reduction observed in children (1 [IQR 1–1] vs. 2 [IQR 2–3], *P* = 0.002), whereas no change was observed in adults (2 [IQR 2–3] vs. 2.5 [IQR 2–3], *P* = 0.99) (Figure 3A).

At baseline, all patients were on glucocorticoids and most of them were on 1 or 2 steroid-sparing agents, such as calcineurin inhibitors (cyclosporine A or tacrolimus) and an antiproliferative agent (mycophenolate mofetil) (Figure 2B). BM-MSC treatment significantly reduced the number of patients on glucocorticoids at 12 months after BM-MSC infusions as compared with baseline (before BM-MSCs). All 16 patients were on glucocorticoids at baseline as compared with 8 of the 15 patients on active follow-up at 12 months (*P* = 0.002; Figure 2B). As observed for relapses, the effect of BM-MSC treatment on glucocorticoid exposure was evident only in children (3 of 10 vs. 10 of 10 at baseline, *P* = 0.003; Figure 2B), with a reduction in the cumulative prednisone dose in the 12 months following BM-MSC treatment compared with the previous 12 months (1.6 [IQR 1.4–2.9] vs. 3.4 [IQR 2.2–5.6] g/m^2^, *P* < 0.05). The proportion of patients on calcineurin inhibitors or mycophenolate mofetil therapies did not change appreciably after BM-MSC treatment in both children and adults (Figure 2B). More generally, the median number of immunosuppressive drugs needed to maintain remission was significantly reduced at 12 months from BM-MSC treatment compared with the baseline in the study group considered as a whole (1 [IQR 1–2] vs. 2 [IQR 1–3], *P* = 0.008) and in children (1 [IQR 1–3] vs. 2.5 [IQR 2–3], *P* = 0.02), but not in adults (1 [IQR 1–2] vs. 1 [IQR 1–2], *P* = 0.99) (Figure 3B).

In a post hoc analysis, we created a scoring system to integrate in a single measurement the cumulative effects of reducing the number of relapses in the previous and subsequent 12-month periods and the number of immunosuppressive drugs needed to maintain remission at time of BM-MSC infusions and at +12 months. To this end, we simply cumulated these 2 values for each patient. We found that the median cumulative score was significantly reduced at 12 months after BM-MSC treatment compared with the previous 12 months in the study group considered as a whole (3 [IQR 2–4] vs. 5 [IQR 4–5], *P* < 0.001) and in children (2 [IQR 2–4] vs. 5 [IQR 4–5]), *P* = 0.002), but did not significantly change in adults (4 [IQR 3.5–4] vs. 4 [IQR 3–5], *P* = 0.63) (Supplemental Figure 1; supplemental material available online with this article; https://doi.org/10.1172/jci.insight.169424DS1).

Thus, autologous BM-MSC treatment was effective in reducing disease activity (i.e., delaying relapse while reducing immunosuppressive therapy) in children with severe steroid-dependent, multi-relapsing INS, but not in adults.

In order to determine whether BM-MSC treatment was effective in improving patient well-being, we evaluated quality of life questionnaires collected at time of BM-MSC infusions (baseline) and at +1, +6, and +12 months after BM-MSC infusions in children for whom we obtained complete data (9 out of the 10 included in the study). Despite the hospitalizations required per protocol both for BM harvesting and for BM-MSC infusions, no child had a worsening in the overall score of well-being and the median score of the parameter representing the physical health conditions significantly increased from 66 [IQR 25–84] to 100 [IQR 84–100] (*P* = 0.02) in comparing baseline with +12 months. As expected on the basis of clinical outcomes, this improvement was not observed in adult participants (data not shown).

We did not find any significant posttreatment change in height, weight, BMI, systolic and diastolic blood pressure (Table 4), incidence of diabetes, or cataract and, when available, DEXA score (data not shown), that could have been induced by prolonged glucocorticoid exposure, as compared with baseline.

### Immunomodulation.

**Figure 4:**
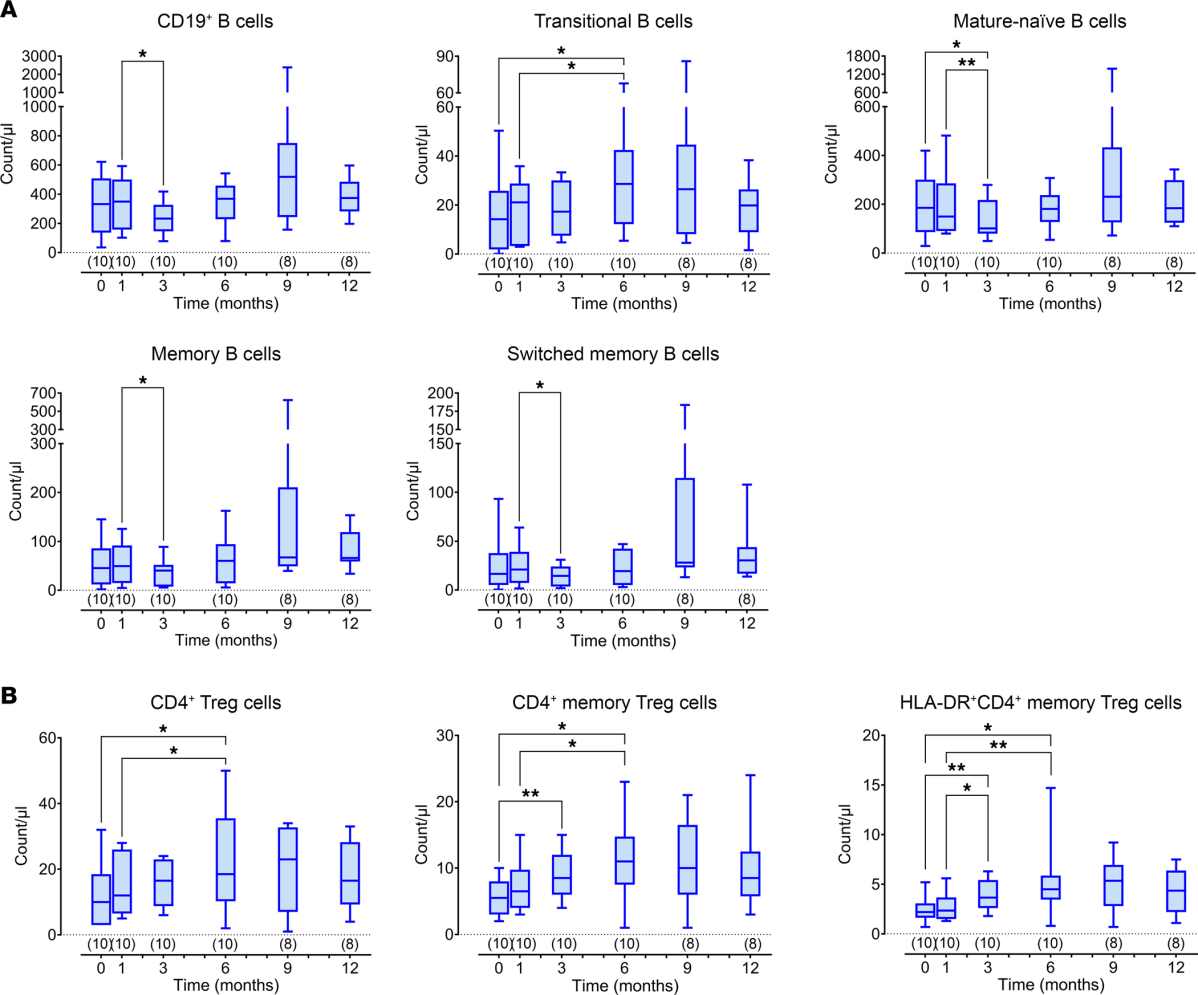
Pediatric immunomonitoring results. (**A**) Total CD19^+^ B cells and B cell subpopulations at baseline and at 1, 3, 6, 9, and 12 months from BM-MSC infusions. Numbers of available samples at different time points are indicated in parentheses (*n*). Differences between groups were compared using the paired Wilcoxon’s test. *P < 0.05; **P < 0.01. (**B**) Total, memory, and HLA-DR^+^ memory Treg cells at baseline and at 1, 3, 6, 9, and 12 months from BM-MSC infusions. Numbers of available samples at different time points are indicated in parentheses (*n*). All box-and-whisker plots represent the median and the 25th and 75th centiles; error bars represent the smallest and the largest values. Within-group differences were compared using paired Wilcoxon’s signed-rank test. *P < 0.05; **P < 0.01.

In keeping with clinical data, a significant but transient immunomodulation of B and T lymphocytes was observed in children but not in adults in the first 3–6 months after BM-MSC administration, despite concomitant tapering of immunosuppression, suggesting the possibility of a direct immunomodulatory effect of infused autologous BM-MSCs (Supplemental Table 1 and Figure 4). In particular, a significant reduction in total CD19^+^, mature, total memory, and switched memory B cells and a significant increase in transitional B cells was found in children at 3 and 6 months after treatment, respectively (Figure 4), along with a parallel significant increase in total, memory, and activated memory (HLA-DR^+^) CD4^+^ Treg cells (Figure 4). No significant modification of serum levels of IgG, IgA, and IgM was observed (Supplemental Table 1). In addition, BM-MSC treatment failed to exert inhibitory effects on B and T cell proliferation and secretion ability when cells, sampled during the follow-up, were activated by appropriate in vitro stimulation (Supplemental Table 1). Circulating levels and functional assessments of the different B and T cell subsets in the study group considered as a whole and in children and adults considered separately during the follow-up are summarized in Supplemental Table 1.

## Discussion

The key finding of this open-label phase I study is that treatment with autologous BM-MSCs of both children and adults affected by steroid-dependent, multi-relapsing INS is feasible, safe, and well tolerated. These results were obtained despite considerable logistical difficulties related to the COVID-19 pandemic, which determined some incompleteness of data. Moreover, over 12 months of observation after BM-MSC infusion, treatment remarkably delayed the onset of relapses and reduced their incidence, and at the same time substantially reduced the need for glucocorticoids to maintain remission in children, whereas it was definitely not effective in adults.

In terms of safety, numerous studies assessing the use of autologous MSCs in a consistent number of patients have shown no adverse effects (23–27). This is the case both in the setting of acute graft-versus-host disease (GVHD) (15), and in the setting of renal transplantation (16), liver cirrhosis (28), respiratory diseases (29), myocardial infarction (30), and diabetes mellitus (31). The safety, tolerability, and efficacy of treatment with autologous MSCs may have major clinical implications. Indeed, the use of MSCs from third-party donors is often preferred because the cells are a ready-to-use, “off-the-shelf” product that offers the advantage of immediate availability. However, the risk of immune recognition and elimination of allogeneic MSCs by the immune system of non–profoundly immunosuppressed individuals, such as those affected by INS, is a major limitation that could affect both the tolerability and the efficacy of allogeneic cells, especially with repeated infusions (32). For this reason, we chose a “patient-designated” approach with the use of autologous, BM-derived cells. In the context of a chronic disease like INS, which offers sufficient time for MSC ex vivo expansion, autologous cells can be employed to overcome the risk of immune rejection, provided that they are functionally active, as we showed with our proof-of-principle study (21). In the present study, BM harvesting and MSC production did not pose substantial hurdles and were performed with identical and GMP-approved protocols, in tight synergy, in both centers.

Consistently with the above considerations, the infusions of autologous BM-MSCs were well tolerated by all treated patients, with no infusion reactions and no subsequent AEs that could be linked to the study intervention. Perhaps more surprisingly, the procedure of harvesting and BM-MSC infusions was deemed tolerable by all patients, with positive feedback in health-related quality of life questionnaires for most patients.

Another interesting finding of the study was that treatment benefit was restricted to children, whereas no treatment effect was observed in adults. Our finding, however, should be interpreted with caution. For reasons linked to the greater rarity of the condition in young adults, and to the major impact of the COVID-19 pandemic on this study, our adult population consisted of 6 patients, which limited the study power. On the other hand, this clinical observation found a correlate in the immunomonitoring, which revealed that in children, treatment with BM-MSCs appeared to directly determine in vivo a significant transient decrease in total, mature, and memory B cells and an increase in total, memory, and activated memory Treg cells, despite the tapering of concomitant immunosuppression. In contrast, we failed to detect any appreciable immune cell modulation in adults. In a previous report, heterologous BM-MSCs were safe and therapeutically effective in patients with refractory GVHD (61 children and 31 adults), with a tendency to a better outcome in terms of response and survival in children compared with adults (33). Although our study is limited by the small number of adult patients that were enrolled, our results suggest that age-related factors might in part explain the efficacy of autologous BM-MSCs in reducing the severity of INS when comparing younger and older patients. The immune system of children is indeed known to be more “plastic” than that of adults for B cells (34) and T cells (35, 36). MSCs are known to exert immunomodulatory effects on both innate and adaptive immune responses (11). They inhibit T and B cell activation and proliferation and induce Treg cell differentiation by in vitro cocultures. This inhibition is directly mediated by the production of MSC-derived soluble factors that, however, require a dynamic crosstalk with cocultured lymphocytes to be secreted, as demonstrated by the lack of an inhibitory effect exerted by MSC supernatants alone (11, 37). In agreement with this observation, our results show that MSC treatment is effective in reducing B cell maturation and differentiation into memory subsets and in inducing Treg cells in vivo, but loses this immunomodulatory effect when in vitro stimulation is performed on ex vivo–isolated B and T cells, supporting the need for paracrine signals for MSC inhibition (11, 37).

Severe forms of steroid-dependent NS are burdened by substantial morbidity, mainly related to the need for prolonged courses of immunosuppressive agents to maintain stable remission, which despite intense treatment is not always achieved. A few patients therefore require, in addition to their maintenance immunosuppression for years, repeated cycles of high-dose oral glucocorticoids. This treatment leads to marked side effects in terms of short stature, reduced bone mineral density, and severe infections and has a major impact on quality of life in terms of repeated hospitalizations, ability to attend school, socialize, participate in sports for patients, and of prolonged care for their families. Compared with standard of care with oral and i.v. immunosuppressive medications, the use of autologous BM-MSCs has the major advantage of low, or nil, morbidity, and allows children receiving it to experience a significant, albeit transient, improvement in their disease relapses and intensity of immunosuppression. We did not observe a significant reduction in the burden of glucocorticoid side effects in this study. Most likely, this is due to the transient benefit of BM-MSC treatment, which was invariably followed within 12 months by disease relapse, with the consequent need for repeated courses of oral prednisone in all patients.

To the best of our knowledge, only 1 study on the safety and efficacy of MSC treatment was performed in an INS setting (19). Eleven children with multidrug-resistant NS, who had failed a median of 3 previous lines of therapy, completed 3 infusions at 0, 14, and 21 days of allogeneic cord blood–derived MSCs at 1.5 × 10^6^ cells/kg and no infusion-related AE or toxicity was observed. In the 9 patients assessable for efficacy, the median proteinuria did not change significantly 12 months after treatment compared with baseline (8.13 vs. 9.07, *P* = 0.98), while 3 patients were in partial or complete remission. Thus, safety and tolerability findings were similar to those we observed in our present study, but treatment effect of infused MSCs was clearly less convincing, most likely because multidrug-resistant forms of INS are significantly more refractory to immunomodulatory interventions than steroid-dependent forms (38). Also, we cannot exclude that allogeneic cells may have a different efficacy in vivo compared with autologous MSCs in these severe forms. This is also suggested by the lack of induction of Treg cells in vivo by allogeneic cord blood–derived MSCs, in contrast to our observation of a clear effect of autologous BM-MSCs on B and T cells in vivo.

The main limitations of our phase I study are the small sample size and the single-arm, open-label design, as well as the use of the patient’s prestudy status as a reference for the assessment of efficacy of BM-MSC infusions. This study design is justified by the rarity and severity of the disease and by the type of intervention. Thus, at this stage, the complexity of the intervention was acceptable only for patients with severe disease who were orphans of any alternative rescue therapy. The study design was approved by the Italian regulatory agency (i.e., AIFA) and conforms to EU guidelines.

A few patients failed to fully recover data due to the major logistical difficulties related to the COVID-19 pandemic that precluded a complete per-protocol patient follow-up. However, we obtained complete clinical and safety data in 9 of 10 children and 5 of 6 adult patients, suggesting a benefit of treatment in children and no appreciable effect in adults. These encouraging findings may provide the background for a prospective, randomized, controlled trial of autologous BM-MSCs even in pediatric patients with less severe disease.

Despite the limitations of a small phase I study, our findings suggest that a single infusion cycle of autologous BM-MSCs is safe and transiently effective in children with severe steroid-dependent NS. The clinical efficacy of this treatment approach in children is also reflected by in vivo modifications in B and T cell subpopulation profiles, with a transient decrease in total, mature, and memory B cells and a transient increase in total, memory, and activated memory Treg cells. More importantly, it identifies 3–6 months as the time period in which the immunomodulatory effect of the BM-MSC infusions peaks and then wanes. At this time, a second infusion cycle could prolong immunomodulation and optimize the therapeutic efficacy of BM-MSC treatment. This approach, which is supported by the reassuring safety profile of BM-MSC treatment in this setting, may be implemented in a phase II/III study targeting children with severe forms of steroid-responsive NS burdened by substantial toxicity, in whom the cost of this treatment may be justified by its significant potential benefit.

## Methods

### Study design and participants.

We performed an open-label, nonrandomized phase I clinical study enrolling and treating pediatric patients at the IRCCS Bambino Gesù Children’s Hospital (Rome, Italy) and young adult patients at the Azienda Socio-Sanitaria Territoriale – Ospedale Papa Giovanni XXIII (Bergamo, Italy). The trial is registered with EudraCT (number 2016-004804-77). The investigational drug (autologous BM-MSCs), trial design, and procedures are described in Supplemental Material (Investigational Medicinal Product Description, Investigator Brochure, and Study Protocol). Patients were eligible for the study if they were between 5 and 40 years of age and had difficult-to-treat, frequently relapsing, or steroid-dependent INS. We included those with at least 2 relapses in the previous year in spite of prednisone and/or 1 or more other immunosuppressive steroid-sparing agent. Only patients reported to invariably relapse upon treatment tapering or withdrawal and on stable (from at least 1 month) complete (<0.3 g/24 hours for adults or <4 mg/h/m^2^ for children) or partial (<3.5 g/24 hours for adults or <40 mg/h/m^2^ for children) remission of the INS were included. Patients with previous rituximab treatment could be included, but reconstitution of B cells, defined as total CD19^+^CD20^+^ lymphocyte count greater than 5% of total lymphocytes by flow cytometry, had to be documented. We included patients with a histological diagnosis of minimal-change disease (MCD), focal segmental glomerulosclerosis (FSGS), or mesangial proliferative glomerulonephritis (MPG). We excluded patients with advanced renal failure (creatinine clearance <20 mL/min/1.73 m^2^), calculated using the Schwartz formula or the Cockroft-Gault formula, as appropriate, refractory, or persistent INS, defined as absence of partial remission after 6 months or absence of complete remission after 2 years and genetic mutations associated with intrinsic abnormalities of the glomerular barrier (NPHS1, NPHS2, WT1, ACTN4, LAMB2, PLCγ1, CD2AP, SMARCAL1, COQ6, TRPC6, LMX1B, INF2, MYO1E, PTPRO, SCARB2, ITGA3) who were therefore expected not to benefit from immunomodulatory therapy. The conditions required for enrolment of patients and the inclusion and exclusion criteria as indicated in the study protocol were assessed in the screening phase, during which potentially eligible patients underwent clinical and laboratory work-up, and when necessary, a renal biopsy.

### Procedures.

Following successful screening, a BM sample (50 mL of BM aspirated in an heparinized syringe) was harvested from iliac crests under sedation from each patient and processed within a timeframe of maximum 4–6 hours from harvesting. Both children and adults were hospitalized briefly for this procedure. BM cells were directly seeded in cell-stack flasks up to first harvesting (P1) and subsequently cells were expanded in multiple (up to 6) 5-layer flasks until final harvesting (P2) following identical procedures in the GMP-approved cell factory facilities in both participating centers, as previously reported (23, 39). Briefly, autologous MSCs were purified and expanded for 6–12 weeks in culture medium consisting of α-MEM with 5% platelet lysate, which avoids the need of fetal calf serum, thereby minimizing adverse immune responses to xenogenic antigens, to obtain autologous BM-MSCs for therapeutic use (23, 39). Upon reassessment of clinical status and disease remission on day 0, each patient received 2 i.v. infusions of autologous BM-MSCs on day 1 and day 8. The planned dose was 1 × 10^6^ MSCs/kg before thawing, with an acceptable dose range of 0.75 × 10^6^ MSCs/kg to 1.25 × 10^6^ MSCs/kg (maximum dose 100 × 10^6^ MSCs). Vital signs were monitored prior to infusion, directly following infusion, and then hourly for 2 hours. Premedication was administered as per center practice with chlorpheniramine and/or paracetamol. Both children and adults were hospitalized briefly for each infusion.

One month after the first BM-MSC infusion, steroid and/or concomitant immunosuppressive therapy was tapered and, whenever possible, progressively withdrawn. Immunosuppressive drugs were withdrawn in the following order: first glucocorticoids by reducing the prednisone dose by 2.5–5 mg every 1–2 weeks, then calcineurin inhibitors (cyclosporine A or tacrolimus), and finally antiproliferative agents (azathioprine, mycophenolate mofetil, or cyclophosphamide) by a quarter of the original dose every 2–3 weeks, in order to achieve complete withdrawal within 6 to 9 months. These guidelines were aimed at standardizing immunosuppressive therapy tapering/withdrawal, to limit the potential confounding effect of different approaches on data interpretation, but the principal investigator had the possibility to delay progressive dose reductions according to patient-specific conditions, reporting, and justifying this on the patient’s case report form.

INS recurrence was defined as 3 or more positive Albustix dipsticks for proteinuria on 3 consecutive days or by positive dipsticks (1+ to 3+) for 7 consecutive days. Patients with a relapse of INS were treated with high-dose glucocorticoids as per center practice. If the reintroduction of a second-line steroid-sparing immunosuppressive agent was deemed necessary, the best approach was chosen as per center practice and recorded in the patient’s case report form.

On follow-up, urine was tested every other day at home by Albustix. Routine laboratory exams, 24-hour proteinuria (or protein/creatinine ratio), and “immunomonitoring” were performed at baseline and after 1, 3, 6, 9, and 12 months from the first MSC infusion, according to the time schedule defined by the study protocol (Supplemental Table 2).

The methods for classifying/recording AEs, the definitions used to describe an AE, an SAE, an unexpected AE together with the criteria used to assess severity of AE, relationship of AE to study drug(s), and outcome are detailed in the Study Protocol (Supplemental Material). Briefly, all AEs of any severity were recorded in the patient records and in trial case report forms for the duration of the study, from enrolment to study completion. No distinction was made between treatment-emergent AEs and others in terms of detection and reporting. Grading of severity was performed according to GCP, and relation to the study drug was at the investigator’s judgment. Safety reports were compiled every 6 months throughout the study and sent to the Safety Committee for evaluation. An interim analysis was performed as per protocol when more than 10 patients had been treated. Common Terminology Criteria for Adverse Events were employed for grading of all AEs.

To record clinical parameters (including urinary protein dipstick, prednisone dose, antibiotic treatment) and tightly monitor for potential side effects of treatment (fever, runny nose, asthma, cough, vomiting, diarrhea), patients or parents of underage patients were asked to complete and submit each month a monthly spreadsheet with daily information. This was particularly useful to maintain a reliable monitoring of all main relevant clinical parameters in the emergency setting of the pandemic, during which for some months in-person visits were not possible in Bergamo.

We assessed quality of life with age-appropriate questionnaires — the Short Form Health Survey 36 (SF-36) for adolescents and adults (40) and Child Health Questionnaire Parent Form 50 (CHQ-PF50) for parents of children (41) — given to patients or parents before treatment (baseline) and at +1, +6, and +12 months following treatment during follow-up visits.

### Outcomes.

The study primarily aimed to assess the safety and feasibility of autologous MSC infusions in patients with severe forms of INS. Secondary study objectives included assessing the efficacy of autologous MSC infusions in reducing disease relapses and exposure to immunosuppressive drugs.

The secondary efficacy endpoints were recurrence of INS, dose of immunosuppressive therapy to prevent further INS relapses, adverse effects of immunosuppressive therapy (arterial hypertension and need for antihypertensive therapy, obesity and impaired glucose tolerance, dyslipidemia, renal dysfunction, stunted statural growth, infections), and kidney function at baseline and at 1 year after MSC administration.

### Quality of life.

Quality of life was assessed by 2 instruments according to the patient’s age group. For patients unable to respond independently, the CHQ-PF50 (41) was chosen. This instrument assesses general quality of life through 14 categories: physical functioning 6 items, role/social limitations — physical, general health perceptions, bodily pain/discomfort, family activities, role/social limitations — emotional/behavioral, parental impact, self-esteem, mental health, behavior, family cohesion, change in health. Scores were transformed in scale from 0 to 100. Higher scores indicate better or more positive quality of life. For all other patients, the SF-36 (40) was selected. This is a self-administered, patient-completed questionnaire designed to quantify health status and measure health-related quality of life. The SF-36 assesses quality of life through 8 scales: physical functioning, limitations due to physical health, limitations due to emotional problems, energy and fatigue, emotional well-being, social activities, pain, and general health perception. Scores were transformed in scale from 0 to 100. Higher scores indicate better or more positive quality of life.

### Cell collection.

Peripheral blood mononuclear cells (PBMCs) were isolated by Ficoll-Paque Plus (Amersham Biosciences) density-gradient centrifugation at baseline and at 1, 3, 6, 9, and 12 months after BM-MSC infusion. Cells were frozen in FBS with 10% DMSO and stored in liquid nitrogen until flow cytometry analysis was performed.

### B cell phenotype and proliferation.

To discriminate the different B cell subpopulations, thawed PBMCs were stained with fluorochrome-conjugated monoclonal antibodies (see Supplemental Table 3) directed against CD19, CD24, CD27, CD38, CD45, IgD, IgM, and IgG (BD Biosciences) and then analyzed by multicolor flow cytometry (see Supplemental Figure 2A for gating strategy). Live cells were identified based on the FSC/SSC lymphocyte gate and then selected as CD45^+^CD19^+^ total B cells. Subsets of gated total B cells were identified based on the expression of surface markers as follows: transitional (CD38^hi^CD24^hi^), mature-naive (CD38^int^CD24^lo^), and plasmablasts (CD27^+^CD38^hi^), expressed as absolute counts. Memory B cells were defined as CD19^+^CD27^+^ cells and memory subclasses were defined as IgM memory (IgM^+^IgD^int^), switched memory (IgM^–^IgD^–^), and IgG^+^ switched memory B cells and expressed as absolute counts.

To evaluate B cell proliferation, PBMCs were loaded with 10 μM CellTrace CFSE (Thermo Fisher Scientific) to track divided cells and then cultured in complete medium at a concentration of 2.5 × 10^6^ cells/mL. CpGB ODN2006 (0.35 μM; Hycult Biotech) was used to stimulate the cells for 7 days. Cultured cells were then harvested and stained with fluorochrome-conjugated monoclonal anti-CD19 antibody to identify B cells. After gating live cells, proliferating B cells were identified as percentages of CFSE^–/lo^ among CD19^+^ B cells.

Serum IgG, IgA, and IgM were measured as part of routine analysis.

### T cell phenotype, proliferation, and cytokine secretion.

Thawed PBMCs were stained with Fixable Viability Stain 520 (FVS520, BD Biosciences) and then incubated with the fluorochrome-conjugated monoclonal antibodies (see Supplemental Table 3) directed against CD3, CD4, CD8, CD25, CD28, CD45, CD45RA, CD45RO, CD127, CD152 (CTLA4), FOXP3, and HLA-DR (BD Biosciences) and then analyzed by multicolor flow cytometry (see Supplemental Figure 2B for gating strategy). CD3^+^CD4^+^ or CD8^+^ T cells were gated on singlet CD45^+^ live (CD45^+^FVS520^–^) and then identified as naive (CD45RA^+^CD45RO^–^) and memory (CD45RO^+^CD45RA^–^) subsets. Subpopulations of CD4^+^ Tregs (CD4^+^CD25^hi^CD127^–^FOXP3^+^) were identified as resting (CD45RA^+^CD45RO^–^) or memory (CD45RO^+^CD45RA^–^) and their expression of HLA-DR and CTLA4 was also assessed.

Proliferation of T cells was evaluated after labeling PBMCs with 10 μM CellTrace CFSE (Thermo Fisher Scientific) and then incubating the cells for 6 days in TexMACS medium with and without T cells TransAct (Miltenyi Biotec) at 37°C and 5% CO_2_. After incubation, PBMCs were stained with fluorochrome-conjugated monoclonal antibodies directed against CD3, CD4, and CD8 (BD Biosciences). PBMCs were stained with Via-Probe Cell Viability Solution (BD Biosciences) and then analyzed by FACS. After gating live cells, proliferating T cells were identified as percentages of CFSE^–/lo^ among CD3^+^CD4^+^ or CD3^+^CD8^+^ T cells.

Levels of IL-10, IFN-γ, and IL-17A (as pg/mL cells) were assessed in the supernatant of proliferating T cells by ELISA (Thermo Fisher Scientific).

All samples (for both B cell and T cell analyses) were acquired by multicolor flow cytometry (FACS Fortessa X-20, BD Biosciences) and analyzed by FlowJo software, version 10.7.1 (BD Biosciences); conjugated antibodies and cells viability markers are detailed in Supplemental Table 3.

### Statistics.

The sample size was calculated based on the following assumptions: the primary purpose of this study was to demonstrate the safety and tolerability of intravenous MSC infusion and to ascertain preliminary efficacy parameters. The efficacy in reducing INS recurrence was the main outcome for sample size estimation, i.e., the first secondary efficacy endpoint. Patients entering the study were expected to have a 100% rate of INS recurrence after steroid/immunosuppressive therapy tapering/withdrawal. Our working hypothesis was that MSC infusion could decrease the expected rate of recurrence by at least 30%. On the basis of this assumption, the inclusion of 18 patients would confer to the analyses a 90% power to demonstrate a statistically significant reduction (α = 0.05, 1-tailed test) in the incidence of recurrences observed after MSC infusion (70% or less) compared with the 100% expected during treatment tapering/withdrawal without previous MSC infusion. To account for a 10% dropout rate, 20 patients should be included in the study. Due to the COVID-19 pandemic, a variety of logistical difficulties emerged in February 2020, particularly in Bergamo where all face-to-face visits and nonemergency medical procedures were interrupted between February and June 2020. At the onset of the pandemic, all 10 pediatric patients had been treated in Rome, while in Bergamo, 8 young adults had been enrolled and 5 treated. When non-emergency medical procedures were restarted in June 2020, 2 enrolled patients had lost inclusion criteria or acquired an exclusion criterion, while 1 adult patient was treated according to protocol. Therefore, due to the unforeseeable emergency, the study treated 16 patients overall. All of them relapsed during the subsequent observation period. This was discussed internally with our statisticians. Based on our initial sample size calculation, we calculated that the inclusion of 15 patients would confer an 80% power to demonstrate a statistically significant reduction (α = 0.05, 1-tailed test) in the incidence of recurrences observed after MSC infusion (70% or less) compared with the 100% expected during treatment tapering/withdrawal without previous MSC infusion. Therefore, with 16 patients we would have greater than 80% power to demonstrate a statistically significant reduction in the incidence of recurrences after MSC infusion. Considering that this parameter was a secondary end-point of this phase I study, whose primary endpoint comprised safety and feasibility, we concluded enrolment and considered the sample size obtained adequate.

Analyses are presented for the intention-to-treat population, defined as all patients treated with autologous BM-MSCs in the study (*n* = 16), using all available data. Data are summarized using descriptive statistics, detailing the incidence and type of AEs. For the secondary efficacy endpoints, analysis once again included all treated participants. Continuous variables in the baseline characteristics re expressed as mean ± standard deviation (SD) or as median and interquartile range (IQR). Categorical variables are expressed as number and percentage. Follow-up data are expressed as median and range. All *P* values are 2-sided and considered statistically significant with a *P* value of less than 0.05.

The Kaplan-Meier method was used to plot the probability of experiencing a relapse. Survival times for each patient were calculated starting from first MSC infusion to the occurrence of the first relapse of NS. In an exploratory analysis, an unmatched Cox’s regression model and a stratified Cox’s regression for matched pairs between the year before versus the year after of first MSC infusion by PROC PHREG (SAS Institute Inc.), using the matching variable “patient” as the STRATA statement was carried out. Within-group comparisons were made using nonparametric Wilcoxon’s signed-rank test or Fisher’s exact test, as appropriate. The therapeutic efficacy of BM-MSC treatment was also scored by evaluating the sum of number of relapses and number of immunosuppressive drugs in the 12 months preceding and 12 months after BM-MSC treatment for each patient. Mean natural course of systolic blood pressure (SBP), diastolic blood pressure (DBP), mean arterial pressure (MAP), total cholesterol, triglycerides, body weight, height and glucose are presented in table format over time (Table 4).

To assess efficacy in a post hoc analysis, we created a scoring system to integrate in a single measurement the cumulative effects of reducing the number of relapses in the previous and subsequent 12-month periods and the number of immunosuppressive drugs needed to maintain remission at time of BM-MSC infusions and at +12 months. To this end, we simply cumulated these 2 values for each patient at time 0 and at +12 months from BM-MSC infusions.

Analyses and graphical representations of results were carried out using SAS version 9.4 (SAS Institute Inc.), STATA version 15 (StataCorp), and GraphPad Prism 9.0.

We used an electronic case report form (eCRF) to collect data in this study, providing a platform for data insertion in a password-encrypted fashion only to authorized study personnel. After study completion, following written authorization by the study principal investigator, the eCRF database was locked. Extracted data was sent in a password-encrypted fashion to the principal investigator, converted into SAS-compliant format for statistic evaluations, and saved onto a pen drive kept among the study material.

### Data availability.

Underlying data for the manuscript can be accessed in the Supporting Data Values XLS file.

### Study approval.

The protocol and all other trial-related materials were approved by the ethics committee of the promoting center (IRCCS Bambino Gesù Children’s Hospital) and by the Italian regulatory authority (Agenzia Italiana del Farmaco – AIFA and Istituto Superiore di Sanità commission for phase I studies) on July 10, 2018. Only 1 protocol amendment was submitted and approved, relating to the change in principal investigator from GR to PR in Bergamo. Written informed consent was obtained from all adults patients and by the parents or legal representatives for all pediatric patients before commencing study-specific procedures. All study procedures were performed in accordance with the Declaration of Helsinki, implementing good clinical and good medicinal practices.

## Author contributions

MV, FL, and GR conceived the original idea. MV and PR designed the study. MV and MC wrote the first draft of the manuscript and PR, FE, FL, and GR the final version. MT, FC, and MC performed flow cytometry analyses. AP and TP performed the statistical analyses. MV, MI, CC, and MA prepared the investigator brochure and the investigational medicinal product dossier. NR and FZ helped with data handling. SR, FZ, ILE, and MB included, managed, and monitored the patients. ADS reviewed and scored the quality of life questionnaires. All authors had full access to study data, contributed to data interpretation, and critically revised the manuscript. MV was the principal investigator of the study and was responsible for manuscript preparation and decision to submit for publication.

## Supplementary Material

Supplemental data

ICMJE disclosure forms

Supporting data values

## Figures and Tables

**Table 1:**
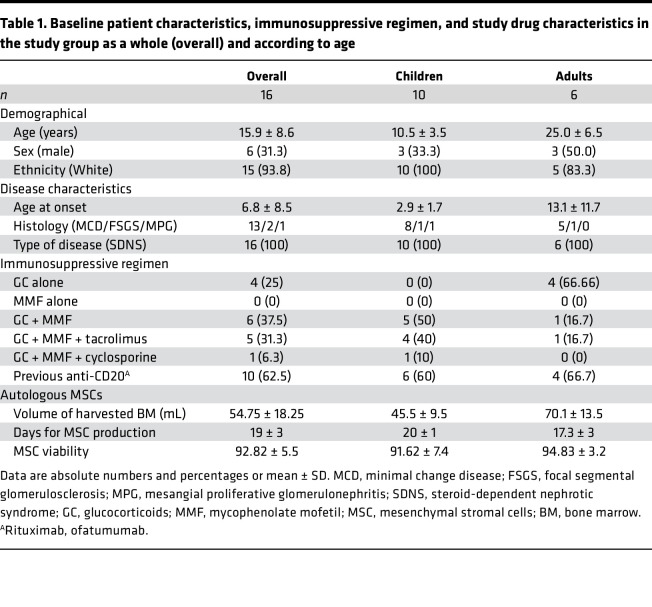
Baseline patient characteristics, immunosuppressive regimen, and study drug characteristics in the study group as a whole (overall) and according to age

**Table 2:**
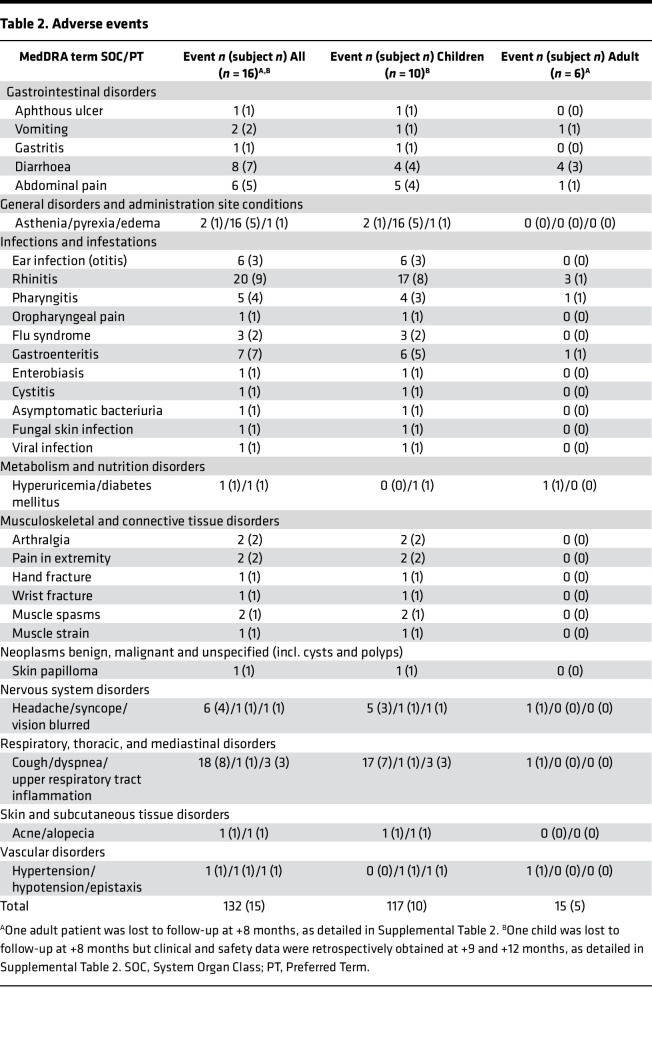
Adverse events

**Table 3:**
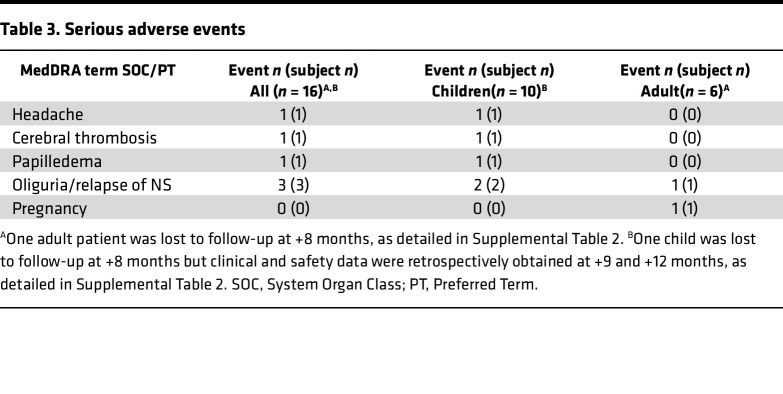
Serious adverse events

**Table 4:**
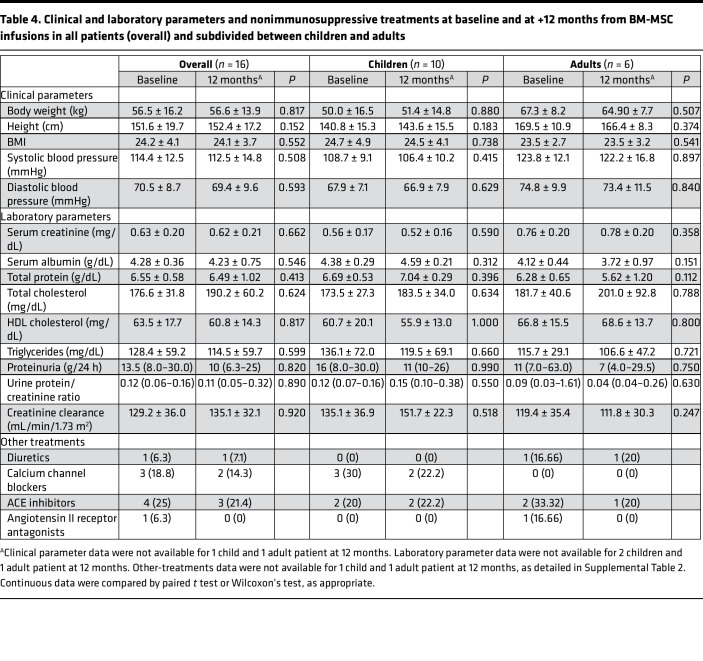
Clinical and laboratory parameters and nonimmunosuppressive treatments at baseline and at +12 months from BM-MSC infusions in all patients (overall) and subdivided between children and adults

## Version 1 08/10/2023

In-Press Preview

## Version 2 09/22/2023

Electronic publication
