# Supplementary material for: A phase I study of autologous mesenchymal stromal cells for severe steroid-dependent nephrotic syndrome
Source: JCI Insight. 2023 Sep 22;8(18):e169424. doi: 10.1172/jci.insight.169424 (PMC10561718; doi:10.1172/jci.insight.169424)
Supplement: Supplemental data [file jciinsight-8-169424-s053.pdf]

Supplementary material

A phase 1 study of autologous mesenchymal stromal cells for severe steroid-dependent nephrotic syndrome

Marina Vivarelli,, Manuela Colucci, Mattia Algeri, Federica Zotta, Francesco Emma, Ines L'Erario, Marco Busutti, Stefano Rota, Chiara Capelli , Martino Introna, Marta Todeschini, Federica Casiraghi, Annalisa Perna, Tobia Peracchi, Andrea De Salvo, Nadia Rubis, Franco Locatelli,, Giuseppe Remuzzi, Piero Ruggenenti,

Figure S1: Composite score of autologous BM-MSC treatment efficacy (post-hoc analysis): **page 2**

Figure S2: Gating strategy for flow cytometry analysis: **page 3**

Table S1: Circulating levels and functional assessments of the different B- and T-cell subsets in the study group considered as a whole and in children and adults considered separately during the follow-up: **page 4**

Table S2. Patient visits: **page 7**

Table S3. List of antibodies used for flow cytometry analysis. **page 8**

Study protocol: page 9

IB (investigator brochure): page 61

IMPD (investigational medicinal product description): page 132

Supplemental material

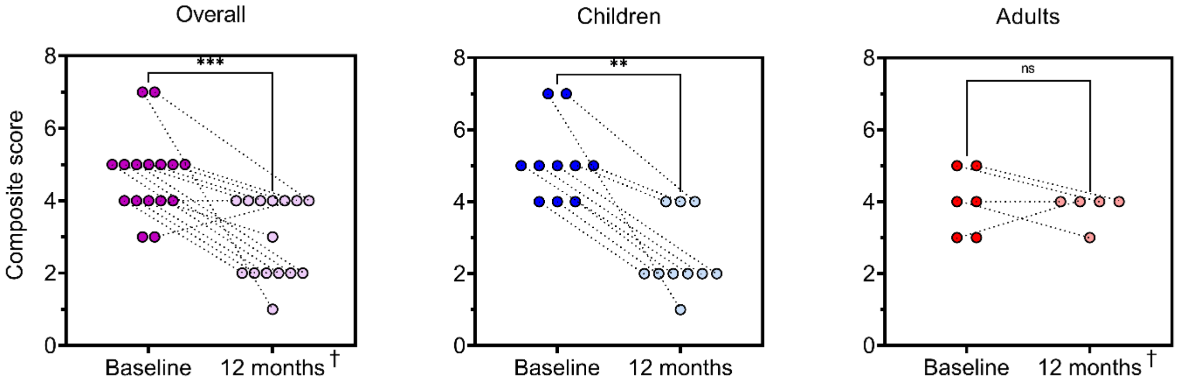

Figure S1: Composite score of autologous BM-MSC treatment efficacy (post-hoc analysis)

Single patient representation of treatment efficacy using a cumulative score of number of relapses in the 12 previous months + number of immunosuppressive drugs at baseline (full dots) and of number of relapses in the 12 months post-BM-MSC infusions + number of immunosuppressive drugs at +12 months (shaded dots) shown overall (purple, left panel), in children (blue, central panel) and in adults (red, right panel). Data were compared by Wilcoxon test.

**, p<0.01; ***, p<0.001; ns= not significant; †, one adult patient was lost to follow-up at +8 months, detailed in Table S2.

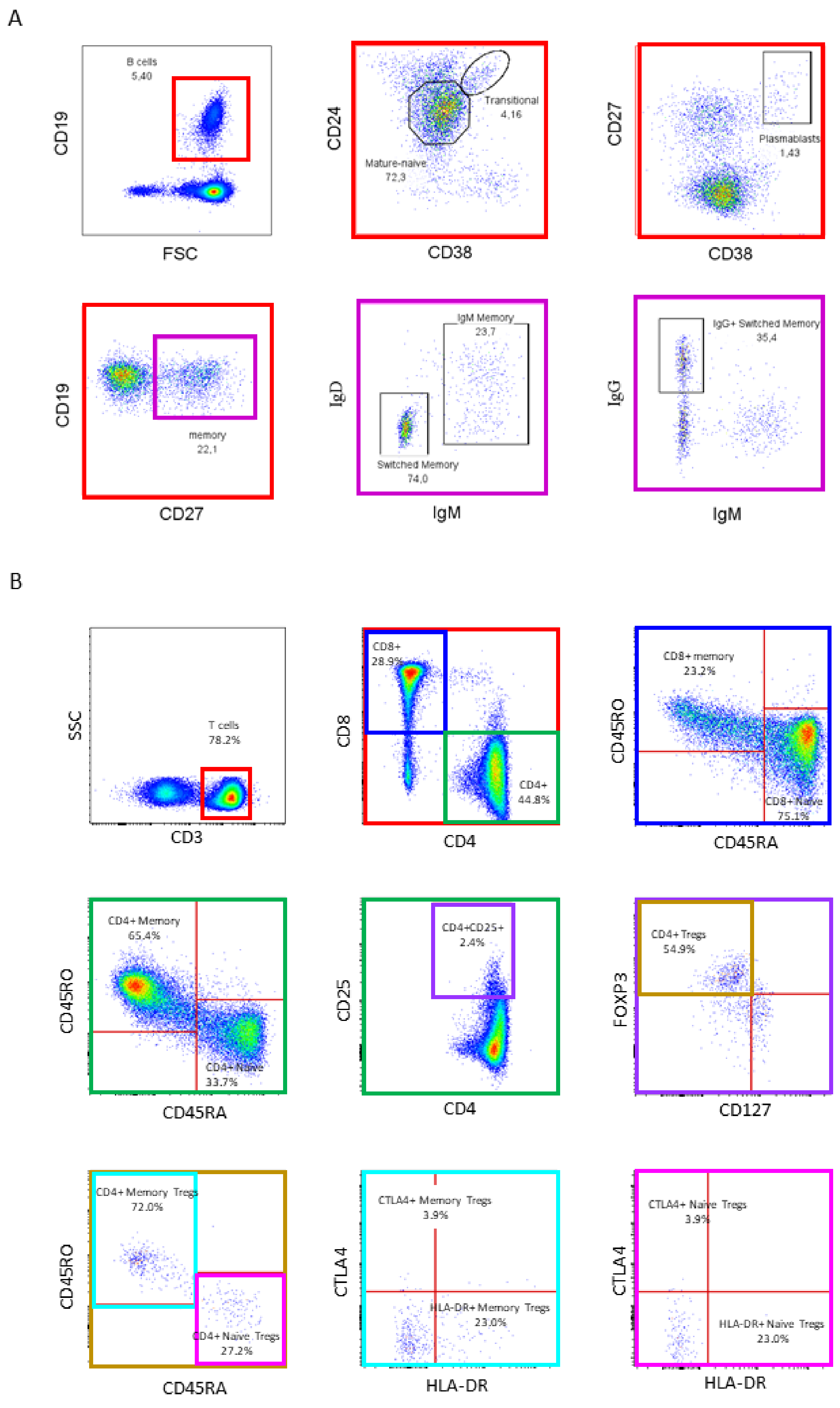

Figure S2: Gating strategy for flow cytometry analysis. A: Gating strategy for B-cell phenotype. B: Gating strategy for T-cell phenotype. Representative pseudocolour plots from a single patient are shown.

Table S1: Circulating levels and functional assessments of the different B- and T-cell subsets in the study group considered as a whole and in children and adults considered separately during the follow-up.

		**Overall**						**Children**						**Adults**					
**Time (months)**		**0**	**1**	**3**	**6**	**9**	**12**	**0**	**1**	**3**	**6**	**9**	**12**	**0**	**1**	**3**	**6**	**9**	**12**
	N	16	16	16	15	10	11	10	10	10	10	8	8	6	6	6	5	2	3
**B cell subsets (count/μl) and function**																			
**$CD19^+$**	**Median**	**352**	**293**	**233**	**296**	**345**	**408**	**332**	**349**	**233**	**368**	**519**	**374**	**409**	**271**	**208**	**262**	**250**	**450**
	P vs 0m			*						*									
	P vs 1m																		
**Transitional**	**Median**	**7.1**	**7.0**	**10.5**	**14.2**	**19.9**	**14.9**	**14.2**	**21.1**	**17.3**	**28.6**	**26.5**	**19.8**	**3.4**	**3.0**	**2.8**	**2.6**	**4.2**	**1.2**
	P vs 0m										*								
	P vs 1m										*								
**Mature**	**Median**	**220**	**188**	**102**	**189**	**198**	**210**	**186**	**149**	**101**	**181**	**231**	**184**	**303**	**233**	**148**	**200**	**116**	**250**
	P vs 0m			**						*									
	P vs 1m			*						**									
**Total memory**	**Median**	**38.7**	**41.0**	**38.8**	**40.1**	**67.3**	**68.6**	**45.5**	**49.5**	**40.3**	**60.3**	**67.3**	**66.3**	**30.7**	**25.8**	**29.5**	**31.6**	**94.5**	**75.8**
	P vs 0m																		
	P vs 1m									*									
**IgM memory**	**Median**	**19.0**	**18.6**	**16.6**	**16.1**	**43.6**	**31.7**	**27.3**	**25.5**	**22.9**	**33.4**	**43.6**	**32.8**	**10.0**	**8.5**	**10.3**	**15.4**	**36.3**	**31.3**
	P vs 0m																		
	P vs 1m																		
**Switched memory**	**Median**	**17.5**	**21.0**	**14.5**	**21.7**	**28.1**	**31.3**	**16.6**	**21.0**	**14.5**	**19.4**	**28.1**	**30.4**	**20.3**	**17.9**	**14.5**	**21.7**	**55.2**	**41.3**
	P vs 0m			*															
	P vs 1m									*									
**IgG switched memory**	**Median**	**9.97**	**9.29**	**7.69**	**13.4**	**17.1**	**19.4**	**9.72**	**9.29**	**7.16**	**10.9**	**17.1**	**17.9**	**12.0**	**9.62**	**8.11**	**13.4**	**35.8**	**27.6**
	P vs 0m			*															
	P vs 1m			*						*									
**Plasmablasts**	**Median**	**1.4**	**1.7**	**1.8**	**2.3**	**3.2**	**1.8**	**1.4**	**1.7**	**1.8**	**3.1**	**5.2**	**1.7**	**2.3**	**1.3**	**3.2**	**1.4**	**0.6**	**1.8**
	P vs 0m			*															
	P vs 1m																		
**% CD19+ proliferation**	**Median**	**80.5**	**82.0**	**80.7**	**83.0**	**74.2**	**80.8**	**74.8**	**81.8**	**82.6**	**83.0**	**80.4**	**79.8**	**74.8**	**82.7**	**75.1**	**81.3**	**30.3**	**83.8**
	P vs 0m																		
	P vs 1m																		
**Serum immunoglobulin (g/l)**																			
**IgG**	**Median**	**6.9**	**6.4**	**7.2**	**6.7**	**8.0**	**6.9**	**7.1**	**7.2**	**7.2**	**9.0**	**9.0**	**7.6**	**6.9**	**6.3**	**7.4**	**6.1**	**6.4**	**6.3**
	P vs 0m																		
	P vs 1m																		
**IgA**	**Median**	**1.2**	**1.2**	**1.1**	**1.4**	**1.1**	**1.3**	**1.0**	**1.1**	**1.0**	**1.1**	**0.9**	**1.1**	**1.5**	**1.5**	**1.6**	**2.5**	**2.2**	**2.5**
	P vs 0m																		
	P vs 1m																		

**IgM**	**Median**	**0.7**	**0.7**	**0.7**	**1.3**	**0.8**	**0.6**	**0.8**	**0.8**	**0.5**	**1.3**	**0.8**	**1.1**	**0.7**	**0.7**	**1.0**	**1.0**	**0.5**	**0.6**
	P vs 0m																		
	P vs 1m																		
**T-cell subsets (count/µl) and function**																			
**$CD3^+$**	**Median**	**1940**	**1696**	**1746**	**1968**	**1599**	**2074**	**1605**	**1696**	**1650**	**1913**	**1691**	**1862**	**2381**	**1483**	**2065**	**2003**	**1466**	**2575**
	P vs 0m																		
	P vs 1m																		
**$CD4^+$**	**Median**	**960**	**999**	**952**	**1020**	**920**	**1105**	**960**	**1090**	**915**	**1027**	**1051**	**992**	**1138**	**826**	**1061**	**999**	**785**	**1309**
	P vs 0m																		
	P vs 1m									*									
**$CD8^+$**	**Median**	**668**	**576**	**667**	**705**	**570**	**756**	**560**	**576**	**488**	**642**	**484**	**505**	**1026**	**582**	**851**	**827**	**614**	**1158**
	P vs 0m																		
	P vs 1m																		
**Naïve $CD4^+$**	**Median**	**377**	**493**	**448**	**396**	**468**	**477**	**392**	**571**	**462**	**462**	**655**	**525**	**377**	**260**	**425**	**375**	**252**	**444**
	P vs 0m																		
	P vs 1m									**									
**Memory**	**Median**	**454**	**475**	**465**	**515**	**447**	**662**	**429**	**475**	**405**	**391**	**441**	**405**	**621**	**452**	**519**	**536**	**476**	**745**
**$CD4^+$**	P vs 0m																		
	P vs 1m																		
**Naïve $CD8^+$**	**Median**	**435**	**369**	**371**	**400**	**396**	**421**	**409**	**374**	**304**	**387**	**374**	**379**	**644**	**316**	**536**	**459**	**420**	**674**
	P vs 0m																		
	P vs 1m																		
**Memory**	**Median**	**181**	**148**	**170**	**187**	**148**	**218**	**145**	**140**	**145**	**145**	**127**	**145**	**308**	**246**	**303**	**328**	**256**	**463**
**$CD8^+$**	P vs 0m																		
	P vs 1m																		
**$CD4^+$ Tregs**	**Median**	**12.5**	**10.5**	**16.5**	**13**	**15**	**13.5**	**10**	**12**	**16.5**	**18.5**	**23**	**16.5**	**13.5**	**10.5**	**18.5**	**12**	**11**	**9.5**
	P vs 0m										*								
	P vs 1m										*								
**Resting $CD4^+$**	**Median**	**2.5**	**3.5**	**5**	**3**	**5**	**4.5**	**2.5**	**5**	**5**	**4.5**	**6**	**4.5**	**2.5**	**2**	**5**	**2**	**1**	**3**
**Tregs**	P vs 0m																		
	P vs 1m																		
**Memory**	**Median**	**6.5**	**7**	**9.5**	**10**	**10**	**8.5**	**5.5**	**6.5**	**8.5**	**11**	**10**	**8.5**	**10**	**7.5**	**13**	**8**	**6**	**8**
**$CD4^+$ Tregs**	P vs 0m			*						**	*								
	P vs 1m			**							*								
**HLA-DR^+**	**Median**	**2.3**	**2.5**	**3.95**	**4.4**	**4.1**	**4.15**	**2.2**	**2.35**	**3.65**	**4.5**	**5.35**	**4.35**	**4.8**	**2.85**	**5.05**	**3.6**	**2.5**	**3.8**
**Memory**	P vs 0m			**						**	*								
**$CD4^+$ Tregs**	P vs 1m			**						*	**								
**$CTLA4^+$**	**Median**	**0.5**	**0.5**	**0.7**	**0.7**	**0.6**	**0.6**	**0.5**	**0.5**	**0.6**	**0.75**	**0.6**	**0.6**	**0.55**	**0.4**	**0.95**	**0.6**	**0.5**	**0.3**
**Memory**	P vs 0m			*												*			
**$CD4^+$ Tregs**	P vs 1m			**							*					*			

**% $CD4^+$ proliferation**	**Median**	**65.8**	**66.0**	**66.1**	**63.2**	**66.9**	**56.3**	**70.1**	**71.8**	**71.9**	**64.9**	**71.5**	**60.8**	**54.8**	**57.8**	**54.0**	**56.0**	**62.8**	**56.3**
	P vs 0m																		
	P vs 1m																		
**% $CD8^+$ proliferation**	**Median**	**76.1**	**77.4**	**74.4**	**72.4**	**77.3**	**73.6**	**78.5**	**77.5**	**77.6**	**73.4**	**74.2**	**70.3**	**71.5**	**70.9**	**64.0**	**71.5**	**77.3**	**77**
	P vs 0m																		
	P vs 1m																		
**Cytokines from stimulated T cells (pg/ml)**																			
**IL-10**	**Median**	**31.3**	**51.0**	**58.8**	**86.6**	**32.5**	**45.3**	**35.6**	**53.4**	**77.1**	**87.3**	**18.0**	**41.2**	**28.8**	**43.2**	**54.8**	**56.3**	**44.4**	**47.3**
	P vs 0m																		
	P vs 1m																		
**IFN-γ**	**Median**	**770**	**781**	**688**	**704**	**624**	**705**	**738**	**721**	**690**	**704**	**619**	**592**	**857**	**1376**	**651**	**790**	**647**	**862**
	P vs 0m																		
	P vs 1m																		
**IL-17A**	**Median**	**988**	**1329**	**1030**	**1234**	**1063**	**1577**	**842**	**1217**	**974**	**1149**	**1085**	**3303**	**1211**	**2801**	**1991**	**1930**	**985**	**1270**
	P vs 0m																		
	P vs 1m			*															

P values vs baseline (0m) or vs 1 month after MSC infusion (1m) were calculated by Wilcoxon-test only when data were available for all patients of each subgroup (n=16, n=10, n=6, for whole patients, children and adults, respectively). *, $p<0.05$; ** $p<0.01$.

Table S2. Patient visits.

**Visit number**	**0**	**1**	**2**	**3**	**4**	**5**	**6**	**7**	**8**	**9**	**10**	**11**	**12**	**13**	**14**	**15**	**16**
Time[1]	Enrollment	-1-3 m	0	1 d	8 d	1 m	2 m	3 m	4 m	5 m	6 m	7 m	8 m	9 m	10 m	11 m	12 m
RM01	x	x	x	x	x	x	(x)	x	(x)	(x)	x	(x)	(x)	x	(x)	(x)	x
RM02	x	x	x	x	x	x	(x)	x	(x)	(x)	x	(x)	(x)	x	(x)	(x)	x
RM03	x	x	x	x	x	x	(x)	x	(x)	(x)	x	(x)	(x)	x	(x)	(x)	x
RM04	x	x	x	x	x	x	(x)	x	(x)	(x)	x	(x)	(x)	x	(x)	(x)	x
RM05	x	x	x	x	x	x	(x)	x	(x)	(x)	x	(x)	(x)	x	(x)	(x)	x
RM06	x	x	x	x	x	x	(x)	x	(x)	(x)	x	(x)	(x)	*	-	-	*
RM07	x	x	x	x	x	x	(x)	x	(x)	(x)	x	(x)	(x)	x	x	(x)	X
RM08	x	x	x	x	x	x	(x)	x	(x)	(x)	x	(x)	(x)	(x)	-	-	(x)
RM09	x	x	x	x	x	x	(x)	x	(x)	(x)	x	(x)	(x)	x	(x)	(x)	X
RM10	x	x	x	x	x	x	(x)	x	(x)	(x)	x	(x)	(x)	x	(x)	(x)	X
BG01	x	x	x	x	x	x	(x)	x	(x)	(x)	x	(x)	(x)	x	(x)	(x)	X
BG02	x	x	x	x	x	x	(x)	x	(x)	(x)	x	(x)	(x)	x	(x)	(x)	X
BG03	x	x	x	x	x	x	(x)	x	(x)	(x)	-	(x)	(x)	-	-	-	-
BG04	x	x	-	-	-	-	-	-	-	-	-	-	-	-	-	-	-
BG05	x	x	x	x	x	x	(x)	x	(x)	(x)	x	(x)	(x)	x	(x)	(x)	X
BG06	x	x	x	x	x	x	(x)	x	(x)	(x)	x	(x)	(x)	x	(x)	(x)	X
BG07	x	x	x	x	x	x	(x)	x	(x)	(x)	x	(x)	(x)	x	(x)	(x)	X
BG08	x	-	-	-	-	-	-	-	-	-	-	-	-	-	-	-	-

1 **Legend**: “d” means days and “m” means months. “x” means that the visit was in person, “(x)” means that the visit was a telephone call, as detailed in the study protocol. * means that a subsequent telephone call gave information relating to safety, relapses and immunosuppression at these timepoints. Visit 3,4: MSC infusions

Table S3. List of antibodies used for flow cytometry analysis.

Antibody and viability cell markers	Clone	Fluorochrome
Mouse anti-human CD19	SJ25C1	PECy7
Mouse anti-human CD24	ML5	PE
Mouse anti-human CD38	HIT2	PerCP-Cy5.5
Mouse anti-human CD27	L128	APC
Mouse anti-human CD45	2D1	V500-C
Mouse anti-human IgM	G20-127	FITC
Mouse anti-human IgD	IA6-2	V450
Mouse anti-human IgG	G18-145	APC-H7
mouse anti-human CD3	HIT3a	BV510
mouse anti-human CD4	SK3	BB700
mouse anti-human CD25	M-A251	BV421
mouse anti-human HLA-DR	G46-6	BV605
mouse anti-human CD8	RPA-T8	BV711
mouse anti-human CD45RO	UHCL1	BV786
mouse anti-human FOXP3	236A/E7	AF647
mouse anti-human CD127	HIL-7R-M21	APC-R700
mouse anti-human CD152	BIN3	PE-Cy5
mouse anti-human CD45RA	HI100	PE-Cy7
mouse anti-human CD45	HI30	BUV395
mouse anti-human CD28	CD28.2	BUV737
Fixable Viability Stain 520	-	FVS520
Via Probe Cell Viability Solution	-	7-AAD

Supplementary File 1: IMPD (investigational medicinal product description)

Supplementary File 2: IB (investigator brochure)

Supplementary File 3: Study protocol

Bambino Gesù
OSPEDALE PEDIATRICO

A prospective study to assess safety and efficacy of the use of bone-marrow derived MESenchymal stromal cells as immunomodulatory therapy for children and young adults with severe and difficult-to-treat frequently relapsing or steroid-dependent idiopathic NEPHrotic syndrome: the MESNEPH study

Open label phase 1 trial

Prepared by
Marina Vivarelli MD, Franco Locatelli MD, Giuseppe Remuzzi MD, Francesco Emma MD, Martino Introna MD, Norberto Perico MD, Mattia Algeri MD, Federica Casiraghi PhD, Rita Carsetti MD PhD

Confidential Statement

This protocol contains strictly confidential information

which is not to be communicated or published unless

previously authorized by

Bambino Gesù Children’s Hospital, IRCCS

**Protocol n.:**	1262_OPBG_2018
**Title:**	A prospective study to assess safety and efficacy of the use of bone-marrow derived MESenchymal stromal cells as immunomodulatory therapy for children and young adults with severe and difficult-to-treat frequently relapsing or steroid-dependent idiopathic NEPHrotic syndrome: the MESNEPH study
**Brief Title:**	MSC as therapy for INS: phase 1 study
**EudraCT Number**	2016-004804-77
**Phase**	1
**Study Design:**	Multicentric, open-label, prospective, non-randomized study
**Objective:**	The primary end-point of the study is to test the safety of autologous mesenchymal stromal cells for the treatment of steroid-dependent nephrotic syndrome.<br>The secondary endpoints are:<br>- Recurrence of INS.<br>- The dose of immunosuppressive therapy to prevent further INS relapses;<br>- Adverse effects of immunosuppressive therapy (arterial hypertension and need for antihypertensive therapy, obesity and impaired glucose tolerance, dyslipidemia, renal dysfunction, stunted statural growth, infections)<br>- Kidney function at baseline and at one year after MSC administration
**Subject number:**	20
**Study End:**	40 months
**Product:**	Autologous mesenchymal stromal cells (MSC)
**Pharmaceutical Form:**	Cell suspension
**Dosage:**	1 x 10^6 cells/kg at day 1 and day 8
**Control Drug:**	None
**Type of Study**	Multicentric

**P.I./Coordinator Centre:**	Dr. Marina Vivarelli<br>Ospedale Pediatrico "Bambino Gesù" di Roma (Istituto di Ricovero e Cura a Carattere Scientifico)
**Participating Centres**	**COORDINATING CENTER**: IRCCS Ospedale Pediatrico Bambino Gesù, Rome<br>Division of Nephrology and Dialysis: Dr Marina Vivarelli, Principal Investigator; Prof Francesco Emma<br>Division of Oncohematology: Dr Mattia Algeri, Prof Franco Locatelli<br>Cell Factory: Dr Franca Fassio<br>Research Laboratories: Prof Rita Carsetti<br>**OPERATIVE UNIT 1**: Azienda Socio Sanitaria Territoriale Papa Giovanni XXIII<br>Unit of Nephrology and Dialysis: Dr Stefano Rota, Dr Valentina Portalupi, Dr Alessia Gennarini, Prof Giuseppe Remuzzi<br>Cell Therapy Laboratory "G. Lanzani"- Unit of Hematology: Prof Martino Introna<br>**OPERATIVE UNIT 2**:<br>Research Laboratories Clinical Research Center for Rare Diseases - IRCCS Istituto di Ricerche Farmacologiche Mario Negri, Bergamo: Dr Federica Casiraghi
**Sponsor:**	Ospedale Pediatrico "Bambino Gesù" di Roma (Istituto di Ricovero e Cura a Carattere Scientifico)

LIST OF ABBREVIATIONS

ACTH	Adrenocorticotrophic Hormone
AE	Adverse Event
AIFA	Agenzia Italiana del Farmaco
ASST-PG23	Azienda Socio Sanitaria Territoriale Papa Giovanni XXIII
BM	Bone Marrow
BMI	Body mass index
BM-MNC	Bone Marrow-derived MonoNuclear Cells
BM-MSC	Bone Marrow-derived Mesenchymal Stromal Cells
CCL18	Chemokine ligand 18
CRF	Case Report Form
CTMP	Cell Therapy Medicinal Product
DL	Decreto Legislativo
DM	Data Management
DM	Decreto Ministeriale
EMA	European Medicines Agency
FDA	Food and Drug Administration
FRNS	Frequently relapsing nephrotic syndrome
GCP	Good Clinical Practice
GMP	Good Manufacturing Practice
GVHD	Graft-versus-host disease
HD	Healthy Donor
HO	Haemoxigenase
IB	Investigator Brochure
ICF	Informed consent form
ICH	International Council for Harmonisation of Technical Requirements for Pharmaceuticals for Human Use
IDO	Indoleamine 2,3-dioxygenase
IFNγ	Interferon gamma
IL-10	Interleukin10
IL-2	Interleukin 2
IL-6	Interleukin 6
IMPD	Investigator Medicinal Product Description
INS	Idiopathic nephrotic syndrome
IRB	Institutional Review Board
IRCCS	Istituto di Ricovero e Cura a Carattere Scientifico
IRFMN	IRCCS Istituto di Ricerche Farmacologiche “Mario Negri”

ISF	Investigator Site File
ISKDC	International Study of Kidney Disease in Children
ISS	Istituto Superiore di Sanità
iv	intravenous
MSC	Mesenchymal stromal cells
N2	nitrogen
NK	natural killer
NSAE	Non Serious Adverse Event
OPBG	Ospedale Pediatrico Bambino Gesu'- IRCCS
PBMC	Peripheral Blood Mononuclear Cells
PDN	Prednisone
PGE2	Prostaglandin E2
SAE	Serious Adverse Event
SDNS	Steroid-dependent nephrotic syndrome
SRNS	Steroid-resistant nephrotic syndrome
SSNS	Steroid-sensitive nephrotic syndrome
TGFβ	Transforming growth factor beta
TNF	Tumor necrosis factor
TREG	T regulatory cells

DEFINITIONS[1]

Nephrotic Syndrome

- edema
- massive proteinuria (>40 mg/m^2/hr in children or urine protein/creatinine ratio > 2 g/g, >3.5 g/day in adults)
- hypoalbuminemia (<2.5 g/dl)

Remission

- resolution of edema
- normalization of serum albumin (≥3.5 g/dl)
- marked reduction in proteinuria
  - complete remission (<4 mg/m^2/hr or negative dipstick or urine protein/creatinine ratio < 0.2 g/g in children, < 0.3 g/day in adults)
  - partial remission (<2 g/1.73 m^2/day, decreased by 50% and serum albumin ≥2.5 g/dl in children, <3.5 g/day and decreased by 50% in adults)

Relapse

- recurrence of massive proteinuria (>40 mg/m^2/hr in children or urine protein/creatinine ratio > 2 g/g, > 3.5 g/day in adults)
- positive urine dipstick (≥3+ for 3 days or positive for 7 days, usually applicable to children)
- ± edema

Steroid Sensitive Nephrotic Syndrome (SSNS)

Response to PDN 60mg/m^2/day within 4-6 weeks ± MPD boluses in children

Response to PDN 1mg/kg/day or 2mg/kg/every other day within 16 weeks in adults

Frequently Relapsing Nephrotic Syndrome (FRNS)

≥2 relapses per 6 months (or ≥4 relapses per 12 months)

Steroid Dependent Nephrotic Syndrome (SDNS)

Relapse during steroid therapy or within 15 days of discontinuation

Refractory or persistent nephrotic syndrome

Absence of partial remission after 6 months OR absence of complete remission after 2 years. Treatment consists of per center practice for steroid-resistant forms, including methylprednisolone boluses, oral prednisone, calcineurin inhibitors and, in some cases, rituximab.

Steroid-resistant nephrotic syndrome (SRNS)

Children: no response to PDN 60 mg/m^2 per day within 4 weeks ± methylprednisolone boluses in children

Adults: no response to PDN 1 mg/kg per day or 2 mg/kg every other day within 16 weeks in adults.

Stunted statural growth (children)

A fall from the statural growth centile that the patient was on pre-treatment to at least one SDS below, persisting at least 6 months corresponding to a growth velocity below the 25°th centile for sex and age in that time period.

Osteoporosis (adults) and reduced bone mineral density (children)

Children: Z score evaluated at the lumbar spine at least 2 SDS below normal range

Adults: T score evaluated at the lumbar spine at least 2 SDS below normal range.

List of genetic mutations that preclude enrolment in the MESNEPH trial: NPHS1, NPHS2, WT1, ACTN4, LAMB2, PLCε1, CD2AP, SMARCAL1, COQ6, TRPC6, LMX1B, INF2, MYO1E, PTPRO, SCARB2, ITGA3.

TABLE OF CONTENTS

Study title and organization pg. 3

List of abbreviations pg. 5

Definitions pg. 7

Table of contents pg. 9

1. Background and rationale pg. 12
   - 1.1 Idiopathic nephrotic syndrome pg. 12
   - 1.2 Therapeutic approach to INS: unmet clinical needs pg. 12
   - 1.3 Mesenchymal stromal cells pg. 13
   - 1.4 Potential for the therapeutic use of mesenchymal stromal cells in idiopathic nephrotic syndrome: in vitro results pg. 15
   - 1.5 Conclusions pg. 17
2. Investigational medicinal product benefit and risk assessment pg. 18
   - 2.1 Investigational medicinal product pg. 18
   - 2.2 Risks pg. 18
   - 2.3 Potential benefits pg. 19
   - 2.4 Comparison to standard therapeutic approach pg. 22
   - 2.5 Summary of benefit/risk assessment pg. 22
3. Study objectives pg. 23
   - 3.1 Primary objectives pg. 23
   - 3.2 Secondary objectives pg. 23
   - 3.3 Primary end-point pg. 23
   - 3.4 Secondary end-points pg.23
4. Investigational plan pg. 24
   - 4.1 Overview of trial pg. 24
   - 4.2 Study timeline pg. 24
   - 4.3 Patient selection pg. 25
   - 4.4 Interim analysis pg.26

4.5 Premature study discontinuation pg.26

5. Patient withdrawal pg. 27

6. Treatment pg. 27

6.1 Description of medicinal product pg. 27

6.2 Preparation of cellular product pg. 27

6.3 Dosing regimen pg. 28

6.4 Administration and concomitant monitoring pg. 29

6.5 Post-administration monitoring pg. 29

6.6 Concomitant medication pg. 29

7. Study assessments pg. 30

Study Enrollment (Visit 0) pg.31

Visit 1 pg. 32

Visit 2, 3, 4 pg. 32

Visit 5-16 pg. 33

Table 1 pg. 34

Table 2 pg. 35

8. Safety management pg. 36

8.1 Adverse events pg. 36

8.2 Definition of a serious adverse event (SAE) pg. 36

8.3 Clarification of severe and serious adverse events pg. 36

8.4 Causality assessment pg. 37

8.5 Definition of severity pg. 37

8.6 Documenting adverse events pg. 38

8.7 Follow-up of adverse events and serious adverse events pg. 38

8.8 IRB notification of SAEs pg. 38

8.9 Post-study reporting requirements pg. 38

9. Statistical methods pg. 39

10. Study administration pg. 40

10.1 Data collection and management pg. 40

10.2 Retention of study records pg. 40

10.3 Confidentiality pg. 40
10.4 Documentation of study results pg. 40
10.5 Use of study results pg. 41
11. Regulatory and ethical considerations pg. 41
11.1 General Principles pg. 41
11.2 Informed consent pg. 42
11.3 Payment to subjects pg. 42
11.4 Timing pg. 42
12. Bibliography pg. 43
Additional File 1: Depletion of memory B cells and hypogammaglobulinemia in patients with idiopathic nephrotic syndrome treated with rituximab pg. 46
Additional file 2: Immunomonitoring pg. 49

1 BACKGROUND AND RATIONALE

1.1 Idiopathic nephrotic syndrome

Idiopathic nephrotic syndrome (INS) is the most frequent glomerular disease in children (1,2). The incidence is around 1-3/100.000 children below 16 years of age. Onset occurs mostly between the ages of 2 and 7 with a slight preponderance in males, often following an infectious episode, classically of the upper respiratory tract. Its clinical manifestation is edema, and laboratory exams reveal intense proteinuria, leading to hypoalbuminemia and therefore to intravascular volume depletion and oliguria. All children at onset are treated with a standard course of oral prednisone, to which the majority (75-90%) respond within a few days/weeks. Patients who do not respond are defined as steroid resistant, and about half of these will subsequently respond in a variable amount of time, up to about 12 months, to second-line immunosuppression. In those who maintain steroid-resistance often a genetic defect of podocyte components leading to disruption of the integrity of the glomerular basement membrane is the cause of disease. All forms that are steroid-sensitive are instead defined as INS, to indicate that the mechanism determining proteinuria is not fully elucidated (2). However, a vast body of experimental evidence, coupled with the clinical response of INS to immunosuppressive therapy, clearly implicates the immune system in disease pathogenesis, through its production of factors that damage the podocyte (3). T cells have been implicated in this process, and a reduction of T regulatory cells (TREG) has been shown (4). The role of B cells, in particular memory B cells, is suggested by the effectiveness of their depletion by anti-CD20 therapy in preventing relapses (5-6).

Most patients have a relapsing course of INS that is treated with corticosteroids, mostly with prednisone (PDN). The frequency of relapses permits the classification of INS in subtypes reflecting disease severity. These include “non relapsing nephrotic syndrome”, “infrequently relapsing nephrotic syndrome”, “frequently relapsing nephrotic syndrome” (FRNS) and “steroid-dependent nephrotic syndrome” (SDNS) (1). This classification has been shown to have a prognostic value that exceeds the relevance of renal pathology findings. Accordingly, a renal biopsy is not indicated in most cases. If performed, the majority of patients have so-called “minimal change disease”, in which changes on light microscopy are minor or absent. Recently, four studies have highlighted that the rapidity of the initial response to corticosteroid treatment represents an important prognostic indicator for children with INS. Specifically, these studies have shown that children who achieve remission in more than 7 days at disease onset are more likely to have a frequently relapsing or steroid-dependent course (7). In a recent review of the long-term evolution of 103 Italian children with steroid-sensitive INS that were treated since their first episode at the "Ospedale Pediatrico Bambino Gesù”, 67% of patients were found to have FRNS or SDNS (7); these data are in agreement with previous studies that were performed in the 1980's. In a small but significant group of patients (around 15-20% of relapsing forms) the disease is severe and prolonged, lasting into adulthood.

1.2 Therapeutic approach to idiopathic nephrotic syndrome: unmet clinical needs

Corticosteroids have been used to treat childhood nephrotic syndrome since 1950 when large doses of adrenocorticotrophic hormone (ACTH) and cortisone given for two to three weeks were found to induce diuresis with loss of edema and proteinuria (2). Since then, oral corticosteroids are the first-line treatment of a child presenting with INS (no controlled prospective trials of corticosteroids compared to placebo have been carried out). However, corticosteroids are known to have adverse effects such as obesity, stunted linear growth, hypertension, diabetes mellitus, osteoporosis, cataract, cushingoid

features and adrenal suppression (2). Adverse effects are particularly prevalent in those children who relapse frequently and thus require multiple courses of steroids. For these patients, the challenge resides in finding a therapeutic strategy that allows maximal steroid sparing while limiting the relapse rate, based on the child's clinical response and drug-related adverse effects. The original treatment schedules for childhood INS were developed in an *ad hoc* manner. The International Study of Kidney Disease in Children (ISKDC) was established in 1966 and determined by consensus a regimen for the first episode of daily prednisone (PDN 60 mg/m^2) for four weeks, followed by PDN given on alternate days for four weeks (40 mg/m^2). In our experience, approximately 50% of children relapse within 3 months with this regimen. Most importantly, the bulk of data that has accumulated over the past decades indicates that corticosteroids are not disease-modifying drugs, i.e. they have little effect on the long-term evolution of the disease.

If toxicity develops, alternative drugs are generally introduced, including calcineurin inhibitors (cyclosporin A or tacrolimus), mycophenolate mofetil and cyclophosphamide. Chronic immunosuppression, however, seldom achieves persistent remission and is invariably burdened by serious adverse effects including gonadotoxicity and sterility, opportunistic infections, malignancies, bone marrow depression and renal toxicity. In our experience, these medications need to be used in more than 50% of children within 5 years of their disease (1).

Eventually, most patients achieve permanent remission, but 20-25% still relapse in adulthood (2). In the last decade, the use of anti-CD20 monoclonal antibodies, which temporarily deplete circulating B cells, has proved effective in the treatment of a substantial number of children with INS, most often frequently relapsing and steroid-dependent forms (5-6). We and others have shown that following this treatment reconstitution of a subgroup of circulating B cells, namely switched memory B cells, predicts relapse (8,9). Moreover, in nephrotic syndrome and more extensively in other immune-mediated diseases where it is routinely employed, some patients post-rituximab experience prolonged hypogammaglobulinemia and maintain low levels of memory B cells for many years following treatment (9,10).

Therefore, in a small but significant group of patients INS is severe and prolonged, lasting into adulthood (2, 11) and requiring much dedication by the family and resources by the National Health System to maintain stable remission and minimize the impact of this chronic disease on the patient's well-being. In these patients, multidrug immunosuppressive therapy leads to significant morbidity and to reduced compliance (12). There is therefore an unmet need for effective and less toxic therapies.

1.3 Mesenchymal stromal cells

Mesenchymal stromal cells (MSC) are multipotent progenitor cells resident in the bone marrow and other tissues (13). MSC can be obtained from bone marrow cells after in vitro culture and expansion. Since their discovery, MSC have been shown to be able to differentiate into several mesenchymal tissues, as well as home to inflamed sites contributing to tissue repair and mediate potent immunomodulatory effects both *in vitro* and *in vivo* (14), figure 1. The capacity to regulate immune responses leading to regeneration of damaged tissues provides a strong rationale for the use of MSC as a new treatment modality, especially in diseases characterized by the presence of an immune dysregulation leading to tissue damage. The early *in vitro* observations that MSC were able to inhibit T cell proliferation induced by allo-antigens, mitogens, CD3, and CD28 agonistic antibodies led to the

notion that MSC could be used therapeutically to modulate immune responses (13). A combination of in vitro co-culture studies and animal studies demonstrated that, in addition to inhibiting the proliferation and activation of effector T cells, MSC can also induce the expansion of regulatory T cells and inhibit the expansion and activation of B cells and NK cells (15). MSC can furthermore inhibit the differentiation and activation of myeloid-derived dendritic cells and induce the skewing of monocytes and macrophages toward an anti-inflammatory phenotype (16). Together, these data indicate that MSC can suppress the activation of the immune system by regulating cells of the adaptive and innate immune systems. Therefore, the therapeutic use of MSC has been attempted in a variety of clinical settings, and several phase 1 and phase 2 studies show their safety and suggest their effectiveness in a number of immune-mediated diseases, including glomerulopathies (15,17,18).

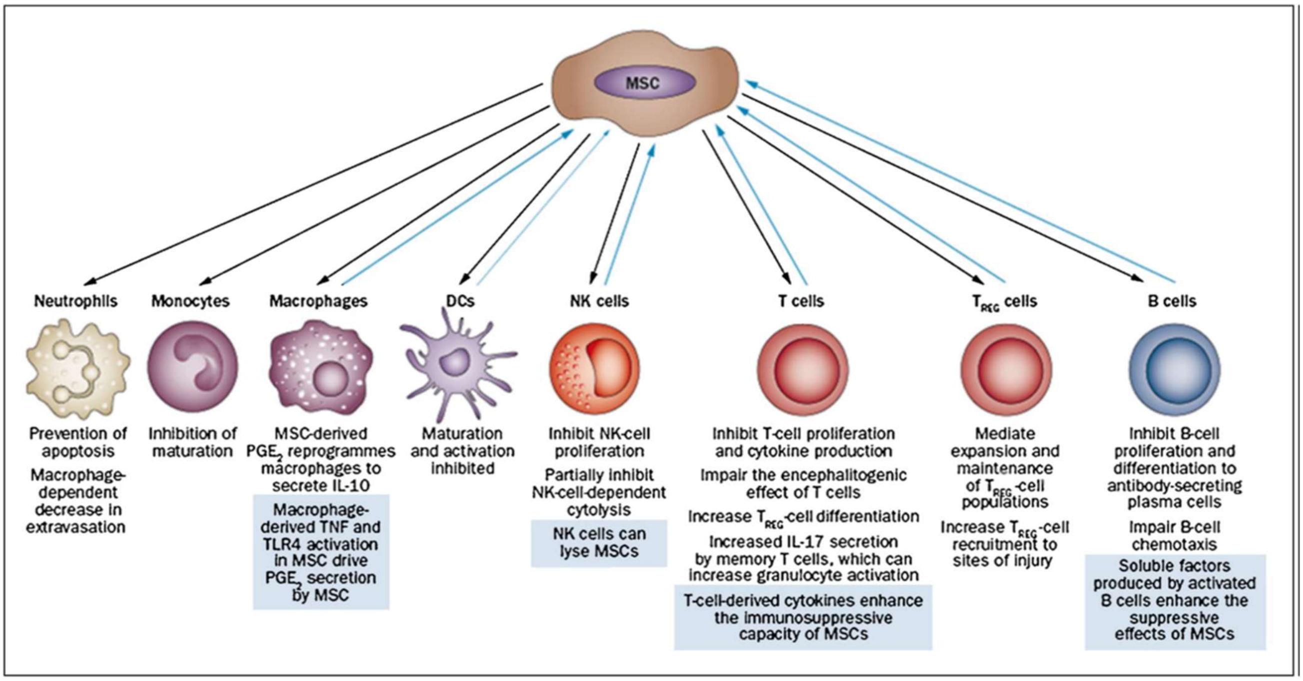

Figure 1 (19): MSC interaction with immune competent cells. Interactions of MSC and immune cells demonstrated in vitro. Each arrow represents functional interaction between the indicated cell types, as detailed in publications in peer-reviewed journals (reviewed elsewhere [19]). In many cases, bi-directional crosstalk exists, which influences the final outcome of the interaction, and thus the effects of MSC-based therapy on disease. Black arrows and text pertain to responses driven by MSC, whereas the effects of immune cells on MSC are indicated by blue arrows and boxes. In general, the effects of MSC on cells of the immune system are anti-inflammatory. DCs: dendritic cells; MSC: mesenchymal stem cells; NK: natural killer (cell); PGE2: prostaglandin E2; TLR4: toll-like receptor 4; TREG: regulatory T (cell).

In terms of safety, numerous studies assessing the use of autologous MSC in a consistent number of patients have shown no adverse effects after 1 year (20). This is the case both in the setting of acute graft-versus-host disease (GVHD), where 55 patients were treated (21) and in the setting of renal transplantation, where 106 patients were treated and compared to 53 controls (22). A more extensive listing can be found in sections 2.2 and 2.3 of this protocol and in the investigator brochure (IB).

1.4 Potential for the therapeutic use of mesenchymal stromal cells in idiopathic nephrotic syndrome: in vitro results

An important issue in the product development of MSC as a therapeutic tool is the source of MSC. The criteria that regulate the choice between autologous and allogeneic MSC remain poorly defined and the results published so far do not allow definitive conclusions. Allogeneic MSC from third party donors represent a ready-to-use, "off-the-shelf" product. This approach has the advantage of immediate availability. However, the risk of immune recognition and clearing of allogeneic MSC by the immune system of non-profoundly immunosuppressed subjects, such as those affected by INS, should be taken into account, especially with repeated infusions (23). For this reason we have chosen a so-called "patient designated" approach with autologous bone marrow-derived cells. In the context of a chronic disease like INS, which offers sufficient time for MSC ex vivo expansion, autologous cells can be employed to overcome the risk of immune rejection, provided that they are functionally active. Indeed, for some diseases it has been debated whether MSC from patients maintain the full spectrum of regenerative and immunomodulatory properties of MSC from third party healthy donors (reviewed in 14).

To confirm that MSC derived from INS patients maintain properties comparable to MSC derived from healthy subjects, we have performed a proof-of-principle pre-clinical study. Five children (3 males, 2 females, median age 13 years) diagnosed with INS at the Bambino Gesù Children's Hospital (OPBG) between September 2013 and December 2015 were included in the study. As controls, we used MSC isolated from residual cells of 8 healthy donors (HD) (5 males, 3 females, median age 15 years), who donated bone marrow for transplantation. Bone marrow-derived MSC were isolated and expanded *ex vivo* from both INS patients and HD, as previously described (24). Proliferative capacity, osteogenic and adipogenic differentiation and senescence assay were performed as described in Starc et al (25), where figures 1, 2 and 3 show that no difference was detected between MSC derived from INS patients and those derived from HD. More relevantly, immunomodulatory properties were assessed as follows:

1) T cell proliferation assay with phytohemagglutinin of HD peripheral blood mononuclear cells alone and with the addition of MSC from INS patients and from HD.

2) B cell proliferation assay with CpG of HD peripheral blood mononuclear cells alone and with the addition of MSC from INS patients and from HD.

3) Cytokine production (IL-2, IL-6, IL-10, TGFβ and IFNγ), in culture supernatants of peripheral blood mononuclear cells alone and with the addition of MSC from INS patients and from HD. As shown in figure 2, MSC from INS patients displayed a normal capacity to inhibit T and B cell proliferation and cytokine production compared to MSC derived from HD. Moreover, supernatant from MSC derived from INS patients was protective of adriamycin-induced podocyte damage in terms of reducing bovine serum albumin permeability.

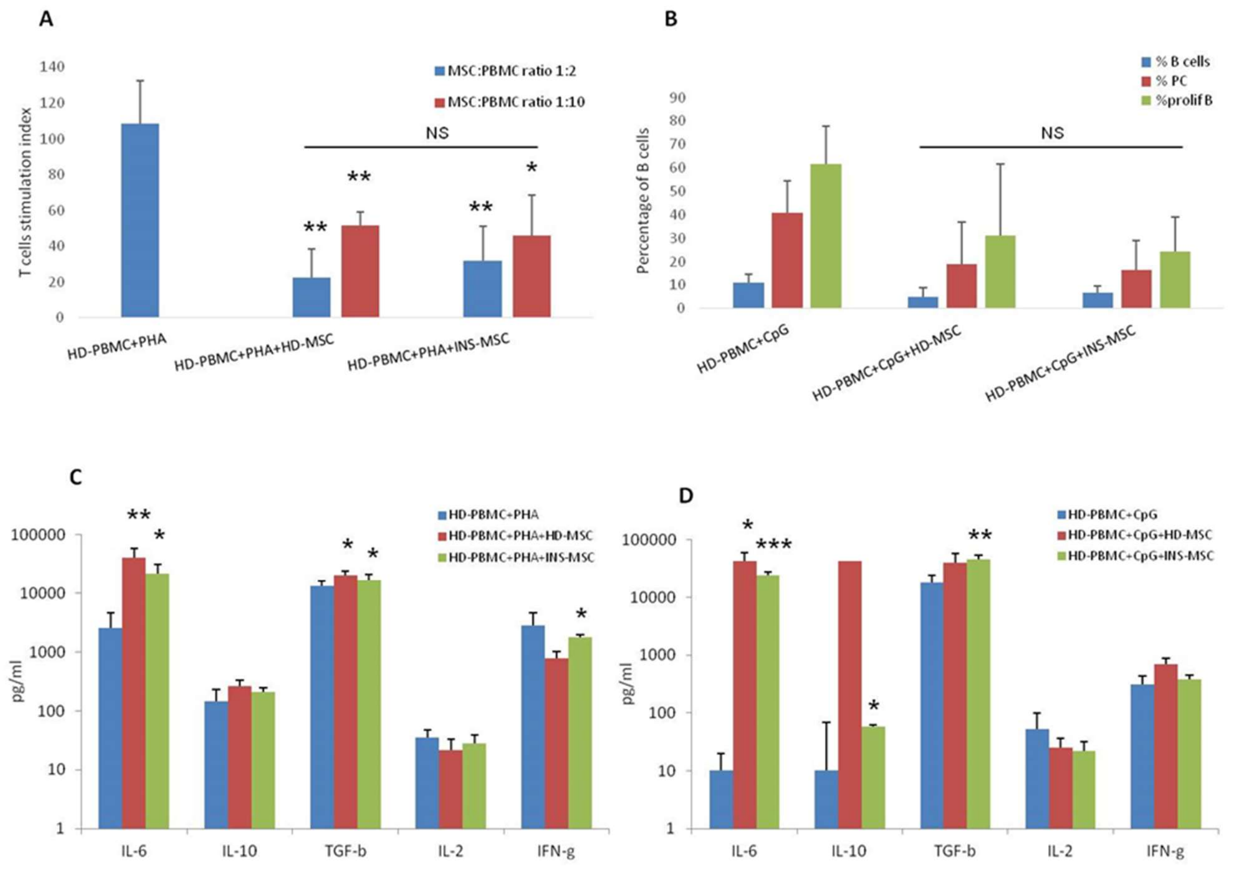

Figure 2. **(A) In vitro immunomodulatory effect of HD- and INS-MSC on healthy donor peripheral blood mononuclear cells (PBMCs) in an allogeneic setting.** The graph shows the percentage of residual proliferation of healthy donor PBMCs stimulated with phytohaemagglutinin (PHA) either in the absence or in the presence of HD- and INS-MSC. Each bar represents the percentage of residual proliferation of 10^5 PBMCs, in the presence of two different MSC:PBMC ratios (MSC:PBMC ratio of 1:2 and 1:10), calculated by measuring 3H-thymidine incorporation after 72 hours co-culture. Each bar represents the mean ± SEM of multiple experiments (each point being in triplicate) with MSC obtained from 5 INS-patients and 8 HDs. P values lower than 0.05 were considered to be statistically significant (compared with PBMC + PHA in absence of MSC: *, p<.05; **, p<.01). **(B) In vitro immunomodulatory effect of HD- and INS-MSC on B-cell proliferation and plasma cells differentiation in an allogeneic setting.** PBMCs were stimulated with CpG for 7 days and co-cultured with or without MSC. Cells were collected, stained for CMFDA, CD19, CD27, CD38 and IgM and analyzed by flow cytometry. The graph shows the percentage of HD-PBMCs stimulated with CpG either in the absence or in the presence of HD- and INS-MSC. Each bar represents the percentage of B cells, the residual proliferation and the percentage of plasma cells of $2x10^5$ PBMCs, in the presence of MSC (MSC:PBMC ratios 1:10). Each bar represents the mean ± SEM of multiple experiments (each point being in triplicate) with MSC obtained from 5 INS-patients and 8 HDs. **(C) In vitro immunomodulatory effect of HD- and INS-MSC on cytokine production by PHA-stimulated PBMC cultures.**An increase in anti-inflammatory cytokines (i.e. IL6, IL10 and TGFβ) was detected in the presence of both HD-MSC and INS-MSC, as compared with PHA-stimulated PBMC cultures and a decrease in pro-inflammatory cytokines (IL2 and IFNγ) was revealed in supernatants collected from the same co-cultures. Each bar represents the mean ± SEM of the results from experiments performed with the same HD- and INS-MSC samples employed in the PBMC proliferation assays. **(D) In vitro immunomodulatory effect of HD- and INS-MSC on cytokine production by CpG-stimulated PBMC cultures.** An increase in anti-inflammatory cytokines (i.e. IL6, IL10 and TGFβ) was detected in the presence of both HD-MSC and INS-MSC, as compared with CpG-stimulated PBMC cultures. A

decrease in pro-inflammatory cytokines IL2 in HD- and INS-MSC was revealed while an increase of IFNγ in HD-MSCs and, in a lower amount, also in INS-MSC was revealed in supernatants collected from the same co-cultures. Each bar represents the mean ± SEM of the results from experiments performed with the same HD- and INS-MSC samples employed in the PBMC proliferation assays. Results are expressed as pg/ml. P values lower than 0.05 were considered to be statistically significant (compared with HD-MSC: *, $p<.05$; **, $p<.01$; ***, $p<0.001$). HD, healthy donor; INS, Idiopathic nephrotic syndrome; NS, not significant; PBMC, peripheral blood mononuclear cell; PHA, phytohemagglutinin; PC, plasmacell; IL, interleukin; TGFβ, transforming growth factor β; IFNγ, interferon γ.

Following this encouraging pre-clinical in vitro data, detailed in the Investigator Brochure, we propose to employ autologous bone marrow-derived MSC *in vivo* in the treatment of children with INS.

1.5 Conclusions

In summary:
1) the etiology of INS remains unclear;
2) response to immunosuppression suggests an immune dysregulation leading to glomerular basement membrane disruption and proteinuria;
3) currently, no disease-modifying drugs have been identified;
4) in INS, disease morbidity is largely attributable to the frequency of relapses, and to the side-effects of drugs (mainly PDN) that are used to treat and prevent these relapses;
5) therefore, new therapeutic approaches with potential and a promising safety profile are much needed
6) MSC from children with severe forms of INS display regenerative and immunomodulatory properties, comparable to MSC derived from healthy controls.

Therefore, the therapeutic use of autologous MSC in severe forms of INS may be a safe and effective therapeutic steroid-sparing approach. To the best of our knowledge, no registered trials for the use of autologous mesenchymal stromal cells in INS are being conducted.

We propose to evaluate the safety and feasibility of the therapeutic use of autologous MSC harvested and purified from the bone marrow (BM) of children and young adults with severe, recurring forms of immune-mediated nephrotic syndrome, expanded *ex vivo* and reinfused to the same patient. Given their immunomodulatory properties, our working hypothesis is that their therapeutic use will permit relapse-free reduction of oral immunosuppression in INS patients.

Twenty patients with severe frequently relapsing or steroid-dependent forms of nephrotic syndrome will be enrolled in 2 centers, the IRCCS Ospedale Pediatrico Bambino Gesù in Rome and the Azienda Socio Sanitaria Territoriale - Ospedale Papa Giovanni XXIII (ASST-PG23), the latter with the support of the Clinical research Center for Rare Diseases *"Aldo e Cele Daccò"* of the IRCCS Istituto di Ricerche Farmacologiche Mario Negri (IRFMN) in Bergamo. At baseline a bone marrow aspirate will be performed, and mesenchymal stromal cells will be isolated and expanded in the Good Manufacturing Practices-approved Cell Factory facilities of both centers for therapeutic use. Identical protocols and compatible specifications, taking into account the weight variability of subjects due to their age span, will be employed. Each patient will be treated with 2 doses of autologous bone marrow-derived mesenchymal stromal cells at a 7-day interval and subsequently followed for 12 months. After treatment, oral immunosuppression will be gradually tapered in 6-9 months and the number of relapses of proteinuria will be monitored with daily Albustix. Clinical and laboratory parameters will be assessed monthly, while more detailed assessments, including full blood exams and evaluation of B and T cell

subsets and function will be performed at baseline, at time of infusion, and after 1,3,6,9 and 12 months. All adverse events will be carefully recorded. The study will be performed according to Good Clinical Practices with independent monitoring, pharmacovigilance and overview by a Safety Committee.
This phase 1 study is expected to provide results on the safety and feasibility of the therapeutic use of bone marrow-derived autologous MSC in children and young adults with severe, difficult-to-treat INS.

2. INVESTIGATIONAL MEDICINAL PRODUCT BENEFIT AND RISK ASSESSMENT

2.1 Investigational medicinal product

The investigational medicinal product consists of autologous mesenchymal stromal cells, referred to as MSC. This is a somatic cell therapy product derived from the isolation and expansion of human bone marrow-derived cells. The tissue is procured in accordance with EMA Tissues & Cells Directive, 2004/23/EC, and the product is manufactured in accordance with Good Manufacturing Practices (GMP), as described in section 6 of this protocol and detailed in the Investigator Medicinal Product Description (IMPD).

2.2 Risks

Given that MSC are a cellular therapeutic agent, representing a physiological component of the recipient's body, they should not show any of the toxicities deriving from standard synthetic therapeutics. Specifically, no teratogenicity or reduction of fertility can be expected. Indeed, MSC have been employed therapeutically since the 1990s and the extensive, albeit very heterogenous literature available, describing mostly very small number of patients per study, collectively reaches more than 3000 patients reported. A number of recent meta-analysis studies (26-29) comparing and pooling results from the best available trials for specific indications (i.e. liver cirrhosis, pulmonary fibrosis, myocardial infarction, diabetes mellitus), in addition to a rigorous comprehensive meta-analysis including all indications and available randomized controlled trials (30) are published. All failed to detect associations between intravenous infusion of MSC and severe adverse effects, such as acute infusional toxicity, organ system complications, infections, death or malignancy. Transient fever upon infusion was reported (30). The "G. Lanzani" Cell Factory of the ASST-PG23 in Bergamo has treated over 100 patients with MSC expanded under conditions identical to those that will be employed in this study and no major adverse events have been observed (31).
Theoretically, the possibility exists that MSC expanded by long-term culture *in vitro* may give rise to tumors after infusion *in vivo*. However, whereas malignant transformation has been demonstrated with murine MSC, no such event has been reported using human MSC (20). Moreover, no transformation in patients treated with MSC has been reported so far, and an autopsy study performed on patients receiving MSC for malignancies showed no ectopic tissue formation or malignant tumors of MSC origin by macroscopic or histological examination (32). In the *in vitro* study performed in our laboratories on MSC expanded from the BM of 5 nephrotic syndrome patients, MSC were cultured until reaching senescence phase. An average of 9 passages were performed for patient-derived MSC compared to 10 for healthy control-derived MSC. No differences were detected between MSC of INS patients and MSC of healthy donors, and no chromosomal alterations were observed. Bearing in mind that our therapeutic expansion protocol plans to execute only one passage in vitro and to freeze cells ready for therapeutic infusion at the second cell detachment step, the risk of transformation appears negligible. In order to achieve total safety, expanded cells pre-infusion will be assessed for clonogenic potential and chromosomal alterations, as detailed in the IMPD.

2.3 Potential benefits

MSC have been shown to have multiple properties: they can differentiate along several lineages to stromal cells (bone, cartilage, fat) and to epithelial cells, contributing to tissue repair and to injury healing (13, 33). Moreover, they have been found to have anti-inflammatory and immunomodulatory properties against a variety of cells and through several mechanisms. Specifically, since the discovery of their ability to inhibit the mixed lymphocyte reaction in an HLA unrestricted manner (34), a number of studies have demonstrated the ability of MSC to modulate the function of immune competent cells. Specifically, MSC are able to inhibit T effector cell proliferation and cytokine production, promote T regulatory cell expansion, inhibit B cell proliferation and differentiation into antibody-producing plasma cells, inhibit NK cell expansion (reviewed in 14, 15, 19), upregulate release of inhibitory prostaglandins and cytokines such as prostaglandin E2 (PGE2) and IL-10 and modulate the function of innate immune and antigen-presenting cells, such as monocyte/macrophages and dendritic cells (16).

To confirm that the proposed source of cellular product for our study, i.e. autologous MSC expanded from the bone marrow of children with severe forms of INS, maintain the properties of MSC from third-party healthy donors, we performed an *in vitro* proof-of-principle study, whose results are detailed in section 1.4 of this protocol and in the IMPD. Briefly, we verified that MSC expanded from the BM of 5 children with INS who present a very similar clinical picture to those that we propose to enroll in this study, in terms of disease severity and of burden of immunosuppression, maintain differentiative and immunomodulatory properties comparable to those of MSC derived from healthy controls. Moreover, supernatants from these MSC were able to ameliorate podocyte damage induced by adriamycin, in a classic *in vitro* model of glomerular filtration barrier injury. These results provide encouraging preliminary data for the *in vivo* use of autologous MSC expanded from patients with difficult INS in a clinical setting (25), and confirm findings in the context of renal transplantation showing that therapy with prednisone, cyclosporin A, mycophenolate do not hinder MSC immunomodulatory properties in vitro (35).

In vivo, as reviewed in Fibbe et al (36), MSC are short-lived and may be lysed soon after being injected into the circulation. Macrophages that phagocytose lysed MSC become polarized to a immunosuppressive phenotype via factors that include IL-6 and PGE2. These macrophages in turn secrete factors that contribute to the immunosuppressive state (including IL-10 and IL-6). They also produce CC chemokine ligand 18 (CCL18) and soluble HLAG (sHLAG), which in conjunction with MSC-derived factors, including transforming growth factor β (TGFβ) and PGE2, favour the induction of regulatory T cells. Activation by the proinflammatory microenvironment in the host via licensing factors such as interferon γ (IFNγ), tumor necrosis factor (TNF) and IL-1 or via direct interaction with activated immune cells induces MSC to express and secrete immunomodulatory molecules, such as indoleamine 2,3-dioxygenase (IDO) and haemoxygenase (HO). These molecules have a role in suppressing the proliferation of target cells, including T cells, B cells and natural killer (NK) cells.

The concept of using a cell product that, though short-lived, exerts specific immunomodulatory actions and has few apparent off-target effects as an alternative to non-specific systemic immunosuppression in the settings of autoimmune disease and transplantation is very appealing (36).

This can clearly be beneficial if exploited therapeutically in a variety of clinical conditions. Encouraging results in this sense have been obtained in a large number of immune-mediated diseases, such as graft-versus-host disease (GVHD) (21), multiple sclerosis (37) and inflammatory bowel disease (38). Considering specifically renal conditions, MSC have been therapeutically employed (17, 18) and are being currently studied in ongoing clinical trials

(https://www.clinicaltrials.gov/ct2/results/details?term=mesenchymal&cond=Renal+Disease) for the treatment of patients with acute kidney injury, renovascular disease (39), diabetic nephropathy, polycystic kidney disease (40), senescence in chronic kidney disease and, more relevantly to the current therapeutic proposal, lupus nephritis (reviewed in 19), graft survival in renal transplantation (reviewed in 41), focal segmental glomerulosclerosis (42) and in one atypical case of nephrotic syndrome (43).

Results of the use of MSC in animal models of nephrotic syndrome

Animal models of nephrotic syndrome are far from perfect. The most reliable and widely employed model is the Adriamycin-induced glomerulosclerosis in Wistar rats, in which a toxic insult (Adriamycin) determines podocyte and glomerular filtration barrier damage leading to proteinuria and progressive loss of renal function. Of note, this is clearly not an immune-mediated process. In this context, experiments have shown, following the *in vivo* infusion of allogeneic bone marrow-derived MSC, a clear-cut reduction of podocyte damage and of glomerulosclerosis with no adverse effects. However, proteinuria was not reduced by this approach (44).

Results of the therapeutic use of MSC in lupus nephritis

The most relevant study to date is the recently published randomized, double-blind, placebo-controlled trial of allogeneic umbilical cord-derived MSC performed on 18 patients with class III or class IV lupus nephritis, all followed in one centre in China. Participants were randomly assigned at a ratio of 2 to 1 to receive either two weekly intravenous injections of human umbilical cord-derived MSC or placebo. In addition, both groups received immunosuppression (intravenous methylprednisolone and cyclophosphamide, followed by maintenance therapy with oral prednisolone and mycophenolate mofetil). A similar proportion of patients in each group achieved complete remission. Therefore, the study failed to prove a beneficial effect of the therapeutic use of MSC in this context, showing however overall excellent outcome for all patients and no adverse effects (45). Before this, a total of 4 studies evaluating a total of 152 patients, using allogeneic MSC derived from BM or from umbilical cord, had been performed. As reviewed by Tyndall (19), these studies showed promising results in terms of improvement of scores of activity, reduction of proteinuria and of serological markers. No major adverse effects that could be ascribed to the MSC infusion were reported.

Results of the therapeutic use of MSC in focal segmental glomerulosclerosis

A single pediatric case describing the use of allogeneic bone marrow-derived MSC in the setting of focal segmental glomerulosclerosis recurring on a renal graft immediately following transplantation is reported (42).The patient was treated with prednisone, calcineurin inhibitors, mycophenolate mofetil and rituximab, but obtained stabilization of his proteinuria only with intensive plasmapheresis, as frequently observed in these cases. Three infusions of MSC were administered and plasmapheresis was tapered and stopped. The MSC infusions were well tolerated with no adverse reactions, and a sustained stabilization of proteinuria and of renal function despite interruption of plasmapheresis was obtained, improving the patient's quality of life. Prolonged reduction of a panel of inflammatory markers was observed, with no adverse events reported at 22 months of follow-up.

Results of the use of MSC in nephrotic syndrome post-allogeneic hematopoietic stem cell transplant

A single case describing the use of allogeneic bone marrow-derived MSC for the treatment of nephrotic syndrome in a 31-year old woman, 10 months following an allogeneic hematopoietic stem cell transplant from a sibling for an hematological malignancy is reported. The nephrotic syndrome proved

unresponsive to treatment with prednisone, cyclosporin A and rituximab, therefore the patient underwent treatment with MSC that proved safe and well tolerated. Two weeks after the first MSC infusion, therefore 6 weeks after the treatment with rituximab, proteinuria decreased substantially. Renal function remained normal, and increased T regulatory and B regulatory populations were observed (43).

<u>Results on the use of MSC in renal transplantation</u>

In the setting of solid organ, in particular renal transplantation, the therapeutic use of MSC has been assessed with the objective of modulating T cell memory and promoting the development of T regulatory cells, thus achieving a beneficial balance between graft rejection avoidance and a less toxic immunosuppressive regimen (46). To this purpose, a step-by-step approach has been employed to determine optimal timing of infusions and concomitant immunosuppressive treatment. Autologous MSC have been largely preferred in this setting, to minimize sensitization and maximize efficacy in the context of partially immunocompromised patients.

The results obtained in these studies are of particular relevance to our proposed study for two reasons:

1) Because the treatment regimen employed to prevent graft rejection entails the use of prolonged immunosuppression with prednisone, mycophenolate mofetil and a calcineurin inhibitor which is very similar to that employed to maintain remission in severe idiopathic nephrotic syndrome patients. Therefore the ultimate objective of these studies, to achieve a reduction of a toxic immunosuppressive regimen, is common to our objective in treating INS patients
2) Because these studies have been performed using autologous MSC at very similar timing and doses as those that we propose, and more precisely 2 of these studies report patients treated with MSC prepared in one of the 2 Cell Factories that this study will employ, with a preparation protocol identical to the one that will be employed in this study.

In total 157 patients have been treated to date. Severe infusion reactions or side effects are not described, and in some cases reduced immunosuppression was achieved without graft rejection (reviewed in 41). It is important to note that in one of the first published studies (35) the MSC infusion 7 days after kidney transplantation was accompanied by a temporary early graft dysfunction in two patients. This led to a modification of the timing of the infusions (before transplantation), with no repetition of this observation (47).

These findings support a role of the use of autologous MSC for immunosuppressive drug minimization, calcineurin inhibitor withdrawal, prevention of rejection and reduction of chronic transplant inflammation and fibrosis. The evaluation of the immunomodulatory properties of this approach *in vivo* has confirmed that an expansion of peripheral T regulatory cells and a functional modulation of memory T cells occurs (41, 46). Taken altogether, these results suggest that autologous MSC might be beneficial also in difficult forms of immune-mediated nephrotic syndrome.

2.4 Comparison to standard therapeutic approach

Among children with steroid-sensitive INS, less than half will have a very benign course, with no or infrequent relapses. The remaining 50-60% of children will present a prolonged disease course, with multiple relapses spanning over decades. If relapses occur more often than twice in the 6 months following onset or more than 4 times per year in subsequent years, patients are defined as having frequent-relapsing INS, whereas if relapses occur during steroid therapy or within 14 days of steroid discontinuation, they are defined as steroid-dependent. These forms require second-line steroid-

sparing immunosuppression, mainly calcineurin inhibitors, mycophenolate mofetil and more recently rituximab (1). These therapeutic approaches have considerably improved the prognosis of INS, minimizing the well-known devastating consequences of prolonged high-dose prednisone on statural growth, body weight and glucose tolerance, hypertension (11, 12, 48). However, all the second-line agents carry their own burden of side effects: calcineurin inhibitors can lead to renal toxicity and hypertension, plus hirsutism and gingival hypertrophy for cyclosporin A and glucose intolerance for tacrolimus. Mycophenolate gives myelotoxicity, teratogenicity and in some patients abdominal pain and diarrhea. Rituximab comes at a considerable cost for the Italian National Health System, is administered i.v. and may determine in children with INS prolonged depletion of memory B cells and hypogammaglobulinemia (Additional File 1), as has already been observed in adults with other diseases (8-10). In a part of these patients a therapeutic approach permitting prolonged steroid-free and relapse-free survival on one immunosuppressive drug (cyclosporin A, mycophenolate mofetil or rituximab) is achieved, and this is considered acceptable in terms of safety and quality of life.
However, in a significant percentage (about 15-20%) of these children remission is maintained only with the concomitant use of 2 or 3 oral immunosuppressive drugs, often with only temporary benefit of rituximab infusions. Past puberty the disease is known to resolve in a substantial proportion of patients, but in 20-30% of frequently-relapsing or steroid-dependent cases disease persists beyond the post-pubertal age into adulthood. For these children and young adults, whose disease history lasts decades, the burden of combined immunosuppression and oral prednisone leads to a very significant morbidity (11, 12, 48).

2.5 Summary of benefit/risk assessment

In conclusion, the above-mentioned considerations suggest:

1) that the therapeutic use of MSC for the treatment of a variety of different conditions appears overall to be very safe in the studies that have been published in the literature
2) that MSC appear both *in vitro* and *in vivo* to present immunomodulatory potential that could theoretically be beneficial in the treatment of idiopathic nephrotic syndrome
3) more specifically, that autologous MSC expanded from patients with INS maintain *in vitro* their proliferative and immunomodulatory potential, and
4) that the use of autologous MSC prepared in exactly the same modality as the ones that will be used for this study appear to be safe and effective in patients with other conditions, including renal transplantation. This last point is important considering that several factors can contribute to the immunomodulatory potential of MSC employed for therapeutic use, and that one of the weaknesses of a number of studies evaluating them is the lack of precise information regarding cellular therapy preparation.
5) Idiopathic nephrotic syndrome is a clinically heterogeneous disease. However, patients with severe, prolonged and difficult forms of INS present an unmet clinical need for safer and less toxic therapeutic approaches.

In this context, the use of autologous mesenchymal stromal cells may represent a safe therapeutic option allowing significant reduction of disease burden by maintaining patients in remission with less concomitant immunosuppression.
The study will be covered by an insurance policy according to the laws in force, in the event of a patient suffering any significant deterioration in health or well-being, which is proven as being a direct result of participation to the study.

3 STUDY OBJECTIVES

3.1 Primary objectives

To assess the feasibility and safety of harvesting from their bone marrow, isolating and expanding ex vivo in an approved facility and then infusing autologous MSC in children and young adults with severe multi-relapsing or steroid-dependent INS.

3.2 Secondary objectives

1. To evaluate whether the therapeutic use of autologous MSC is able to prevent disease relapse despite tapering of prednisone and/or other immunosuppressive treatments in children and young adults with severe multi-relapsing or steroid-dependent INS.
2. To assess whether the therapeutic use of autologous MSC may reduce the need for prednisone and/or other immunosuppressive agents to prevent and treat further disease relapses;
3. To evaluate whether tapering or withdrawal of immunosuppressant therapy is associated with regression of the related toxicities, such as growth retardation, hypertension, impaired glucose tolerance and obesity, infections, B cell suppression;
4. To evaluate the immunomodulatory effect of the MSC infusion in vivo in a clinical setting.

3.3 Primary end-point

Primary outcome: safety parameters
- Safety outcomes will include serious and non-serious adverse events, including acute reactions during MSC infusion, infectious episodes and malignancies.

3.4 Secondary end-points

Secondary outcomes:
- Recurrence of INS, defined as 3+ or more positive Albustix dipsticks for proteinuria on 3 consecutive days or by positive dipsticks (1+ to 3+) for 7 consecutive days, in the 12 months before and in the 12 months following MSC infusions.
- The dose of immunosuppressive therapy required to prevent further INS relapses
- Adverse effects of immunosuppressive therapy, such as arterial hypertension and need for antihypertensive therapy, obesity and impaired glucose tolerance, dyslipidemia, renal dysfunction, stunted statural growth, infections, immunocompetence;
- Kidney function at baseline and at one year after MSC administration.

4 INVESTIGATIONAL PLAN

4.1 Overview of trial

This will be a prospective phase 1 open-label non-randomized study.
At the beginning of the screening period, potential patients and their families will receive information regarding the study, will have time to formulate questions, and if in agreement will consent to participating in the study. Following written informed consent/assent obtained from parents/legal representatives, as well as from underage adolescents and children as appropriate, the screening period will begin, leading if successful to enrollment in the trial.
During the enrolment visit, inclusion and exclusion criteria will be checked to confirm eligibility and baseline data will be collected (see below and Table 1). At baseline, laboratory parameters (see Table 1) will be assessed and a second visit will be arranged, to take place within 7-14 days, during which bone marrow aspirate will be performed (50ml of blood will be aspirated in an heparinated syringe). Patients will be sedated to avoid discomfort. In this occasion, if not previously performed in the last 2 years, at the same time patients will also undergo renal biopsy.
The patients will then enter a 6-12 week cell expansion period during which all therapy will be maintained and every other day urinary dipstick with Albustix will be recorded to verify remission of proteinuria. During this period, the bone marrow will be used in a GMP-approved Cell Factory facility either in Rome or in Bergamo to purify and expand an adequate amount of MSC for therapeutic use. The protocol of MSC preparation in the 2 facilities will be identical, and all procedures will be standardized to ensure reproducible results within the patient cohort. After the expansion period, each patient will receive 2 intravenous infusions of autologous MSC 7 days apart. One month after the first MSC infusion, steroid and/or concomitant immunosuppressive therapy will be tapered and progressively withdrawn over 6 to 9 months. On follow-up, urine will be tested every other day at home by Albustix. Routine laboratory exams, 24-h proteinuria (or protein/creatinine ratio) and "immunomonitoring" will be performed at baseline, after 1,3,6,9 and 12 months from the first MSC infusion (Table 1). "Immunomonitoring" will comprise laboratory assays of B and T cell phenotype, proliferation and cytokine production (detailed in Additional File 2) to evaluate the immunomodulatory effect of the MSC infusion and the patient's immunocompetence as the oral immunosuppression is tapered in vivo in a clinical setting.

4.2 Study Timeline:

Screening period: 7-14 days following informed consent, comprising enrollment, baseline evaluation (Visit 1), arrangements for visit 2
Expansion period: 6-12 weeks starting from Visit 2 (bone marrow aspirate, ± renal biopsy)
Therapeutic phase: 8 days, including reassessment to verify clinical status and disease remission at time 0, followed by 2 MSC infusions i.v. at day 1 and day 8 (detailed below).
Follow-up observational phase: 12 months
Enrollment period: 18 months
Time for data collection and scientific publication of completed study results: 6 months
Total duration of the study: 40 months
End of study: it is defined as the last visit of the last patient + 6 months for data collection, data analysis and preparation of the scientific publication.

The study will last a total of 15 months (3 months of screening and expansion plus 12 months post-treatment) for each patient. At 12 months after the first MSC infusion (end of follow-up period), all baseline evaluations will be repeated. No change in diet and concomitant INS medications, in particular with angiotensin-converting enzyme inhibitors or angiotensin II receptor blockers, will be introduced throughout the study period.
Following the therapeutic phase, during the 12 months follow-up tapering of immunosuppression will be attempted, starting 1 month after the first MSC infusion. Immunosuppressive drugs will be withdrawn in the following order: first steroids, then calcineurin inhibitors (cyclosporin A or tacrolimus) and finally antiproliferative agents (azathioprine, mycophenolate mofetil or cyclophosphamide). Immunosuppressive tapering/withdrawal will start at 1 month after the first MSC infusion by reducing the doses for prednisone by 2,5-5 mg every 1-2 weeks and for subsequent second-line agents (in the order listed above) by a quarter of the original dose every 2-3weeks, in order to achieve complete withdrawal within 6 to 9 months. These guidelines are aimed at standardizing immunosuppressive therapy tapering/withdrawal, to limit the potential confounding effect of different approaches on data interpretation, but the Investigator will have the possibility to delay progressive dose reductions according to patient-specific conditions. Any deviation from the above guidelines, however, will be reported and justified on the patient's record forms (eCRF).
INS recurrence will be defined as 3+ or more positive Albustix dipsticks for proteinuria on 3 consecutive days or by positive dipsticks (1+ to 3+) for 7 consecutive days. Patients with recurrent INS will be treated with high-dose steroids as per Center practice. If the reintroduction of a second-line steroid-sparing immunosuppressive agent is deemed necessary, the best approach will be chosen as per Center practice.

4.3 Patient selection

Patients will be identified among the subjects who are referred to the Pediatric Nephrology (Ospedale Pediatrico Bambino Gesù - Rome) and Nephrology (Azienda Socio Sanitaria Territoriale Papa Giovanni XXIII - Bergamo) centers involved in the trial. Both centers are third-level referral hospitals for patients of high complexity, and care for large cohorts of patients with INS, with hundreds of patients with steroid-sensitive nephrotic syndrome followed on a regular basis. The total number of patients with INS followed by these 2 centers should guarantee the success of the enrolment phase, which in our estimate should last 18 months for 10 patients for each center, for a total of 20 patients. Enrollment will entail selection of candidates possessing the required criteria, detailed illustration of the study by the clinicians involved in it and acquisition of informed consent as per GCP.

INCLUSION CRITERIA:

- Males and females aged 5 to 40 years.
- Difficult to treat FRNS or SDNS patients, defined as steroid-dependent or multirelapsing INS patients with 2 or more relapses in the previous year in spite of prednisone and/or one or more other immunosuppressive steroid-sparing agent. Only patients reported to invariably relapse upon treatment tapering or withdrawal who are on stable (from at least 1 month) complete (<0.3 g/24h for adults or <4 mg/h/m^2 for children) or partial (<3.5 g/24h for adults or <40 mg/h/m^2 for children) remission of the INS will be included. Rituximab treatment can have been previously performed but reconstitution of B cells, defined as total CD19/CD20 lymphocyte count above 5% of total lymphocytes by cytofluorimetry, must have occurred and must be recorded.

- Histological diagnosis of MCD, FSGS, mesangial proliferative glomerulonephritis.
- Written informed consent (or consent from parents or tutors for underage patients, as appropriate).
- If applicable, female participants must have pregnancy test by beta-HCG dosing and be negative.
- Patients of child-bearing or child-fathering potential must be willing to practice and must contact their physician With their physician, they must agree on the most appropriate approach for birth control from the time of enrollment in this study and for 3 months after receiving the latest MSC infusion, such as abstinence from sexual intercourse or such as other methods, of which the scientific limitations will be explained, such as, specifically:

  - progestogen-only oral hormonal contraception, where inhibition of ovulation is not the primary mode of action;
  - male or female condom with or without spermicide;
  - cap, diaphragm or sponge with spermicide.

EXCLUSION CRITERIA:

- Advanced renal failure (creatinine clearance less than 20 ml/min/1.73m^2), calculated using the Schwartz formula or the Cockroft-Gault formula, as appropriate;
- Refractory or persistent NS;
- Genetic mutations associated with intrinsic abnormalities of the glomerular barrier;
- Pregnancy or lactating;
- Women of childbearing potential without following a scientifically accepted form of contraception;
- Infectious pathogen testing positive for active infection;
- Legal incapacity;
- Evidence of an uncooperative attitude;
- Previous diagnosis of: intellectual disability/mental retardation, dementia, schizophrenia.
- Any evidence that patient will not be able to complete the trial follow-up.

The Steering Committee will be chaired by the principal investigator and will include Prof. Emma, Prof. Locatelli from OPBG and Prof. Remuzzi, Prof. Introna from ASST-PG23 and the three members of the Safety Committee. This latter committee will be composed of three physicians (Dr. Ciro Esposito – Fondazione “S. Maugeri”, Pavia, Dott.ssa Nadia Rubis - IRFMN, Bergamo, Dott.ssa Marta Ciofi degli Atti– OPBG, Roma) who are not directly involved in the study and have agreed to monitor adverse events and to review the study safety data every 6 months.

4.4 Interim analysis

An interim analysis will be performed after 10 enrolled patients have been treated, assessing the safety of the therapeutic approach in terms both of number and severity of adverse events and in terms of number of disease relapses. Results will be evaluated by the Steering Committee, which in conjunction with the Ethical Committee and with the Safety Committee can rule the discontinuation of the study. The Safety Committee will review results every 6 months and in case of unexpected events throughout the course of the study.

4.5 Premature study discontinuation

The sponsor may decide to terminate the study prematurely based on the following stopping rules:

1) Safety issue, i.e. there is evidence of unacceptable risk for study patients, more specifically:

Early adverse events: the study will be discontinued if more than 2 out of 6 patients enrolled in the study experience an early adverse event such as anaphylaxis.

Late adverse events: the study will be discontinued if more than 2 out of 6 patients enrolled in the study experience a late adverse event such as neoplasia or severe infection.

Efficacy: the study will be discontinued if more than 2 out of 6 patients experience an increase in the number of relapses in the 12 months following treatment with MSC compared with the previous 12 months, suggesting that treatment increases the likelihood of a disease relapse.

2) There is reason to conclude that it will not be possible to collect the data necessary to reach the study objectives and it is therefore not ethical to continue enrolment of more patients; for example insufficient enrolment that cannot be improved.
3) The Safety Committee or the Steering Committee recommend to end the trial based on viable arguments other than those described above.

The sponsor will promptly notify all concerned investigators, the Ethics Committees and the regulatory authorities of the decision to terminate the study. The sponsor will provide information regarding the time lines of study termination and instructions regarding treatment and data collection of enrolled patients.

5 PATIENT WITHDRAWAL

Patients must be withdrawn for the following reasons:
- at their own (or their parent's/legal guardian's if underage) request without giving reasons;
- at the discretion of the investigator;
- if adverse events (including intercurrent illnesses) develop, which rule out continuation of the study.

If administration of any drug is necessary, which is not permitted according to the study design, this should be discussed between the investigator and the clinical project manager.

In all cases, the reason for withdrawal must be recorded in the subject's medical record and on the eCRF. All withdrawals motivated by the development of an adverse event must be reported in accordance with the procedures detailed in the Adverse Event Reporting Section. Investigators will make every effort to contact subjects lost during follow-up. Subjects who have experienced an AE during the study will be followed until the event resolves, or until the Safety Committee decides that further follow-up is not necessary.

6 TREATMENT

6.1 Description of medicinal product

The investigational medicinal product consists of autologous mesenchymal stromal cells, referred to as MSC. This is a somatic cell therapy product derived from the isolation and expansion of human bone marrow (BM)-derived cells. The tissue is procured in accordance with EMA Tissues & Cells Directive, 2004/23/EC, and the product is manufactured in accordance with Good Manufacturing Practices (GMP), as detailed below and in the Investigator Medicinal Product Description (IMPD).

6.2 Preparation of cellular product

The manufacturing process for the cell product has been developed by the "G. Lanzani" Cell Factory ASST-PG23 in Bergamo and is based on a xeno-free expansion protocol. The BM aspirate of the patient will be collected according to local protocols and transported to the manufacturing facility using a designated transport box. Transport will occur at room temperature (15°-25°C). The BM aspirate will be processed within a timeframe of maximum 4-6 hours from harvesting. In the GMP facility, BM will be directly seeded in cell-stack flasks up to first harvesting (P1) and subsequently cells will be expanded in multiple (up to six) 5-layer flasks until final harvesting (P2).The excess P1 cells will be aliquoted, frozen and banked in vials containing 4-10 x10^6 cells/vial to be used in case of possible further clinical need. The seeded cells are incubated for a maximum of 15 days in a humidified 37°C incubator with 95% (v/v) air/5%(v/v) CO2.

For continuous expansion the MSC culture needs to be regularly supplied with fresh medium. MSC culture medium consists of alpha-MEM with 5% Platelet Lysate (PL), 50 mcg/ml Gentamicin. Medium is refreshed every 3-4 days by removing the medium from the culture flask and replacing the 40% with fresh MSC culture medium. Culture confluence is estimated using an inverted microscope. When the cell cultures reach a confluence of 70%, the cells in culture are collected. In this stage cells are detached from flasks with TrypLESelect which, compared to porcine-derived trypsin, is gentler on cells, does not require neutralization and does not contain xenogeneic proteins. Final product is re-suspended in human AB plasma +10% clinical-grade DMSO (final) and frozen in a heat-sealed ethyl vinyl acetate (EVA) double freezing bags by means of a controlled-rate freezer. The cell-containing inner bag is sealed by heat using a tube sealer and sealing is verified mechanically. Then the inner bag is inserted in an outer bag. Once frozen, the bags are stored in the vapour phase of liquid nitrogen (N2) tank until the time of infusion.

The preparation and labeling of the MSC product will be performed as detailed in the IMPD according to GMP guidelines. All products will be maintained in a controlled environment and stored under appropriate conditions. Both study sites have standard operating procedures in place for storage, release, thawing, preparing and administration of the investigational medicinal MSC.

The "G. Lanzani" Cell Factory at the ASST-PG23 in Bergamo has treated over 100 patients with MSC expanded under these conditions. The other MSC production partner is the OPBG Cell Factory. It has already implemented identical Standard Operation Procedures for bone marrow harvesting and MSC isolation, expansion, harvesting, cryopreservation and thawing. Through tight coordination, the two cell factories will develop a fully harmonized production procedure to ensure product consistency across both sites. Both MSC production facilities will ensure standardization by implementing a stringent monitoring plan.

For product release, analysis methods that have been validated according to European Pharmacopeia guidelines will be employed, complementing them with centralized characterization and release testing. All MSC batches will be tested for microbial and mycoplasma contamination, cell morphology, viability, surface marker expression (including CD73, CD90, CD105) and for contamination with residual hematopoietic cells (CD14, CD34, CD45). On 103 consecutive MSC productions at the center in Bergamo, 100% cases have shown full compliance with the above release criteria, whereas in only 4 cases sporadic chromosomal aberrations were observed as already reported (49).

6.3 Dosing regimen

Two administrations of the investigational medicinal product MSC will take place with an interval of 7 days between them (day 1 and day 8). A dose of MSC consisting of 1 x 10^6 MSC/kg pre-thawing, in a range of 0.75 x 10^6 MSC/kg to 1.25 x 10^6 MSC/kg (maximum dose 100 x 10^6 MSC) will be used.
Intravenous MSC therapy is usually dosed at 1-2 x 10^6/kg for adults and children alike. This dose has a historic basis. The first patients were treated with MSC from volunteer family donors. From 50ml bone marrow from a single donor, approximately 120-400 x 10^6 MSC could be cultured. This is usually sufficient for 2 infusions of 1-2 x 10^6/kg MSC for a single adult patient.
Since these first studies, this dose has been maintained for comparability and for lack of evidence that another dose is either safer or more efficacious. Indeed, although higher doses have been employed without adverse effects in certain clinical contexts, such as acute GVHD (50), available data suggest that a dose of 1-2 x10^6 cells/kg is safe, well-tolerated and capable of inducing clinical benefit (35, 37, 38, 49). The MSC dose was also chosen based on practical considerations, i.e. the ability to grow this quantity of cells for most patients within approximately 28 days (49).

6.4 Administration and concomitant monitoring

The cryopreserved investigational medicinal MSC must be thawed prior to administration. For some patients multiple bags may need to be thawed in sequence for a single administration. Body material and/or material that has come in contact with it should always be regarded as infectious and treated as such. Products must be inspected for container integrity prior to administration.
Vital signs will be monitored prior to infusion, directly following infusion and then hourly for 2 hours. Premedication will be administered as per center practice with chlorpheniramine and/or paracetamol. The infusion procedure will be performed according to the hospital's local stem cell product infusion procedures. Care will be taken to infuse the investigational medicinal product as soon as possible but maximum within 30 minutes from thawing and as rapidly as tolerated following the thawing procedure to minimize cell death. MSC will be administered by intravenous infusion using a 200 μm transfusion filter via an in-situ venous catheter of ≤ 22 gauge.
During both infusions the patient will be monitored for signs of an infusion reaction. The occurrence of infusions reactions, if present, will be recorded in detail on the patient's record forms (eCRF). The Investigator will attend the start of treatment and an intensivist will be on call throughout the whole duration of the infusion. Facilities for resuscitation will be available at patient bedside.
At both MSC producing trial sites records will be maintained of the MSC product's delivery to the clinical unit, the use by each patient, problems and irregularities during infusion, alternative disposition of unused product(s). These records will include dates, quantities, batch/serial numbers, expiration dates (if applicable), and code numbers assigned to the investigational product and trial patients.
Investigators will maintain records that document adequately that the patients were provided the doses specified by the protocol and reconcile all investigational products received from the MSC producing site.

6.5 Post-administration monitoring

Following the first MSC infusion (day 1), patients will be assessed monthly (visit 5-16). At each visit, they will be clinically evaluated, while blood and urinary laboratory parameters will be assessed at 1, 3, 6, 9 and 12 months after the first MSC infusion (see Table 1). Moreover, all information collected by the patients or by the parents of underage patients daily (see Table 2) and weekly (blood pressure) will be recorded on a sheet that will be renewed monthly, and where in addition to Albustix any symptom that may be considered an adverse reaction will be recorded in a timely and detailed fashion. For visits 6, 8,

9, 11, 12, 14 and 15 (corresponding to 2, 4, 5, 7, 8, 10, 11 months after the first MSC infusion), as they are purely an assessment of clinical well-being and remission and a check on medication dosage and tapering, patients who live far away from the Center that has performed treatment (Rome or Bergamo) will be given the option of a telephonic assessment, in which a review of the previous month's events and therapy and a plan for the following month will be performed. These will be recorded on the eCRF with specification of whether the visit is telephonic or in person. Inthis case the monthly information sheet (Table 2) will be sent by the patient before the telephonic visit by fax or e-mail and stored in the patient's records as all the others.

6.6 Concomitant medication

At enrollment, all patients will be on alternate day prednisone and at least one additional second line steroid-sparing immunosuppressive agent. Any medication taken by a subject prior to and during the course of the study, as well as the reason for using it, will be recorded. Subject will be instructed to report the use of any medication to the investigators and to consult their referring nephrologist prior to taking any medication.

Immunosuppressive drugs will be withdrawn starting 1 month after the first MSC infusion in the following order: first steroids, then calcineurin inhibitors (cyclosporin A or tacrolimus) and finally antiproliferative agents (azathioprine, micophenolate mofetil or cyclophosphamide). Immunosuppressive tapering/withdrawal will start at 1 month after the first MSC infusion by reducing the doses for prednisone by 2,5-5 mg every 1-2 weeks and for subsequent second-line agents (in the order listed above) by a quarter of the original dose every 2-3 weeks, in order to achieve complete withdrawal within 6 to 9 months. These guidelines are aimed at standardizing immunosuppressive therapy tapering/withdrawal, to limit the potential confounding effect of different approaches on data interpretation, but the Investigator will have the possibility to delay progressive dose reductions according to patient-specific conditions, or to re-introduce immunosuppressive agent that had previously been stopped if deemed necessary following disease relapse. Medications that are authorized include diuretics, vitamin D, pain relief medications, proton pomp inhibitors and other antacid drugs, ACE-inhibitors, angiotensin-converting blockers or amlodipine. Any deviation from the above guidelines, however, will be reported and justified on the patient's record forms (eCRF).

If patients experience a relapse, defined as 3+ or more positive Albustix dipsticks for proteinuria on 3 consecutive days or by positive dipsticks (1+ to 3+) for 7 consecutive days during the study period, they will be put back on daily prednisone at standard doses as per Center practice. If the reintroduction of a second-line steroid-sparing immunosuppressive agent is deemed necessary, the best approach will be chosen as per Center practice.

7 STUDY ASSESSMENTS

By entering the study, families will give consent to regular recording of daily information and prompt reporting of any changes in clinical status and/or proteinuria. Parents will be instructed to check the urine of their children for proteinuria using reagent strips (dipstick) at least every other day in the morning, every day if positive. Relapse is defined as 3+ positive dipsticks on 3 consecutive days or by positive dipsticks (1+ to 3+) for 7 consecutive days. Also, they will be asked to measure blood pressure weekly and report values at each study visit.

To record clinical parameters including urinary dipstick and monitor for potential side effects of treatment, patients or parents of underage patients will be asked to complete a spreadsheet with daily information (see Table 2).

Daily information includes:
- dipstick proteinuria (0, trace, 1+,2+,3+) (every other day)
- PDN dose
- fever >37.5° in the past 24 h (yes/no)
- cough in the past 24 h (yes/no)
- asthma in the past 24 h (yes/no)
- runny nose in the past 24 h (yes/no)
- diarrhea in the past 24 h (yes/no)
- vomiting in the past 24 h (yes/no)
- vaccination in the past 24 h (yes/no)
- antibiotic treatment in the past 24 h (yes/no)

Patients will be evaluated at study enrollment (visit 0), at time of BM aspirate for cell harvesting (and of renal biopsy for those patients who have not performed a biopsy in the last 2 years), immediately before (day 0) and at each infusion of MSC (day 1 and day 8). Subsequently they will be assessed monthly for a total of 12 months. Att 1, 3, 6, 9 and 12 months following first MSC infusion both a clinical evaluation and laboratory exams on blood and urine will be performed (corresponding to visit 5, 7, 10, 13, 16). At 2, 4, 5, 7, 8, 10, 11 months after the first MSC infusion a clinical evaluation of the monthly monitoring sheet, adverse events and therapy will be performed. These evaluations (corresponding to visit 6, 8, 9, 11, 12, 14, 15) may be, upon patient's request, performed by telephone call if the patient lives far from the Center (Rome or Bergamo) where he/she has been treated with MSC. In this case each phone call will be recorded fully on the eCRF and will take place after the patient has sent by fax or email the previous month's monitoring sheet.
Blood laboratory exams and so-called "Immunomonitoring" (detailed in Additional File 2) will be performed at baseline, at time of treatment, and after 1,3,6,9 and 12 months. Laboratory parameters assessed at enrollment and subsequently are detailed in Table 1. Creatinine clearance will be estimated by the Schwartz formula, based on serum creatinine and height in children and by the Cockroft-Gault formula, based on weight, age, sex and serum creatinine in adults. At any time patients will report adverse events to the enrolling centers. All telephone communications between families and the local investigators will be recorded on web-based forms.
At each visit, investigators will record weight, height, blood pressure, status of INS, adverse events, and concomitant medications. Results of laboratory parameters and of the daily information spreadsheets collected by participating patients/parents of underage patients will also be recorded. Data will be stored electronically in an anonymous fashion in electronic Case Report Forms (eCRF). The eCRF will record whether visits 6, 8, 9, 11, 12, 14, 15 have been performed physically or telephonically.
The study will be monitored by on-site visits (to open the center, during enrollment and at the end of the trial), that will include inspection of the eCRFs for completeness and exactness of data entered, and validation of original source documents. The monitoring visits will be conducted by independent qualified staff. Adequate time for these visits will be made available by the investigators.

Visit 0: Screening Visit and Enrollment

Investigators will inform potential subjects on the nature of the study, explain potential risks and benefits, and obtain written informed consent from patients or, if underage, from parent(s) or the subject's legal representative(s), as appropriate.
If enrolled, a study number will be assigned to each subject, based on this system: RM for patients enrolled at the Ospedale Pediatrico "Bambino Gesù" in Rome, followed by 1 to 10 in chronological order of date of signed informed consent (RM01 to RM10); BG for patients enrolled at the ASST-PG23 in Bergamo, again followed by 1 to 10 in chronological order of date of signed informed consent (BG01 to BG10).
Baseline evaluations will consist of the following:

1. General demographics (date of birth, age, gender, race)
2. Physical examination (height, weight, blood pressure, BMI)
3. Medical history (including family history): age of onset of INS, time to remission at the first episode (if available) and number of previous relapses in the past 12 months, past medications, cumulative dose of PDN in the past 12 months, past exposure to other immunosuppressive drugs, childbearing potential, chronic nephropathy, previous diseases and previous treatments.
4. Clinical laboratory tests at enrollment include:
    - infectious pathogen testing: HBV (Ab anti-HBc and HBsAg), HCV (Ab), HIV-1 and 2 (Ab), or TriNAT test (HIV RNA, HBV DNA, HCV RNA), and syphilis (Ab) within 30 days before BM aspirate and within 7 days after BM aspirate;
    - full blood count, coagulation (fibrinogen, prothrombin time, partial thromboplastin time), serum creatinine, urea, sodium, potassium, AST, ALT, alkaline phosphatase, gamma GT, glucose, uric acid, total serum cholesterol, HDL cholesterol, triglycerides, total albumin and total proteins, C-reactive protein (PCR). Creatinine clearance will be estimated by the Schwartz formula [0.5 x (height (cm) / serum creatinine (mg/dl))], based on serum creatinine and height in children and by the Cockroft-Gault formula [(140-age (years) / serum creatinine (mg/dl) x weight (kg) / 72) x 1 for male or x 0.85 for female gender], based on weight, age, gender and serum creatinine in adults.
    - urinary exams (urinalysis and proteinuria, either by 24 hour proteinuria or by spot protein/creatinine ratio) (Table 1).

Subjects will be allowed to enter the study if results of the screening evaluation meet all the inclusion and none of the exclusion criteria. All data will be recorded by the investigators on an eCRF. After enrollment patients or parents of underage patients will be requested to provide daily information by spreadsheet, which will be collected at each visit (see Table 2).
"Immunomonitoring", including total T and B cells and subpopulations will also be performed (Additional file 2).

Visit 1: BM aspirate ± renal biopsy, beginning of expansion period (1-3 months)

After verification that the subject consents and fulfills all requirements for enrollment, a brief (48-72 hour) hospitalization will be arranged in order to perform BM aspirate and, if not performed in the last 2 years, a renal biopsy (performed according to site-specific age-appropriate standard procedures). The procedure/s will be performed under sedation to avoid discomfort, with bed rest and ice pack on the

site of puncture following the procedure/s overnight. For the BM aspirate, a maximum of 50 ml of BM will be harvested under sterile conditions. The amount will be based on the patient's weight and on the sedation procedure, as per center practice. The heparinized BM will be shipped to the GMP-approved cell preparation facility of each participating institution, where MSC will be seeded and expanded, as detailed in the IMPD, according to standard operating procedures in place for storage, release, and preparation to yield autologous MSC for therapeutic use. The protocol of MSC preparation in the 2 facilities will be identical, and all procedures will be standardized to ensure reproducible results within the patient cohort. This expansion procedure should take approximately 4-6 weeks.
During this so-called expansion period, in addition to the MSC expansion in vitro, all therapy will be maintained and alternate day urinary dipstick with Albustix will be recorded to verify remission of proteinuria.

Visit 2, 3 and 4 (day 0, day +1 and day +8 day: MSC infusions)

At the end of the expansion period, if possible as early as 6 weeks following the harvesting of BM from each enrolled subject, patients will be clinically evaluated, laboratory parameters will be assessed (see Table 1) (day 0). If in order, each patient will receive 2 MSC infusions i.v. at correct dosage (1 x 10^6cells/kg pre-thawing) at day 1 and day 8. Premedication will be employed as detailed above. In addition, the following information will be recorded:

- weight
- height
- BMI
- physical examination
- blood pressure
- status of INS (relapse/remission)
- adverse events
- dose of PDN
- other medications

Laboratory parameters at time 0 will include: full blood count, urinary exams (urinalysis and proteinuria, either by 24 hour proteinuria or by spot protein/creatinine ratio) serum creatinine, urea, sodium, potassium, AST, ALT, alkaline phosphatase, gamma GT, glucose, uric acid, total serum cholesterol, HDL cholesterol, triglycerides, total albumin and total proteins, C-reactive protein (PCR).
Moreover, at time 0 "Immunomonitoring" parameters will be assessed, including IgG, IgA, IgM levels, total T and B cells and subpopulations (Additional File 2) and a Quality of Life questionnaire will be completed (CHQ-SF50 for pediatric patients, SF36 1.6 for adult patients, both in the Italian version).

Visit 5-16: Follow-up STUDY VISIT AFTER MSC COMPLETION (FOLLOW-UP):

Following the first MSC infusion (day 1), patients will be assessed monthly for a total of 12 months (visit 5-16). At each visit, they will be clinically evaluated. Urinary exams (urinalysis and proteinuria, either by 24 hour proteinuria or by spot protein/creatinine ratio), blood exams and "Immunomonitoring" will be performed at 1, 3, 6, 9 and 12 months (corresponding to visits 5, 7, 10, 13 and 16,see Table 1). The purely clinical visits at 2, 4, 5, 7, 8, 10, 11 months following the first MSC infusion (corresponding to visit number 6, 8, 9, 11, 12, 14, 15) will be aimed at collecting the monthly sheet recording any symptom compatible with a side effect, proteinuria, therapy and blood pressure, verifying correct tapering of immunosuppression and planning subsequent tapering for the following month. Patients who live far away from the Center where they have been treated (Rome or Bergamo) will be given the option to

execute this visit over the phone, if they prefer for logistical reasons. In this cse they will be required to send the monthly monitoring sheet by fax or email. Moreover, all information collected by patients or by parents of underage patients daily (see Table 2) and weekly (blood pressure) will be recorded. Investigators will fill an electronic CRF form at each visit (in person or over the phone for visits 6, 8, 9, 11, 12, 14 and 15), where they will record:

- weight
- height
- BMI will be automatically calculated
- physical examination
- blood pressure
- status of INS (relapse/remission)
- adverse events
- dose of PDN
- other medications

Laboratory parameters that will be assessed at visits 5, 7, 10, 13 and 16 include: urinary exams (urinalysis and proteinuria, either by 24 hour proteinuria or by spot protein/creatinine ratio); full blood count, serum creatinine, urea, sodium, potassium, AST, ALT, alkaline phosphatase, gamma GT, glucose, uric acid, total serum cholesterol, HDL cholesterol, triglycerides, total albumin and total proteins, C-reactive protein (PCR). (see Table 1).
"Immunomonitoring" will be assessed at visits 5, 7, 10, 13 and 16 includes IgG, IgA, IgM levels, total T and B cells and subpopulations (Additional File 2).
A Quality of Life questionnaire will be completed (CHQ-SF50 for pediatric patients, SF36 1.6 for adult patients, both in the Italian version) at months 1, 6 and 12 (corresponding to visits 5, 10 and 16).

Table 1: Data collection

Visit number	0	1	2	3	4	5	6	7	8	9	10	11	12	13	14	15	16
Time[1]	Enrollment	-1-3 m	0	1 d	8 d	1 m	2 m	3 m	4 m	5 m	6 m	7 m	8 m	9 m	10 m	11 m	12 m
Patient's Informed consent	x																
Pregnancy Test	x																
Inclusion Criteria	x																
Exclusion Criteria	x																
Baseline Data[2]	x																
Physical Examination	x		x	x	x	x	(x)	x	(x)	(x)	x	(x)	(x)	x	(x)	(x)	x
Vital Signs	x		x	x	x	x	(x)	x	(x)	(x)	x	(x)	(x)	x	(x)	(x)	x
Blood Lab Examinations[3]	x		x			x		x			x			x			x
Infectious Pathogen testing[4]	x	x															
Immunomonitoring[5]	x		x			x		x			x			x			x
Quality of Life			x			x					x						x
24 hour protu or Spot urine pr/cr ratio and Urinalysis	x		x		x	x		x			x			x			x
BM aspirate ± renal biopsy		x															
MSC infusion				x	x												

Concomitant Treatments	x
Albustix[6]	x
Adverse Events	x

[1] **Legend**: "d" means days and "m" means months. "(x)" means that the visit maybe a telephone call (optional)

[2] **Baseline data:** baseline data include:

1. General demographics (date of birth, age, gender, race)
2. Physical examination (height, weight, blood pressure, BMI)
3. Medical history (including family history): age of onset of INS, time to remission at the first episode (if available) and number of previous relapses in the past 12 months, past medications, cumulative dose of PDN in the past 12 months, past exposure to other immunosuppressive drugs, childbearing potential, chronic nephropathy, previous diseases and previous treatments.
4. Clinical laboratory tests: urinary exams (urinalysis and proteinuria, either by 24 hour proteinuria or by spot protein/creatinine ratio); infectious pathogen testing; full blood count, coagulation, serum creatinine, urea, sodium, potassium, AST, ALT, alkaline phosphatase, gamma GT, glucose, uric acid, total serum cholesterol, HDL cholesterol, triglycerides, total albumin and total proteins, C-reactive protein (PCR). Creatinine clearance will be estimated by the Schwartz or Cockroft-Gault formula.

[3] **Blood laboratory examinations**: see above EXCEPT infectious pathogen testing and coagulation

[4] **Infectious pathogen testing:** HBV (Ab anti-HBc and HBsAg), HCV (Ab), HIV-1 and 2 (Ab), or TriNAT test (HIV RNA, HBV DNA, HCV RNA), and syphilis (Ab) within 30 days before BM aspirate and within 7 days after BM aspirate

[5] **Immunomonitoring:** Total IgG, IgA, IgM, total T and B cells and subpopulations, functional tests (Additional File 2)

[6] **Albustix**: spot urine will be tested daily by albustix for proteinuria to early detect disease relapses. NS recurrence will be suspected as soon as a +++ or more positive albustix test will disclose the presence (in the case of previous negative test) or an increase (in the case of previous trace or +/++ positive test) of proteinuria.

Table 2
Daily information spreadsheet

Mese ______ (Da _____________ a _______________)

	Albustix (+)	PDN (dose)	Febbre	Tosse	Rinite	Antibiotico	Vomito	Diarrea	Asma	Vaccino	PA
1											
2											
3											
4											
5											
6											
7											
8											
9											
10											
11											
12											
13											
14											
15											
16											
17											
18											
19											
20											
21											
22											
23											
24											
25											
26											
27											
28											
29											
30											
31											

8 SAFETY MANAGEMENT

8.1 Adverse Events

Adverse events (AEs) will be collected during the entire duration of the study, including the 12 months follow-up period. All AEs, whether observed by the Investigator or reported directly by subjects, will be recorded in the subject's medical records and on the CRF, regardless of the severity of the event or potential relationship with the study medications.

Definition of Adverse Events: An adverse event (AE) is any event, side effect, or other untoward medical occurrence that occurs in conjunction with the use of a medicinal product in humans, whether or not considered to have a causal relationship to this treatment. Therefore, an AE can be any unfavorable and unintended sign (including an abnormal laboratory finding), symptom, or disease temporally associated with the use of an investigational product, whether or not related to the investigational product.
AEs do not include: Non planned medical or surgical procedures (e.g., surgery, endoscopy, tooth extraction, transfusion); conditions leading to the procedure however, are AEs. Planned surgical procedures; condition(s) leading to these procedures are not AEs. Pre-existing diseases or conditions diagnosed prior to the study entrance.

Adverse events fall into the categories "non-serious" and "serious."

8.2 Definition of a Serious Adverse Event (SAE)

A serious adverse event (SAE) is any adverse event occurring at any dose of medication that results in any of the following outcomes:
a. death;
b. life-threatening adverse drug reaction (i.e. the subject is at immediate risk of death)
c. inpatient hospitalization or prolongation of existing hospitalization, not associated with planned study procedures;
d. persistent or significant disability/incapacity

The term "life-threatening" in the definition of "serious" refers to an event in which the subject was at immediate risk of death at the time of the SAE. It does not refer to a SAE which hypothetically might have caused death if it were more severe.
Medical and scientific judgment should be exercised in deciding whether other AEs, such as important medical events that may not be immediately life-threatening, may jeopardize the subject or may require intervention to prevent one of the other outcomes listed above. These should also usually be considered SAE. Examples of such events are intensive treatment in an emergency room or at home for allergic bronchospasm, blood dyscrasias, convulsions that do not result in hospitalization, development of drug dependency or drug abuse.

8.3 Clarification of Severe and Serious Adverse Events

Clinical Laboratory Abnormalities and other Abnormal Assessments as Adverse Events and Serious Adverse Events

Abnormal laboratory findings (e.g., clinical chemistry, hematology, urinalysis) or other abnormal assessments (e.g., electrocardiogram, X-rays, vital signs) *per se* are not reported as AEs. However, those abnormal findings that are deemed clinically significant or are associated with signs and/or symptoms must be recorded as AEs if they meet the definition of an adverse event.
The Investigator should exercise his or her medical and scientific judgment in deciding whether an abnormal laboratory finding or other abnormal assessment is clinically significant.

8.4 Causality Assessment

The Investigator's causality assessment will consider the potential etiologies for the observed adverse event. An adverse event may be related to the study drug, other concomitant medications, the underlying disease pathology, an intercurrent illness or another reason. Among the potential etiologies, the investigator will make a determination based on the most likely causal relationship. When a causality assessment is provided for a serious adverse event, it is important to include a rationale for the assessment so that a better understanding of the reported event can be compiled. The rationale will be accompanied by all available supporting evidence, including relevant laboratory tests and the results of other diagnostic procedures.
Definition of Relationship to Study Medication: Association or relatedness to the study medication will be graded as either "probably," "possibly," or "unlikely." Determination of relatedness includes:

PROBABLY – The adverse event:
follows a reasonable temporal sequence from drug administration; abates upon discontinuation of the drug; cannot be reasonably explained by the known characteristics of the subject's clinical state.
POSSIBLY – The adverse event:
follows a reasonable temporal sequence from drug administration; could have been produced by the subject's clinical state or by other modes of therapy administered to the subject.
UNLIKELY – The adverse event:
does not follow a reasonable temporal sequence from drug administration; is readily explained by the subject's clinical state or by other modes of therapy administered to the subject.

8.5 Definition of Severity

All AEs will be graded if possible by the Common Terminology Criteria for Adverse Events v3.0 (CTCAE). The severity of AEs that cannot be graded by CTCAE version 3.0 will be categorized as follows:
Grade 1 – Transient or mild discomfort; no limitation in activity; no medical intervention/therapy required.
Grade 2 – Mild to moderate limitation in activity, some assistance may be needed; no or minimal medical intervention/therapy required.
Grade 3 – Marked limitation in activity, some assistance usually required; medical intervention/therapy required hospitalizations possible.
Grade 4 – Extreme limitation in activity, significant assistance required; significant medical intervention/therapy required hospitalization or hospice care probable.
Grade 5 - Death

8.6 Documenting Adverse Events

Any AE occurrence, including SAEs, during the study will be documented in the subject's medical records in accordance with the Investigator's normal clinical practice and on the CRF.

The Investigator will attempt to establish a diagnosis of the event based on the signs, symptoms and/or other clinical information. In such cases, the diagnosis will be documented as the AE (and SAE if serious) and not the individual signs/symptoms.

If a clinically significant abnormal laboratory finding or other abnormal assessment meets the definition of an AE, then the AE CRF page will be completed, as appropriate. In addition, if the abnormal laboratory finding meets the criteria for being serious, the SAE form will also be completed. A diagnosis, if known, or clinical signs or symptoms if the diagnosis is unknown, rather than the clinically significant laboratory finding or abnormal assessment, will be used to complete the AE/SAE page. If no diagnosis is known and clinical signs or symptoms are not present, then the abnormal finding will be recorded.

The SAE page will be completed and signed by the Investigator before transmittal to the Safety Committee by email within 24h.A dedicated email address farmacovigilanza.mesneph@opbg.net will be used. It is very important that the Investigator provide an assessment of the causal relationship between the event and the study drug at the time of the initial report as this will be useful for submissions to regulatory authorities.

8.7 Follow-Up of Adverse Events and Serious Adverse Events

All AEs and SAEs will be followed-up until resolution, until the condition stabilizes, until the event is otherwise explained or until the subject completes the study or is lost to follow-up. The Investigator is responsible for ensuring that follow-up includes any supplemental investigations as may be indicated to elucidate as completely as possible the nature and/or causality of the AE/SAE. This may include additional laboratory tests or investigations or consultation with other health care professionals.

8.8 IRB Notification of SAEs

The Investigator is responsible for promptly notifying the IRB of all study on-site SAEs and other unanticipated problems related to research, using the study SAE reporting form and in accordance with local requirements.

In medical emergencies, the Investigator should use medical judgment and remove the subject from the immediate hazard. The Study Safety Committee as well as the IRB/ Independent Ethics Committee must be notified regarding the type of emergency and course of action taken and the procedures for SAE reporting must be followed.

8.9 Post-Study Reporting Requirements

All SAEs, regardless of cause or relationship, which occur up to 12 months after completion of the study must be reported to the Safety Committee of the coordinating center. If the Investigator learns of any SAE at any time after a subject has been discharged from the study, and such event(s) is/are reasonably related to the study drug, the Investigator should promptly notify the principal investigator of the study.

9 STATISTICAL METHODS

Sample size calculation

The primary purpose of this study is to demonstrate the safety and tolerability of intravenous MSC infusion and to ascertain preliminary efficacy parameters. The initial efficacy in reducing INS occurrence represents the main outcome for sample size estimation, i.e. the first secondary efficacy endpoint. Patients entering the study are expected to have a 100% rate of INS recurrence after steroid/immunosuppressive therapy tapering/withdrawal. The working hypothesis is that MSC infusion may decrease the expected rate of recurrence by 30% or less. On the basis of this assumption, the inclusion of 18 patients would confer to the analyses a 90% power to demonstrate a statistically significant reduction (alpha=0.05, one tailed test) in the incidence of recurrences observed after MSC infusion (70% or less) compared to the 100% expected during treatment tapering/withdrawal without previous MSC infusion. To account for a 10% drop-out rate, 20 patients should be included into the study to have 18 patients available for statistical analyses.

Statistical analysis

This is a multicentric, open-label, prospective, non-randomized phase 1 study. For safety data, analyses will include those participants who have received some or all of a MSC infusion. The statistical analysis that we will employ for the primary endpoint will describe the incidence of adverse events in the study population. The rate of adverse events will be described by type of adverse events (reaction infusion or infections or serious adverse events). Rates of the events of interest together with their 95% confidence intervals will be estimated using a Poisson regression model.

For the secondary efficacy endpoints, analysis will include all enrolled participants. Continuous variables in the baseline characteristics will be expressed as mean ± standard deviation or as median and interquartile range. Categorical variables will be expressed as number and percentage. Follow-up data will be expressed as median and range. The Kaplan-Meier method will be used to plot the probability of achieving a relapse. Survival times for each patient will be calculated starting from MSC administration to the occurrence of the first relapse of INS. Comparisons between groups will be made using non parametric Wilcoxon signed-rank test and McNemar test, as appropriate. Mean natural course of systolic blood pressure, diastolic blood pressure, mean arterial pressure, total cholesterol, triglycerides, body weight and height will be presented in table and/or plotted over time visits.

All statistical analyses will be carried out using SAS version 9.4 or subsequent version (SAS Institute Inc., Cary, NC, USA) and STATA version 14 or subsequent version (StataCorp., College Station, TX, USA).

An interim analysis will be performed after 10 of the enrolled patients have been treated, assessing the safety of the therapeutic approach in terms both of number and severity of adverse events and in terms of number of disease relapses. Results will be evaluated by the Steering Committee, which in conjunction with the Ethical Committee and with the Safety Committee can rule the discontinuation of the study. The Safety Committee will review results every 6 months and in case of unexpected results throughout the course of the study.

10 STUDY ADMINISTRATION

10.1 Data Collection and Management

Monitoring will be performed by an independent monitor according to DM 15/7/1997, in respect of privacy rules. Throughout the course of the study, the Study Monitor will make contact with Investigators. An on-site monitoring in both participating centres and including all patients is planned, with periodical routine visits. In case of problems, for cause monitoring visits may be performed. As part of the data audit, source documents must be made available to the Study Monitor for review. The Study Monitor may also perform drug quality and administration checks and will request to perform a review of the Investigator study files and eCRFs to assure completeness of documentation in all respects of clinical study conduct. Monitoring will ensure that the study is performed according to ICH GCP, and that the protocol is adhered to. All the study documentation held by the investigators, as well as the drug logs of the study medication will be verified. A final integrated report will be filed in accordance with ICH. Monitoring and auditing procedures, developed or endorsed by Coordinator/Delegate will be followed in order to comply with GCP guidelines. Direct access to the on-site study documentation and medical records will be ensured.

Upon completion of the study, the Study Monitor will arrange for a final review of the study files, after which the files should be secured for the appropriate time period. The Investigator, or appointed delegate, will meet with the Study Monitor during the on-site visits and will cooperate in providing the documents for inspection and responding to inquiries. In addition, the Investigator will permit inspection of the study files by authorized representatives of Coordinator/Delegate or the regulatory agencies.

Domestic and foreign regulatory authorities, the IRB may request access to all source documents, CRFs and other study documentation for on-site audit or inspection. Direct access to these documents must be guaranteed by the Investigator, who must provide support at all times for these activities. Medical records and other study documents may be copied during audit or inspection provided that subject names are obliterated on the copies to ensure confidentiality.

10.2 Retention of Study Records

The following records must be retained by the Investigator, in accordance to the Italian law, for seven years.

10.3 Confidentiality

Only the subject number and subject initials will be recorded in CRF, and if the subject name appears on any other document (e.g., pathologist report), it must be obliterated in accordance with local data protection laws. Subjects will be told that IRB or regulatory authorities may inspect their medical records to verify the information collected, and that all personal information made available for inspection will be handled in strictest confidence and in accordance with local data protection laws.

The Investigator will maintain a personal subject identification list (subject numbers with the corresponding subject names) to enable records to be identified.

10.4 Documentation of Study Results

All protocol-required information collected during the study must be entered by the Investigator, or designated representative, in the CRF. Details of CRF completion and correction will be explained to

the Investigator. If the Investigator authorizes other persons to make entries in the CRF, the names, positions, signatures, and initials of these persons must be supplied to the Coordinator.
The Investigator, or designated representative, should complete the CRF pages as soon as possible after information is collected, preferably on the same day that a study subject is seen for an examination, treatment, or any other study procedure. Any outstanding entries must be completed immediately after the final examination. An explanation should be given for all missing data.
By signing the completed CRFs, the Investigator attests to the accuracy, completeness, legibility, and timeliness of the data collected on the CRF and casebooks.

10.5 Use of Study Results

The Investigator will agree to use the information only for the purposes of carrying out this study and for no other purpose unless prior written permission from Coordinator is obtained. The Coordinator has full ownership of the CRFs completed as part of the study.
By signing the study protocol, the Investigator agrees that the results of the study may be used for the purposes of publication, and information for medical and pharmaceutical professionals by Coordinator. A final report will be prepared that provides detailed results of this study. Study results will be published irrespective of the study outcome of the study.

11 REGULATORY AND ETHICAL CONSIDERATIONS

11.1 General Principles

The study will be conducted in accordance with GCP. This is a phase 1 study, and therefore safety and feasibility of this therapeutic approach will be our main focus. However, the pharmaceutical product evaluated, i.e. autologous BM-MSC, have been rather extensively used for other applications in different disease settings, and their safety profile appears to be excellent (30). Pharmacovigilance will be performed according to law (DL 211/2003) and to AIFA and ICH guidelines, as detailed in section 8. Findings judged to be clinical significant for worsening in pre-existing conditions or new onset diseases will be reported as adverse events. OPBG, the Coordinating Center, satisfies the criteria to conduct phase 1 studies according to AIFA decree dated 19/6/2015 (Gazzetta Ufficiale 158 10/7/2015).

The study protocol will be in accordance with the Declaration of Helsinki and will be approved by the Ethics Committee of each center. Written informed consent/assent has to be obtained from the patient or parents/legal representatives and from the minors before enrolment into the trial. Based on published evidence, very little relevant additional risks are expected in participating in the study, as detailed above. However, study participation does entail at least one invasive procedure (bone marrow aspirate, for some patients in addition to a renal biopsy if not performed in the previous 2 years) and 2 intravenous infusions. Potential side effects are primarily related to infections deriving from the concomitant immunosuppression that the patient is on and that will be progressively tapered as the study progresses. The total amount of blood to be drawn for the analyses (at baseline, at time of treatment and after 1, 3, 6, 9 and 12 months) is limited and not very different from that required for the routine follow-up of these difficult patients, that are on average seen every 2-3 months.

Immunomonitoring will require an additional 10-20 ml of blood, depending on the patient's age, to be drawn at each time point.

The study will be covered by an insurance policy according to the laws in force, in the event of a patient suffering any significant deterioration in health or well-being, proven to be a direct result of participation in the study.

11.2 Informed Consent

Before being enrolled in the clinical study, subjects or parents/legal representatives of underage patients must provide written consent to participate after the nature, scope, and possible consequences of the clinical study have been explained in a form understandable to them and/or their legal representative(s).

An informed consent document that includes both information about the study and the consent form will be prepared and given to the subject. The document must be in a language understandable to the subject and must specify who informed the subject. The person who informs the subject will be a physician.

After reading the informed consent document, the subject or their legal representative will give consent in writing. The subject's consent must be confirmed at the time of consent by the personally dated signature of the subject and by the personally dated signature of the person conducting the informed consent discussions.

A copy of the signed consent document will be given to the subject. The original signed consent document will be retained by the Investigator. If the subject is unable to read, oral presentation and explanation of the written informed consent form and information to be supplied to subjects must take place in the presence of an impartial witness. Consent must be confirmed at the time of consent orally and by the personally dated signature of the subject or by a local legally recognized alternative (e.g., the subject's thumbprint). The witness and the person conducting the informed consent discussions must also sign and personally date the consent document.

The Investigator will not undertake any measures specifically required only for the clinical study until valid consent has been obtained.

The Investigator will inform the subject's primary physician about the subject's participation in the trial if the subject has a primary physician and if the subject agrees to the primary physician being informed.

11.3 Payment to Subjects

There will be no payments to subjects or their legal representatives

11.4 Timing

We predict that the recruitment phase can be completed in 18 months. Follow-up will be continued until the last patient has completed 12 months post-MSC infusion. Six additional months will be required to complete data monitoring and validation, to perform statistical analyses, and to write the final report. The main scientific publication could be finalized within 40 months.

12 BIBLIOGRAPHY

1. Vivarelli, M, Massella L, Ruggiero B, Emma F. Minimal change disease. Clin J Am Soc Nephrol 2017;12(2):332-345
2. Niaudet P, Boyer, O. Idiopathic Nephrotic Syndrome in Children: Clinical Aspects. In: Pediatric Nephrology-7th edition. Springer-Verlag 2016;839-82.
3. Colucci M, Corpetti G, Emma F, Vivarelli M. Immunology of idiopathic nephrotic syndrome. Pediatr Nephrol 2017 in press
4. Bertelli R, Bonanni A, Di Donato A, et al. Regulatory T cells and minimal change nephropathy: in the midst of a complex network. Clin Exp Immunology 2016;183(2):166-74
5. Ravani P, Bonanni A, Rossi R, et al. Anti-CD20 Antibodies for Idiopathic Nephrotic Syndrome in Children. CJASN 2016;11(4):710-20.
6. Ruggenenti P, Ruggiero B, Cravedi P, et al for the Rituximab in Nephrotic Syndrome of Steroid-Dependent or Frequently Relapsing Minimal Change Disease Or Focal Segmental Glomerulosclerosis (NEMO) Study Group: Rituximab in steroid-dependent or frequently relapsing idiopathic nephrotic syndrome. JASN 2014;25(4): 850-63.
7. Vivarelli M, Moscaritolo E, Tsalkidis A, Massella L, Emma F. Time for Initial Response to Steroids Is a Major Prognostic Factor in Idiopathic Nephrotic Syndrome. J Pediatr 2010;156:965-71
8. Colucci M, Carsetti R, Cascioli S, et al. B Cell Reconstitution after Rituximab Treatment in Idiopathic Nephrotic Syndrome. JASN 2016;27(6):1811-22
9. Anolik JH, Barnard J, Owen T, et al. Delayed memory B cell recovery in peripheral blood and lymphoid tissue in systemic lupus erythematosus after B cell depletion therapy. Arthritis and Rheum 2007;56(9):3044-56.
10. Roberts DM, Jones RB, Smith RM, et al. Rituximab-associated hypogammaglobulinemia: incidence, predictors and outcomes in patients with multi-system autoimmune disease. J Autoimm 2015;57:60-5.
11. Fakhouri F, Bocquet N, Taupin P, et al. Steroid-sensitive nephrotic syndrome: from childhood to adulthood. AJKD 2003;41(3): 550-7
12. Cameron JS. The nephrotic syndrome and its complications. AJKD 1987;10(3): 157-71.
13. Bernardo ME, Fibbe WE. Mesenchymal stromal cells: sensors and switchers of inflammation, Cell Stem Cell 2013;13:392.402.
14. Nauta AJ, Fibbe WE. Immunomodulatory properties of mesenchymal stromal cells. Blood. 2007;110(10):3499-506.
15. Keating A. Mesenchymal stromal cells: new directions. Cell Stem Cell 2012;709.716.
16. Le Blanc K, Mougiakakos D. Multipotent mesenchymal stromal cells and the innate immune system. Nat Rev Immunology 2012;12:383.396
17. Peired AJ, Sisti A, Romagnani P. Mesenchymal Stem Cell-Based Therapy for Kidney Disease: A Review of Clinical Evidence. Stem Cells International 2016;4798639
18. Perico N, Casiraghi F, Remuzzi G. Clinical Translation of Mesenchymal Stromal Cell Therapies in Nephrology. Journal American Society Nephrol 2018; 29:362-375

19. Tyndall A, van Laar J. Stem cell transplantation and mesenchymal cells to treat autoimmune diseases. Presse Med 2016;45(6 Pt 2):e159-69
20. Casiraghi F, Remuzzi G, Abbate M et al. Multipotent mesenchymal stromal cell therapy and risk of malignancies. Stem Cell Rev 2013;9:65-79.
21. Le Blanc K, Frassoni F, Ball L, et al. Mesenchymal stem cells for treatment of steroid-resistant, severe, acute graft-versus-host disease: a phase II study. Lancet 2008;371(9624):1579-86.
22. Tan J, Wu W, Xu X, et al. Induction therapy with autologous mesenchymal stem cells in living-related kidney transplants. A randomized controlled trial. JAMA 2012;307(11):1169-77
23. Ankrum JA, Ong JF, Karp JM. Mesenchymal stem cells: immune evasive, not immune privileged. Nat Biotechnol. 2014;32(3):252-60.
24. Bernardo ME, Zaffaroni N, Novara F, et al. Human bone marrow derived mesenchymal stem cells do not undergo transformation after long-term in vitro culture and do not exhibit telomere maintenance mechanisms. Cancer Res 2007;67(19):9142-9
25. Starc N, Min L, Algeri M et al. Phenotypic and functional characterization of mesenchymal stromal cells isolated from pediatric patients with severe idiopathic nephrotic syndrome. Cytotherapy, 2017 in press
26. Pan X, Zheng L, Lai L. Bone marrow-derived mesenchymal stem cell therapy for decompensated liver cirrhosis: A meta-analysis. World J Gastro 2014;20(38): 14051-14057
27. Zhao R, Su Z, Wu J et al. Serious adverse events of cell therapy for respiratory diseases: a systematic review and meta-analysis. Oncotarget 2017;8(18): 30511-30523
28. Li R, Li X, Cheng J. Clinical efficacy and safety of autologous stem cell transplantation for patients with ST-segment elevation myocardial infarction. Therapeutics and Clinical Risk Management 2016;12 1171–1189
29. El-Badawy A, El-Badri N. Clinical Efficacy of Stem Cell Therapy for Diabetes Mellitus: A Meta-Analysis. PlosONE 2016;11(4):e0151938
30. Lalu M, McIntyre L, Pugliese C et al. Safety of Cell Therapy with Mesenchymal Stromal Cells (SafeCell): A Systematic Review and Meta-Analysis of Clinical Trials. PlosONE 2012;7(10):e47559
31. Introna M, Lucchin G, Dander E et al. Treatment of graft versus host disease with mesenchymal stromal cells: a phase I study on 40 adult and pediatric patients, Biol. Blood Marrow Transplant. 2014;375.381
32. von Bahr L, Batsis I, Moll G et al. Analysis of tissues following mesenchymal stromal cell therapy in humans indicates limited long-term engraftment and no ectopic tissue formation. Stem Cells 2012;30(7):1575-8
33. Bocelli-Tyndall C, Trella E, Frachet A et al. FGF2 induces RANKL gene expression as well as IL1β regulated MHC class II in human bone marrow-derived mesenchymal progenitor stromal cells. Ann Rheum Dis 2015;74(1):260-6
34. Le Blanc K, Rasmusson I, Gotherstom C et al. Mesenchymal stem cells inhibit the expression of CD25 (interleukin-2 receptor) and CD38 on phytohaemagglutinin-activated lymphocytes. Scand J Immunol 2004;60(3):307-15

35. Perico N, Casiraghi F, Introna M et al. Autologous Mesenchymal Stromal Cells and Kidney Transplantation: A Pilot Study of Safety and Clinical Feasibility. Clinical Journal American Society Nephrol 2011;6:412–422
36. Fibbe W, Rabelink T. Mesenchymal stromal cells in lupus nephritis. Nat Rev Neph 2017; 13(8):452-453
37. Connick P, Kolappan M, Crawley C et al. Autologous mesenchymal stem cells for the treatment of secondary progressive multiple sclerosis: an open-label phase 2a proof-of-concept study. Lancet Neurol 2012;11: 150.156.
38. Ciccocioppo R, Bernardo ME, Sgarella A et al. Autologous bone marrow-derived mesenchymal stromal cells in the treatment of fistulising Crohn's disease. Gut 2011; 60: 788.798.
39. Saad A, Dietz M, Hermann S et al. Autologous Mesenchymal Stem Cells Increase Cortical Perfusion in Renovascular Disease. Journal American Society Nephrol 2017;28: 2777–2785
40. Makhlough A, Shekarshian S, Mhogadasali R et al. Safety and tolerability of autologous bone marrow mesenchymal stromal cells in ADPKD patients. Stem Cell Research & Therapy 2017;8:116
41. Casiraghi F, Perico N, Cortinovis M and Remuzzi G. Mesenchymal stromal cells in renal transplantation: opportunities and challenges. Nat Rev Nephrology 2016;12:241-253
42. Belingheri M, Lazzari L, Parazzi V et al. Allogeneic mesenchymal stem cell infusion for the stabilization of focal segmental glomerulosclerosis. Biologicals 20134;1:439e445
43. Zhang Y, Peng X, Fan Z et al. Mesenchymal stem cells may ameliorate nephrotic syndrome post-allogeneic hematopoietic stem cell transplantation-Case Report Front Imm 2017;8:962
44. Zoja C, Garcia P, Rota C et al. Mesenchymal stem cell therapy promotes renal repair by limiting glomerular podocyte and progenitor cell dysfunction in adriamycin-induced nephropathy. Am J Physiol Renal Physiol 2012;303: F1370–F1381
45. Deng D, Zhang P, Guo Y et al. A randomised double-blind, placebo-controlled trial of allogeneic umbilical cord-derived mesenchymal stem cell for lupus nephritis. Ann. Rheum. Dis. 2017;76(8):1436-1439
46. Casiraghi F, Cortinovis M, Perico N, Remuzzi G. Recent advances in immunosuppression and acquired immune tolerance in renal transplants. Am J Physiol Renal Physiol. 2016;310(6):F446-53.
47. Perico N, Casiraghi F, Gotti E, et al. Mesenchymal stromal cells and kidney transplantation: pretransplant infusion protects from graft dysfunction while fostering immunoregulation. Transpl Int 2013;26:867-78
48. Koskimies O, Vilska J, Rapola J, Hallman N. Long-term outcome of primary nephrotic syndrome. Arch Dis Child 1982;57:544-8
49. Introna M, Lucchin G, Dander E et al. Treatment of graft versus host disease with mesenchymal stromal cells: a phase I study on 40 adult and pediatric patients, Biol. Blood Marrow Transplant. 2014;375–381.
50. Kebriaei P, Robinson S. Treatment of graft-versus-host-disease with mesenchymal stromal cells. Cytotherapy 2011;13(3):262-8

Additional file 1

DEPLETION OF MEMORY B CELLS AND HYPOGAMMAGLOBULINEMIA IN PATIENTS WITH IDIOPATHIC NEPHROTIC SYNDROME TREATED WITH RITUXIMAB

Study patients

From the 40 rituximab-treated nephrotic syndrome (NS) pediatric patients followed at the Ospedale Pediatrico Bambino Gesù, 7 patients, with a minimum of 5 years follow-up and with determinations of B cell subsets and circulating Igs levels were retrospectively assessed. Rituximab therapy was attempted in patients with frequent relapses despite double- or triple immunosuppressive therapy and/or a severe drug–related toxicity. Relapse was defined as proteinuria of at least 3+ for at least 3 consecutive days by urine dipstick. At baseline, all patients were on prednisone, and one (calcineurin inhibitors for 3 patients) or two (calcineurin inhibitors and mycophenolate mophetil for 4 patients) steroid-sparing agents, to maintain remission before rituximab treatment. Patients were treated with a single infusion of RTX (375 mg/m^2) during steroid-induced complete remission of their NS, followed by a second infusion at 7 days in case of non-complete depletion of total B cells defined as CD19+ B cells > 1% of the total peripheral blood lymphocytes assessed 2–7 days after the first infusion (2 patients). All patients started tapering of concomitant oral immunosuppressive treatment, and three patients relapsed after 9, 15, and 18 months, respectively. One patient who relapsed after 15 months was retreated with a single infusion of rituximab 3 months after relapse. Within 12 months, the 4 patients who maintained a prolonged remission discontinued all immunosuppressive drugs, whilst the 3 relapsers reintroduced prednisone, and restarted tapering of immunosuppressive treatment only after the induced remission. At the last follow-up (5-7 years), all patients were in remission, and the 4 non-relapsers were off-therapy, whereas the 3 relapsers were still on mycophenolate mofetil treatment.

Methods

To discriminate different B cell subpopulations, PBMCs were stained with fluorochrome-conjugated mAbs directed against CD19, CD24, CD27, CD38, IgD (BD Biosciences), and IgM (Jackson Immuno-Research Laboratories) and then analyzed by multicolor flow cytometry (FACSCanto II; BD Biosciences). Subsets of gated CD19+ (total) B cells were identified on the basis of the expression of surface markers as follows: transitional (CD38high CD24high), mature/naïve (CD38intermediate CD24low), memory (CD19pos CD27pos), IgM memory (IgMpos IgDintermediate) or switched memory (IgMneg IgDneg), and they were expressed as the percentage of total circulating lymphocytes. All analyses were performed with the FACSDiva software. Gated events (50,000) on living lymphocytes were analyzed for each sample.

Levels of total circulating immunoglobulins (Igs) were determined on freshly isolated serum samples by nephelometric assays and expressed as mg/dl.

Results

At baseline, the mean ± SEM of the percentage of total lymphocytes of CD19+ B cells was 8.6 ± 2.5 %, constituted prevalently by mature (2.7 ± 0.8%) and memory (3.2 ± 1.2%) B cells. Rituximab treatment induced a complete depletion of all the B cell subsets, and total B cells recovered after 12 months with

an initial re-emergence of transitional B cells followed by mature B cells and, finally, memory B cells, as previously described and recapitulating B cell ontogeny (1). After 5-7 years from rituximab treatment, levels of total CD19+ (11.9 ± 1.9%) and mature (6.3 ± 1.4%) B cell subsets were elevated, transitional B cells were still significantly higher than the basal levels (0.7 ± 0.2% vs 0.2 ± 0.05%, p < 0,02) and memory B cells resulted still partially depleted compared to basal levels (Fig.1), mainly in their switched compartment (0.4 ± 0.2% vs 1.7 ± 0.6%, p < 0.02) (Fig.2). In parallel with the reduced levels of switched memory B cells, levels of circulating switched IgG and IgA, normal at baseline (729 ± 30.36mg/dl IgG, and 158 ± 38 mg/dl IgA, respectively), were found to be reduced at the last follow-up (5-7 years) (516 ± 130 mg/dl IgG, and 76.3 ± 36 IgA, respectively). In contrast, total IgM levels were not affected (114 ± 25 mg/dl at last follow-up vs 133 ± 27 mg/dl at baseline). Interestingly, when we analyzed the circulating immunoglobulin levels for each patient at the last follow-up (Fig.3), we observed that levels of circulating IgG and IgA were in the normal range for the relapsed patients, but were reduced in non-relapsed patients, suggesting a pathogenic role of the isotype switched immunoglobulins. Of note, the one patient who received a second treatment of rituximab after 15 months showed reduced levels of IgG, IgM, and IgA, six years after the second infusion, suggesting that rituximab is able to reduce circulating levels of immunoglobulins.

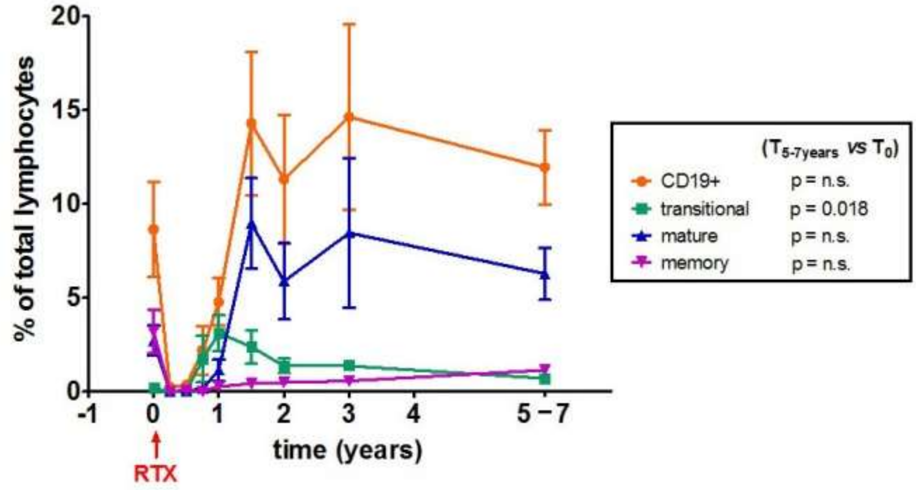

Figure 1. Reconstitution of B cell subsets after rituximab treatment. Seven steroid-dependent nephrotic syndrome pediatric patients were treated with one (5 patients) or two (2 patients) infusions of rituximab at time zero, and one patients was retreated after 15 months following relapsing. Reconstitution of total CD19+, transitional, mature and memory B cells were monitored and assessed at time 0, 3, 6, and 9 months, and after 1, 1.5, 2, 3, and 5-7 years by FACS analysis. For the patient retreated, time 0 refers to pre-rituximab treatment, and the other indicated time points (from 3 months on) have been assessed after the second rituximab infusion. Data are represented as means ± SEM.

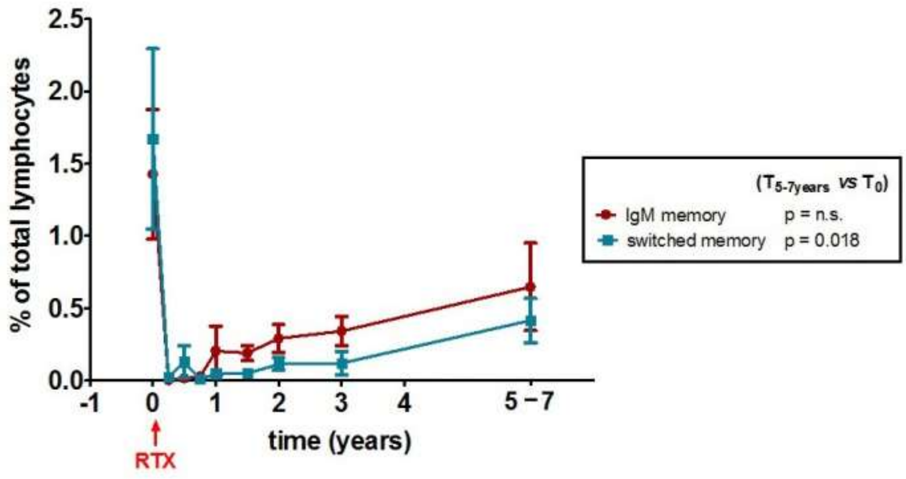

Figure 2. Reconstitution of memory B cell subsets after rituximab treatment. Reconstitution of IgM memory and switched memory B cells after rituximab treatment was monitored by FACS analysis in the same patients describer in figure 1. Data are represented as means ± SEM.

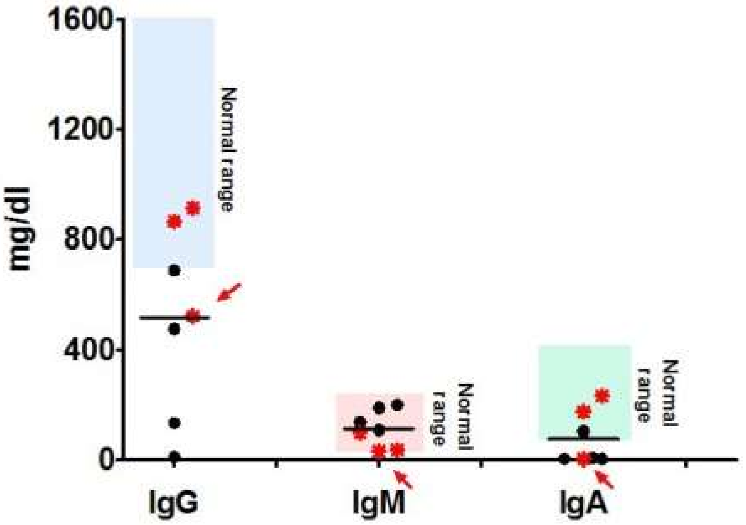

Figure 3. Levels of circulating IgG, IgM, and IgA after 5-7 years from rituximab treatment. Levels of circulating immunoglobulins (Igs) were determined by a nephelometric assay in the same patients described in figure 1. The normal ranges of IgG, IgM, and IgA are indicated by the colored areas. Levels of circulating Igs from each patient who maintained a prolonged remission (black points) or who relapsed (red asterisks) after rituximab treatment are indicated. Arrows indicate levels of circulating Igs from the one patient who received the second infusion of rituximab after 15 months.

1. Colucci M, Carsetti R, Cascioli S et al. B cell reconstitution after rituximab treatment in idiopathic nephrotic syndrome. JASN 2016;27(6):1811-22

Additional File 2: IMMUNOMONITORING

Samples for immunomonitoring, i.e. evaluation *in vivo* of the potential immunomodulatory effects of the MSC infusions in patients enrolled in the study, will be performed at baseline, and at 1, 3, 6, 9 and 12 months following MSC infusions. Total serum IgG, IgA, IgM will be dosed by standard laboratory analyses in both participating centers at the above-mentioned times.

The studies detailed below will be performed on PBMC isolated from whole blood samples in the laboratory facilities of both centers, by Dr Rita Carsetti in the OPBG Research Laboratory in Rome and by Dr Federica Casiraghi in the IRFMN Clinical Research Center in Bergamo. Whole blood samples, (10-20 ml in EDTA depending on participant's age) will be collected at baseline and at 1, 3, 6, 9 and 12 months from participating patients in both centers, and PBMCs will be isolated by Ficoll-Paque Plus density-gradient centrifugation and frozen as per standard practice.

Frozen PBMC will be appropriately labelled and stored in 2 separate vials in liquid N2 vapor until analysis or shipment. Shipments will be performed every 6 months by express courier on dry ice. Analyses pertaining to T cell subpopulations and function will be performed by Dr Federica Casiraghi in the IRFMN Clinical Research Center in Bergamo, and pertaining to B cell subpopulations and function by Dr Rita Carsetti in the OPBG Research Laboratory in Rome. Results of these analyses will be updated by both laboratories every 6 months in the reports as requested by the AIFA research project reporting timeline. The specifics of the analyses performed in both centers are detailed below. Any unused samples remaining after performing these analyses will be returned to the OPBG Research Laboratory.

T cells:

We will evaluate whether autologous MSC infusion induces regulatory T cell expansion in the peripheral blood by staining PBMC for CD3, CD4, CD25, Foxp3, CD127 and CD45 RA expression. Resting Tregs will be identify by FACS as $CD3^+CD4^+CD25^{high}Foxp3^+CD127^-CD45RA^+$ and memory Tregs as $CD3^+CD4^+CD25^{high}Foxp3^{high}CD127^-CD45RA^-$ (1).

Expression of CTLA4 and HLADR (markers of Treg activation) on resting and memory Tregs will be also performed.

The effect of MSC on number and percentages of memory ($CD45RA^-RO^+$) and naïve ($CD45RA^+RO^-$) $CD4^+$ and $CD8^+$ T cells will be also evaluated. Proliferation (by CFSE dilution assay) of and pro-inflammatory cytokine release in the supernatant will be evaluated after incubating PBMC with phytohemagglutinin A (PHA) for 72 hours.

1. Miyara M, et al. Functional delineation and differentiation dynamics of human $CD4^+$ T cells expressing the FoxP3 transcription factor. Immunity 2009, 30:899-911.

B cells:

In order to evaluate the reconstitution of the B-cell compartment we will perform cytofluorimetry staining with one 8-color tube that allows the discrimination of the 10 following populations of B cells:

transitional, naïve, IgM memory, IgG and IgA switched memory and atypical CD27neg switched memory B cells that express either IgG or IgA, plasma blasts expressing IgM, IgG, IgA.

**B Cell Tube**	Fluorochrome	V450	V500-C	FITC	PE	PerCP-Cy5.5	PE-Cy7	APC	APC-H7
	Specificity	**IgD**	**CD45**	**IgM**	**CD24**	**CD38**	**CD19**	**CD27**	**IgG**
	Clone	IA6-2	2D1	G20-127	ML5	HIT2	341113	337169	G18-145
	Cat #	561309	655873	555782	555428	551400	SJ25C1	L128	561297

CPG

The stimulation in vitro with CpG evaluates the number and function of memory and transitional B cells. PBMCs will be loaded with CFSE (Life Technologies) to track divided cells and then cultured in complete medium at a concentration of 2.5 × 10^6 cells/mL. 0.35 µM CpGB ODN2006 (Hycult Biotech) will be used to stimulate the cells for 7 days. Cultured cells will be harvested and stained with the appropriate combination of fluorochrome-conjugated antibodies to identify B-cell subsets. At least 50.000 events gated on living cells will be analyzed, whenever possible, for each sample. Samples will be acquired on a BD Fortessa X-20. Secreted antibodies in the supernatant of stimulated cells will be measured by ELISA.

1. Human B-cell memory is shaped by age- and tissue-specific T-independent and GC-dependent events.
Aranburu A, Piano Mortari E, Baban A, Giorda E, Cascioli S, Marcellini V, Scarsella M, Ceccarelli S, Corbelli S, Cantarutti N, De Vito R, Inserra A, Nicolosi L, Lanfranchi A, Porta F, Cancrini C, Finocchi A, Carsetti R.
Eur J Immunol. 2016 Nov 16. doi: 10.1002/eji.201646642. [Epub ahead of print]

2. B-cell activation with CD40L or CpG measures the function of B-cell subsets and identifies specific defects in immunodeficient patients.
Marasco E, Farroni C, Cascioli S, Marcellini V, Scarsella M, Giorda E, Mortari EP, Leonardi L, Scarselli A, Valentini D, Cancrini C, Duse M, Grimsholm O, Carsetti R.
Eur J Immunol. 2016 Nov 1. doi: 10.1002/eji.201646574. [Epub ahead of print]

3. Longitudinal Evaluation of Immune Reconstitution and B-cell Function After Hematopoietic Cell Transplantation for Primary Immunodeficiency.
Scarselli A, Di Cesare S, Capponi C, Cascioli S, Romiti ML, Di Matteo G, Simonetti A, Palma P, Finocchi A, Lucarelli B, Pinto RM, Rana I, Palumbo G, Caniglia M, Rossi P, Carsetti R, Cancrini C, Aiuti A. J Clin Immunol. 2015 May;35(4):373-83. doi: 10.1007/s10875-015-0154-4.

4. Inhibition of B-cell proliferation and antibody production by mesenchymal stromal cells is mediated by T cells.
Rosado MM, Bernardo ME, Scarsella M, Conforti A, Giorda E, Biagini S, Cascioli S, Rossi F, Guzzo I, Vivarelli M, Dello Strologo L, Emma F, Locatelli F, Carsetti R.
Stem Cells Dev. 2015 Jan 1;24(1):93-103. doi: 10.1089/scd.2014.0155.

5. Evaluating B-cells: from bone marrow precursors to antibody-producing cells.
Rosado MM, Scarsella M, Cascioli S, Giorda E, Carsetti R.
Methods Mol Biol. 2013;1032:45-57. doi: 10.1007/978-1-62703-496-8_4.

6. TLR ligation triggers somatic hypermutation in transitional B cells inducing the generation of IgM memory B cells.
Aranburu A, Ceccarelli S, Giorda E, Lasorella R, Ballatore G, Carsetti R. J Immunol. 2010 Dec 15;185(12):7293-301. doi: 10.4049/jimmunol.1002722.

7. CpG drives human transitional B cells to terminal differentiation and production of natural antibodies.
Capolunghi F, Cascioli S, Giorda E, Rosado MM, Plebani A, Auriti C, Seganti G, Zuntini R, Ferrari S, Cagliuso M, Quinti I, Carsetti R.
J Immunol. 2008 Jan 15;180(2):800-8.

Investigator Brochure MESNEPH

Study Title: A prospective study to assess safety and efficacy of the use of bone-marrow derived MESenchymal stromal cells as immunomodulatory therapy for children and young adults with severe and difficult-to-treat frequently relapsing or steroid-dependent idiopathic NEPHrotic syndrome: the MESNEPH study

Short title: MSC as therapy for INS: phase 1study

Product: Autologous bone marrow-derived mesenchymal Stromal cells (Aut-MSC); Somatic cell therapy medicinal product; sterile, injectable, small volume

Producers:

1) Officina Farmaceutica of the Ospedale Pediatrico "Bambino Gesù", Rome, GMP compliant and authorized by AIFA for Cellular Therapy Medicinal Product. Qualified person: Franca Fassio
2) Center of Cellular Therapy "G. Lanzani", part of the Division of Hematology of the ASST Papa Giovanni XXIII,GMP compliant and authorized by AIFA for Cellular Therapy Medicinal Product. Qualified person: Martino Introna

Principal Investigator: Marina Vivarelli

Co-investigators: Giuseppe Remuzzi, Franco Locatelli, Francesco Emma, Mattia Algeri, Norberto Perico, Martino Introna, Rita Carsetti, Federica Casiraghi

Sponsor: Ospedale Pediatrico "Bambino Gesù"- IRCCS

EUDRACT number:2016-004804-77

Protocol number: 1262_OPBG_2018

Date: February 27, 2018

Version: 01

Confidential Statement

This Investigator Brochure contains strictly confidential information

which is not to be communicated or published unless

previously authorized by

Bambino Gesù Children's Hospital, IRCCS

TABLE OF CONTENTS

Abbreviations pg. 6

1. SUMMARY pg. 8

2. INTRODUCTION pg. 9
2.1 Idiopathic nephrotic syndrome pg. 9
2.2 Unmet therapeutic needs in idiopathic nephrotic syndrome pg. 11
2.3 Mesenchymal stromal cells and their therapeutic potential pg. 12
2.4 Study rationale pg. 13

3. STUDY DESIGN AND PATIENT ENROLLMENT pg. 14
3.1 Primary objective pg. 14
3.2 Secondary objectives pg. 15
3.3 Patient inclusion and exclusion criteria pg. 15
3.4 MSC treatment pg. 16
3.5 Clinical follow-up pg. 16
3.6 Immunomonitoring pg. 17
3.7 Sample size pg. 17

4. MANUFACTURING OF THE PRODUCT pg. 18
4.1 Product description and formulation pg. 18
4.2 Biological, physical, chemical and pharmaceutical properties of MSC pg. 18
4.3 Drug product formulation pg. 19
4.4 Excipients of MSC products pg. 19
4.5 Storage and handling of the MSC product pg. 22

5. PRE-CLINICAL STUDIES pg. 22
5.1 Mechanism of action and pharmacodynamics of MSCs: in vitro evidence pg. 22
5.1.1 Immunomodulatory activities of MSC in vitro pg. 23
5.1.2 Properties of autologous MSC from patients with INS in vitro pg. 24
5.2 Mechanism of action and pharmacodynamics of MSC: animal models pg. 30
5.2.1 Adriamycin-induced nephrotic syndrome pg. 30
5.2.2 Kidney transplant pg. 32
5.2.3 Repair of kidney damage pg. 34
5.3 Pharmacokinetics and Immunogenicity of MSC: animal studies pg. 35
5.4 Conclusions from pre-clinical studies pg. 37

6. EFFECTS IN HUMANS - CLINICAL STUDIES pg. 38
6.1 Overview of safety and efficacy pg. 38
6.2 Immunomodulatory activities of MSC: evidence from clinical studies pg. 39

6.3 Safety and Efficacy of MSC infusion for kidney diseases pg. 40
6.3.1 Results of the therapeutic use of MSC in lupus nephritis pg. 43
6.3.2 Results of the therapeutic use of MSC in focal segmental glomerulosclerosis pg. 43
6.3.3 Results of the use of MSC in nephrotic syndrome post-allogeneic hematopoietic stem cell transplant pg. 44
6.3.4 Results on the use of MSC in renal transplantation pg. 44
6.4 Pharmacodynamic interactions pg. 45
6.5 Conclusions from clinical studies pg. 45

7. SPECIFIC SAFETY ISSUES pg. 46
7.1 Risk assessment of raw material pg. 46
7.2 Risk assessment in connection with sterility pg. 47
7.3 Adverse reactions, precautions and handling of overdose pg. 47
7.3.1 Adverse reactions and precautions pg. 47
7.3.2 Overdose pg. 48
7.4 Risk of neoplastic transformation pg. 48
7.5 Reproductive toxicity and teratogenicity pg. 49
7.6 Conclusions on potential risks pg. 49
7.7 Benefits pg. 49

8. CONCLUSIONS pg. 50

9. REPORTING REQUIREMENTS pg. 50

REFERENCES pg. 52

APPENDIX 1 Project Plan MESNEPH protocol pg. 63

APPENDIX 2 Infusion Report MESNEPH protocol pg. 69

LIST OF TABLES

Table 1: Properties of the drug substance according to the certificate of analysis pg.19

Table 2: List of excipients of MSC pg. 20

Table 3: Allogeneic MSC survival time in different animal models pg. 35

Table 4: Human trials of MSCs for kidney disease registered in ClinicalTrials.gov at February 21, 2018 pg. 41

LIST OF FIGURES

Figure 1: Putative immunomodulatory mechanism of action of mesenchymal stromal cells (MSCs) pg. 24

Figure 2:Phenotypic and differentiative features of autologous MSCs expanded from children with INS compared to those expanded from healthy controls pg. 26

Figure 3: In vitro immunomodulatory effect of HD- and INS-MSCs on healthy donor peripheral blood mononuclear cells (PBMCs) in an allogeneic setting pg. 28

Figure 4: *In vitro* immunomodulatory effect of HD- and INS-MSCs on B-cell proliferation and plasma-cell (PC) differentiation in an allogeneic setting pg. 29

Figure 5: Effects of HD-MSC and INS-MSC on albumin permeability in podocyte-endothelial cell co-culture pg. 30

ABBREVIATIONS

ACTH	Adrenocorticotropic hormone
ADR	Adriamycin
aGvHD	Acute Graft versus Host disease
AIFA	Agenzia Italiana del Farmaco
AKI	Acute kidney injury
AN	Adriamycin nephropathy
ASST	Azienda Socio Sanitaria Territoriale
ATMP	Advanced Therapy Medicinal Products
BM	Bone Marrow
BM-MSC	Bone Marrow derived Mesenchymal stromal Cells
BSA	Bovine serum albumin
CD2AP	CD2-associated protein
CF	Cell Factory
CFMDA	5-chloromethylfluorescein diacetate
DC	Dendritic cell
DMSO	DiMethylSulphOxide
DP	Drug Product
DS	Drug Substance
EBMT	European Bone Marrow Transplantation
EMA	European Medicines Agency
EU	Endotoxin Units
FACS	Fluorescent Activated Cell Sorting
FRNS	Frequently relapsing nephrotic syndrome
FSGS	Focal segmental glomerulosclerosis
GMP	Good Manufacturing Practice

HD	Healthy donor
HLA	Human Leukocyte Antigen
IB	Investigator Brochure
IMPD	Investigational Medicinal Product Dossier
INS	Idiopathic nephrotic syndrome
iPS	Induced pluripotent stem cells
ISKDC	International Society of Kidney Disease in Children
ISS	Istituto Superiore di Sanità
iv	Intravenous
JACIE	Joint Accreditation Committee-ISCT & EBMT
MCD	Minimal change disease
MCP-1	Monocyte chemoattractant protein 1
MLR	Mixed lymphocyte reaction
MMF	Mycophenolate mofetil
MSC	Mesenchymal Stromal Cells
NOD-SCID	Non-obese diabetic-Severe combined immunodeficiency
OF	Officina Farmaceutica
OPBG	Ospedale Pediatrico Bambino Gesù
PBMC	Peripheral blood mononuclear cell
PHA	Phytohaemagglutinin
PEC	Progenitor parietal epithelial cells
QC	Quality control
SDNS	Steroid-dependent nephrotic syndrome
SOP	Standard Operating Procedure

1. SUMMARY

This Investigator Brochure relates to a Phase 1 study on the administration of the somatic cell product autologous mesenchymal stromal cells (Aut-MSC) to patients, both children and young adults, affected by severe forms of steroid-dependent or frequently relapsing idiopathic nephrotic syndrome.

Patients will be treated in two centers: the Ospedale Pediatrico “Bambino Gesù” in Rome, Italy and the Azienda Socio-Sanitaria Territoriale Papa Giovanni XXIII, Bergamo, Italy.

In the present study we will evaluate the safety and feasibility of the systemic infusion of autologous bone marrow-derived *ex-vivo* expanded MSC in children and young adults with idiopathic nephrotic syndrome. In particular, 20 patients, 10 from each center, will receive two infusions of autologous MSC derived from 50 ml of their bone marrow aspirated 2-3 months previously and *ex vivo* expanded by the GMP compliant and authorized Cell Factory facilities of the two participating centers. The 2 infusions will be 7 days apart and at the dose of 1 x 10^6/kg of body weight (range 0,75 – 1,25 x 10^6/kg of body weight). Following the infusions, 1 month after the first infusion, concomitant immunosuppression with be gradually tapered with the objective, in the absence of disease relapses, of discontinuing all oral immunosuppression in 6-9 months. The tapering protocol will be kept as uniform as clinically feasible between all patients enrolled in the study. During the 2 infusions and subsequently for the following 12 months, patients will be closely monitored and any clinical event interpretable as an adverse reaction or adverse event will be carefully recorded. Patients will record any such event daily at home on a monthly spreadsheet, and will be evaluated clinically monthly, while laboratory exams will be performed after 1, 3, 6, 9 and 12 months. In such visits, in addition to physical examination, vital parameters, therapy and medical history, blood and urine laboratory exams will be performed. Urinary dipstick by Albustix will be performed on alternate days at home as is standard practice for patients affected by nephrotic syndrome, and blood pressure will be recorded weekly as well.

In addition to standard laboratory exams, at baseline and following the MSC infusions a panel of descriptive and functional assays will be performed on peripheral blood mononuclear cells isolated from full blood of all patients enrolled in order to assess the immunomodulatory effect of the MSC *in vivo* in this clinical setting.

MSC will be expanded in human Platelet Lysate according to an established protocol in the authorized Cell Factory under GMP conditions starting from 50 ml of autologous bone marrow as detailed in the IMPD.

Our group has previously shown (1) that the well-established immunomodulatory properties of MSC are maintained in *in vitro* experiments performed on MSC expanded *ex vivo* from the bone marrow of 5 pediatric patients with difficult forms of idiopathic nephrotic syndrome, clinical features identical to the patients that we plan to enroll in this study. These patient-derived MSC were also able to ameliorate glomerular filtration barrier damage simulated *in vitro* by exposure to Adriamycin, reducing albumin leakage. Moreover, these MSC maintain a differentiative and proliferative potential that is fully comparable to MSC derived from healthy controls in an *in vitro* setting.

Animal studies and two case reports provide additional evidence of the potential therapeutic value of this therapeutic approach in idiopathic nephrotic syndrome, as detailed below.

Acute toxicity has never been described in more than 3000 patients treated so far by MSC and specifically in over 100 patients treated with cellular products prepared in the “G. Lanzani” Cell Factory with the identical protocol that we will employ for this study.

The MSC lots will arrive at the Hospital as a frozen material which will have to be thawed at 37°C and immediately infused.

Documents will have to be prepared by the Medical Doctor in charge to comply with the National laws and requirements of the Istituto Superiore di Sanità (ISS).

2. INTRODUCTION

This Investigator’s Brochure (IB) presents information relating to the somatic cell therapy product derived from the isolation and expansion of human derived bone marrow (BM) for use in the trial “A prospective study to assess safety and efficacy of the use of bone-marrow derived MESenchymal stromal cells as immunomodulatory therapy for children and young adults with severe and difficult-to-treat frequently relapsing or steroid-dependent idiopathic NEPHrotic syndrome: the MESNEPH study”. This product is classified as ‘somatic cell therapy medicinal product’.

2.1 Idiopathic nephrotic syndrome

Idiopathic nephrotic syndrome (INS) is the most frequent glomerular disease in children (2,3). The incidence is around 1-3/100.000 children below 16 years of age. Its clinical manifestation is edema, and laboratory exams reveal intense proteinuria, leading to hypoalbuminemia and therefore to intravascular volume depletion and oliguria. All children at onset are treated with a standard course of oral prednisone, to which the majority (75-90%) respond within a few days/weeks. Patients who do not respond are defined as steroid resistant, and about half of these will subsequently respond in a variable amount of time to second-line immunosuppression. In those who maintain steroid-resistance often a genetic defect of podocyte components leading to disruption of the integrity of the glomerular basement membrane is the cause of disease. All forms that are steroid-sensitive are instead defined as INS, to indicate that the mechanism determining proteinuria is not fully elucidated (3). However, a vast body of experimental evidence, coupled with the clinical response of INS to immunosuppressive therapy, clearly implicates the immune system in disease pathogenesis, through its production of factors that damage the podocyte (4). T cells have been implicated in this process, and a reduction of T regulatory cells has been shown (5). The role of B cells, in particular memory B cells, is suggested by the effectiveness of their depletion by anti-CD20 therapy in preventing relapses (6-7).

Most patients have a relapsing course of INS that is treated with corticosteroids, mostly with prednisone (PDN). The frequency of relapses permits the classification of INS in subtypes reflecting disease severity. These include “non relapsing nephrotic syndrome”, “infrequently relapsing nephrotic syndrome”, “frequently relapsing nephrotic syndrome” (FRNS) and “steroid-dependent nephrotic syndrome” (SDNS) (1). This classification has been shown to have a prognostic value that exceeds the relevance of renal pathology findings. Accordingly, a renal biopsy is not indicated in most cases, and if performed most patients are found to have alterations that are visible only by electron microscopy, where effacement of foot processes is found, and little else (so-called “minimal change disease”). Outcome of INS is extremely variable. In a recent review of the long-term evolution of 103 Italian children with steroid-sensitive INS that were treated since their first episode at the "Ospedale Pediatrico Bambino Gesù”, 67% of patients were found to have FRNS or SDNS (8); these data are in agreement with previous studies that were performed in the 1980's. In a small but significant group of patients (around 15-20% of relapsing forms) the disease is severe and prolonged, lasting into adulthood.

2.2 Unmet therapeutic needs in idiopathic nephrotic syndrome

Corticosteroids have been used to treat childhood nephrotic syndrome since 1950 when large doses of adrenocorticotrophic hormone (ACTH) and cortisone given for two to three weeks were found to induce diuresis with loss of edema and proteinuria (3). Since then, oral corticosteroids are the first-line treatment of a child presenting with INS (no controlled prospective trials of corticosteroids compared to placebo have been carried out). However corticosteroids are known to have adverse effects such as obesity, stunted linear growth, hypertension, diabetes mellitus, osteoporosis, cataract, cushingoid features and adrenal suppression (3). Adverse effects are particularly prevalent in those children who relapse frequently and thus require multiple courses of steroids. For these patients, the challenge resides in finding a therapeutic strategy that allows maximal steroid sparing while limiting the relapse rate, based on the child's clinical response and drug-related adverse effects. The original treatment schedules for childhood INS were developed in an ad hoc manner. The International Study of Kidney Disease in Children (ISKDC) was established in 1966 and determined by consensus a regimen for the first episode of daily prednisone (PDN 60 mg/m^2) for four weeks, followed by PDN given on alternate days for four weeks (40 mg/m^2). In our experience, approximately 50% of children relapse within 3 months with this regimen. Most importantly, the bulk of data that has accumulated over the past decades indicates that corticosteroids are not disease-modifying drugs, i.e. they have little effect on the long-term evolution of the disease.

If toxicity develops, alternative drugs are generally introduced, including calcineurin inhibitors (cyclosporin A or tacrolimus), mycophenolate mofetil (MMF) and cyclophosphamide. Chronic immunosuppression, however, seldom achieves persistent remission and is invariably burdened by serious adverse effects including gonadotoxicity and sterility, opportunistic infections, malignancies, bone marrow depression and renal toxicity. In our experience and based on the existing literature, these medications need to be used in more than 50% of children within 5 years of their disease (3). Eventually, most patients achieve permanent remission, but 20-25% still relapse in adulthood (3, 9).

In the last decade, the use of anti-CD20 monoclonal antibodies, which temporarily deplete circulating B cells, has proved effective in the treatment of a substantial number of children with INS, most often frequently relapsing and steroid-dependent forms (6,7). We and others have shown that following this treatment reconstitution of a subgroup of circulating B cells, namely switched memory B cells, predicts relapse (10). Moreover, in nephrotic syndrome and more extensively in other immune-mediated diseases where it is routinely employed, some patients post-rituximab

experience prolonged hypogammaglobulinemia and maintain low levels of memory B cells for many years following treatment (see Additional File 1 of MESNEPH protocol, 11, 12).

Therefore, in a small but significant group of patients INS is severe and prolonged, lasting into adulthood (3, 13) and requiring much dedication by the family and resources by the National Health System to maintain stable remission and minimize the impact of this chronic disease on the patient's well-being. In these patients, multidrug immunosuppressive therapy leads to significant morbidity and to reduced compliance (13). There is therefore an unmet need for effective and less toxic therapies.

In summary:

1) the etiology of INS remains unclear;

2) response to immunosuppression suggests an immune dysregulation leading to glomerular basement membrane disruption and proteinuria;

3) currently, no disease-modifying drugs have been identified;

4) in INS, disease morbidity is largely attributable to the frequency of relapses, and to the side-effects of drugs (mainly PDN) that are used to treat and prevent these relapses;

5) therefore, new therapeutic approaches with potential and a promising safety profile are much needed.

2.3 Mesenchymal stromal cells and their therapeutic potential

The bone marrow stroma contains, in addition to hematopoietic stem cells, a population of pluripotent marrow stromal cells (i.e., MSC), capable of differentiation into fibroblasts, osteoblasts, adipocytes and chondrocyte progenitors (14-16). This heterogeneous population of cells provides growth factors, cell-to cell interactions, and matrix proteins that play a role in the regulation of hematopoiesis (17). Moreover, MSC have been shown to redistribute into a wide range of tissues contributing to tissue repair (18), and several studies have indicated that MSC play a role in modulation of in vitro and in vivo immune responses (19-21). MSC themselves are of inherently low immunogenicity due to their immune phenotype, the low expression of human leukocyte antigen (HLA) class II, and the absence of costimulatory molecules. Cultured and expanded MSC do not stimulate T cell proliferation in Mixed Lymphocyte Reactions (MLR) and are able to down regulate allo-reactive T cell responses when added to MLR (22). The bone marrow serves as the primary reservoir for MSC, but their presence has been reported in a variety of other tissues. These include

adipose tissue, periosteum, muscle connective tissue, fetal bone marrow, fetal liver and blood, and cytokine (G-CSF) mobilized peripheral blood (23-28). Although a number of markers are expressed on MSC that can be used to enrich MSC from populations of adherent bone marrow stromal cells (29), at present no unique phenotype has been identified that allows the reproducible isolation of MSC precursors with predictable developmental potential. Nevertheless, minimal criteria for defining MSC have been defined by the International Society for Cellular Therapy, which include the expression of CD105, CD73 and CD90 as positive markers for MSC (30). The isolation of stromal cell function further relies primarily on their ability to adhere to plastic.

Multiple studies have demonstrated that MSC are endowed with unique immunomodulatory and anti-inflammatory properties directed towards cells involved in both the adaptive and innate immune responses (31, 32) (see section 4.1.1). The therapeutic use of allogeneic third-party MSC has been evaluated in a number of clinical settings and of renal diseases, including graft versus host disease, lupus nephritis, renal transplantation, relapse of nephrotic syndrome following renal transplantation and nephrotic syndrome post-allogeneic bone marrow transplant (33-38). Moreover, many studies demonstrate that the use of autologous MSC obtained from patient's tissues can be a feasible, safe and beneficial therapy in several diseases, including autoimmune diseases and renal diseases (39-50).

The major advantages of utilizing autologous MSC for the treatment of nephrotic syndrome are: i) an easily accessible product manufactured from a good quality source of autologous bone-marrow derived MSC, ii) the reduction of the risk of rejection and immunization due to the lack of immunogenicity, particularly in the case of recipients that are not profoundly immunosuppressed.

2.4 Study rationale

The rationale of the MESNEPH study is to investigate the safety, feasibility and therapeutic potential of the medicinal use of autologous bone marrow-derived mesenchymal stromal cells in children and young adults with severe, recurring forms of immune-mediated nephrotic syndrome. The goal is to clarify whether or not this therapeutic approach may merit further investigation.

We propose to evaluate the safety and feasibility of the therapeutic use of autologous MSC harvested and purified from the bone marrow (BM) of children and young adults with severe, recurring forms of immune-mediated nephrotic syndrome, expanded ex vivo and reinfused to the

same patient. Given their immunomodulatory properties, our working hypothesis is that their therapeutic use will permit relapse-free reduction of oral immunosuppression in INS patients.

3 STUDY DESIGN AND PATIENT ENROLLMENT

Twenty patients with severe frequently relapsing or steroid-dependent forms of nephrotic syndrome will be enrolled in 2 centers, the IRCCS Ospedale Pediatrico Bambino Gesù in Rome and the ASST Papa Giovanni XXIII –in Bergamo. At baseline a bone marrow aspirate will be performed, and MSC will be isolated and expanded in the Good Manufacturing Practice-approved Cell Factory facilities of both centers for therapeutic use. Identical protocols for preparation of the medicinal product will be employed. Each patient will be treated with 2 doses of autologous bone marrow-derived MSC at a 7-day interval and subsequently followed for 12 months. After treatment, oral immunosuppression will be gradually tapered in 6-9 months and the number of relapses of proteinuria will be monitored with daily Albustix. Clinical evaluation will occur monthly following the first infusion, while laboratory parameters, including full blood exams and evaluation of B and T cell subsets and function will be performed at baseline, at time of infusion, and after 1, 3, 6, 9 and 12 months. All adverse events will be carefully recorded. The study will be performed according to Good Clinical Practice with independent monitoring, pharmacovigilance and overview by a Safety Committee.

This phase 1 study is expected to provide results on the safety and feasibility of the therapeutic use of bone marrow-derived autologous MSC in children and young adults with severe, difficult-to-treat INS.

3.1 Primary objective

To assess the feasibility and safety of harvesting from their bone marrow, isolating and expanding *ex vivo* in an approved facility and then infusing autologous MSC in children and young adults with severe multi-relapsing or steroid-dependent INS.

Two infusions of autologous MSC 7 days apart will be performed, at the dose of 1 x 10^6 cells/kg of body weight. At the time of infusion, allergic reactions and all vital parameters will be monitored. Infusional toxicity, if present, will be assessed and recorded. Infusions will take place in a hospital setting with monitoring of all vital parameters and medical evaluation. Following this therapy,

patients will be evaluated clinically and with a complete blood and urine work-up at 1, 3, 6, 9 and 12 months, and any adverse events, such as infections, will be recorded. Concomitant immunosuppression with oral prednisone and one or more other oral agents will be gradually tapered.

3.2 Secondary objectives

- To evaluate whether the therapeutic use of autologous MSC is able to prevent disease relapse despite tapering of prednisone and/or other immunosuppressive treatments in children and young adults with severe multi-relapsing or steroid-dependent INS.
- To assess whether the therapeutic use of autologous MSC may reduce the need for prednisone and/or other immunosuppressive agents to prevent and treat further disease relapses;
- To evaluate whether tapering or withdrawal of immunosuppressive therapy is associated with regression of the related toxicities, such as growth retardation, hypertension, impaired glucose tolerance and obesity, infections, B cell suppression;
- To evaluate the immunomodulatory effect of the MSC infusion in vivo in a clinical setting.

3.3 Patient inclusion and exclusion criteria

INCLUSION CRITERIA

- Males and females aged 5 to 40 years.
- Steroid-dependent or multirelapsing INS patients with 2 or more relapses in the previous year in spite of prednisone and/or one or more other immunosuppressive steroid-sparing agent. Only patients reported to invariably relapse upon treatment tapering or withdrawal who are on stable (from at least 1 month) complete (<0.3 g/24h for adults or <4 mg/h/m^2 for children) or partial (<3.5 g/24h for adults or <40 mg/h/m^2 for children) remission of the INS will be included. Rituximab treatment can have been previously performed but reconstitution of B cells, defined as total CD19/CD20 lymphocyte count above 5% of total lymphocytes by cytofluorimetry, must have occurred and must be recorded.
- histological diagnosis of MCD, FSGS, mesangial proliferative glomerulonephritis;
- written informed consent (or consent from parents or tutors for underage patients, as appropriate).

EXCLUSION CRITERIA

- Advanced renal failure (creatinine clearance less than 20 ml/min/1.73m^2), calculated using the Schwartz formula or the Cockroft-Gault formula, as appropriate;
- refractory or persistent NS;
- genetic mutations associated with intrinsic abnormalities of the glomerular barrier;
- pregnancy or lactating;
- women of childbearing potential without following a scientifically accepted form of contraception;
- infectious pathogen testing positive for active infection;
- legal incapacity;
- evidence of an uncooperative attitude;
- previous diagnosis of: intellectual disability/mental retardation, dementia, schizophrenia.
- any evidence that patient will not be able to complete the trial follow-up.

3.4 MSC treatment

Following enrollment and baseline evaluations, a bone marrow aspirate will be performed (50 ml of blood will be aspirated in a heparinated syringe). Patients will be sedated to avoid discomfort. If not previously performed in the last 2 years, at the same time patients will also undergo renal biopsy. The patients will then enter a 2-month run-in period during which all therapy will be maintained and daily urinary dipstick with Albustix will be recorded to verify remission of proteinuria. During this period, the bone marrow will be used in a GMP-approved Cell Factory facility either in Rome or in Bergamo to purify and expand an adequate amount of MSC for therapeutic use. The protocol of MSC preparation in the 2 facilities will be identical, and all procedures will be standardized to ensure reproducible results within the patient cohort. After the run-in period, each patient will receive 2 intravenous infusions of autologous MSC 7 days apart. One month after the first MSC infusion, steroid and/or concomitant immunosuppressive therapy will be tapered and progressively withdrawn over 6 to 9 months.

3.5 Clinical follow-up

Following study enrollment, patients will receive a monthly spreadsheet, where they will be asked to record daily their proteinuria assessed by Albustix as is standard practice for their disease, weekly

their blood pressure, and any relevant symptom or therapeutic modification. Following the first MSC infusion (day 1), patients will be evaluated clinically monthly for 12 months. Moreover, laboratory parameters will be assessed at 1, 3, 6, 9 and 12 months. To assess the safety profile of this therapeutic approach, all information collected by the parents daily and weekly (blood pressure) will be recorded. Investigators will fill an electronic CRF form at each visit, where they will record:

- weight
- height
- BMI will be automatically calculated
- physical examination
- blood pressure
- status of INS (relapse/remission)
- adverse events
- dose of PDN
- other medications

Laboratory parameters include: creatinine, urea, sodium, potassium, AST, ALT, alkaline phosphatase, gamma GT, glucose, uric acid, total proteins, albumin, total cholesterol, HDL cholesterol, triglycerides, leukocytes, neutrophils, lymphocytes, monocytes, eosinophils, basophils, erythrocytes, hemoglobin, hematocrit, and platelets, C reactive protein (CRP), procalcitonin (PCT).

3.6 Immunomonitoring

To evaluate the immunomodulatory effect of the 2 MSC infusions *in vivo*, an in-depth descriptive and functional evaluation of T and B cells, so-called "Immunomonitoring", will be performed at baseline and at 1, 3, 6, 9 and 12 months post-infusion of autologous MSC in all study participants. Immunomonitoring includes total serum IgG, IgA, IgM levels, total T and B cells and subpopulations, and the functional evaluations listed in Additional File 2 of the MESNEPH protocol.

3.7 Sample size

The study proposes to enroll 20 patients, 10 from Rome and 10 from Bergamo. This is a pilot study and its primary objective is to investigate feasibility and safety. The initial efficacy in reducing INS recurrence represents the main outcome for sample size estimation, i.e. the first secondary efficacy endpoint. Based on the working hypothesis that MSC infusion may decrease the expected rate of recurrence of 30% or less, the inclusion of 18 patients would confer to the analyses a 90% power to

demonstrate a statistically significant reduction (alpha=0.05, one tailed test) in the incidence of recurrences observed after MSC infusion (70% or less) compared to the 100% expected during treatment tapering/withdrawal without previous MSC infusion. To account for a 10% drop-out rate, 20 patients should be included into the study to have 18 patients available for statistical analyses.

4. MANUFACTURING OF THE PRODUCT

4.1 Product description and formulation

The active substance is autologous ex vivo expanded MSC. Autologous MSC are categorized as somatic cell therapy medicinal product, belonging to group of Advanced Therapy Medicinal Products (ATMP). The MSC investigational medicinal product is composed of a cryopreserved suspension of ex vivo expanded autologous human bone marrow-derived MSC, which is thawed immediately prior to administration to patients. The tissue is procured in accordance with EMA Tissues & Cells Directive, 2004/23/EC, as amended, and the product is produced in accordance with Good Manufacturing Practice (GMP) at the Centre of Cellular Therapy “G. Lanzani”, ASST Papa Giovanni XXIII, Bergamo and at the Officina Farmaceutica Ospedale Pediatrico Bambino Gesù, Roma. The manufacturing process for the MSC product is continuous. Cells derived from bone marrow explants are cultured and expanded in vitro. All plastic adherent cells are then collected and cryopreserved. All the steps of the manufacturing process are described in detail in the Investigational medicinal product dossier (IMPD) (sections 3.2.S).

4.2 Biological, physical, chemical and pharmaceutical properties of MSC

Mesenchymal stromal cells (MSC) are multipotent progenitor cells resident in the bone marrow and other tissues (15). MSC can be obtained from bone marrow cells after in vitro culture and expansion. Since their discovery, MSC have been shown to be able to differentiate into several mesenchymal tissues, as well as home to inflamed sites contributing to tissue repair and mediate potent immunomodulatory effects both in vitro and in vivo (31,32). Flow cytometry testing of MSC shows that the cells express epitopes consistent with a mesenchymal cell phenotype (30). Characterization data are provided in IMPD section 3.2.S.3.1, Elucidation of Structure and Other Characteristics.

Table 1: Properties of the drug substance according to the certificate of analysis:

Test	Specification
Viability	% viable cells ≥ 80%
Sterility EP 2.6.27	sterile
Mycoplasma EP 2.6.7	negative
Endotoxin EP 2.6.14	≤ 5 EU/mL
Immunophemotype:<br>$CD73^+$ percentage<br>$CD90^+$ percentage<br>$CD105^+$ percentage	≥ 90%
$CD14^+$ percentage<br>$CD34^+$ percentage<br>$CD45^+$ percentage	≤ 5
Colony assay in methilcellulose	Absence of clones
Karyology	No evidence of chromosomal aberrations

4.3 Drug product formulation

The investigational medicinal product consists of autologous MSC, cultured for a maximum of 2 passages and cryopreserved at a final concentration of 1 x 10^6 cells /ml in administration buffer consisting of human AB plasma and 10% clinical grade DMSO.

4.4 Excipients of MSC products

Table 2: List of excipients of MSC

Component	20 ml dose Amount per dose (ml)	50 ml dose Amount per dose (ml)
**Human AB plasma**	18	45
**Dimethylsulfoxide DMSO**	2	5
**MSC**	$20*10^6$ cells	$50*10^6$ cells

Human AB plasma: Human AB plasma is a medicinal product containing plasma proteins within the reference ranges for healthy blood donors (45-70 mg/mL). Out of a mean total protein content of 58 mg/mL, albumin accounts for 50% (29 mg/mL), whereas the immunoglobulin classes G, A, and M are present at levels of 8.1, 1.6, and 0.8 mg/mL, respectively.

The manufacturing process levels out inter-donor variations and maintain the plasma proteins in a functional state. When medicinal products prepared from human blood or plasma are administered, the possibility of transmitting infective agents cannot be totally excluded.

Standard measures to prevent infections resulting from the use of medical products prepared from human blood or plasma include selection of donors, screening of individual donations and plasma pool for specific markers of infection and the inclusion of effective manufacturing steps for the inactivation/removal of viruses and prions. Despite this, when medicinal products prepared from human blood or plasma are administered, the possibility of transmitting infective agents cannot be totally excluded. This also applies to unknown and emerging viruses and other pathogens.

To date, human AB plasma has not been reported to be associated with embryo-foetal toxicity, or oncogenic or mutagenic potential. Mild reactions such as flush, urticaria, fever and nausea may occur.

DMSO: DMSO is an active ingredient required for cryopreservation of cells and is registered as a medical device in the European Union. DMSO acts by penetrating the cell where it binds to water molecules in solution. This in turn blocks the efflux of water from the cytoplasm during freezing, preventing cellular dehydration or shrinkage and maintaining stable intracellular salt concentrations

and pH levels. By slowing the rate of freezing it also prevents the formation of harmful ice crystals within the cell. The LD50 values (amount of DMSO required to kill 50% of test animals) reported for intravenous infusion of DMSO are 3.1-9.2 g/kg for mice and 2.5 g/kg for dogs (51). The acute toxic dose for humans has not been determined. DMSO is not considered to be directly embryotoxic but may be teratogenic at high levels coinciding with the maximum tolerated dose (52). Use of DMSO as a cryo-protectant for cell therapy products has been considered standard of care since the introduction of cryopreserved autologous HSCT in the 1970's. Currently, ± 30.000 autologous stem cell grafts cryopreserved in DMSO are infused annually in both adult and pediatric patients (53). The most frequently used DMSO concentration is 10% v/v in combination with normal saline and human albumin. Based on the ample clinical experience with DMSO as a cryo-protectant for autologous stem cell grafts, DMSO is considered effective and safe in adults and children and considered a critical reagent for cryopreserved cellular therapy products. There is however a clinically significant side effect profile. Nausea, vomiting, and abdominal cramps have been reported in about half of the infusions. Also cardiovascular, respiratory, central nervous system including seizures, renal, hemolytic, and hepatotoxic presentations have been described, including rare fatalities (54-57). In a survey of 95 European Bone Marrow Transplantation (EBMT) centers, the mean incidence per centre of DMSO-associated toxicity other than nausea and vomiting was 2.1%. Between these centers, approximately 34000 transplants were carried out. 78 of the included centers used a final concentration of 10% v/v DMSO, the other centers used DMSO concentrations varying from 2.2% to 20%. There are no conclusive data on repeat administration of DMSO-containing cell products as this is not standard practice in the setting of HSCT. There are no published data on safety levels of DMSO for children. No dose-finding studies have been performed. However, DMSO infusion related toxicities do appear to be related to the amount of DMSO infused (58,59). Based on these experiences, most transplant centers now adhere to a maximum infusion rate of 1 ml DMSO per recipient kg body weight per 24 hours. The investigational medicinal MSC product contains 10% v/v DMSO; in the case of a dose of $30x10^6$ cells, this would correspond approximately to 30 ml investigational medicinal product and thus less than 0.1 ml DMSO per kg body weight. This dose is administered to both adults and children and is considered well within safety margins for both adults and children.

4.5 Storage and handling of the MSC product

All procedures are regulated and detailed in Standard Operating Procedures

Storage: The investigational medicinal MSC product is cryopreserved and stored in the vapor phase of liquid nitrogen. Stability data indicate that MSC is stable for at least 60 months when stored at the recommended storage condition of liquid nitrogen vapor phase. Following the thawing procedure the product should be brought to room temperature and infused as rapidly as possible (approximately 5-10 minutes).

Preparation: The cryopreserved investigational medicinal MSC must be thawed prior to administration. For some patients multiple bags may need to be thawed in sequence for a single administration. Body material and/or material that has come in contact with it should always be regarded as infectious and treated as such. To ensure sterility of the product, all procedures are performed in a Class A LAF cabinet in a class D environment, and products are inspected for container integrity.

Administration: For the purpose of the study two doses of investigational medicinal MSC product will be administered by intravenous infusion via an in-situ venous catheter of ≤ 22 gauge with a 7 day interval between the two doses. A dose of MSC consists of 1 x 10^6 MSC/ kg in a range of 0.75 x 10^6 MSC/ kg to 1.25 x 10^6 MSC/ kg (maximum 200 x10^6 MSC). Care should be taken to infuse the investigational medicinal product as soon as possible but maximum within 30 minutes from thawing and as rapidly as tolerated following the thawing procedure to minimize cell death, approximately in 5-10 minutes. The infusion rate should not exceed 1 mL of DMSO per kilogram of recipient weight per minute. Vital signs should be monitored prior to infusion, directly following infusion and then hourly for 2 hours. During infusion the patient should be monitored for signs of an infusion reaction.

5. PRE-CLINICAL STUDIES

5.1. Mechanism of action and pharmacodynamics of MSC: in vitro evidence

In this section, we will summarize the available data on the effects of MSC on cells of the immune system in vitro. More relevantly for the purpose of this study, we will show the results of a proof-

of-principle study that we performed on autologous MSC expanded from the bone marrow of children with severe forms of INS, comparable to those that we propose to treat in the current study.

5.1.1 Immunomodulatory activities of MSC

MSC have the ability to home to injured tissues and suppress immune responses, both in vitro and in vivo (32, 19, 33, 60-61). The capacity to regulate immune responses leading to regeneration of damaged tissues provides a strong rationale for the use of MSC as a new treatment modality, especially in diseases characterized by the presence of an immune dysregulation leading to tissue damage. Several in vitro studies demonstrated that MSC were able to inhibit T cell proliferation induced by allo-antigens, mitogens, CD3 and CD28 agonistic antibodies (20-22). A combination of in vitro co-culture studies, in vivo and animal studies demonstrated that, in addition to inhibiting the proliferation and activation of effector T cells, MSC can also induce the expansion of regulatory T cells and inhibit the expansion and activation of B cells and NK cells (62-64). MSC can furthermore modulate the innate immune response by inhibiting the differentiation and activation of myeloid-derived dendritic cells (65) and induce the skewing of monocytes and macrophages toward an anti-inflammatory phenotype (66-68). Together, these data indicate that MSC can suppress the activation of the immune system by regulating cells of the lymphoid immune and innate immune systems. These observations led to the notion that MSC could be used therapeutically to modulate immune responses. Low expression of surface MHC class I, II, antigen processing and co-stimulatory molecules (69) results in a low-stimulatory capacity of MSC in the interaction with other (activated) immune cells. The T cell inhibitory effect might partly be mediated via cell-cell contact (70), for instance through inhibitory molecules such as PD-L1 (71) and B7-H4 (72), Fas-mediated apoptosis (73) and surface expression of CD39 (74).

Trans-well experiments suggested that the T cell inhibition is further mediated via soluble factors such as transforming growth factor β (75), hepatocyte growth factor, HLA-G (76), prostaglandin E2 (19) and galectins (77). Immune-modulatory effects of MSC can be augmented when MSC are activated through IFN-γ (78-79) or through toll-like receptors (80). Activation can lead to the production of the T cell inhibitory enzyme IDO (indoleamine-2,3-dioxgenase) (80-85) which, through conversion of tryptophan into its metabolites kynurenine, inhibits the proliferation of immune cells,

such as NK cells, T and pro-inflammatory Th17 cells (86) and also induces regulatory T cells (62, 68, 70, 85). IDO can further induce anti-inflammatory M2 macrophages (66) (Figure 1).

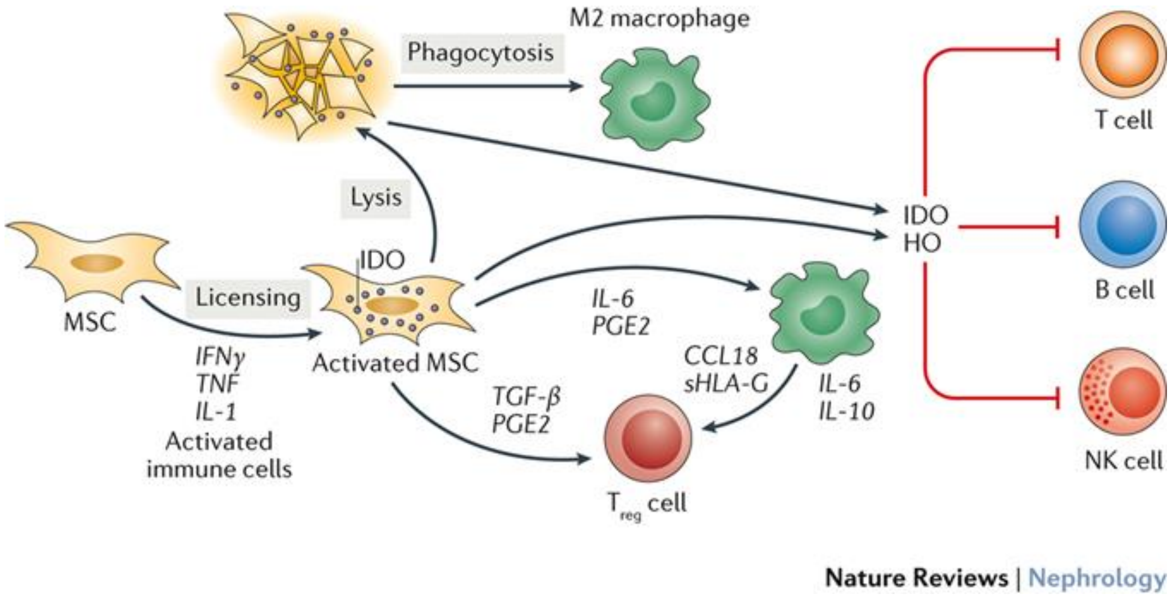

Figure 1: Putative immunomodulatory mechanisms of action of mesenchymal stromal cells (MSC)(87)

5.1.2 Properties of autologous MSC from patients with INS: in vitro evidence

For the purpose of this study, we have chosen to employ autologous bone-marrow derived MSC. The use of autologous MSC may have some advantages over that of allogeneic MSC, including the reduction of the risk of rejection, immunization and transmission of infectious agents, particularly in recipients that are not profoundly immunosuppressed. However, it is still debated whether the potency of autologous MSC derived from patients bearing an immune system dysregulation is comparable to that of healthy-donor (HD) MSC. Therefore, before employing patient-derived MSC in any clinical context, it is mandatory to investigate whether these cells are endowed with the same full regenerative, differentiation and immunomodulatory properties (88-89). For this purpose, we performed a proof-of-principle study by isolating and expanding ex-vivo bone-marrow derived MSC from 5 children with severe forms of INS, therefore presenting comparable clinical features to those of the patients that we propose to enroll in the present study. In this pilot study, we evaluated in vitro their phenotypic and functional characteristics, in view of their potential clinical application in immunomodulatory and regenerative cellular therapy approaches to the treatment of INS (1).

No differences in terms of recovery of MSC from BM aspirates of patients as compared with HD were found, suggesting that the underlying immunological disease does not seem to alter the frequency of mesenchymal progenitors in the BM of INS patients. INS-MSC biological and functional properties were compared with those of cells obtained from the BM of 8 HDs. Both INS- and HD-MSC were characterized by morphology, proliferative capacity, immunophenotype and differentiation potential. INS-MSC displayed the characteristic spindle-shaped morphology, similar to that shown by HD-MSC (Figure 2A). INS-MSC were immunophenotypically characterized by flow-cytometry at early passages, P2 or P3. Their immunophenotype was in agreement with previous publications (30) and comparable with that of HD-MSC. In particular, the hematopoietic markers (CD34, CD45, HLA-DR, CD14, CD19) were no longer detectable by P2 and P3 and almost all the cells expressed the surface marker pattern (CD73, CD13, CD105, CD90) typical of HD-MSC (Figure 2B).

In order to compare their proliferative capacity, INS- and HD-MSC were plated in parallel utilizing the same culture conditions; the number of PDs through at least 5 culture passages was calculated. INS- and HD-MSC showed a comparable proliferative capacity: the cumulative PDs from P1 to P5 for HD-MSC was 9.5 (SEM ± 1.9), whereas that of INS-MSC was 10 (SEM ± 2.3) [P=not significant (NS)] (Figure 2C).

All MSC were grown in culture until they reached a senescence phase. In particular, HD-MSC showed a decreased ability to proliferate in vitro between P8 and P15 (median P10) similarly to INS-MSC that ceased to grow between P7 and P15 (median P9) suggesting a similar in vitro life-span. Senescent state was confirmed in both cell types by the positivity for β-galactosidase staining (Figure 2D). Furthermore, in order to examine the differentiation ability of INS-MSC, and compare it with that of HD-MSC at different passages (from P2 until P8), cells were induced to differentiate in vitro into osteoblasts and adipocytes and then examined by histological staining. Both INS- and HD-MSC were able to differentiate into osteoblasts, as highlighted by the detection of calcium deposition after staining with Alizarin Red (Figure 2E) and by the histological demonstration of alkaline phosphatase activity (Figure 2F), as well as into adipocytes, as revealed by the formation of lipid droplets identified after Oil Red O staining (Figure 2G).

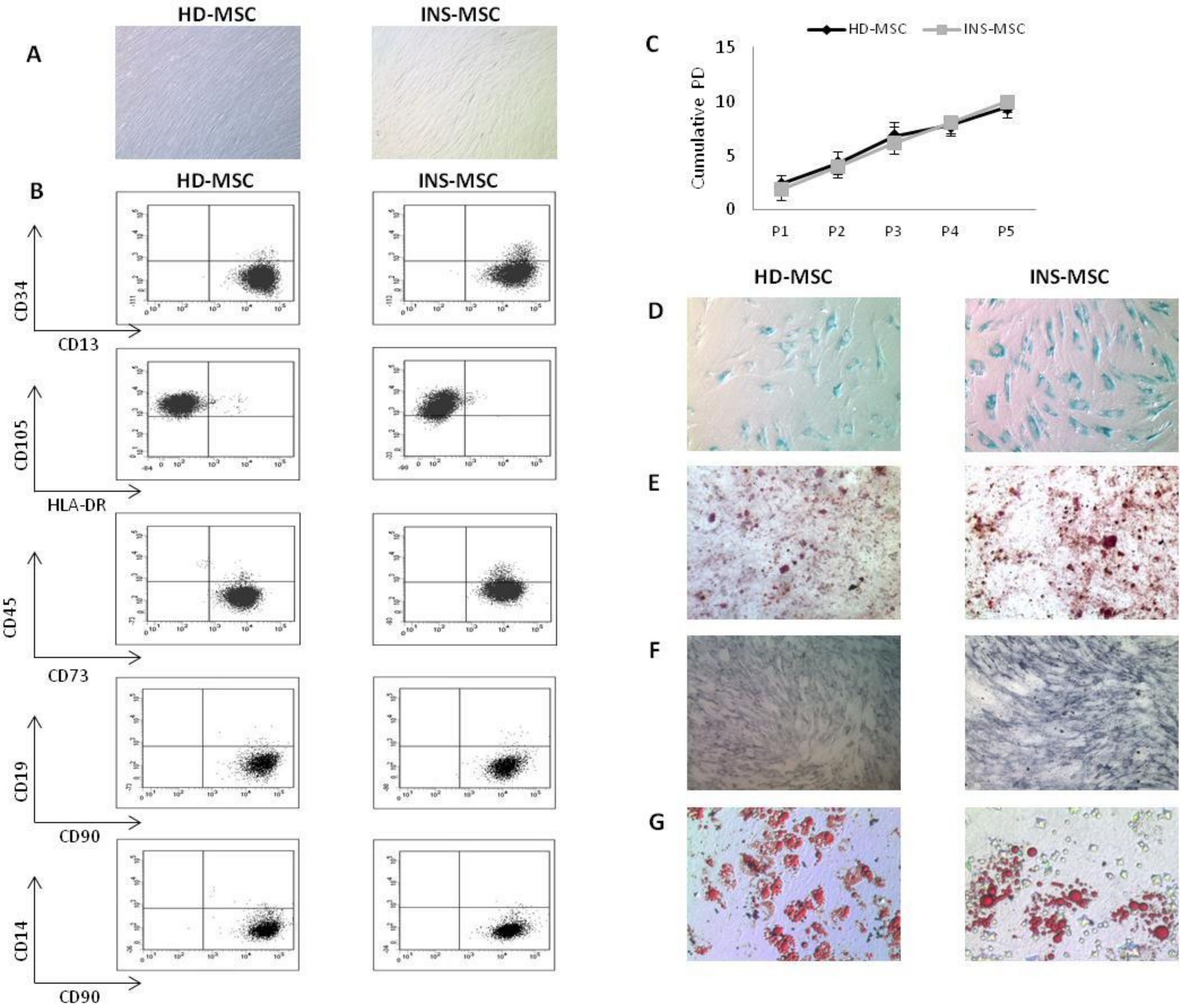

Figure 2: Phenotypic and differentiative features of autologous MSC expanded from children with INS compared to those expanded from healthy controls **(A).** Morphology of culture-expanded MSC obtained from a representative INS patient (INS-MSC) and from a healthy donor (HD-MSC). MSCs from both patient and donor display the characteristic spindle-shaped morphology (magnification x4). **(B)** FACS analysis of a representative MSC sample at passage (P) 2 from 1 HD and 1 INS patient. Histograms of surface marker expression are similar: positive for CD90, CD13, CD105 and CD73 surface antigens and negative for CD34, HLA-DR, CD45, CD14 and CD19 molecules. **(C)** Cumulative population doublings (PDs) from passage P1 to P5 of MSC isolated from HDs (black line) and from INS-patients (grey line). The data represent the mean (±SEM) of 5 INS-MSC and 8 HD-MSC. **(D)** Senescence was evaluated by β galactosidase staining. One representative example of HD-MSC at P13 and of INS-MSC at P12 is shown. (E-F-G) Multilineage differentiation potential was assessed by culturing MSC for 21 days with osteogenic and adipogenic medium and then staining the cells with Alizarin Red, Fast Blue and Oil red O. **(E)** The differentiation into osteoblasts is demonstrated by the histological detection of calcium depositions positive for Alizarin Red, magnification x20. **(F)** The differentiation into osteoblasts is demonstrated by the histological detection of alkaline phosphatase (AP) activity (purple reaction), magnification x20. **(G)** The differentiation into adipocytes is revealed by the formation of lipid droplets stained with Oil Red O, magnification x20.

MSC, mesenchymal stromal cell; HD, healthy donor; INS, Idiopathic nephrotic syndrome; PD, population doubling; ALP, alkaline phosphatase activity.

In order to evaluate the immunomodulatory capacity of *ex-vivo* expanded INS- and HD-MSC on T lymphocytes, PHA-induced PBMC proliferation was measured either in the presence or in the

absence of HD- or patient MSC in an allogeneic setting for all 5 INS patients and in an autologous setting for 1 INS-patient. In agreement with previously reported studies (31-32), HD-MSC exerted a strong and dose-dependent *in vitro* inhibitory effect on PHA-induced PBMC proliferation, with a mean percentage of proliferation in the presence of HD-MSC of 22% (SEM ±16.1; P<0.01, as compared with PBMCs+PHA alone) and 51% (SEM ±7.4; P<0.01, as compared with PBMCs+PHA alone) at MSC:PBMC ratio 1:2 and 1:10, respectively. With regard to INS-MSC, they were able to significantly prevent proliferation of allogeneic HD-PBMCs at a similar degree as compared with HD-MSC, with a mean percentage of proliferation of 31% (SEM ± 19.5; P<0.01, as compared with PBMCs+PHA alone) and 45% (SEM ± 22.5; P<0.05, as compared with PBMCs+PHA alone) at MSC:PBMC ratio 1:2 and 1:10, respectively (Figure 2A). No statistically significant differences were found between INS- and HD-MSC in terms of ability to influence PBMC proliferation after PHA stimulation in an allogeneic setting (P=NS at any MSC:PBMC ratio).

Moreover, when INS-MSC were co-cultured with autologous PBMCs, they were able to inhibit PHA-induced PBMC proliferation. In particular, in patient #1 (Pt.#1) INS-MSC co-cultured with autologous PBMCs exerted an inhibitory effect with a mean residual proliferation of 30% (SEM ±24.6; P<0.05 as compared with PBMCs+PHA alone) and 48% (SEM ±21.1; P<0.05 as compared with PBMCs+PHA alone) at MSC:PBMC ratio 1:2 and 1:10 respectively (Figure 2B). This appeared comparable to the inhibitory effect exerted by HD-MSC co-cultured with autologous PBMCs at the same ratios. Due to limited availability of PBMCs from INS patients and to the fact that, probably secondary to receiving several lines of IS, their proliferation in response to PHA was suboptimal, we were unable to test the immunoregulatory effect of INS-MSC in the autologous setting in a larger number of patients. These results indicate that MSC isolated from the BM of INS-patients maintain their *in vitro* anti-proliferative effect on PHA-induced proliferation of PBMCs isolated from HDs and also from 1 INS-patient.

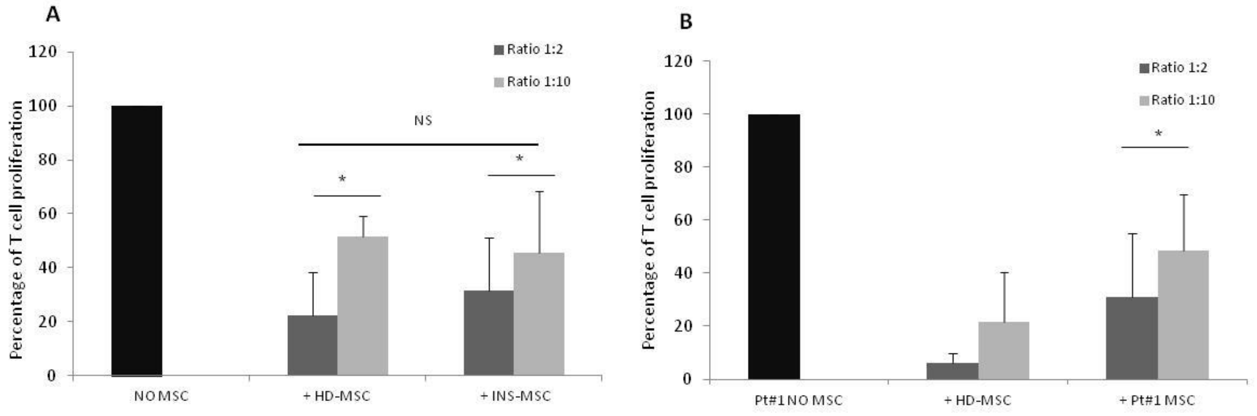

Figure 3 (A) *In vitro* immunomodulatory effect of HD- and INS-MSC on healthy donor peripheral blood mononuclear cells (PBMCs) in an allogeneic setting. The graph shows the percentage of residual proliferation of healthy donor PBMCs stimulated with phytohaemagglutinin (PHA) either in the absence or in the presence of HD- and INS-MSC. Each bar represents the percentage of residual proliferation of 10^5 PBMCs, in the presence of two different MSC:PBMC ratios (MSC:PBMC ratio of 1:2 and 1:10), calculated by measuring 3H-thymidine incorporation after 72 hour co-culture. Each bar represents the mean ± SEM of multiple experiments (each point being in triplicate) with MSC obtained from 5 INS-patients and 8 HDs. P values lower than 0.05 were considered to be statistically significant. **(B)** *In vitro* immunomodulatory effect of HD- and patient #1-MSC (Pt#1-MSC) on Pt#1 PBMCs in an autologous setting. The graph shows the percentage of residual proliferation of Pt#1-PBMCs stimulated with phytohaemagglutinin (PHA) either in the absence or in the presence of HD- and Pt#1-MSC.

With the aim to test the immunomodulatory effect of INS- and HD-MSC on B lymphocytes, MSC were co-cultured with allogeneic PBMCs stimulated or not with CpG and B-cell proliferation and plasma-cell (PC) differentiation were measured after 7 days. As previously shown (90), we found that INS-MSC were able to inhibit B-cell proliferation, with a frequency of proliferating CD19+ cells of 24.2% (SEM ± 14.9, P=0.09, as compared with PBMCs+CpG alone), as compared to 61.6% in the condition HD-PBMCs+CpG in the absence of MSC (SEM ± 14). INS-MSC were also able to inhibit the differentiation into PC with a percentage of PC of 16.3% (SEM ± 12.5; P=0.16, as compared with PBMCs+CpG alone) for INS-MSC, as compared to 40.7% in the condition HD-PBMCs+CpG in the absence of MSC (SEM ± 12). These results are comparable with those obtained with HD-MSC + PBMCs stimulated with CpG, where the frequency of proliferating B cells was 31.2% (SEM ± 30; P=0.19 as compared with PBMCs+CpG alone) and the percentage of PC was 19% (SEM ± 17; P=0.18 as compared with PBMCs+CpG alone) (Figure 3).

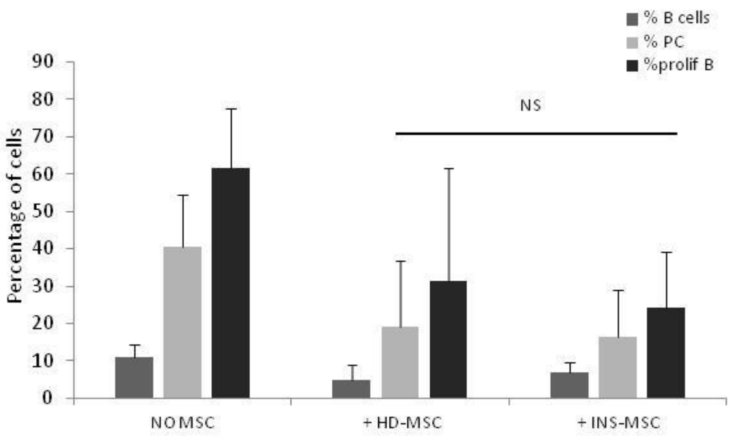

Figure 4. *In vitro* immunomodulatory effect of HD- and INS-MSC on B-cell proliferation and plasma-cell (PC) differentiation in an allogeneic setting. PBMCs were stimulated with CpG for 7 days and co-cultured with or without MSC. Cells were collected, stained for CMFDA, CD19, CD27, CD38 and IgM and analyzed by flow-cytometry. The graph shows the percentage of B cells, their residual proliferation and the percentage of PCs either in the absence or in the presence of HD- and INS-MSC (MSC:PBMC ratios 1:10). Each bar represents the mean ± SEM of multiple experiments (each point being in triplicate) with MSC obtained from 5 INS-patients and 8 HDs.

To validate further, both mechanistically and from a safety perspective, the therapeutic use of MSC derived from patients with INS, we exploited a model of podocyte injury to assess whether INS-MSC had any effect in this context. Adriamycin (ADR), a cytotoxic drug with a very wide spectrum of activity in human cancers, was used to induce the well-established rat (91, 92) and mouse (93-95) models of chronic proteinuric renal disease similar to human nephrotic syndrome. As in human nephrotic syndrome, the initial injury of rodent ADR-induced nephropathy (AN) is podocyte damage with fusion and effacement of foot processes, followed by glomerulosclerosis, tubulo-interstitial inflammation and fibrosis. The pathogenesis of AN has not been fully understood. Studies have showed that initial direct toxicity can cause podocyte death (96, 97), promotion of a senescence-like growth arrest (98) and can lead to fusion and effacement of podocyte foot processes (99). Lymphocytes and macrophages are important in renal injury progression in this disease model (100), which has allowed to probe mediators that may modulate or accelerate disease progression. Both immunological and non-immunological interventions, such as $CD8^+$ depletion (101), $CD4^+CD25^+$ T cell reconstitution (102), macrophage depletion (103) and, most relevantly, MSC (104-106) have been shown to play a protective effect on AN. This protective effect is confirmed by our *in vitro* experiments, in which MSC derived from INS patients and from HDs were assayed. As shown in Figure 4, co-culture of the *in vitro* filtration barrier damaged by ADR with INS-MSC and HD-MSC not only restored albumin permeability to its initial value (i.e. prior to ADR treatment), but also markedly

reduced it below the control values. These results may be ascribed to the role played by MSC in anti-apoptosis and prevention of podocyte loss (104) and to their ability to restore the normal actin cytoskeleton of podocytes with consequent improved cellular layer organization (99).

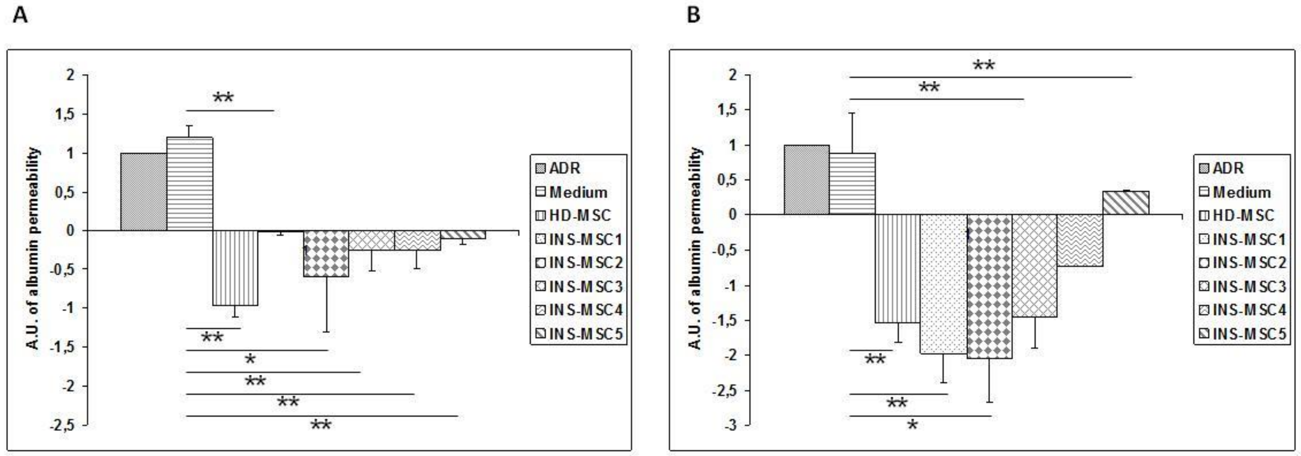

Figure 5 Effects of HD-MSC and INS-MSC (n=5) on albumin (BSA) permeability in podocyte-endothelial cell co-culture inserts simulating in vitro the glomerular filtration barrier. After podocyte damage induced by adriamycin (ADR), the BSA permeability increased. Compared with inserts positioned in the wells containing medium alone, exposure of the inserts to HD-MSC and INS-MSC after 24h **(A)** induced a significant decrease of albumin permeability, more evident after 48h exposure **(B).** The results were expressed as arbitrary unit of BSA permeability vs. ADR treatment (as 1). Each bar represents the mean ± SEM of triplicate experiments. * $P < 0.05$; A.U., arbitrary unit; ADR, adriamycin.

In conclusion, the present pilot study by Starc et al. provides safety and feasibility support for the therapeutic use of autologous BM-derived MSC in INS patients requiring prolonged exposure to more toxic immunosuppressive treatments. The choice of employing autologous MSC in the clinical setting has undeniable advantages in terms of safety, but requires a preliminary assessment of the quality and functions of these cells expanded from INS patients, to exclude that the disease itself and/or the immunosuppressive treatments administered may have an impact on the MSC compartment.

5.2 Mechanism of action and pharmacodynamics of MSC: animal models

In this section, we will discuss available data in animal models utilized to optimize the use of MSC in the context of kidney disease.

5.2.1 Adriamycin-induced nephrotic syndrome

Several rodent studies used the adriamycin-induced nephrotic injury as a model of chronic proteinuric renal disease similar to human nephrotic syndrome, to investigate the effects of MSC on disease progression and to elucidate potential mechanisms. Taken altogether, in these studies MSC have been shown to play a protective effect on ADR-induced nephropathy (AN) (104-106). As in human nephrotic syndrome, the initial injury of rodent AN is podocyte damage with fusion and effacement of foot processes, followed by glomerulosclerosis, tubulo-interstitial inflammation and fibrosis (95). Of note, this model is far from perfect, as the injury is secondary to a toxic insult, therefore not a primarily immune-mediated process. However, lymphocytes and macrophages are important in renal injury progression in this disease model (100-103), which has allowed to probe mediators that may modulate or accelerate disease progression. Zoja et al. (105) demonstrated that the early podocyte injury and subsequent chaotic migration of parietal epithelial progenitor cells which pave the way to crescents-like lesions and glomerulosclerosis was improved by treatment with MSC. Repeated MSC injections, by exerting a remarkable antiapoptotic effect, limited podocyte depletion and partially restored nephrin and CD2AP expression. This therapeutic approach created a glomerular pro-regenerative environment possibly by enhancing glomerular levels of VEGF, a factor able to activate Akt, a kinase upstream target of antiapoptotic and prosurvival pathways in podocytes. The restoration of podocyte number and function could limit migration and proliferation of parietal progenitor epithelial cells (PEC) of the Bowman's capsule thereby reducing the early formation of PEC-podocyte bridges. These renoprotective effects were also accomplished by anti-inflammatory effects of MSC therapy, with markedly reduced glomerular macrophage infiltration and local release of chemoattractants possibly involved in PEC-podocyte activation. However, in this model the authors failed to detect a significant reduction of proteinuria in the animals treated with MSC. Moreover, another study on a rat model of AN (106), demonstrated that MSC infusion could efficiently prevent or reverse podocyte structural damage induced by Adriamycin. Several mechanisms for the beneficial effects of MSC on injured kidney were proposed: immunosuppressive and anti-inflammatory actions and anti-fibrotic, anti-apoptotic and proangiogenic effects. This data suggested that MSC protect kidney tissue from damage in multiple ways. First, MSC inhibit podocyte apoptosis and promote cell repair by enhancing p21 expression. Second, MSC protect glomeruli from doxorubicin induced damage through maintaining the structural integrity of the podocyte cytoskeleton protein synaptopodin. Third, MSC inhibited the secretion of inflammatory promoter MCP-1, thereby reducing the inflammatory injury of kidney tissue. MSC improved the renal function

and slowed the disease progression in this rat model of nephrotic syndrome. These results were recently confirmed also in a study assessing the effect of MSC derived from induced pluripotent stem cells (iPS) reprogrammed from human fibroblasts via viral transduction and administered iv to NOD/SCID mice with or without adriamycin nephropathy. This treatment had a beneficial effect on renal function loss by limiting tubular apoptosis and promoting tubular proliferation, attenuating oxidative stress and reducing fibrosis (107).

5.2.2 Kidney transplant

As recently reviewed (108), MSC are emerging as a promising and innovative cell therapy in organ transplantation, such as in kidney transplantation, the therapy of choice for end-stage renal disease (ESRD) (109-111). The rationale for this approach is that administration of cells with immunoregulatory properties to transplant recipients could tip the balance between effector and regulatory pathways, ultimately promoting the potential of the host immune system to control the immune response to the allograft (112). Some studies have assessed the immunomodulatory properties of MSC in kidney transplant models. In mice, post-transplant infusion of donor BM-derived MSC induced donor-specific tolerance toward life-supporting, fully allogeneic kidney transplants, by generating immature DCs and regulatory Tcells (113).

MSC from a third-party source have also been shown to confer long-term protection of kidney allografts. In Lewis rat recipients of Fisher344 rat kidneys, infusion of third-party MSC at 11 weeks after transplantation inhibited intragraft T-cell and macrophage infiltration and prevented the development of interstitial fibrosis and tubular atrophy (114). Notably, in this model MSCs also limited the activation of the humoral immune response, as indicated by lower levels of anti-donor MHC antibodies in MSC-treated rats than in untreated controls.

In contrast to these positive studies, multiple peri transplant infusions of syngeneic MSC have failed to prolong rat kidney graft survival, although they significantly inhibited intragraft macrophage and DC infiltration in response to ischaemia–reperfusion injury (115). Rat kidney recipients that received syngeneic MSC pre-transplantation in combination with low-dose mycophenolate mofetil (MMF) developed graft dysfunction 7 days post-transplantation, which was associated with histological evidence of tissue damage, increased expression of proinflammatory cytokines and increased B-cell infiltration and C4d deposition in the graft (116). Similarly, other studies in rats have shown that peritransplant injection of syngeneic BM-derived MSC results in severe renal insufficiency within 15

days after allogeneic kidney transplantation. Histologic analysis of kidney grafts from MSC-treated rats showed granulocyte infiltration and signs of thrombotic microangiopathy, despite decreased graft T-cell infiltration (117). The conflicting findings with donor and syngeneic BM-derived MSC with or without MMF therapy are difficult to interpret and reconcile; however, they suggest that MSC could acquire *in vivo* pro-tolerogenic or proinflammatory function, at least in experimental kidney transplant models.

Several factors might contribute to dictating the tolerogenic or inflammatory properties of MSCs. As comparable doses of cells have been used in different experimental settings, the timing of cell infusion in relation to kidney transplantation could have a major role. In a murine kidney transplant model, intravenous infusion of syngeneic MSC 1 day before transplantation induced indefinite graft survival (118). This effect occurred even when MSC were administered to recipient mice that had previously been sensitized with an infusion of donor spleen cells, and thus carried donor-specific memory T cells. Tolerance induction was associated with regulatory T cell expansion within the recipient lymphoid organs in which the syngeneic MSC localized. By contrast, MSC that were given 2 days after transplantation migrated into the transplanted kidney. Similarly other investigators have shown that in rodents the majority of intravenously infused MSC localize immediately to the lungs, with a small proportion gradually migrating to other tissues such as the liver, spleen and kidney, and to the site of injuries and tumours within hours of the infusion (119). Notably, the level of engraftment of MSC into the kidney increases following ischaemia–reperfusion injury caused by kidney transplantation (120) or renal pedicle clamping (121). MSC engrafted in the injured kidney promote neutrophil infiltration, complement activation and expression of proinflammatory cytokines, leading to impairment of graft function (118). The mechanism by which intragraft MSC can be activated to a proinflammatory phenotype remains ill-defined. The possibility exists that MSC might be exposed to several inflammatory mediators induced by renal ischemia–reperfusion injury that ultimately direct them to acquire proinflammatory functions. Further studies are needed to identify and characterize the molecular mechanisms that underlie the pro-tolerogenic and proinflammatory phenotypes of MSCs, in order to optimize the conditions of MSC administration and fully exploit the tolerogenic potential of this cell therapy in kidney transplantation. MSC therapy undoubtedly has potential in kidney transplantation. Preclinical and early clinical results seem promising, but moving the concept of MSC-based therapy forward to large-scale clinical application should be assessed critically.

5.2.3 Repair of kidney damage

A vast body of experimental evidence has been accumulating on the therapeutic potential of MSC in the repair of renal injury, preserving renal function and prolonging animal survival, both in models of acute kidney injury (AKI) and in models of different forms of chronic kidney disease, primarily in diabetic nephropathy (reviewed in 122). These beneficial effects are exerted both through the homing of MSC to the site of injury and the release of growth factors and mediators with anti-inflammatory, anti-fibrotic and immunomodulatory effects and via the release by MSC of microvesicles, able to deliver genetic and proteic material with anti-inflammatory and pro-regenerative properties to injured renal cells.

A recent study performed in a porcine model of metabolic syndrome and unilateral renal artery stenosis, combining hypoxic and inflammatory glomerular and tubular damage, assessed the therapeutic use of extracellular vesicles derived from autologous adipose tissue MSC injected intrarenally. This approach led to i) a reduction in inflammation assessed by a reduction in the number of infiltrating inflammatory M1 macrophages and by decreased renal artery levels of MCP-1, IL-6, TNF-α, IL1-β and increased levels of IL-10 and IL-4, ii) a reduction in medullary hypoxia, assessed by magnetic resonance imaging, iii) an improvement in serum creatinine with a reduction in tubule-interstitial fibrosis and glomerular damage assessed by kidney biopsy (123).

Multiple studies in animal models (reviewed in 124) suggest that MSC may suppress multiple pathogenic pathways and promote regenerative mechanisms by secretion of paracrine anti-inflammatory and cyto-protective factors, leading to reduction of progressive damage in models of renal chronic damage secondary to diabetic nephropathy. However, only a few early phase clinical trials have been started and the real efficacy and optimal approach of this therapeutic strategy remains to be proven.

Taken altogether, studies in animal models of different diseases affecting kidney function suggest that MSC may be of therapeutic value in renal diseases. The beneficial effects of MSCs appear to be exerted in two ways: 1) systemically, by immunomodulatory, i.e. reduction of effector T cell function, inhibition of B cell proliferation, differentiation and immunoglobulin production and anti-inflammatory, i.e. through a decrease in M1 macrophage homing and function, effects and 2) locally

by reducing intrarenal inflammatory cell infiltration, preventing or reversing renal fibrosis, stimulating tubular repair and regeneration and promoting tubular cell survival by reducing apoptosis and oxidative stress (125).

5.3 Pharmacokinetics and Immunogenicity of allogeneic MSC: animal studies

Whereas the majority of *in vitro* studies considered MSC to be immune-privileged, being unable to induce allo-responses (126), recent animal studies revealed a low level of immunogenicity (127-128). Allogeneic murine BM-MSC for instance were rejected in MHC-mismatched murine recipients (129) and under certain conditions activated MSC could act as antigen-presenting cells (130,131), thus potentially inducing immune responses. For instance, baboons produced allo-antibodies after repetitive immunization of 5 $x10^6$ allogeneic BM-MSC/kg (132). The biological consequences of such anti-donor immune responses in animal models are not evident and there is substantial variability with regards to experimental species, disease model, route of MSC administration, cell dose and stringency of the immunological assays employed (Table 3).

Table 3: Allogeneic MSC survival time in different animal models

Article	Lung Trap	Method of detection	Site of inflammation	Recipient	Allogeneic donor	Detection time
Schrepfer et al. 2007	Yes	Luciferase/CFSE, bioluminescence + FACS	None	mice	mice	Up to 30 min
Eggenhofer et al. 2012	Yes	DsRed/ Cr-51 label, FACS/histology/ gamma counter	None	mice	mice	Up to 72 hours
Fischer et al. 2009	Yes	Qtracker FACS, infrared imaging	None	rats	rats	Up to 10 min
Gao et al. 2001	Yes	^{111}In-Oxine Gamma imaging	None	rats	rats	Up to 48 hours
Assis et al. 2010	Yes	99mTc/DAPI, gamma counter, Histology	Infarcted myocard	rats	rats	5 min to 7 days
Kraitchman et al. 2005	Yes	^{111}In-Oxine, CT	Infarcted myocard	canine	canine	1-7 days
Sackstein et al. 2008	Yes	Tracer dye, Imaging	None	mice	human	1-24 hour
Lee et al. 2009	Yes	Human Alu sequences PCR	Infarcted myocard	mice	human	0-100 hours
Kidd et al. 2009	Yes	Luciferase, bioluminescence, histology	Wound	mice	human	1-10 days

Sasaki et al. 2008	No	GFP transgene, histology/FACS	Biopsy skin wounds	mice	mice	Up to 12 weeks
Jin et al. 2012	?	PKH26 dye, histology	Hepatic injury	mice	mice	4 weeks
Sullivan et al. 2013	?	GFP, PCR	Inflamed joints	mice	mice	5-14 days
McFarlin et al. 2006	No	Iron oxide + DiI dye, Histology	Incision wounds	rats	rats	6 days
Assis et al. 2010	Yes	99mTc/DAPI, gamma counter, histology	Infarcted myocard	rats	rats	5 min to 7 days
Jackson et al. 2010	No	Iron oxide + FITC, MRI, histology	Inflammatory brain injury	rats	rats	up to 10 days
Wu et al. 2008	No	GFP transgene, histology	Brain ischemia	rats	rats	Day 7
Mouiseddine et al. 2007	?	Human bGlobulin PCR, aHuman histology	Radiation injury sites	mice	human	15 days
Mahmood et al. 2003	No	aHuman histology	Inflammatory brain injury	rats	human	28 days
Kidd et al. 2009	Yes	Luciferase, bioluminescence, histology	Wound	mice	human	1-10 days
Devine et al. 2003	Yes	GFP, PCR	None, still migration to wide range of tissues	baboon	baboon	9-21 months

These studies provided valuable information on the mechanisms through which the immune system responds to MHC-mismatched MSC. Anti-donor immune responses generally result in reduced in vivo survival of allogeneic-MSC mediated by rejection and/or the promotion of donor-specific tolerance (133). Nevertheless, no anti-allogeneic HLA antibodies were detected in a clinical trial of 12 aGvHD patients receiving allogeneic MSC infusion (134). To date, measurements of anti-donor antibodies in clinical trials are rare and considerable care must be taken in drawing conclusion on the in vivo immunogenicity from pre-clinical studies (135).

5.4 The choice of allogeneic versus autologous MSC

The criteria that regulate the choice between autologous and allogeneic MSC remain poorly defined and the results published so far do not allow definitive conclusions. Allogeneic MSC from third party donors represent a ready-to-use, “off-the-shelf” product. This approach has the advantage of immediate availability and high homogeneity. However, the risk of immune recognition and clearing

of allogeneic MSC by the immune system of non-profoundly immunosuppressed subjects, such as those affected by INS, should be taken into account (135). Recent evidence highlights that this aspect may be more relevant that what was previously thought (136). For this reason we have chosen a so-called "patient designated" approach with autologous bone marrow-derived cells. In the context of a chronic disease like INS, which offers sufficient time for MSC *ex vivo* expansion, autologous cells can be employed to overcome the risk of immune rejection, provided that they are functionally active. The choice of employing autologous MSCs in the clinical setting of INS has undeniable advantages, but requires a preliminary assessment of the quality and functions of these cells expanded from INS patients, to exclude that the disease itself and/or the immunosuppressive treatments administered may have an impact on the MSC compartment. Indeed, for some diseases it has been debated whether MSC from patients maintain the full spectrum of regenerative and immunomodulatory properties of MSC from third party healthy donors. To confirm that the proposed source of cellular product for our study, i.e. autologous MSC expanded from the bone marrow of children with severe forms of INS, maintain the properties of MSC from third-party healthy donors, we performed an *in vitro* proof-of-principle study, whose results are detailed in section 5.1.2 of this Investigator Brochure. Briefly, we verified that MSC expanded from the BM of 5 children with INS who present a very similar clinical picture to those that we propose to enroll in this study, in terms of disease severity and of burden of immunosuppression, maintain differentiative and immunomodulatory properties comparable to those of MSC derived from healthy controls. Moreover, supernatants from these MSC were able to ameliorate podocyte damage induced by adriamycin, in a classic in vitro model of glomerular filtration barrier injury. These results provide encouraging preliminary data for the in vivo use of autologous MSC expanded from patients with difficult INS in a clinical setting (1), and confirm findings in the context of renal transplantation showing that therapy with prednisone, cyclosporine A, MMF do not hinder MSC immunomodulatory properties in vitro (137).

5.5 Conclusions from pre-clinical studies

The results presented above, taken altogether, suggest:

1) that MSC appear both *in vitro* and *in vivo* in animal studies to present immunomodulatory and regenerative potential that could theoretically be beneficial in the treatment of idiopathic nephrotic syndrome

2) that, in the setting of this study with largely immunocompetent children, the use of allogeneic MSC could potentially be hindered by immunogenicity

3) that autologous MSC expanded from patients with INS maintain *in vitro* a proliferative, differentiative and immunomodulatory potential comparable to those expanded from healthy controls, and appear beneficial in an in vitro model of glomerular filtration barrier damage.

In this context, the use of autologous MSC may represent a safe therapeutic option for the treatment of severe cases of idiopathic nephrotic syndrome, allowing significant reduction of disease burden by maintaining patients in remission with less concomitant immunosuppression.

6. EFFECTS IN HUMANS- CLINICAL STUDIES

6.1 Overview of safety and efficacy

MSC have been employed therapeutically since the 1990s and the extensive, albeit very heterogenous literature available, describing mostly very small number of patients per study, collectively reaches more than 3000 patients reported. A number of recent meta-analysis studies (138-141) comparing and pooling results from the best available trials for specific indications (i.e. liver cirrhosis, pulmonary fibrosis, myocardial infarction, diabetes mellitus), in addition to a rigorous comprehensive meta-analysis including all indications and available randomized controlled trials (142) are published. All failed to detect associations between intravenous infusion of MSC and severe adverse effects, such as acute infusional toxicity, organ system complications, infections, death or malignancy. Transient fever upon infusion was reported (142). The “G. Lanzani” Cell Factory in Bergamo has treated over 100 patients with MSC expanded under conditions identical to those that will be employed in this study and no major adverse events have been observed (143).

MSC are multipotent cells that can be isolated from several human tissues, including bone marrow (BM), and consistently expanded *ex vivo* for clinical use (31). MSC are endowed with unique immunomodulatory and anti-inflammatory properties directed towards cells involved in both the adaptive and innate immune responses (32, 62-65). Moreover, they are able to home to sites of injury and promote tissue repair (144-154). Because of these properties, their therapeutic use has been evaluated in a number of clinical settings.

In 2012 a large systematic review and meta-analysis was published including 36 prospective clinical trials in both adults and children with a total of 1012 participants (142). Clinical conditions under study included ischemic stroke, acute and chronic kidney disease, Crohn's disease, cardiomyopathy, myocardial infarction, aGvHD, and healthy volunteers. 289 of the patients described in this meta-analysis received bone marrow derived MSC from allogeneic healthy donors. Due to limited data on the MSC product used in these studies, it is not known which patients received fresh or cryopreserved (DMSO containing) MSC. However, a meta-analysis of 6 randomized controlled trials (RCTs) included in this review revealed no significant differences in acute infusion toxicity (Odds ratio (OR) 2.12, 95% confidence interval (CI) 0.55-8.77) other than an increase in body temperature (OR 16.82, 95% CI 5.33-53.10). As to organ system related events, no significant differences were found for arrhythmias (OR 0.33, 95% CI 0.10-1.04), pooled gastro-intestinal and renal events (OR 1.15, 95% CI 0.38-3.50), and infection-related events (OR 1.08, 95% CI 0.48-2.38). Pulmonary events were only reported in one RCT in which MSC treatment was associated with a significant improvement in pulmonary function. Similarly, neurologic events were only reported in one RCT in 3/16 (19%) MSC treated patients versus 5/16 (14%) control participants. No association between the long-term events death and malignancy was detected. No MSC-related SAE was documented. The maximum follow up was 5 years, but most studied incorporated in the meta-analysis had a short follow-up. Other factors hindering extrapolation are the use of different MSC types (both autologous and allogeneic) and the different clinical conditions under study as well as differences in dosage, dose interval, etc. Because the patient's condition (inflammatory milieu) may affect the activation status and thus the safety profile of MSC post-infusion, long-term follow up would be necessary for all the different conditions studied.

6.2 Immunomodulatory activities of MSC: evidence from clinical studies

Currently there are no techniques available to measure the immunological efficacy of MSC administration in patients. At present, the effects of MSC in humans are measured almost exclusively by means of clinical outcomes. As mentioned above, the therapeutic use of MSC has been evaluated in a number of clinical settings and in a variety of allo- and auto-immune disorders (reviewed in 47), for example to treat steroid-resistant acute graft-versus-host disease in the context of hematopoietic stem cell transplantation (HSCT) (33, 143), to repair inflamed fistulas in

Crohn's disease (39-41), to prevent chronic rejection in solid organ transplantation (reviewed in 36), including relapse of nephrotic syndrome following renal transplantation (38). Moreover, many studies demonstrate that the use of autologous MSC obtained from patient's tissues can be a feasible, safe and beneficial therapy in several diseases, including renal glomerulopathies (40-42, 49, 50, 35, 125).

6.3 Safety and Efficacy of MSC infusion for kidney disease

MSC treatment has been evaluated for a large number of renal conditions (reviewed in 35, 125), including for the treatment of patients with acute kidney injury, renovascular disease (49), diabetic nephropathy (124), polycystic kidney disease (50), senescence in chronic kidney disease and, more relevantly to the current therapeutic proposal, lupus nephritis (34, reviewed in 87), graft survival in renal transplantation (reviewed in 36), focal segmental glomerulosclerosis (37) and in one atypical case of nephrotic syndrome (38). Currently, 21 ongoing or recently completed phase I and II clinical trials have been uploaded in the NIH database (https://www.clinicaltrials.gov/ct2/results/details?term=mesenchymal&cond=Renal+Disease). These studies (see Table 4) aim to test mainly the safety of using MSC and their efficacy in treating CKD (3 studies), diabetic nephropathy (2 studies), different forms of acute kidney injury (4 studies), renal transplantation (6 studies), lupus nephritis (3 studies), polycystic kidney disease (1 study), FSGS (1 study), arteriovenous fistula in CKD (1 study). Four of them propose the use of autologous BM-MSC (NCT02166489, NCT02195323, NCT01840540 and NCT00659217), three adipose-tissue derived MSC (NCT02266394, NCT03321942and NCT02808208), one umbilical cord-derived MSC (NCT01539902), the others allogeneic MSC. These explorative studies are either ongoing or only just completed, and few preliminary result has been provided so far. As described above, MSC infusion appears to be a safe treatment that is associated with no systemic toxicity. However, there is a specific set of safety concerns that are associated with cell therapies in general and are especially relevant in immunocompromised patient populations.

Table 4 Human trials of MSC for kidney disease registered in ClinicalTrials.gov at February 21, 2018

NUMBER	TITLE	LOCATION	STATUS	PHASE
NCT03321942	Treatment of Chronic Renal Failure With Adipose Tissue-derived Mesenchymal Stem Cells	China	Recruiting	-
NCT03174587	Evaluate the Safety of CS20AT04 Inj. in Subjects With Lupus Nephritis	Republic of Korea	Enrolling by invitation	Phase 1
NCT03015623	A Study of Cell Therapy for Subjects With Acute Kidney Injury Who Are Receiving Continuous Renal Replacement Therapy	United States	Recruiting	Phase 1-2
NCT02966717	Rituximab Combined With MSCs in the Treatment of PNS (3-4 Stage of CKD)	China	Active, not recruiting	Phase 2
NCT02808208	Autologous Adipose Derived Mesenchymal Stem Cells (AMSC) in Reducing Hemodialysis Arteriovenous Fistula Failure	United States	Recruiting	Phase 1
NCT02585622	NEPHSTROM for Diabetic Kidney Disease	Ireland<br>Italy<br>United Kingdom	Not yet recruiting	Phase 1 - 2
NCT02563366	Effect of BM-MSCs on Early Graft Function Recovery After DCD Kidney Transplant.	China	Unknown	Phase 1 - 2
NCT02561767	Effect of BM-MSCs in DCD Kidney Transplantation	China	Unknown	Phase 1 - 2
NCT02382874	Allogenic AD-MSC Transplantation in Idiopathic Nephrotic Syndrome (Focal Segmental Glomerulosclerosis)	Islamic Republic of Iran	Unknown	Phase 1

NCT02266394	Hypoxia and Inflammatory Injury in Human Renovascular Hypertension	United States	Recruiting	Phase 1
NCT02195323	Autologous Bone Marrow Derived Mesenchymal Stromal Cells (BM-MSCs) in Patients With Chronic Kidney Disease (CKD)	Royan Institute, Tehran, Iran, Islamic Republic of	Completed	Phase 1
NCT02166489	Mesenchymal Stem Cells Transplantation in Patients With Chronic Renal Failure Due to Polycystic Kidney Disease	Islamic Republic of Iran	Completed	Phase 1
NCT01843387	Safety and Efficacy of Mesenchymal Precursor Cells in Diabetic Nephropathy	Australia	Completed	Phase 1 - 2
NCT01840540	MSC for Occlusive Disease of the Kidney	United States	Completed	Phase 1
NCT01602328	A Study to Evaluate the Safety and Efficacy of AC607 for the Treatment of Kidney Injury in Cardiac Surgery Subjects	United States<br>Canada	Terminated	Phase 2
NCT01539902	Phase 2 Study of Human Umbilical Cord Derived Mesenchymal Stem Cell for the Treatment of Lupus Nephritis	China	Unknown	Phase 2
NCT01429038	Mesenchymal Stem Cells After Renal or Liver Transplantation	Belgium	Unknown	Phase 1 - 2
NCT01275612	Mesenchymal Stem Cells In Cisplatin-Induced Acute Renal Failure In Patients With Solid Organ Cancers	Italy	Recruiting	Phase 1
NCT00659620	Mesenchymal Stem Cell Transplantation in the Treatment of Chronic Allograft Nephropathy	China	Unknown	Phase 1 - 2

NCT00659217	Effect of Mesenchymal Stem Cell Transplantation for Lupus Nephritis	China	Unknown	Phase 1 - 2

6.3.1 Results of the therapeutic use of MSC in lupus nephritis

The most relevant study to date is the recently published randomized, double-blind, placebo-controlled trial of allogeneic umbilical cord-derived MSC performed on 18 patients with class III or class IV lupus nephritis, all followed in one centre in China. Participants were randomly assigned at a ratio of 2 to 1 to receive either two weekly intravenous injections of human umbilical cord-derived MSC or placebo. In addition, both groups received immunosuppression (intravenous methylprednisolone and cyclophosphamide, followed by maintenance therapy with oral prednisolone and mycophenolate mofetil). A similar proportion of patients in each group achieved complete remission. Therefore, the study failed to prove a beneficial effect of the therapeutic use of MSC in this context, showing however overall excellent outcome for all patients and no adverse effects (34). Before this, a total of 4 studies evaluating a total of 152 patients, using allogeneic MSC derived from BM or from umbilical cord, had been performed. As reviewed by Tyndall (47), these studies showed promising results in terms of improvement of scores of activity, reduction of proteinuria and of serological markers. No major adverse effects that could be ascribed to the MSC infusion were reported (87).

6.3.2 Results of the therapeutic use of MSC in focal segmental glomerulosclerosis

A single pediatric case describing the use of allogeneic bone marrow-derived MSC in the setting of focal segmental glomerulosclerosis recurring on a renal graft immediately following transplantation is reported (37). The patient was treated with prednisone, calcineurin inhibitors, mycophenolate mofetil and rituximab, but obtained stabilization of his proteinuria only with intensive plasmapheresis, as frequently observed in these cases. Three infusions of MSC were administered and plasmapheresis was tapered and stopped. The MSC infusions were well tolerated with no adverse reactions, and a sustained stabilization of proteinuria and of renal function despite interruption of plasmapheresis was obtained, improving the patient's quality of life. Prolonged

reduction of a panel of inflammatory markers was observed, with no adverse events reported at 22 months of follow-up.

6.3.3 Results of the use of MSC in nephrotic syndrome post-allogeneic hematopoietic stem cell transplant

A single case describing the use of allogeneic bone marrow-derived MSC for the treatment of nephrotic syndrome in a 31-year old woman, 10 months following an allogeneic hematopoietic stem cell transplant from a sibling for an hematological malignancy is reported. The nephrotic syndrome proved unresponsive to treatment with prednisone, cyclosporine A and rituximab, therefore the patient underwent treatment with MSC that proved safe and well tolerated. Two weeks after the first MSC infusion, therefore 6 weeks after the treatment with rituximab, proteinuria decreased substantially. Renal function remained normal, and increased T regulatory and B regulatory populations were observed (38).

6.3.4 Results on the use of MSC in renal transplantation

In the setting of solid organ, in particular renal, transplantation, the therapeutic use of MSC has been assessed with the objective of modulating T cell memory and promoting the development of T regulatory cells, thus achieving a beneficial balance between graft rejection avoidance and a less toxic immunosuppressive regimen (36). To this purpose, a step-by-step approach has been employed to determine optimal timing of infusions and concomitant immunosuppressive treatment. Autologous MSC have been largely preferred in this setting, to minimize sensitization and maximize efficacy in the context of partially immunocompromised patients.

The results obtained in these studies are of particular relevance to our proposed study for two reasons:

1) Because the treatment regimen employed to prevent graft rejection entails the use of prolonged immunosuppression with prednisone, mycophenolate mofetil and a calcineurin inhibitor which is very similar to that employed to maintain remission in severe idiopathic nephrotic syndrome patients. Therefore the ultimate objective of these studies, to achieve a reduction of a toxic immunosuppressive regimen, is common to our objective in treating INS patients

2) Because these studies have been performed using autologous MSC at very similar timing and doses as those that we propose, and more precisely 2 of these studies report patients treated with MSC prepared in one of the 2 Cell Factories that this study will employ, with a preparation protocol identical to the one that will be employed in this study.

In total 157 patients have been treated to date. Severe infusion reactions or side effects are not described, and in some cases reduced immunosuppression was achieved without graft rejection (reviewed in 36, 108). It is important to note that in one of the first published studies (137) the MSC infusion 7 days after kidney transplantation was accompanied by a temporary early graft dysfunction in two patients. This led to a modification of the timing of the infusions (before transplantation), with no repetition of this observation (155).

These findings support a role of the use of autologous MSC for immunosuppressive drug minimization, calcineurin inhibitor withdrawal, prevention of rejection and reduction of chronic transplant inflammation and fibrosis. The evaluation of the immunomodulatory properties of this approach in vivo has confirmed that an expansion of peripheral T regulatory cells and a functional modulation of memory T cells occurs (118, 137). Taken altogether, these results suggest that autologous MSC might be beneficial also in difficult forms of immune-mediated nephrotic syndrome.

6.4 Pharmacodynamic interactions

Since MSC are a cellular therapy product, by their own nature their mode of action is through direct cell-cell interaction, release of soluble factors and microvesicles, as detailed above. Interestingly, in vitro data on cyclosporine (156), our ex-vivo data on patients with severe forms of nephrotic syndrome taking cyclosporine, mycophenolate mofetil or both (1), and the results of human studies on subjects undergoing renal transplantation detailed above, all concur in suggesting that these drugs, which most patients enrolled in the present study will be taking, do not interfere or possibly enhance the immunomodulatory effects of human MSC.

6.5 Conclusions from clinical studies

The therapeutic use of autologous MSC is expected to be safe, as has been reported in all other disease settings currently reported in the literature. As has been shown *in vitro* by our group for

nephrotic syndrome (1) and *in vivo* by our collaborators at the Mario Negri Institute in another disease setting with many similarities in terms of concomitant immunosuppression (renal transplantation, 137, 155), autologous MSC derived from the bone marrow of patients and used as a medicinal product are expected to exert an immunomodulatory effect. This may allow the relapse-free reduction of oral immunosuppressive therapy, which in turn may contribute to lessening the burden of disease for these difficult patients. The potential immunomodulatory effect of this therapeutic effect in vivo will be investigated in this study by the so-called “Immunomonitoring” (see Protocol Additional File 2), a series of laboratory assays at baseline and at 1, 3, 6, 9 and 12 months following treatment that will evaluate T and B cell function in detail. Therefore, this phase 1 study is expected to provide results on the safety and feasibility of this therapeutic approach and to clarify whether or not it may merit further investigation in randomized controlled trials.

7. SPECIFIC SAFETY ISSUES

Since the discovery of human MSC in the bone marrow, MSC have served as a valuable resource for cell-based therapeutics. Current clinical trials of MSC-based therapies for degenerative diseases have shown promising results, and the applications of MSC have been expanded into immunomodulation to treat graft-versus-host disease and other immune diseases. MSC can be obtained from various sources such as bone marrow, adipose tissues, umbilical cords, and peripheral blood. Due to the rarity of MSC in these biological sources, however, *ex vivo* expansion is usually required to achieve the desired cell numbers for *in vivo* use.

7.1 Risk assessment of raw material

The cellular starting material (autologous bone marrow) and all raw materials have been tested for bacterial and fungal contamination and are required to be negative. In addition, the patient is screened to be negative for HIV1 and 2, HTLV1 and 2, HCV, HBV, and syphilis.

The risk of contamination of the end product with prions, animal or human viruses is considered to be almost non-existent. No animal-derived products are used in the production process. Human derived products such as human platelet lysate is used during the production of *ex vivo* expanded BM derived MSC product and human AB plasma is used as excipient for the product. However, both

these human derived materials have been collected from healthy donors and sample of their donations were tested individually and found to be negative for viral diseases.

7.2 Risk assessment in connection with sterility

The production process is carried out in a class B environment. Opening of containers is reduced to a minimum time at each work shift and is performed in a class A environment constantly monitored for particle contamination. Furthermore, class A and class B environments are monitored for microbiological contamination during critical workshifts. The production personnel has received specific training on aseptic manufacturing including the requirement to successfully perform media fill as part of their qualification. At all steps of the expansion protocol, the cell suspension is visually examined for signs of contamination. Possible microbial contamination of the final product is controlled by a series of tests that include aerobic and anaerobic bacteria, fungi, mycoplasma and endotoxin.

7.3 Adverse reactions, precautions and handling of overdose

7.3.1 Adverse reactions and precautions

To date, MSC have been administered to more than 3000 patients in the treatment of different conditions and no significant side effect was detected (157, 142). No other study has so far shown early or late toxicity associated with MSC infusion. More specifically, according to the safety information derived from data on prospective clinical trials with intravenous delivery of autologous MSC to kidney transplant recipients, performed with the same protocol that we propose for this study and by one of the 2 facilities that will prepare the cellular product for this study, to date in 157 participants no severe infusion reactions or side effects have been described (137, 155).

We can expect fever, local reactions at the infusion site, and/or infusion reaction as possible adverse reactions. These reactions are common following administration of DMSO containing cell therapy products. We plan to minimize this risk by employing premedication with intravenous clorpheniramine at the correct dose and according to local procedures. The acute treatment of infusion reactions consists in supportive measures such as oxygen and intravenous administration of glucocorticoids, antihistamine and antiemetic agents, as detailed in local procedures.

In conclusion, the infusion of *ex vivo* expanded bone marrow derived MSC is a safe procedure associated with a low probability of inducing severe reactions.

7.3.2 Overdose

Although higher doses have been employed without adverse effects in certain clinical contexts, such as acute GvHD (143, 159), available data suggest that a dose of 1-2 x10^6 cells/kg is safe, well-tolerated and capable of inducing clinical benefit (137, 39, 158). The MSC dose was also chosen based on practical considerations, i.e. the ability to grow this quantity of cells for most patients within approximately 28 days (143). Dose-response studies have not been performed in this clinical setting, but at the dose of 1 x 10^6 MSC/kg given twice 7 days apart, as planned in the present study, we do not anticipate any issue of overdosing, as confirmed by the results of studies mentioned above. Moreover, although there are no data regarding accidental overdosage, the nature of the medicinal product and the modality of administration make it extremely unlikely that overdosage could occur. The MSC product is administered by intravenous infusion. The bag(s) containing the MSC product are thawed on a named patient basis following a written and signed doctor's order containing the exact number and volume of bags to be thawed based on the patient's weight in kilograms.

7.4 Risk of neoplastic transformation

One of the major concerns associated with long-term *in vitro* culture of MSC are genomic instability and tumorigenicity. MSC could in theory differentiate in vivo into several mesenchymal tissues and thus lead to ectopic and dysfunctional tissue. Karyotypic abnormalities in cultured BM-MSC from both humans as well as animals have indeed been observed, but these cases merely seem to reflect senescence of gradually diminishing cell populations. While tumor-like cells have been observed in long-term cultures of MSC from rodents (160, 161), such cells have not been seen in long-term human MSC cultures except following culture contamination (162-165). So far, no ectopic tissue formation has been reported in clinical trials utilizing intravenous administered ex vivo cultured allogeneic MSC. Of these studies only one study described one patient developing calcified ectopic lesions on the scalp and foot. Excision biopsies revealed no evidence of MSC donor DNA and therefore these lesions were not considered MSC related (166). The general consensus is that no conclusions can be drawn at the moment, as there is insufficient long-term follow-up data available to detect a slight increase in incidence of malignancy.

For safety reasons the MESNEPH study will therefore only use MSC from short-term culture.

7.5 Reproductive toxicity and teratogenicity

Clinical data on reproductive toxicity in humans are not available. Reproductive toxicity is not expected. As to teratogenicity, mesenchymal cells of fetal origin have been detected in maternal bone marrow (167). It is therefore likely that transfer of mesenchymal cells from mother to fetus is also possible. The effects of such transfer upon the fetus are unknown. Recently 2 children received a prenatal MSC transplantation for osteogenesis imperfecta without any adverse events, but long term follow-up results from these 2 patients have not yet been published (168).

7.6 Conclusions on potential risks

MSC, being a cellular therapeutic agent, should not show any of the toxicities shown by standard therapeutics. Extensive literature suggest that MSC are extremely safe both in terms of early and of late toxicity, since no severe adverse event has been described in about 3000 patients described to date (157, 142). These analyses excluded acute infusional toxicity, organ system complications, infections, death or malignancy. There was only a significant association between MSC administration and transient fever (142). The only potential concern is that MSCs may become transformed in long-term culture and give rise to tumors after in vivo infusion. However, whereas transformation of murine MSC has been reported, such event has never been demonstrated in human MSC. Moreover, we will employ short-term culture (2 passages), with only 1 passage *in vitro* and freezing of MSCs at the second cell detachment step, with a mean expansion time of approximately 24 days, as detailed in the IMPD (section 2.1.S.2.2.2).

We can therefore conclude that the overall potential toxicity of cells grown with our protocol is extremely low.

7.7 Benefits

This is a Phase 1 study with autologous BM, platelet-lysate expanded MSC in patients with difficult forms of idiopathic nephrotic syndrome. The proof of principle study that we performed in vitro confirms that MSC expanded from these patients have comparable regenerative, differentiative and immunomodulatory features compared to MSC expanded from healthy controls. They also appear to have normal senescence and caryotype features, which is relevant from a safety standpoint. No clinical reports are available for patients with this disease. However, in similar clinical contexts, albeit involving only 2 patients, one with focal segmental glomerulosclerosis relapsed post-renal

transplant and one with post-bone marrow transplant nephrotic syndrome, the use of MSC infusions was safe and appeared helpful in improving disease outcome. It has also to be remembered that MSC infusions may provide additional benefits other than immunomodulation to patients with nephrotic syndrome, as in vitro our results showed a beneficial effect on the glomerular filtration barrier following experimental damage induced by Adriamycin. We conclude that the small risk of long term effects, not yet observed following MSC infusions, is fully outweighed by the potential therapeutic benefit from MSC infusions lacking early toxicity and on the contrary several useful biologic properties.

8. CONCLUSIONS

The studies discussed above suggest that MSC treatment appears to be safe, and it has the potential to improve the outcome of idiopathic nephrotic syndrome. Nevertheless, rigorous evaluation in a phase 1 trial is necessary before randomized clinical trials can be considered. The study which we propose has the potential to verify the safety of MSC as therapy for INS and to elucidate in vivo the immunomodulatory effects of MSCs in this clinical setting.

9. REPORTING REQUIREMENTS

After medical decision to enroll a patient in the protocol, a formal request has to be sent to the Cell Factory of each participating center (Officina Farmaceutica IRCCS Ospedale Pediatrico “Bambino Gesù” in Rome and Centro di Terapia Cellulare “G. Lanzani”, ASST Papa Giovanni XXIII in Bergamo) with all the required information. In particular, the doctor should verify whether there is a signed informed consent form and that the regulatory authorities have authorized the treatment. The MSC bag will arrive at the Dipartimento OncoEmatologia e Medicina trasfusionale of the IRCCS Ospedale Pediatrico “Bambino Gesù” in Rome or at theHematology and Bone Marrow Transplant Unit, , Azienda Ospedaliera Papa Giovanni XXIII in Bergamo still frozen in dry ice containing plastic box by authorized courier and the doctor will sign a document at the arrival. After arrival of the bag at the Hospital, the doctor should verify the integrity as well as the state of conservation. Finally, the doctor will verify the label and the release document. The bag will be thawed in a water bath prepared at 37 °C and the doctor will infuse the cells immediately after

thawing at appropriate velocity (upon premedication with anti-histaminic drug) (as detailed in the MESNEPH Project Plan, Appendix 1). The doctor shall immediately inform the cell factory should any adverse event manifest in the 3 hours following infusion, and will fill out an Infusion Report (Appendix 2). During the course of the treatment the doctor in charge will regularly notify the clinical data to the ISS registry of somatic cell therapy.

REFERENCES

1. Starc N, Min L, Algeri M et al. Phenotypic and functional characterization of mesenchymal stromal cells isolated from pediatric patients with severe idiopathic nephrotic syndrome. Cytotherapy, in press
2. Vivarelli, M, Massella L, Ruggiero B, Emma F. Minimal change disease. Clin J Am Soc Nephrol 2017
3. Niaudet P, Boyer, O. Idiopathic Nephrotic Syndrome in Children: Clinical Aspects. In: Pediatric Nephrology-7th edition. Springer-Verlag; 2016: 839-82
4. Colucci M, Corpetti G, Emma F, Vivarelli M. Immunology of idiopathic nephrotic syndrome. Pediatr Nephrol. 2017
5. Bertelli R, Bonanni A, Di Donato A, et al. Regulatory T cells and minimal change nephropathy: in the midst of a complex network. Clin Exp Immunology 2016;183(2):166-74
6. Ravani P, Bonanni A, Rossi R, et al. Anti-CD20 Antibodies for Idiopathic Nephrotic Syndrome in Children. CJASN 2016;11(4):710-20.
7. Ruggenenti P, Ruggiero B, Cravedi P, et al for the Rituximab in Nephrotic Syndrome of Steroid-Dependent or Frequently Relapsing Minimal Change Disease Or Focal Segmental Glomerulosclerosis (NEMO) Study Group: Rituximab in steroid-dependent or frequently relapsing idiopathic nephrotic syndrome. JASN 2014;25(4): 850-63.
8. Vivarelli M, Moscaritolo E, Tsalkidis A, Massella L, Emma F. Time for Initial Response to Steroids Is a Major Prognostic Factor in Idiopathic Nephrotic Syndrome. J Pediatr 156:965-71 (2010)
9. Fakhouri F, Bocquet N, Taupin P, et al. Steroid-sensitive nephrotic syndrome: from childhood to adulthood. AJKD 2003; 41(3): 550-7
10. Colucci M, Carsetti R, Cascioli S, et al. B Cell Reconstitution after Rituximab Treatment in Idiopathic Nephrotic Syndrome. JASN 2016;27(6):1811-22
11. Roberts DM, Jones RB, Smith RM, et al. Rituximab-associated hypogammaglobulinemia: incidence, predictors and outcomes in patients with multi-system autoimmune disease. J Autoimm 2015; 57:60-5.
12. Anolik JH, Barnard J, Owen T, et al. Delayed memory B cell recovery in peripheral blood and lymphoid tissue in systemic lupuserythematosus after B cell depletion therapy. Arthritis and Rheum 2007;56(9):3044-56.
13. Cameron JS. The nephrotic syndrome and its complications. AJKD 1987;10(3): 157-71.
14. Caplan, A.I. The mesengenic process. *Clin Plast Surg* **21**, 429-435 (1994).
15. Deans, R.J. & Moseley, A.B. Mesenchymal stem cells: biology and potential clinical uses. *Experimental hematology* **28**, 875-884 (2000).
16. Pittenger, M.F.*, et al.* Multilineage potential of adult human mesenchymal stem cells. *Science* **284**, 143-147 (1999).
17. Tavassoli, M. & Friedenstein, A. Hemopoietic stromal microenvironment. Am J Hematol 15, 195-203 (1983).
18. Devine, S.M., Cobbs, C., Jennings, M., Bartholomew, A. & Hoffman, R. Mesenchymal stem cells distribute to a wide range of tissues following systemic infusion into nonhuman primates. Blood 101, 2999-3001 (2003).

19. Aggarwal, S. & Pittenger, M.F. Human mesenchymal stem cells modulate allogeneic immune cell responses. Blood 105, 1815-1822 (2005).
20. Di Nicola, M., et al. Human bone marrow stromal cells suppress T-lymphocyte proliferation induced by cellular or nonspecific mitogenic stimuli. Blood 99, 3838-3843 (2002).
21. Krampera, M., et al. Bone marrow mesenchymal stem cells inhibit the response of naive and memory antigen-specific T cells to their cognate peptide. Blood 101,3722-3729 (2003).
22. Le Blanc, K., Tammik, L., Sundberg, B., Haynesworth, S.E. & Ringden, O. Mesenchymal stem cells inhibit and stimulate mixed lymphocyte cultures and mitogenic responses independently of the major histocompatibility complex. Scandinavian journal of immunology 57, 11-20 (2003).
23. Bieback, K. & Kluter, H. Mesenchymal stromal cells from umbilical cord blood. Curr Stem Cell Res Ther 2, 310-323 (2007).
24. Campagnoli, C., et al. Identification of mesenchymal stem/progenitor cells in human first-trimester fetal blood, liver, and bone marrow. Blood 98, 2396-2402 (2001).
25. Erices, A., Conget, P. & Minguell, J.J. Mesenchymal progenitor cells in human umbilical cord blood. Br J Haematol 109, 235-242 (2000).
26. Fernandez, M., et al. Detection of stromal cells in peripheral blood progenitor cell collections from breast cancer patients. Bone Marrow Transplant 20, 265-271 (1997).
27. Nakahara, H., et al. In vitro differentiation of bone and hypertrophic cartilage from periosteal-derived cells. Exp Cell Res 195, 492-503 (1991).
28. Nathanson, M.A. Bone matrix-directed chondrogenesis of muscle in vitro. Clin Orthop Relat Res, 142-158 (1985).
29. Fibbe, W.E. & Noort, W.A. Mesenchymal stem cells and hematopoietic stem cell transplantation. Ann N Y Acad Sci 996, 235-244 (2003).
30. Dominici, M., Le Blanc K, Mueller I et al. Minimal criteria for defining multipotent mesenchymal stromal cells. The International Society for Cellular Therapy position statement. Cytotherapy 8, 315-317 (2006).
31. Bernardo ME, Fibbe WE. Mesenchymal stromal cells: sensors and switchers of inflammation, Cell Stem Cell (2013);13:392.402.
32. Nauta AJ, Fibbe WE. Immunomodulatory properties of mesenchymal stromal cells. Blood. 2007;110(10):3499-506
33. Le Blanc K, Frassoni F, Ball L, et al. Mesenchymal stem cells for treatment of steroid-resistant, severe, acute graft-versus-host disease: a phase II study. Lancet. 2008 371(9624):1579-86
34. Deng D, Zhang P, Guo Y et al. A randomised double-blind, placebo-controlled trial of allogeneic umbilical cord-derived mesenchymal stem cell for lupus nephritis. Ann. Rheum. Dis. (2017)
35. Peired AJ, Sisti A, Romagnani P. Mesenchymal Stem Cell-Based Therapy for Kidney Disease: A Review of Clinical Evidence. Stem Cells International (2016)
36. Casiraghi F, Perico N, Cortinovis M and Remuzzi G. Mesenchymal stromal cells in renal transplantation: opportunities and challenges. Nat Rev Nephrology 2016; 12:241-253
37. Belingheri M, Lazzari L, Parazzi V et al. Allogeneic mesenchymal stem cell infusion for the stabilization of focal segmental glomerulosclerosis. Biologicals (2013) 41:439e445

38. Zhang Y, Peng X, Fan Z et al. Mesenchymal stem cells may ameliorate nephrotic syndrome post-allogeneic hematopoietic stem cell transplantation-Case Report Front Imm (2017) 8:962
39. Ciccocioppo R, Bernardo ME, Sgarella A, Maccario R, Avanzini MA, Ubezio C, et al. Autologous bone marrow-derived mesenchymal stromal cells in the treatment of fistulising Crohn's disease. Gut 2011;60:788-98
40. Duijvestein M, Vos AC, Roelofs H, Wildenberg ME, Wendrich BB, Verspaget HW, et al. Autologous bone marrow-derived mesenchymal stromal cell treatment for refractory luminal Crohn's disease: results of a phase I study. Gut 2010;59:1662-9.
41. Dietz AB, Dozois EJ, Fletcher JG, Butler GW, Radel D, Lightner AL, et al. Autologous Mesenchymal Stem Cells, Applied in a Bioabsorbable Matrix, for Treatment of Perianal Fistulas in Patients With Crohn's Disease'. Gastroenterology 2017; doi:10.1053/j.gastro.2017.04.001.
42. De Jesus MM, Santiago JS, Trinidad CV, See ME, Semon KR, Fernandez MO Jr et al. Autologous adipose-derived mesenchymal stromal cells for the treatment of psoriasis vulgaris and psoriatic arthritis: A case report. Cell Transplant 2016.
43. Cohen JA, Imrey PB, Planchon SM, Bermel RA, Fisher E, Fox RJ, et al. Pilot trial of intravenous autologous culture-expanded mesenchymal stem cell transplantation in multiple sclerosis. Mult Scler 2017.
44. Sykova E, Rychmach P, Drahoradova I, Konradova S, Ruzickova K, Vorisek I, et al. Transplantation of Mesenchymal Stromal Cells in Patients With Amyotrophic Lateral Sclerosis: Results of Phase I/IIa Clinical Trial. Cell Transplant 2017;26:647-58.
45. Poulin MF, Deka A, Mohamedali B, Schaer GL. Clinical Benefits of Stem Cells for Chronic Symptomatic Systolic Heart Failure: A Systematic Review of the Existing Data and Ongoing Trials. Cell Transplant 2016;25:1911-23.
46. Carlsson PO, Schwarcz E, Korsgren O, Le Blanc K. Preserved beta-cell function in type 1 diabetes by mesenchymal stromal cells. Diabetes 2015;64:587-92.
47. Tyndall A, van Laar JM. Stem cell transplantation and mesenchymal cells to treat autoimmune diseases. Presse Med 2016;45:e159-69.
48. Eirin A, Zhu XY, Puranik AS, Tang H, McGurren KA, van Wijnen AJ, et al. Mesenchymal stem cell-derived extracellular vesicles attenuate kidney inflammation. Kidney Int 2017;doi: 10.1016/j.kint.2016.12.023.
49. Saad A, Dietz AB, Herrmann SMS, Hickson LJ, Glockner JF, McKusick MA, et al. Autologous Mesenchymal Stem Cells Increase Cortical Perfusion in Renovascular Disease. J Am Soc Nephrol 2017
50. Makhlough A, Shekarshian S, Mhogadasali R et al. Safety and tolerability of autologous bone marrow mesenchymal stromal cells in ADPKD patients. Stem Cell Research & Therapy (2017) 8:116
51. Willhite, C.C. & Katz, P.I. Toxicology updates. Dimethyl sulfoxide. J Appl Toxicol 4, 155-160 (1984).
52. Brayton, C.F. Dimethyl sulfoxide (DMSO): a review. Cornell Vet 76, 61-90 (1986)
53. Gratwohl, A., et al. Hematopoietic stem cell transplantation: a global perspective.JAMA 303, 1617-1624 (2010)

54. Berz, D., McCormack, E.M., Winer, E.S., Colvin, G.A. & Quesenberry, P.J.Cryopreservation of hematopoietic stem cells. Am J Hematol 82, 463-472 (2007)
55. Zambelli, A., et al. Clinical toxicity of cryopreserved circulating progenitor cells infusion. Anticancer Research 18, 4705-4708 (1998)
56. Windrum, P., et al. Variation in dimethyl sulfoxide use in stem cell transplantation: a survey of EBMT centres. Bone Marrow Transpl 36, 601-603 (2005).
57. Shu, Z., Heimfeld, S. & Gao, D. Hematopoietic SCT with cryopreserved grafts:adverse reactions after transplantation and cryoprotectant removal before infusion. Bone Marrow Transplant 49, 469-476 (2014)
58. Davis, J.M., Rowley, S.D., Braine, H.G., Piantadosi, S. & Santos, G.W. Clinical toxicity of cryopreserved bone marrow graft infusion. Blood 75, 781-786 (1990).
59. Morris, C., et al. Should the standard dimethyl sulfoxide concentration be reduced? Results of a European Group for Blood and Marrow Transplantation prospective noninterventional study on usage and side effects of dimethylsulfoxide. Transfusion 54, 2514-2522 (2014).
60. Gerdoni, E., et al. Mesenchymal stem cells effectively modulate pathogenic immune response in experimental autoimmune encephalomyelitis. Ann Neurol 61, 219-227 (2007).
61. Stagg, J. Immune regulation by mesenchymal stem cells: two sides to the coin. Tissue Antigens 69, 1-9 (2007).
62. Burr, S.P., Dazzi, F. & Garden, O.A. Mesenchymal stromal cells and regulatory T cells: the Yin and Yang of peripheral tolerance? *Immunology and cell biology* **91**,12-18 (2013).
63. Casado, J.G., Tarazona, R. & Sanchez-Margallo, F.M. NK and MSCs crosstalk: the sense of immunomodulation and their sensitivity. *Stem cell reviews* **9**, 184-189 (2013).
64. Franquesa, M., Hoogduijn, M.J., Bestard, O. & Grinyo, J.M. Immunomodulatory effect of mesenchymal stem cells on B cells. *Frontiers in immunology* **3**, 212 (2012).
65. Nauta, A.J., Kruisselbrink, A.B., Lurvink, E., Willemze, R. & Fibbe, W.E. Mesenchymal stem cells inhibit generation and function of both CD34(+)-derived and monocyte-derived dendritic cells. J Immunol 177, 2080-2087 (2006).
66. Francois, M., Romieu-Mourez, R., Li, M. & Galipeau, J. Human MSC suppression correlates with cytokine induction of indoleamine 2,3-dioxygenase and bystander M2 macrophage differentiation. Molecular therapy : the journal of the American Society of Gene Therapy 20, 187-195 (2012).
67. Maggini, J., et al. Mouse bone marrow-derived mesenchymal stromal cells turn activated macrophages into a regulatory-like profile. PloS one 5, e9252 (2010).
68. Melief, S.M., et al. Multipotent Stromal Cells Induce Human Regulatory T Cells Through a Novel Pathway Involving Skewing of Monocytes Toward Antiinflammatory Macrophages. Stem cells 31, 1980-1991 (2013).
69. Le Blanc, K., Tammik, C., Rosendahl, K., Zetterberg, E. & Ringden, O. HLA expression and immunologic properties of differentiated and undifferentiated mesenchymal stem cells. Experimental hematology 31, 890-896 (2003)
70. Erkers, T., Nava, S., Yosef, J., Ringden, O. & Kaipe, H. Decidual stromal cells promote regulatory T cells and suppress alloreactivity in a cell contact-dependent manner. Stem Cells Dev 22, 2596-2605 (2013).

71. Augello, A., *et al.* Bone marrow mesenchymal progenitor cells inhibit lymphocyte proliferation by activation of the programmed death 1 pathway. *European journalof immunology* **35**, 1482-1490 (2005).
72. Xue, Q., *et al.* The Negative Co-Signaling Molecule B7-H4 Is Expressed by Human Bone Marrow-Derived Mesenchymal Stem Cells and Mediates its T-Cell Modulatory Activity. *Stem Cells Dev* **19**, 27-37 (2010).
73. Akiyama, K., *et al.* Mesenchymal-stem-cell-induced immunoregulation involves FAS-ligand-/FAS-mediated T cell apoptosis. *Cell stem cell* **10**, 544-555 (2012).
74. Saldanha-Araujo, F., et al. Mesenchymal stromal cells up-regulate CD39 andincrease adenosine production to suppress activated T-lymphocytes. Stem cell research 7, 66-74 (2011).
75. Nasef, A., et al. Identification of IL-10 and TGF-beta transcripts involved in the inhibition of T-lymphocyte proliferation during cell contact with human mesenchymal stem cells. Gene expression 13, 217-226 (2007).
76. Selmani, Z., *et al.* HLA-G is a crucial immunosuppressive molecule secreted by adult human mesenchymal stem cells. *Transplantation* **87**, S62-66 (2009).
77. Gieseke, F., *et al.* Human multipotent mesenchymal stromal cells use galectin-1 to inhibit immune effector cells. *Blood* **116**, 3770-3779 (2010).
78. Krampera, M., et al. Role for interferon-gamma in the immunomodulatory activity of human bone marrow mesenchymal stem cells. Stem cells 24, 386-398 (2006).
79. Ren, G., et al. Mesenchymal stem cell-mediated immunosuppression occurs via concerted action of chemokines and nitric oxide. Cell stem cell 2, 141-150 (2008).
80. Opitz, C.A., et al. Toll-like receptor engagement enhances the immunosuppressive properties of human bone marrow-derived mesenchymal stem cells by inducing indoleamine-2,3-dioxygenase-1 via interferon-beta and protein kinase R. Stem cells 27, 909-919 (2009).
81. Meisel, R., et al. Human bone marrow stromal cells inhibit allogeneic T-cell responses by indoleamine 2,3-dioxygenase-mediated tryptophan degradation. Blood 103, 4619-4621 (2004).
82. Ryan, J.M., Barry, F., Murphy, J.M. & Mahon, B.P. Interferon-gamma does not break, but promotes the immunosuppressive capacity of adult human mesenchymal stem cells. Clinical and experimental immunology 149, 353-363 (2007).
83. Waterman, R.S., Tomchuck, S.L., Henkle, S.L. & Betancourt, A.M. A new mesenchymal stem cell (MSC) paradigm: polarization into a pro-inflammatory MSC1 or an Immunosuppressive MSC2 phenotype. PloS one 5, e10088 (2010).
84. Grohmann, U., Fallarino, F. & Puccetti, P. Tolerance, DCs and tryptophan: much ado about IDO. Trends Immunol 24, 242-248 (2003).
85. Ge, W., et al. Regulatory T-cell generation and kidney allograft tolerance induced by mesenchymal stem cells associated with indoleamine 2,3-dioxygenase expression. Transplantation 90, 1312-1320 (2010).
86. Tatara, R., et al. Mesenchymal stromal cells inhibit Th17 but not regulatory T-cell differentiation. Cytotherapy 13, 686-694 (2011).

87. Fibbe, W. E. & Rabelink, T. J. (2017) Mesenchymal stromal cells in lupus nephritis Nat. Rev. Nephrol. 2017.100
88. Broekman W, Roelofs H, Zarcone MC, Taube C, Stolk J, Hiemstra PS. Functional characterisation of bone marrow-derived mesenchymal stromal cells from COPD patients. ERJ Open Res 2016;2: 10.1183/23120541.00045-2015.
89. Davies LC, Alm JJ, Heldring N, Moll G, Gavin C, Batsis I, et al. Type 1 Diabetes Mellitus Donor Mesenchymal Stromal Cells Exhibit Comparable Potency to Healthy Controls In Vitro. Stem Cells Transl Med 2016;5:1485-95.
90. Rosado MM, Bernardo ME, Scarsella M, Conforti A, Giorda E, Biagini S, et al. Inhibition of B-cell proliferation and antibody production by mesenchymal stromal cells is mediated by T cells. Stem Cells Dev 2015;24:93-103
91. Bucciarelli E, Binazzi R, Santori P, Vespasiani G. Nephrotic syndrome in rats due to adriamycin chlorhydrate. Lav Ist Anat Istol Patol Univ Studi Perugia 1976;36:53-69.
92. Bertani T, Poggi A, Pozzoni R, Delaini F, Sacchi G, Thoua Y, et al. Adriamycin-induced nephrotic syndrome in rats: sequence of pathologic events. Lab Invest 1982;46:16-23.
93. Okuda S, Oh Y, Tsuruda H, Onoyama K, Fujimi S, Fujishima M. Adriamycin-induced nephropathy as a model of chronic progressive glomerular disease. Kidney Int 1986;29:502-10.
94. Chen A, Wei CH, Sheu LF, Ding SL, Lee WH. Induction of proteinuria by adriamycin or bovine serum albumin in the mouse. Nephron 1995;69:293-300.
95. Chen A, Sheu LF, Ho YS, Lin YF, Chou WY, Chou TC, et al. Experimental focal segmental glomerulosclerosis in mice. Nephron 1998;78:440-52.
96. Gewirtz DA. A critical evaluation of the mechanisms of action proposed for the antitumor effects of the anthracycline antibiotics adriamycin and daunorubicin. Biochem Pharmacol 1999;57: 727-41.
97. Park SS, Eom YW, Choi KS. Cdc2 and Cdk2 play critical roles in low dose doxorubicin-induced cell death through mitotic catastrophe but not in high dose doxorubicin-induced apoptosis. Biochem Biophys Res Commun 2005;334:1014-21.
98. Rebbaa A, Zheng X, Chou PM, Mirkin BL. Caspase inhibition switches doxorubicin-induced apoptosis to senescence. Oncogene 2003;22:2805-11.
99. Li M, Armelloni S, Zennaro C, Wei C, Corbelli A, Ikehata M, et al. BDNF repairs podocyte damage by microRNA-mediated increase of actin polymerization. J Pathol 2015;235:731-44.
100. Wang Y, Wang Y, Feng X, Bao S, Yi S, Kairaitis L, et al. Depletion of CD4(+) T cells aggravates glomerular and interstitial injury in murine adriamycin nephropathy. Kidney Int 2001;59:975-84.
101. Wang Y, Wang YP, Tay YC, Harris DC. Role of CD8(+) cells in the progression of murine adriamycin nephropathy. Kidney Int 2001;59:941-9.
102. Mahajan D, Wang Y, Qin X, Wang Y, Zheng G, Wang YM, et al. CD4+CD25+ regulatory T cells protect against injury in an innate murine model of chronic kidney disease. J Am Soc Nephrol 2006;17:2731-41.

103. Wang Y, Mahajan D, Tay YC, Bao S, Spicer T, Kairaitis L, et al. Partial depletion of macrophages by ED7 reduces renal injury in Adriamycin nephropathy. Nephrology (Carlton) 2005;10:470-7.
104. Magnasco A, Corselli M, Bertelli R, Ibatici A, Peresi M, Gaggero G, et al. Mesenchymal stem cells protective effect in adriamycin model of nephropathy. Cell Transplant 2008;17:1157-67.
105. Zoja C, Garcia PB, Rota C, Conti S, Gagliardini E, Corna D, et al. Mesenchymal stem cell therapy promotes renal repair by limiting glomerular podocyte and progenitor cell dysfunction in adriamycin-induced nephropathy. Am J Physiol Renal Physiol 2012;303:F1370-81.
106. Guo J, Zou Y, Wu Z, Wu W, Xu Z, Hu H, et al. Protective effects of mesenchymal stromal cells on adriamycin-induced minimal change nephrotic syndrome in rats and possible mechanisms. Cytotherapy 2014;16:471-84.
107. Wu HJ, Yiu WH, Wong DWL et al Human induced pluripotent stem cell-derived mesenchymal stem cells prevent adriamycin nephropathy in mice. Oncotarget 2017 10;8(61).
108. Casiraghi F, Perico N, Remuzzi G. Mesenchymal stromal cells for tolerance induction in organ transplantation. Hum Immunol. 2017 in press
109. Kawai, T. et al. Long-term results in recipients of combined HLA-mismatched kidney and bone marrow transplantation without maintenance immunosuppression. Am. J. Transplant. 14, 1599–1611, 2014
110. Leventhal, J. et al. Chimerism and tolerance without GVHD or engraftment syndrome in HLA-mismatched combined kidney and hematopoietic stem cell transplantation. Sci. Transl. Med. 4, 124ra28, 2012
111. Scandling, J. D. et al. Tolerance and chimerism after renal and hematopoietic-cell transplantation. N. Engl. J. Med. 358, 362–368, 2008
112. Wood, K. J., Bushell, A. & Hester, J. Regulatory immune cells in transplantation. Nat. Rev. Immunol. 12, 417–430, 2012
113. Ge, W. et al. Regulatory T cell generation and kidney allograft tolerance induced by mesenchymal stem cells associated with indoleamine 2,3 dioxygenase expression. Transplantation 90, 1312–1320, 2010
114. Franquesa, M. et al. Mesenchymal stem cell therapy prevents interstitial fibrosis and tubular atrophy in a rat kidney allograft model. Stem Cells Dev. 21, 3125–3135, 2012
115. Hara, Y. et al. In vivo effect of bone marrow-derived mesenchymal stem cells in a rat kidney transplantation model with prolonged cold ischemia. Transpl. Int. 24, 1112–1123, 2011
116. Seifert, M., Stolk, M., Polenz, D. & Volk, H. D. Detrimental effects of rat mesenchymal stromal cell pre-treatment in a model of acute kidney rejection. Front. Immunol. 3, 202, 2012
117. Koch, M. et al. Isogeneic MSC application in a rat model of acute renal allograft rejection modulates immune response but does not prolong allograft survival. Transpl. Immunol. 29, 43–50, 2013

118. Casiraghi, F. et al. Localization of mesenchymal stromal cells dictates their immune or proinflammatory effects in kidney transplantation. Am. J. Transplant. 12, 2373–2383, 2012
119. Marquez-Curtis, L. A. & Janowska-Wieczorek, A. Enhancing the migration ability of mesenchymal stromal cells by targeting the SDF 1/CXCR4 axis. Biomed. Res. Int. 2013, 561098, 2013
120. Cao, Z. et al. Protective effects of mesenchymal stem cells with CXCR4 up regulation in a rat renal transplantation model. PLoS ONE 8, e82949, 2013
121. Liu, N., Patzak, A. & Zhang, J. CXCR4 overexpressing bone marrow-derived mesenchymal stem cells improve repair of acute kidney injury. Am. J. Physiol. Renal Physiol. 305, F1064–F1073, 2013
122. Morigi M, Rota C, Remuzzi G: Mesenchymal stem cells in kidney repair, Methods Mol Biol, Mesenchymal Stem Cells, vol 1416, 89-107, 2016
123. Eirin A, Xiang-Yang Zhu, Amrutesh S et al. Mesenchymal stem cell–derived extracellular vesicles attenuate kidney inflammation, Kidney International 92(1):114-124 2017
124. Griffin TP, Martin WP, Islam N, O'Brien T, Griffin MD: The promise of mesenchymal stem cell therapy for diabetic kidney disease, Curr Diabetes reports 16:42, 2016
125. Perico N, Casiraghi F, Remuzzi G. Clinical Translation of Mesenchymal Stromal Cell Therapies in Nephrology. Journal American Society Nephrol 2018 Feb;29(2):362-375.
126. Krampera, M. Mesenchymal stromal cell 'licensing': a multistep process. Leukemia 25, 1408-1414 (2011).
127. Ren, G., et al. Species variation in the mechanisms of mesenchymal stem cell mediated immunosuppression. Stem cells 27, 1954-1962 (2009).
128. Le Blanc, K., Tammik, C., Rosendahl, K., Zetterberg, E. & Ringden, O. HLA expression and immunologic properties of differentiated and undifferentiated mesenchymal stem cells. Experimental hematology 31, 890-896 (2003).
129. Eliopoulos, N., Stagg, J., Lejeune, L., Pommey, S. & Galipeau, J. Allogeneic marrow stromal cells are immune rejected by MHC class I- and class II mismatched recipient mice. Blood 106, 4057-4065 (2005).
130. Chan, J.L., et al. Antigen-presenting property of mesenchymal stem cells occurs during a narrow window at low levels of interferon-gamma. Blood 107, 4817- 4824 (2006).
131. Stagg, J., Pommey, S., Eliopoulos, N. & Galipeau, J. Interferon-gamma stimulated marrow stromal cells: a new type of nonhematopoietic antigen presenting cell. Blood 107, 2570-2577 (2006).
132. Beggs, K.J., et al. Immunologic consequences of multiple, high-dose administration of allogeneic mesenchymal stem cells to baboons. Cell transplantation 15, 711-721 (2006).
133. Nauta, A.J., et al. Donor-derived mesenchymal stem cells are immunogenic in an allogeneic host and stimulate donor graft rejection in a nonmyeloablative setting. Blood 108, 2114-2120 (2006).
134. Schu, S., et al. Immunogenicity of allogeneic mesenchymal stem cells. Journal of cellular and molecular medicine 16, 2094-2103 (2012).
135. Griffin, M.D., et al. Anti-donor immune responses elicited by allogeneic mesenchymal stem cells: what have we learned so far? Immunology and cell biology 91, 40-51 (2013).

136. Berglund AK, Fortier LA, Douglas F et al. Immunoprivileged no more: measuring the immunogenicity of allogeneic adult mesenchymal stem cells. Stem Cell Research and Therapy 22;8(1):288 2017
137. Perico N, Casiraghi F, Introna M et al. Autologous Mesenchymal Stromal Cells and Kidney Transplantation: A Pilot Study of Safety and Clinical Feasibility. CJASN (2011) 6:412–422
138. Pan X, Zheng L, Lai L. Bone marrow-derived mesenchymal stem cell therapy for decompensated liver cirrhosis: A meta-analysis. World J Gastro (2014): 20(38): 14051-14057
139. Zhao R, Su Z, Wu J et al. Serious adverse events of cell therapy for respiratory diseases: a systematic review and meta-analysis. Oncotarget (2017):8(18): 30511-30523
140. Li R, Li X, Cheng J. Clinical efficacy and safety of autologous stem cell transplantation for patients with ST-segment elevation myocardial infarction. Therapeutics and Clinical Risk Management (2016):12 1171–1189
141. El-Badawy A, El-Badri N. Clinical Efficacy of Stem Cell Therapy for Diabetes Mellitus: A Meta-Analysis. PlosONE (2016)
142. Lalu M, McIntyre L, Pugliese C et al. Safety of Cell Therapy with Mesenchymal Stromal Cells (SafeCell): A Systematic Review and Meta-Analysis of Clinical Trials. PlosONE (2012)
143. Introna M, Lucchini G, Dander E et al. Treatment of graft versus host disease with mesenchymal stromal cells: a phase I study on 40 adult and pediatric patients, Biol. Blood Marrow Transplant. (2014)375.381
144. Jin, S.Z., et al. Ex vivo-expanded bone marrow stem cells home to the liver and ameliorate functional recovery in a mouse model of acute hepatic injury Hepatobiliary & pancreatic diseases international : HBPD INT 11, 66-73 (2012).
145. Sasaki, M., et al. Mesenchymal stem cells are recruited into wounded skin and contribute to wound repair by transdifferentiation into multiple skin cell type. J Immunol 180, 2581-2587 (2008).
146. Sullivan, C., et al. Allogeneic murine mesenchymal stem cells: migration to inflamed joints in vivo and amelioration of collagen induced arthritis when transduced to express CTLA4Ig. Stem Cells Dev 22, 3203-3213 (2013).
147. Jackson, J.S., Golding, J.P., Chapon, C., Jones, W.A. & Bhakoo, K.K. Homing of stem cells to sites of inflammatory brain injury after intracerebral and intravenous administration: a longitudinal imaging study. Stem cell research & therapy 1, 17 (2010).
148. McFarlin, K., et al. Bone marrow-derived mesenchymal stromal cells accelerate wound healing in the rat. Wound repair and regeneration : official publication of the Wound Healing Society [and] the European Tissue Repair Society 14, 471-478 (2006).
149. Wu, J., et al. Intravenously administered bone marrow cells migrate to damaged brain tissue and improve neural function in ischemic rats. Cell transplantation 16, 993-1005 (2008).
150. Kidd, S., et al. Direct evidence of mesenchymal stem cell tropism for tumor and wounding microenvironments using in vivo bioluminescent imaging. Stem cells 27, 2614-2623 (2009).

151. Lee, R.H., et al. Intravenous hMSCs improve myocardial infarction in mice because cells embolized in lung are activated to secrete the anti-inflammatory protein TSG-6. Cell stem cell 5, 54-63 (2009).
152. Sackstein, R., et al. Ex vivo glycan engineering of CD44 programs human multipotent mesenchymal stromal cell trafficking to bone. Nature medicine 14, 181-187 (2008).
153. Mahmood, A., Lu, D., Lu, M. & Chopp, M. Treatment of traumatic brain injury in adult rats with intravenous administration of human bone marrow stromal cells. Neurosurgery 53, 697-702; discussion 702-693 (2003).
154. Mouiseddine, M., et al. Human mesenchymal stem cells home specifically to radiation-injured tissues in a non-obese diabetes/severe combined immunodeficiency mouse model. The British journal of radiology 80 Spec No 1, S49-55 (2007).
155. Perico N, Casiraghi F, Gotti E, Introna M, Todeschini M, Cavinato RA, Capelli C, Rambaldi A, Cassis P, Rizzo P, Cortinovis M, Noris M, Remuzzi G. Mesenchymal stromal cells and kidney transplantation: pretransplant infusion protects from graft dysfunction while fostering immunoregulation. Transpl Int. 2013 Sep;26(9):867-78
156. Maccario, R., A. Moretta, A. Cometa, D. Montagna, P. Comoli, F. Locatelli, M.Podesta, and F. Frassoni. 2005. Human mesenchymal stem cells and cyclosporin a exert a synergistic suppressive effect on in vitro activation of alloantigen-specific cytotoxic lymphocytes. Biol Blood Marrow Transplant 11: 1031-1032.
157. Casiraghi, F., G. Remuzzi, M. Abbate, and N. Perico. 2012. Multipotent mesenchymal stromal cell therapy and risk of malignancies. Stem Cell Rev 9: 65-79
158. Connick P, Kolappan M, Crawley C et al. Autologous mesenchymal stem cells for the treatment of secondary progressive multiple sclerosis: an open-label phase 2a proof-of-concept study. Lancet Neurol 2012;11: 150-6
159. Kebriaei P, Robinson S. Treatment of graft-versus-host-disease with mesenchymal stromal cells. Cytotherapy 2011;13(3):262-8.
160. Miura, M., et al. Accumulated chromosomal instability in murine bone marrow mesenchymal stem cells leads to malignant transformation. Stem cells 24, 1095-1103 (2006).
161. Tolar, J., et al. Sarcoma derived from cultured mesenchymal stem cells. Stem cells 25, 371-379 (2007).
162. Bernardo, M.E., et al. Human bone marrow-derived mesenchymal stem cells do not undergo transformation after long-term In vitro culture and do not exhibit telomere maintenance mechanisms. Cancer Research 67, 9142-9149 (2007).
163. Rosland, G.V., et al. Long-term Cultures of Bone Marrow-Derived Human Mesenchymal Stem Cells Frequently Undergo Spontaneous Malignant Transformation (This article contains errors due to a cross contamination of the cell lines used. To correct this issue a letter in Cancer Res. 2010 was published Aug 1,70(15),6393-6). Cancer Research 69, 5331-5339 (2009).
164. Rubio, D., et al. Molecular Characterization of Spontaneous Mesenchymal Stem Cell Transformation. PloS one 3(2008).

165. Torsvik, A., et al. Spontaneous Malignant Transformation of Human Mesenchymal Stem Cells Reflects Cross-Contamination: Putting the Research Field on Track (Correction letter for Cancer Res. 2009 Jul 1, 69(13), 5331-9).Cancer Research 70, 6393-6396 (2010).
166. Prasad, V.K., et al. Efficacy and Safety of Ex Vivo Cultured Adult Human Mesenchymal Stem Cells (Prochymal (TM)) in Pediatric Patients with Severe Refractory Acute Graft-Versus-Host Disease in a Compassionate Use Study.Biology of Blood and Marrow Transplantation 17, 534-541 (2011).
167. O'Donoghue, K. & Chan, J. Human fetal mesenchymal stem cells. Curr Stem Cell Res Ther 1, 371-386 (2006).
168. Chan, J.K. & Gotherstrom, C. Prenatal transplantation of mesenchymal stem cells to treat osteogenesis imperfecta. Front Pharmacol 5, 223 (2014).

Appendix 1

PROJECT PLAN MESNEPH PROTOCOL

MESNEPH MSC Version 1
A prospective study to assess safety and efficacy of the use of bone marrow derived MESenchymal stromal cells as immunomodulatory therapy for children and young adults with severe and difficult-to-treat frequently relapsing or steroid-dependent idiopathic NEPHrotic syndrome: the MESNEPH study.

Principal Investigator : Marina Vivarelli

Coordinating investigator :

Sponsor : OPBG

EudraCT number : 2016-004804-77

INDEX

1 PROTOCOL DESCRIPTION 64
1.1 OBJECTIVES 64
1.2 DURATION OF THE STUDY 64
1.3 ACTIVITIES TO BE IMPLEMENTED FOR THE PROPER PERFORMANCE OF THE PROTOCOL 64
1.4 DESCRIPTION OF IMPLEMENTATION OF PROCEDURES 64

PROTOCOL DESCRIPTION

OBJECTIVES

The primary end-point of the study is to test the safety of autologous mesenchymal stromal cells for the treatment of steroid-dependent nephrotic syndrome.

The secondary endpoints are:

- Recurrence of INS.
- The dose of immunosuppressive therapy to prevent further INS relapses;
- Adverse effects of immunosuppressive therapy (arterial hypertension and need for antihypertensive therapy, obesity and impaired glucose tolerance, dyslipidemia, renal dysfunction, stunted statural growth, infections);
- Kidney function at baseline and at one year after MSC administration;

DURATION OF THE STUDY

40 months

ACTIVITIES TO BE IMPLEMENTED FOR THE PROPER PERFORMANCE OF THE PROTOCOL

ACTIVITIES	RELATED SOPs
STORAGE	OPBG_AMCC_POS210
THAWING	OPBG_AMCC_POS102
TAKING CHARGE	OPBG_AMCC_POS100
INFUSION	OPBG_PT_POS427
RELEASE	OPBG_PT_POS102

DESCRIPTION OF IMPLEMENTATION OF PROCEDURES

ACTIVITIES	RELATED SOPs	IMPLEMENTATIONS REQUIRED
STORAGE	OPBG_AMCC_POS210	☐Study products are stored in the drawer "transit outgoing" in TANK 3. The recording of the temporary storage unit in the drawer transit will done on the "AMCC-MOD212 output transit drawer TANK3".(see chapter 5 of the releated SOP). This SOP also applies to MSC product
RECEPTION	OPBG_AMCC_POS100	☐Study product are taken in charge from AMCC operators according to the procedure for reception (see chapter 5.4.2 of the related SOP). This SOP also applies to MSC product
THAWING	OPBG_AMCC_POS105	☐ Contact the physician or nurse attending the study product administration to double-check whether the thawing procedure can indeed be initiated. Ensure that transport to the site of study product administration will be ready in 10 minutes from start of the thawing procedure.<br><br>☐ Retrieve one unit of the MESNEPH study product using a transport container filled with liquid N2.<br><br>☐ Put on protective gloves and retrieve the unit of MESNEPH study product from the transport-container. Leave the unit (still inside the cassette) at room temperature for exactly 5 minutes.<br><br>☐ After exactly 5 minutes, take the unit (still inside the cassette) and place it in the extra plastic bag.<br>☐ Place the plastic bag containing the unit in the plasma thawer or waterbath, leave it in for exactly 1 minute under constant gentle movement.

		☐ Take the plastic bag containing the unit out of the plasma thawer of waterbath, remove the unit from the plastic bag, gently remove the MESNEPH study product bag from the cassette, and place the MESNEPH study product bag (without the cassette) back in the plastic bag.<br><br>☐ Place the plastic bag containing the MESNEPH study product in the plasma thawer or waterbath. Continue thawing under gentle constant movement until the product is almost completely thawed (a few little ice clumps remain visible).<br><br>☐ Take the plastic bag containing the MESNEPH study product from the plasma thawer or waterbath. Remove the plastic bag from the product bag. Gently mix the contents once more (by gently moving the product bag).<br><br>☐Check the contents for irregularities.<br><br>☐ Place the plastic bag containing the MESNEPH study product in the transport container with cooling elements 4-8°C.<br><br>☐ Fill out and add any required forms as per institutional guidelines as well as the **MESNEPH infusion report** (for both infusions). Add the following instructions via an additional label or via an additional document as per institutional guidelines.<br>☐This study product should be administered directly following arrival at the site of administration.

		☐ If additional units of the MESNEPH study product need to be thawed, repeat the Thawing Procedure.
INFUSION	OPBG_PT_POS427	☐The procedure for infusion of MESNEPH study product follows the procedure for the infusion of cryopreserved hematopoietic progenitor cells (HPC)(see chapter 5.2 of the releated SOP).<br><br>☐The study product is thawed in the clean room directly prior to administration and is released for immediate infusion. After packaging the product is immediately transported on cold packs (4°C). Following the thawing procedure, the product is placed in the labeled non-transparent overpouch that is then sealed to ensure blinding of the patients and medical and nursing staff<br><br>☐The study product should be infused directly following arrival at the site of administration.<br><br>☐Following infusion the system should be flushed with 0.9% Sodium Chloride, Injection (USP) or facility approved solutions as per local guidelines.<br><u>The product bag should not be flushed at the end of the infusion.</u><br><br>☐The investigational medicinal product infusion report form must be filled out by the physician on call and sent to the address provided on the form.

OPBG_AMCC_POS210

OPBG_AMCC_POS100

OPBG_AMCC_POS102

OPBG_PT_POS105

OPBG_PT_POS427

AMCC-MOD212 output transit drawer TANK3

MESNEPH PROTOCOL v1

Appendix 2

INFUSION REPORT MESNEPH PROTOCOL

MESNEPH MSC Version 1
A prospective study to assess safety and efficacy of the use of bone marrow derived MESenchymal stromal cells as immunomodulatory therapy for children and young adults with severe and difficult-to-treat frequently
relapsing or steroid-dependent idiopathic NEPHrotic syndrome:
the MESNEPH study.

Principal Investigator : Marina Vivarelli

Sponsor : OPBG

EudraCT number : 2016-004804-77

THIS STUDY FORM MUST BE DISTRIBUTED TO THE PATIENT WARD

N.B.: To be filled in by the local investigator

MESNEPH patient study number:

Infusion: 1st / 2nd

PATIENT Attach hospital record label of patient

Attach label of MESNEPH study product

To be filled in by the responsible physician for administration of the MESNEPH study product

1. Was there an infusion reaction < 24 hours following administration of the MESNEPH study product?

[ ] No [ ] Yes

If yes:

Time product administration: …..:….

Time occurrence reaction: …..:….

Severity reaction:

☐ grade 0 (no morbidity)

☐ grade I (limitid morbidity)

☐ grade II (significant morbidity)

☐ grade III (life-threatening)

☐ grade IV (death due to reaction)

Prior transfusion reactions?: yes / no

Alternative explanation?: yes/no

If so, please specify ……………………...

☐ ≥ 2° C rise in body temperature

☐ Chills

☐ Tachycardia: HR of ……. to ……./min

☐ Hypotension: BP of …../….. to …../….. mmHg

☐ Hypertension: BP of …../….. to …../….. mmHg

☐ Dyspnea, hypoxia: O2 sat of …..% to .....%

☐ Thoracic pain

☐ Nausea/vomiting

☐ Itch, urticaria

☐ Erythema of the skin

☐ Glottic edema

☐ ICU admission < 24 hour following administration

☐ Other, please specify ………………………… ……………………………………………………...

2. Did any other problems occur during administration of the MESNEPH study product?

[ ] No

[ ] Yes, please specify ……………………………………………….

STATEMENT

The above has been filled out completely and truthfully:

Name of the responsible physician:
……………………………………………………………………...

Date: . . /. ./. . . . Signature: ……………………………...

This MESNEPH study product infusion report must be filled out and signed within 48 hours following administration of the product, and should be sent to the quality officer of the OPBG: **farmacovigilanza.mesneph@opbg.net**.

INVESTIGATIONAL MEDICINAL PRODUCT DOSSIER

**Drug Product**	MSC
**Clinical Trial title**	Phase I prospective study to assess safety and efficacy of the use of bone-marrow derived mesenchymal stromal cells as immunomodulatory therapy for children and young adults with severe and difficult-to-treat frequently relapsing or steroid-dependent idiopathic nephrotic syndrome
**Clinical trial acronym**	MESNEPH
**Eudra CT number**	2016-004804-77
**Version**	02
**Release Date**	June 2018
**Sponsor**	Ospedale Pediatrico Bambino Gesù

TABLE OF CONTENT

2.1 Quality data 9
2.1.S DRUG SUBSTANCE 9
2.1.S.1 General information 9
2.1.S.1.1 Nomenclature 9
2.1.S.1.2 Structure 9
2.1.S.1.3 General properties 9
2.1.S.2 Manufacture 10
2.1.S.2.1 Manufacturer(s) 10
2.1.S.2.2 Description of the manufacturing process and process controls 12
2.1.S.2.2.1 Batch and scale definition 12
2.1.S.2.2.2 Manufacturing process description 12
2.1.S.2.3 Control of materials 15
2.1.S.2.3.1 Raw materials and reagents 15
2.1.S.2.4 Control of critical steps and intermediates 17
2.1.S.2.5 Process validation and/or evaluation 17
2.1.S.2.6 Manufacturing process development 18
2.1.S.3 Characterization 20
2.1.S.3.1 Elucidation of structure and other characteristics 20
2.1.S.3.2 Impurities 21
2.1.S.3.2.1 Process related impurities 21
2.1.S.3.2.2 Product related impurities and other cellular impurities 22
2.1.S.4 Control of Drug Substance 22
2.1.S.4.1 Specification 22
2.1.S.4.2 Analytical procedures 23
2.1.S.4.2.1 Cell viability 23
2.1.S.4.2.2 Sterility 23
2.1.S.4.2.3 Mycoplasma 24
2.1.S.4.2.4 Endotoxin 24
2.1.S.4.2.5 Immunophenotype analysis 24
2.1.S.4.2.6 Colony assay in methylcellulose 24
2.1.S.4.2.7 Karyotyping 25
2.1.S.4.3 Validation of analytical procedures 25
2.1.S.4.4 Batch analyses of the Drug substance 25
2.1.S.4.5 Justification of the specifications 26
2.1.S.5 Reference standard materials 27
2.1.S.6 Container closure 27
2.1.P DRUG PRODUCT 28
2.1.P.1 Manufacturer 28
2.1.P.1.1 Manufacturer 28
2.1.P.1.2 Batch formula 29
2.1.P.1.3 Description of manufacturing process and process controls 30
2.1.P.1.4 Controls of critical steps and intermediates 31
2.1.P.1.5 Process validation and/or evaluation 31
2.1.P.1.5.1 Validation of aseptic manufacturing process 31
2.1.P.2 Control of excipients 32
2.1.P.2.1 Specifications 32
2.1.P.2.2 Excipients of human or animal origin 32

2.1.P.3 Control of the Investigational Medicinal Product ... 32
2.1.P.3.1 Specifications ... 32
2.1.P.3.2 Analytical procedures ... 32
2.1.P.3.3 Validation of Analytical procedures ... 33
2.1.P.3.4 Batch analyses of the final product ... 33
2.1.P.3.5 Characterization of impurities ... 33
2.1.P.3.6 Justification of the specifications ... 33
2.1.P.4 Reference standard ... 33
2.1.P.5 Container closure system ... 33
2.1.P.6 Stability ... 33
2.1.A APPENDICES ... 36
2.1.A.1 Facilities and equipment ... 36
2.1.A.1.1 Description of the facilities and equipment ... 43
2.1.A.1.1.1 Description of the production area ... 45
2.1.A.1.1.2 Description of the quality control area ... 53
2.1.A.1.1.3 Description of the warehouse and GMP product storage area ... 54
2.1.A.1.1.4 Environmental cleaning and monitoring ... 57
2.1.A.1.1.5 GMP declaration ... 58
2.1.A.1.2 Transportation, storage and traceability of the DS and the DP and starting relevant material ... 58
2.1.A.1.2.1 Transportation of the Drug Substance ... 58
2.1.A.1.2.2 Transportation of the Drug Product ... 59
2.1.A.1.2.3 Transportation of relevant starting material ... 59
2.1.A.1.2.4 Storage of the Drug Substance ... 59
2.1.A.1.2.5 Storage of the Drug Product ... 59
2.1.A.1.2.6 Storage of relevant starting materials ... 59
2.1.A.1.2.7 Traceability of the DS and the DP and relevant materials ... 59
2.1.A.1.2.7.1 Labelling and traceability of the Drug Substance 59
2.1.A.1.2.7.2 Labelling and traceability of the Drug Product 59
2.1.A.1.2.7.3 Labelling and traceability of relevant starting material 61
2.1.A.2 Adventitious agents safety evaluation ... 62
2.2 Section 2.2 – Non clinical pharmacology and toxicology data 63

LIST OF FIGURES

FIGURE 1.FLOW CHART DRUG SUBSTANCE 13
FIGURE 2. FLOW CHART DRUG PRODUCT 30
FIGURE 3 ANNEX 6A 37
FIGURE 4 ANNEX 6B 38
FIGURE 5 ANNEX 6C 39
FIGURE 6 ANNEX 6D 40
FIGURE 7 ANNEX 6E 41
FIGURE 8 ANNEX 6F 42
FIGURE 9: HVAC SYSTEM LAYOUT 44
FIGURE 10 ROOMS CLASSIFICATION 48
FIGURE 11 OVER PRESSURE SYSTEMS 49
FIGURE 12 MATERIAL FLOW DIAGRAM 50
FIGURE 13 PERSONNEL FLOW DIAGRAM 51
FIGURE 14 WAREHOUSE AND GMP PRODUCT STORAGE AREA 56
FIGURE 15A EXAMPLE OF DRUG PRODUCT LABEL 60
FIGURE 15B EXAMPLE OF DRUG PRODUCT LABEL 61
FIGURE 16 LABELS FOR AUTOLOGOUS DONOR CELLS 61

LIST OF TABLES

TABLE 1 LIST OF THE REAGENTS 16
TABLE 2 OF-OPBG PROCESS EVALUATION RESULTS 17
TABLE 3 LABORATORIO LANZANI PROCESS EVALUATION RESULTS........ 17
TABLE 4 IMPLEMENTED CHANGES 19
TABLE 5 OF-OPBG PRODUCED MSC RESULTS........ 20
TABLE 6 OF-OPBG PRODUCED MSC RESULTS AND SPECIFICTIONS........ 20
TABLE 7 TEST MATERIAL, CHARACTERIZATION AND RELEASE TESTS 21
TABLE 8 MINIMAL THEORETICAL DILUTION FACTOR OF RAW MATERIALS........ 22
TABLE 9 TESTS AND SPECIFICATION FOR DS QUALITY CONTROL 23
TABLE 10 DATA FROM 2 EXPANSIONS OF MSC FROM HEALTHY DONORS’ BM IN ROME 25
TABLE 11 DATA FROM 2 EXPANSIONS OF MSC FROM LIVING-DONOR KIDNEY TRANSPLANT PATIENTS’BM IN BERGAMO 26
TABLE 12 TEST METHODS AND ACCEPTANCE CRITERIA 32
TABLE 13 MSC STABILITY EVALUATION AFTER THAWING 35
TABLE 14 FUNCTION OF DIFFERENT CLASS AREAS 46
TABLE 15 LIMITS FOR NON VIABLE PARTICLES. 58
TABLE 16 LIMITS FOR VIABLE PARTICLES 58

ABBREVIATIONS

AIFA	Agenzia Italiana del Farmaco
ATMP	Advanced therapy medicinal product
BM	Bone Marrow
BM-MSC	Bone Marrow derived Mesenchymal stromal Cells
CF	Cell Factory
DMSO	DiMethylSulphOxide
DP	Drug Product
DS	Drug Substance
EMA	European Medicine Agency
EP	European Pharmacopeia
EU	Endotoxin Units
FACS	Fluorescent Activated Cell Sorting
GMP	Good Manufacturing Practice
HBV	Hepatitis B Virus
HCV	Hepatitis C Virus
HIV-1	Human Immunodeficiency Virus 1
HLA	Human Leukocyte Antigen
IMPD	Investigational Medicinal Product Dossier
INS	Idiopathic nephrotic syndrome
ISO	International Organization for Standardization
IV	Intravenous
JACIE	Joint Accreditation Committee-ISCT & EBMT
JCI	Joint Commission international
LAL	Lymulus Amebocyte Lysate
MSC	Mesenchymal stromal Cells
NK	Natural Killer
OF	Officina Farmaceutica
OPBG	Ospedale Pediatrico Bambino Gesù
PCR	Polymerase Chain Reaction
QC	Quality control
SOP	Standard Operating Procedure

LIST OF ANNEXES

Annex 1	AIFA_Autorizzazione OF-OPBG
Annex 2	AIFA_Autorizzazione Lab. LanzaniBergamo
Annex 3	CoA Alpha MEM with GlutaMAX
Annex 4	CoA Platelet Lysate
Annex 5	CoA Triple select Enzyme
Annex 6	CoA CryoSure DMSO
Annex 7	CoA CryoMACS Freezing bag 250
Annex 8	CoA MSC OF-OPBG
Annex 9	CoA MSC Lab. Lanzani Bergamo

1 Section 1 - Introduction

This Clinical Trial Application presents information concerning a novel treatment for idiopathic nephrotic syndrome using Bone Marrow derived Mesenchymal stromal Cells (BM-MSCs).

Idiopathic nephrotic syndrome (INS) is the most common glomerular disease of childhood(1-2). The incidence is around 1-3/100.000 children below 16 years of age. Onset occurs mostly between the ages of 2 and 7 with a slight preponderance in males, often following an infectious episode, classically of the upper respiratory tract. Its clinical manifestation is edema, and laboratory exams reveal intense proteinuria, leading to hypoalbuminemia and therefore to intravascular volume depletion and oliguria. All children at onset are treated with a standard course of oral prednisone, to which the majority (75-90%) respond within a few days/weeks. Patients who do not respond to 4 weeks of prednisone given at 60 mg/m2 are defined as steroid resistant, and about half of these will subsequently respond in a variable amount of time, up to about 12 months, to second-line immunosuppression. In those who maintain steroid-resistance often a genetic defect of podocyte components leading to disruption of the integrity of the glomerular basement membrane is the cause of disease. All forms that are steroid- sensitive are instead defined as INS, to signify that the mechanism determining proteinuria is not fully elucidated (2). However, a vast body of experimental evidence, coupled with the clinical response of INS to immunosuppressive therapy, clearly implicates the immune system in disease pathogenesis, through its production of factors that damage the podocyte (3). T cells have been implicated in this process, and a reduction of T regulatory cells has been shown (4). The role of B cells, in particular memory B cells, is suggested by the effectiveness of their depletion by anti-CD20 therapy in preventing relapses (5-6). In addition to prednisone, to which the vast majority of patients are exquisitely sensitive, second-line steroid-sparing immunosuppression used in patients with INS includes levamisole, calcineurin inhibitors (cyclosporine A and tacrolimus), mycophenolate mophetil and antiproliferative agents (azathioprine, cyclophosphamide). In the last decade the successful use of rituximab, a monoclonal anti-CD20 antibody, has suggested that B lymphocytes may be pivotal in disease pathogenesis (5-6).

Mesenchymal stromal cells (MSC) are multipotent progenitor cells resident in the bone marrow and other tissues. MSC can be obtained from bone marrow cells after in vitro culture and expansion. Since their discovery, MSC have been shown to be able to differentiate into several mesenchymal tissues, as well as home to inflamed sites contributing to tissue repair and mediate potent immunomodulatoryeffects both in vitro and in vivo (7). The capacity to regulate immune responses leading to regeneration of damaged tissues provides a strong rationale for the use of MSC as a new treatment modality, especially in diseases characterized by the presence of an immune dysregulation leading to tissue damage. The early in vitro observations that MSC were able to inhibit T cell proliferation induced by allo-antigens, mitogens, CD3, and CD28 agonistic antibodies led to the notion that MSC could be used therapeutically to modulate immune responses (8). A combination of in vitro co-culture studies and animal studies demonstrated that, in addition to inhibiting the proliferation and activation of effector T cells, MSC can also induce the expansion of regulatory T cells and inhibit the expansion and activation of B cells and NK cells (9). MSC can furthermore inhibit the differentiation and activation of myeloid-derived dendritic cells and induce the skewing of monocytes and macrophages toward an anti-inflammatory phenotype (10). Together, these data indicate that MSC can suppress the activation of the immune system by regulating cells of the adaptive and innate immune systems. Therefore, the therapeutic use of MSC has been attempted in a variety of immune-mediated diseases and of kidney diseases, with encouraging results in terms both of safety and of efficacy (9,11-12).

We propose to evaluate the safety and feasibility of the therapeutic use of autologous MSC harvested and purified from the bone marrow (BM) of children with INS, expanded ex vivo and reinfused to the same patient. Given their immunomodulatory properties, our working hypothesis is that their therapeutic use will permit relapse-free reduction of oral immunosuppression in INS patients.

2 Section 2

2.1 Quality data

2.1.S Drug substance

2.1.S.1 General information

The investigational medicinal product consists of autologous mesenchymal stromal cells (MSC), a somatic cell therapy product obtained from isolation and expansion of patient's derived bone marrow (BM) cells. The tissue is procured in accordance with EMA Tissues & Cells Directive, 2004/23/EC, as amended and the product is produced in accordance with Good Manufacturing Practices (GMP).

The manufacturing process for the cell product is continuous. Bone Marrow derived mononuclear cells (BM-MNC) are isolated, culture expanded and finally collected.

These steps are described in the drug substance sections 2.1.S. The final wash step, resuspension in product formulation buffer, filling in the primary container and cryopreservation of the final product is described in the drug product sections 2.1.P. It has been decided to put all information concerning the active substance manufacture (e.g., manufacturing process, impurities, raw materials, cell stock release testing) in the applicable S sections. Information, specific to the product (e.g., fill and finish, release testing, drug product stability, container closure system, composition, excipients) is described in the applicable P sections.

2.1.S.1.1 Nomenclature

Autologous ex vivo expanded and cryopreserved bone marrow-derived Mesenchymal Stromal Cells (MSC).

The term MSC will be used throughout the document.

2.1.S.1.2 Structure

MSC is a somatic cell therapy product consisting of autologous mesenchymal stromal cells isolated from bone marrow through ex vivo expansion using their plastic adherent properties and are characterized by the expression of certain cell surface markers (CD73, CD90 e CD105) or lack thereof (CD45; CD34; CD14 or CD11b, CD79alpha or CD19 or human leukocyte antigen HLA-DR). MSC are adherent and present a fibroblast-like shape.

Structure based on flow cytometry analysis of cell surface markers (details are provided in section 2.1.S.4.2.5) indicates that a homogenous cell population is obtained in the final product expressing cell surface markers consistent with mesenchymal phenotype.

2.1.S.1.3 General properties

MSC is an autologous human somatic cell therapy product composed of a suspension of ex-vivo expanded human bone marrow derived cells produced in accordance with GMP. MSC is a cryopreserved cell suspension that is thawed immediately prior to administration to patients. Flow cytometry testing of MSC shows that the cells express epitopes consistent with a mesenchymal cell phenotype.

MSC are multipotent progenitor cells resident in the bone marrow and other tissues. Since their discovery, MSC have been shown to be able to differentiate into several mesenchymal tissues, as well as home to inflamed sites contributing to tissue repair and mediate potent immunomodulatory effects both in vitro and in vivo. The capacity to regulate immune responses leading to regeneration of damaged tissues provides a strong rationale for the use of MSC as a new treatment modality, especially in diseases characterized by the presence of an immune dysregulation leading to tissue damage. The early in vitro observations that MSC were able to inhibit T cell proliferation induced by allo-antigens, mitogens, CD3, and CD28 agonistic antibodies led to the notion that MSC could be used therapeutically to modulate immune responses (8). A combination of in vitro co-culture studies and animal studies demonstrated

that, in addition to inhibiting the proliferation and activation of effector T cells, MSC can also induce the expansion of regulatory T cells and inhibit the expansion and activation of B cells and NK cells (9). MSC can furthermore inhibit the differentiation and activation of myeloid-derived dendritic cells and induce the skewing of monocytes and macrophages toward an anti-inflammatory phenotype (10). Together, these data indicate that MSC can suppress the activation of the immune system by regulating cells of the adaptive and innate immune systems.

MSC derived from INS patients maintain properties comparable to MSC derived from healthy subjects (13).

All these evidences support the scientific rationale for employing autologous bone marrow-derived MSC in vivo in the treatment of patients with INS.

2.1.S.2 Manufacture

2.1.S.2.1 Manufacturer(s)

The bone marrow of the patient will be collected at the Joint Accreditation Committee-ISCT & EBMT (JACIE). accredited OPBG facility according to JACIE standards or collected at the Hematology Unit of Azienda Socio Sanitaria Territoriale Papa Giovanni XXIII and transported at room temperature (15-25°C) to OF-OPBG or to the Cell Factory in Bergamo.

Suitable collection procedures are in compliance with EU Directive 2004/23/EC and EU Directive 2006/17/EC.

Human BM-MSC are produced and cryopreserved at the following GMP facilities:

- Officina Farmaceutica-Ospedale Pediatrico Bambino Gesù

  Viale San Paolo 15

  00146 Roma

  (GMP licence **aM86/2016;** QP Franca Fassio)

- Centro di Terapia Cellulare "G. Lanzani" (Laboratorio Lanzani)

  Azienda Socio Sanitaria Territoriale Papa Giovanni XXIII (ASST-PG23)

  Via Garibaldi 11/13

  24124 Bergamo

  (Last GMP licence **aM-28/2017;** QP Martino Introna)

OF-OPBG is an academic GMP facility devoted exclusively to production of investigational ATMPs for human therapeutic trials. The design, construction, and validation of the GMP production facility was reviewed by the Agenzia Italiana del Farmaco (AIFA) to assure full compliance with all current GMPs. The Officina Farmaceutica Ospedale Pediatrico Bambino Gesù has been certified by the Italian medicine agency AIFA, according to D.L.vo 211/2003, for the production of sterile medicinal products, of cell based medicinal products (mesenchymal cells), of gene therapy products (T cells transduced with retroviral), for secondary packaging operations and for microbiological(sterility), chemical, physical, biological tests, all in compliance with GMP regulations (Annex 1).

The Centro di Terapia Cellulare "G. Lanzani" (Laboratorio Lanzani) is an academic GMP facility authorized to produce investigational ATMPs for human therapeutic trials since 2008. The design, construction, and validation of the GMP production facility has been reviewed by AIFA in 6 separate inspections to assure full compliance with all current GMPs. The Laboratorio Lanzani has been certified by AIFA, according to D.L.vo 211/2003 and D.L.vo 200/2007, for the production of sterile cell based

medicinal products of small volume, for secondary packaging operations and for biological quality control tests, all in compliance with GMP regulations (Annex 2).

Other facilities have been selected by Officina Farmaceutica OPBG and by Centro di Terapia Cellulare "G. Lanzani" (Laboratorio Lanzani) for additional activities; in particular Laboratorio di Genetica Medica, JCI and ISO 9001 accreditated and led by Dr Antonio Novelli -OPBG ; Eurofins Biolab Vimodrone (MI), Italy and Laboratorio di Citogenetica e Genetica Medica, led by Dr. Ursula Giussani, ASST Papa Giovanni XXIII

In process controls cell count, viability, FACS analyses, clonogenic assay, endotoxin and stability testing will be performed at the above indicated Cell Factories.

Bioburden, Mycoplasma, are performed at OF-OPBG and Eurofins Biolab.

Karyology testing is performed at Laboratorio di Genetica Medica led by Dr Antonio Novelli – OPBG and at Laboratorio di Citogenetica e Genetica Medica, led by Dr. Ursula Giussani, ASST-PG23

Drug Substance Manufacturing Facilities

Location	Activity
Officina Farmaceutica-OPBG<br>Viale San Paolo 15<br>00146 Roma	Manufacturing of MSC and cryopreservation
Centro di Terapia Cellulare "G. Lanzani"<br>Azienda Ospedaliera Papa Giovanni XXIII –<br>Via Garibaldi, 11/13<br>24124 Bergamo	Manufacturing of MSC and cryopreservation

Drug Substance Testing Facilities

Location	Activity
Officina Farmaceutica-OPBG<br>Viale San Paolo 15<br>00146 Roma	Viability, cell count, flow cytometry, clonogenic assay, sterility, endotoxin, mycoplasma, release testing, stability
Centro di Terapia Cellulare "G. Lanzani"<br>Azienda Ospedaliera Papa Giovanni XXIII –<br>Via Garibaldi, 11/13,<br>24124 Bergamo	Viability, cell count, flow cytometry, clonogenic assay, endotoxin, mycoplasma, release testing, stability
Laboratorio di Genetica Medica-OPBG<br>Viale San Paolo 15<br>00146 Roma	Karyology testing
Laboratorio di Citogenetica e Genetica Medica,<br>ASST Papa Giovanni XXIII<br>Piazza OMS, 1<br>24127 Bergamo	Karyology testing
Eurofins Biolab Srl<br>Via Bruno Buozzi, 2<br>20090 Vimodrone (MI)<br>Italy	Sterility and mycoplasma testing of final product for Bergamo

2.1.S.2.2 *Description of the manufacturing process and process controls*

2.1.S.2.2.1 Batch and scale definition

One batch is defined as the pool of the autologous MSCs at the end of the culture. Each batch produced at OF-OPBG is coded as XXYYY.

Where: XX = year

YYY = sequential number

Each batch produced at Laboratorio Terapia Cellulare "G. Lanzani" is coded as MSC and sequential number.

The cells are re-suspended in 10% DMSO and 90% human AB plasma and cryopreserved in aliquots of 20-50 ml (pediatric use) and 50 ml (adult formulation) at the concentration of 1-3 x10^6 cells/ml. The scale is related to the clinical protocol and patient's weight.

2.1.S.2.2.2 Manufacturing process description

A flow diagram of the manufacturing process for the MSC active substance from bone marrow dilution and expansion through cell harvest of MSC with the in-process controls performed during the manufacturing process is provided in Figure 1.

Figure 1.Flow Chart Drug Substance

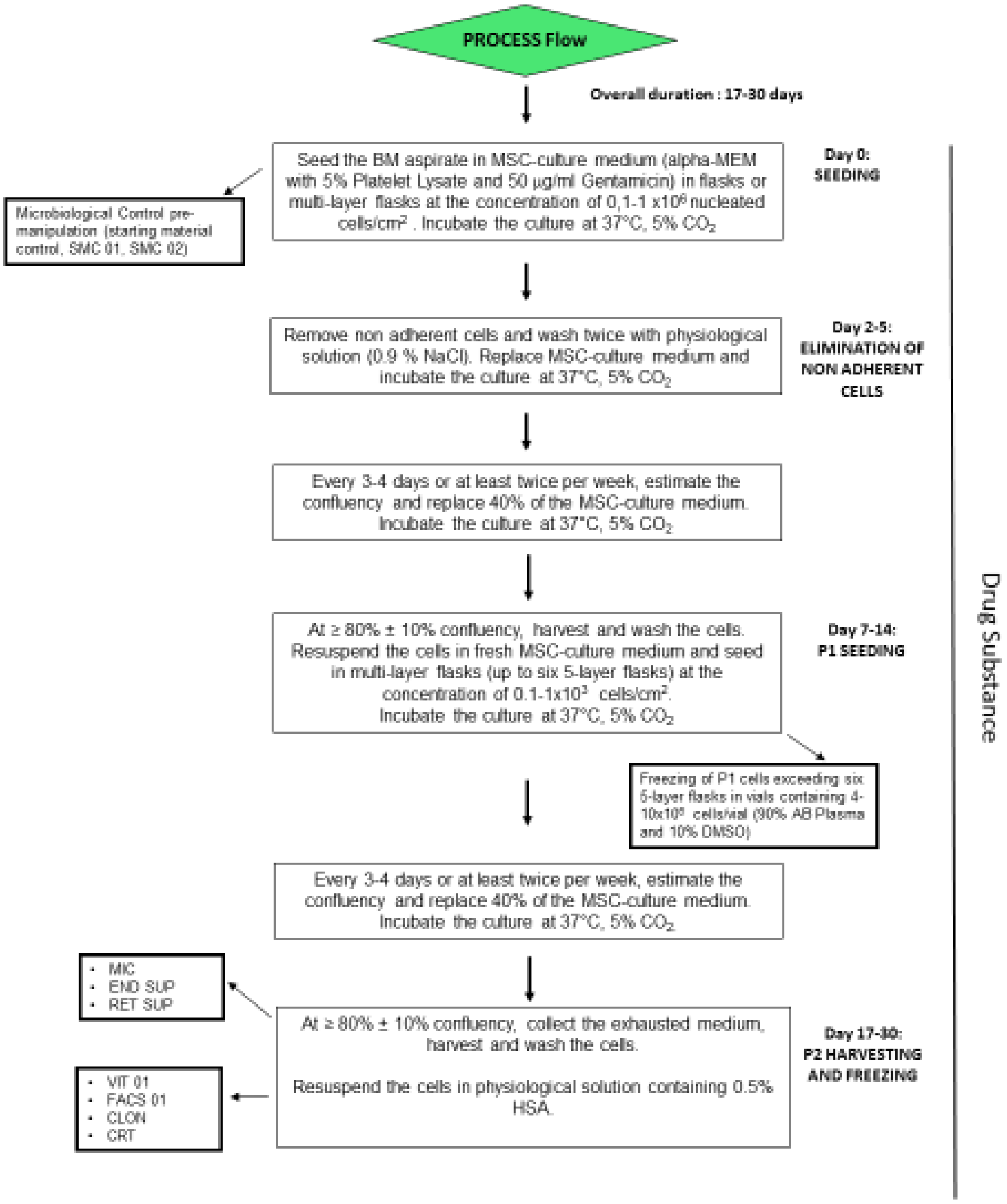

The manufacturing process from patient's BM aspirate to MSC starts with the dilution of bone marrow cells in alpha-MEM enriched with 5% human platelet lysate. The plastic adherent properties of MSC and composition of the culture medium allow for enrichment of the desired cell type; the number of MSC is expanded while other cells residing in BM are removed during culture medium refreshment steps. When confluence of adherent cells is reached, they are harvested by Tryple Select treatment, re-suspended in MSC expansion medium and re-plated in multilayered flasks at a density of 100-1000 cells/cm^2. Further growth with twice weekly medium change is carried out until near confluence. Samples are aseptically collected at each passage to measure viable cell number and percentage of viable cells. Furthermore, cells are checked visually and/or microscopically during the ex vivo expansion process for confluency, morphology and contamination. Open system procedures are executed in a class A Laminar Airflow (LAF) cabinet located in a class B cleanroom environment, where critical parameters for the aseptic process are monitored.

The manufacture process lasts 17-30 days as following

DAY 0: INITIATION OF CULTURE

The patient's bone marrow is collected according to local protocols. After collection in presence of Heparin, the BM aspirate is labelled with a minimum of the patient ID, type cell product, date of sampling and storage conditions. This material is transported to the manufacturing facility at room temperature (15-25°C) using a designated transport box within a timeframe of maximum 4 hours. In the GMP facility the BM aspirate is processed normally within 4-6 hours.

SEEDING: BM is seeded in flasks or multi-layer flasks in MSC-culture medium (alpha-MEM supplemented with 5% Platelet Lysate and 50 mcg/ml Gentamicin) at the concentration of 0.1-1x10^6 nucleated cells/cm^2. Cells are transferred to a CO_2 humidified incubator at the atmosphere containing 5% CO_2 at 37°C.

DAY 2-5: ELIMINATION OF NON ADHERENT CELLS

Non adherent cells are removed and adherent cells are washed twice with physiological solution (0.9 % NaCl). MSC-culture medium is replaced.

Every 3-4 days culture or at least twice per week, confluency is estimated and 40% of MSC-culture medium volume is replaced.

DAY 7-14: P1 SEEDING

At ≥ 80% ± 10% confluency the cells are washed twice with physiological solution and detached with TrypLE Select. The cells are seeded in multi-layer flasks (up to six 5-layer flasks) in MSC-culture medium at the concentration of 0.1-1x10^3 cells/cm^2. P1 MSC exceeding six 5-layer flasks are freezed in vials containing 4-10 x10^6 cells/vial as back up. In both GMP facilities, P1 MSC stability plan studies are ongoing.

Every 3-4 days culture or at least twice per week, confluency is estimated and 40% of MSC-culture medium volume is replaced.

DAY 17-30: P2 HARVESTING AND FREEZING

At ≥ 80% ± 10% confluency the cells are washed twice with physiological solution and detached with TrypLE Select. The cells are then resuspended in physiological solution supplemented with 0.5% HAS, counted and centrifuged.

Cell resuspension and onwards, is described in section *2.1.P.1.3, Description of manufacturing Process and Process Controls,* as are the release and stability tests of the final product.

2.1.S.2.3 Control of materials

2.1.S.2.3.1 Raw materials and reagents

The starting material derives exclusively from patient's bone marrow. It is obtained using aseptic techniques according to hospital protocols and labelled with a unique identification number.

All materials, media and other critical components are received with an accompanying certificate of analysis/conformance. Upon QC review and acceptance of the data and documentation associated with each vendor lot, these materials are released for use in the GMP area.

No animal derived raw material is used in the manufacturing process.

The comprehensive list of the reagents to be used for the production of autologous MSC is reported in the following Table 1. Example certificates of analysis for the reagents in the order presented in Table 1 are provided as appendixes. For raw materials that are authorised as medicinal products, the certificates of analysis of the supplier are not provided.

All the plastic materials which the cells come in contact with are also certified and traceable

Table 1 List of the reagents

Raw Material	Grade	Manufacturing process stage	Concentration used in the process	Source	Vendor	Qualification
Alpha MEM with GlutaMAX	cGMP	Cell culture	94,875%	Chemical	GIBCO	CoA Annex 3
Platelet lysate	Clinical	Cell culture	5%	Biological	COOK Regentec	CoA Annex 4
AB Plasma	Clinical	Cell Freezing	90%	Biological	Octapharma Italia	Medicinal Product: AIC n. 034540017
Gentamicin	Clinical	Cell culture	0,125%	Biological	Hospira Italia	Medicinal Product: AIC n. 032703047
					Fisiopharma	Medicinal Product: AIC n. 031423066
TrypLE Select	cGMP	Cell detachment	100%	Chemical	GIBCO	CoA Annex 5
Sodium Chloride 0.9%	Clinical	Cell washes	0,88%	Chemical	BAXTER SPA	Medicinal Product: AIC n. 030942674
					S.A.L.F.	Medicinal Product: AIC n. 030684070
					Monico	Medicinal Product: AIC n. 030805079
Human Albumin	Clinical	Cell washes	0,5%	Biological	BAXALTA	Medicinal Product: AIC n. 024735033
					KEDRION	Medicinal Product: AIC n. 022515163
DMSO (Cryosure)	USP	Cell Freezing	10%	Chemical	WAK-Chemie Medical GmbH	CoA Annex 6

2.1.S.2.4 Control of critical steps and intermediates

The manufacturing process of MSC is a continuous process without hold steps. In-process sampling is conducted throughout the entire manufacturing process.

Patient's cells can be considered as critical starting material in MSC manufacturing process. Cell count, viability and sterility are performed on the patient BM material. If the microbiological assay gives negative result, the product will be stated "in compliance with the specification".

Upon the arrival at the manufacturing facility, the starting material contained in appropriate cell bag, will be controlled and released according to internal SOPs.

The QC personnel will verify the starting material for several parameters (product identification, bag integrity, the product submission form, compliance with the Italian regulation, microbiological aspect,). After its release, the biological sample will be transferred to classified area and manipulated under class A laminar flow hood in class B background. Sterility is also investigated but results are obtained after initial release of starting material and start of processing.

Cell count and viability are monitored during the production process to determine seeding density for subsequent step in the process and to ensure the final product meets the target dose requirements. During the expansion process medium is changed twice a week.

Cells are visually examined every 3 to 4 days for morphology (spindle shape) and contamination of the culture (culture medium needs to be clear).

2.1.S.2.5 Process validation and/or evaluation

The aseptic manufacturing process from collection of bone marrow aspirate to MSC product harvest was evaluated by conducting process simulation studies. Successful process simulations using Tryptic Soy Broth (TSB) growth media showed no growth and provided documented evidence that the process can be performed aseptically. Process simulation studies for individual steps in the manufacturing process are repeated at least once per year.

Specific process validation studies were addressed in each facility according to their own specific protocol.

Process evaluation data from OF-OPBG production site are presented in table 2. The production date and days in culture between BM-aspirate processing and cryopreservation are depicted. Viability was >90% for all batches at time of cryopreservation. Sterility and mycoplasma were negative.

Table 2 OF-OPBG process evaluation results

Batch	Manufacture date	Volume of BM (ml)	Days in culture	Cell Yield (P2)	% CD73+	% CD90+	% CD105+	% CD45+	% CD3+
15005	17/4/2015	22	28	75x10^6	95.6	99.7	93.3	0.0	0.0
15006	12/5/2015	19	30	120x10^6	99.7	99.8	98.6	0.0	0.1
15007	22/9/2015	25	28	35x10^6	99.3	99.7	95.5	0.1	0.1

Process validation in the Bergamo Cell Factory was performed in 2008-2009. Data of MSC productions (first 3) from living-donor kidney transplant patients' BM, expanded according to the same protocol) are presented in table 3.

Table 3 Laboratorio Lanzani process evaluation results

Batch	Manufacture date	Volume of BM (ml)	Days in culture	Cell Yield (P2)	% $CD73^+$	% $CD90^+$	% $CD105^+$	% $CD45^+$	% $CD3^+$
MSC10	05/05/2008	35	25	202x10^6	99.0	99.0	92.0	2.5	nd
MSC22	05/12/2008	28	22	80x10^6	99.7	99.6	99.4	0.4	0.5
MSC28	10/04/2009	45	22	156x10^6	99.7	99.0	98.9	0.0	0.5

2.1.S.2.6 Manufacturing process development

The definitive manufacturing process is the result of a long experience developed in the Bergamo facility which has produced so far more than 100 batches of BM derived MSC for validation or therapeutic purposes within 7 different clinical protocols. In no case has significant toxicity or immediate avverse reaction been noted upon MSC administration. (14-17)

Efficacy and safety of MSC produced according to this protocol are still under evaluation in several clinical trials, as detailed in the Investigator Brochure and in the specific publications (14-21)

In order to harmonize the production protocol between the two facilities, the OF-OPBG implemented some changes in accordance with the Bergamo facility protocol. These modifications are detailed in table 4.

Table 4 Implemented changes

	MSC OF-OPBG	MSC OF-OPBG and Laboratorio Lanzani for the present study
Seeding	BM is seeded in T25/T75/T175 flasks after ficoll separation	BM is seeded in T175 flasks or multi-layer flasks without separation
Culture Medium	DMEM + 10% FBS	Alpha-MEM + 5% Platelet Lysate
Antibiotics	NO	50 mcg/ml Gentamicin
Cell culture support	Flasks	Cell stack / Flasks
Freezing Medium	Physiologic solution (NaCl 0,9%), Human Serum Albumin 2,25%, DMSO 10%	Human AB Plasma 90%, DMSO 10%
Medium Exchange	100% of MSC-culture medium volume is replaced	40% of MSC-culture medium volume is replaced
Number of passages in culture	3<br>(P1-P2-P3)	2<br>(P1-P2)
Cell Density (P1)	160 x 10^3/cm^2	100-1000 x 10^3 /cm^2
Cell Density (P2-P3)	25 x 10^3/cm^2	0.1-1 x 10^3/cm^2

Although these changes have reduced manipulation to a minimum, process simulation studies for individual steps in the manufacturing process were made in the OF-OPBG and Laboratorio Lanzani.

Furthermore, in the Rome production site, two independent studies have been performed to assess the impact of the changes on the final product.

The produced cells were spindle shaped and did not grow in suspension.

Flow cytometric studies testing MSC cells against 3 defined MSC markers indicated that these cells are of mesenchymal cell phenotype. A single peak is observed for each of the markers tested, indicating a single population of cells.

The results of these studies are summarized in the table 5 and 6 below.

Table 5 OF-OPBG produced MSC results

Batch	BM Volume (ml)	Total Nucleated Cells (x10^{6})	BM Cell Stack	P1 Harvested Cells (x10^{6})	P1 Seeded Cells (x10^{6})	Cell Stack	P2 Harvested Cells (x10^{6})	Overall Duration (days)
**17030**	24	378	1 x CS1	11,4	11,4	6 x CS5	497,6	19
**17031**	45	967,5	3 x CS1	73,5	6,2	6 x CS5	480	19

Table 6 OF-OPBG produced MSC results and specifications

TEST	Specification	Results	
		Lot 17030	Lot 17031
Sterility (anaerobic microorganisms, EP 2.6.27)	Sterile	Sterile	Sterile
Sterility (aerobic microorganisms, EP 2.6.27)	Sterile	Sterile	Sterile
Bacterial Endotoxins (EP 2.6.14)	≤ 5 E.U./mL	< 0.054 E.U./mL	< 0.050 E.U./mL
Mycoplasma (EP 2.6.7)	NEGATIVE	Negative	Negative
Karyotype	No metaphases or no chromosomic aberrations or non-clonal chromosomic aberrations in ≤10% of metaphases	Conform	Conform
Clonogenic assay	Negative	Negative	Negative
Viability	≥ 80 %	92.8 %	90.6 %
Identity	$CD73^+ \geq 90\%$<br>$CD90^+ \geq 90\%$<br>$CD105^+ \geq 90\%$<br>$CD45^+ \leq 5\%$<br>$CD14^+ \leq 5\%$<br>$CD34^+ \leq 5\%$	$CD73^+$ 99.9 %<br>$CD90^+$ 99.8 %<br>$CD105^+$ 99.5 %<br>$CD45^+$ 0.6 %<br>$CD14^+$ 0.3 %<br>$CD34^+$ 0.6 %	$CD73^+$ 99.2 %<br>$CD90^+$ 99.9 %<br>$CD105^+$ 99.3 %<br>$CD45^+$ 0.9 %<br>$CD14^+$ 0.1 %<br>$CD34^+$ 0.5 %
Viability after thawing	F.I.O.	95.3 %	95.2 %
Identity after thawing	F.I.O.	$CD73^+$ 99.4 %<br>$CD90^+$ 99.6 %<br>$CD105^+$ 99.8 %<br>$CD45^+$ 0.4 %<br>$CD14^+$ 0.1 %<br>$CD34^+$ 0.5 %	$CD73^+$ 99.7 %<br>$CD90^+$ 99.9 %<br>$CD105^+$ 99.5 %<br>$CD45^+$ 0.1 %<br>$CD14^+$ 0.0 %<br>$CD34^+$ 0.5 %

2.1.S.3 Characterization

2.1.S.3.1 Elucidation of structure and other characteristics

The active substance produced according to the protocol consists of the patient’s autologous MSCs.

The cells are fully characterized as follows:

- Plastic adherence and spindle shape morphology
- Expression of CD73, CD90 and CD105
- Lack of expression of CD14, CD34 and CD45

In the following table test material, characterization tests and release tests are summerized:

Table 7 Test material, characterization and release tests

	Test name	Starting Material	Exhausted medium	DS	DP
**Safety**	Sterility	X		X*	
	Mycoplasma			X*	
	Serology for viral and microbial infections	X			
	Endotoxin		X*		
**Identity**	Karyotype			X*	
	Flow cytometry			X*	
	Clonogenic assay			X*	
**Purity**	Flow cytometry			X*	
	Cell count			X	X
	Viability			X*	X

*** Release tests**

2.1.S.3.2 Impurities

2.1.S.3.2.1 Process related impurities

The MSC manufacturing process utilizes raw materials described in 2.1.S.2.3 Control of Materials, which are diluted throughout the manufacturing process. The final preparation for cryopreservation is performed by mixing one volume of cells in administration buffer with one volume of cryopreservation mix (20% DMSO) It cannot be excluded that traces of raw material are present in the final MSC product. The minimal dilution factor of all individual raw materials is listed in Table xx. The theoretical concentrations of the components were calculated based on culture and wash volumes used in production.

Table 8 Minimal theoretical dilution factor of raw materials

Raw material name	(minimal theoretical) Dilution factor in end product	Maximal theoretical volume in 100 ml final product	Concentration in final product
gentamicin	$3x10^6$	NA	17 pg/ml
medium	$3x10^6$	16 nl	NA
TrypLE	1000	50 µl	NA

2.1.S.3.2.2 Product related impurities and other cellular impurities

Sterility testing confirms that microbial contaminants cannot be detected.

Antibiotic (gentamicin) is employed in the manufacturing process of MSC to significantly reduce the low residual bioburden associated with BM aspirate collection.

Test method for detection of microbiological contamination is provided in section 2.1.S.4, Control of Drug Substance. This test is qualified taking into account the potential inhibitory effect of growth by gentamicin.

2.1.S.4 Control of Drug Substance

2.1.S.4.1 Specification

The tests reported in the table below are routinely performed on the DS.

Table 9 Tests and specification for DS Quality Control

Test	Specification
Viability	% viable cells ≥ 80%
Sterility EP 2.6.27	negative
Mycoplasma EP 2.6.7	negative
Endotoxin EP 2.6.14	≤ 5 EU/ml
Immunophenotype:<br>$CD73^+$ percentage<br>$CD90^+$ percentage<br>$CD105^+$ percentage	≥ 90%
$CD14^+$ percentage<br>$CD34^+$ percentage<br>$CD45^+$ percentage	≤ 5%
Colony assay in methylcellulose	No colony
Karyotype	No metaphases or no chromosomic aberration or non-clonal chromosomic aberrations in ≤10% of metaphases

2.1.S.4.2 Analytical procedures

2.1.S.4.2.1 Cell viability

After cell collection the washed and concentrated MSC suspension is sampled for cell counting and viability assessment. Manual counting is performed according to analytical method CRE01.

Trypan blue is used for cell counting and to distinguish between viable and dead cells. Non-viable cells are permeable to trypan blue dye and are stained in blue when in contact with the dye. The % viability (%V) is calculated using the following formula: %V = (amount viable cells/ total cells) x 100. The test is performed on the starting material, throughout production and at product release.

2.1.S.4.2.2 Sterility

Sterility of the final cell product is performed according to the chapter 2.6.27 “Microbiological control of cellular products” of the European Pharmacopeia. Microorganism detection is performed by fluorimetric assessment of CO2 production in an automated microbial detection system (BacT/ALERT 3D, Biomerieux) after 7 days incubation in aerobic and anaerobic conditions.

Qualification status: the absence of interference of test article with the sensitivity of the assay (<10 CFU inoculous for bacteria and fungi) has been verified in three independent runs. The assay is validated according to EP 2.6.27 chapter with respect to sensitivity (<10 CFU of prescribed microbial strains) repeatability and intermediate precision.

2.1.S.4.2.3 Mycoplasma

The presence or absence of Mycoplasma in the drug substance is determined by rapid PCR method in accordance with the EP, chapter 2.6.7. The specification for PCR method is the absence of Mycoplasma which results in an acceptance criterion of “negative”.

Qualification status: The assay has been qualified for specificity, sensitivity and repeatability. The absence of interference of the test article with the assay has been verified.

2.1.S.4.2.4 Endotoxin

The level of endotoxin in the final cell product is determined by using the kinetic chromogenic LAL assay in accordance with the EP chapter 2.6.14 ”Bacterial Endotoxins” method D.

The test article dilution is 1:2. At this dilution, the sensitivity of the assay is 0.01 EU/mL. The acceptance criterion is < 5.0 EU/mL.

Qualification status: The assay has been qualified for linearity, precision, and the absence of interference of the test article.

2.1.S.4.2.5 Immunophenotype analysis

The identity of MSC is verified by immunostaining with fluorochrome-conjugated monoclonal antibody directed against the mesenchymal stem cells markers CD73, CD90 and CD105. The possible presence of non-mesenchymal contaminant cells is ruled out by immunostaining with fluorochrome-conjugated monoclonal antibody directed against pan-leukocyte marker CD45, monocyte marker CD14 and the hematopoietic progenitor cell antigen CD34. Cells are immunostained with irrelevant fluorochrome-conjugated isotype control antibodies as negative control.

Stained cells are analyzed by flow cytometry using FACS Canto II flow cytometer in a Forward vs Side scatter -defined morphological gate to exclude cell debris.

MSC must display a percentage of positive cells for CD73, CD90 and CD105, equal or greater than 90% and a percentage of positive cells for CD45, CD14 and CD34 equal or below 5%.

2.1.S.4.2.6 Colony assay in methylcellulose

To determine the absence of transformed cells in the final product, MSC are cultivated in methylcellulose medium. The semisolid nature of the methylcellulose medium enable the cultivation of cells in the absence of adherence to the substrate. The test aims at ruling out the capacity to grow in the absence of adherence to the substrate which is a characteristic of transformed cells. For this purpose, cells re-suspended in methyl cellulose medium are seeded in 35 mm Petri dishes in duplicate, cultivated at 37°C, 5% CO2 for 12-14 days and the presence of colonies is ruled out by microscopy observation. As positive control, the Ewing’s sarcoma cell line PDE-02 is cultivated in parallel with MSCs in the same condition.

The DP must show no clonogenic capacity with concomitant positive growth of PDE-02 cells.

2.1.S.4.2.7 Karyotyping

The test is used to determine that the cells have a normal karyotype of 46 chromosomes. Forty eight to 120 hours from seeding, cell cultures (higher than 1,000,0000 cells) were treated for 3 hours with colcemid solution at a final concentration of 0.05µg/ml to arrest cells in metaphase. Cells were then treated with hypotonic solution and with a fixative solution according to standard procedures. Metaphases of cells were GTG-banded and karyotyped in accordance with the International System for Human Cytogenetic nomenclature recommendations (ISCN, 2016). At least 20 metaphases were analyzed for each sample.

2.1.S.4.3 Validation of analytical procedures

The analytical procedures Sterility, Mycoplasma, Endotoxin used are all Ph.Eur. methods verified for the intended use.

The enumeration of CD73+, CD90+, CD105+, CD14+, CD34+, CD45+ using flow cytometry analysis is performed according to local SOPs.

2.1.S.4.4 Batch analyses of the Drug substance

Data obtained from MSC produced in Rome and in Bergamo are reported in Table 10 and 11.

Table 10 Data from 2 expansions of MSC from healthy donors' BM in Rome

TEST	Specification	Results	
		Lot 17030	Lot 17031
Sterility (anaerobic microorganisms, EP 2.6.27)	Sterile	Sterile	Sterile
Sterility (aerobic microorganisms, EP 2.6.27)	Sterile	Sterile	Sterile
Bacterial Endotoxins (EP 2.6.14)	≤ 5 E.U./mL	< 0.054 E.U./mL	< 0.050 E.U./mL
Mycoplasma (EP 2.6.7)	NEGATIVE	Negative	Negative
Karyotype	No metaphases or no chromosomic aberrations or non-clonal chromosomic aberrations in ≤10% of metaphases	Conform	Conform
Clonogenic assay	Negative	Negative	Negative
Viability	≥ 80%	92.8 %	90.6 %
Identity	$CD73^+$ ≥ 90%<br>$CD90^+$ ≥ 90%<br>$CD105^+$ ≥ 90%<br>$CD45^+$ ≤ 5%<br>$CD14^+$ ≤ 5%<br>$CD34^+$ ≤ 5%	$CD73^+$ 99.9 %<br>$CD90^+$ 99.8 %<br>$CD105^+$ 99.5 %<br>$CD45^+$ 0.6 %<br>$CD14^+$ 0.3 %<br>$CD34^+$ 0.6 %	$CD73^+$ 99.2 %<br>$CD90^+$ 99.9 %<br>$CD105^+$ 99.3 %<br>$CD45^+$ 0.9 %<br>$CD14^+$ 0.1 %<br>$CD34^+$ 0.5 %
Viability after thawing	F.I.O.	95.3 %	95.2 %
Identity after thawing	F.I.O.	$CD73^+$ 99.4 %<br>$CD90^+$ 99.6 %<br>$CD105^+$ 99.8 %<br>$CD45^+$ 0.4 %<br>$CD14^+$ 0.1 %<br>$CD34^+$ 0.5 %	$CD73^+$ 99.7 %<br>$CD90^+$ 99.9 %<br>$CD105^+$ 99.5 %<br>$CD45^+$ 0.1 %<br>$CD14^+$ 0.0 %<br>$CD34^+$ 0.5 %

Table 11 Data from 2 expansions of MSC from living-donor kidney transplant patients'BM in Bergamo

TEST	Specification	Results	
		MSC28	MSC54
Sterility (anaerobic microorganisms, EP 2.6.27)	Sterile	Sterile	Sterile
Sterility (aerobic microorganisms, EP 2.6.27)	Sterile	Sterile	Sterile
Bacterial Endotoxins (EP 2.6.14)	<7 EU/mL	< 5 EU/mL	< 2.5EU/mL
Mycoplasma (EP 2.6.7)	NEGATIVE	Negative	Negative
Karyotype	No metaphases or no chromosomic aberrations or non-clonal chromosomic aberrations in ≤10% of metaphases	Conform	Conform
Clonogenic assay	Negative	Negative	Negative
Viability	≥ 80%	94.3 %	96.6 %
Identity	$CD73^+$ ≥ 90%<br>$CD90^+$ ≥ 90%<br>$CD105^+$ ≥ 90%<br>$CD45^+$ ≤ 5%<br>$CD14^+$ ≤ 5%<br>$CD34^+$ ≤ 5%	$CD73^+$ 99.7 %<br>$CD90^+$ 99%<br>$CD105^+$ 98.9 %<br>$CD45^+$ 0%<br>$CD14^+$ 0 %<br>$CD34^+$ 0 %	$CD73^+$ 98.9 %<br>$CD90^+$ 99.9 %<br>$CD105^+$ 98.9 %<br>$CD45^+$ 0.2 %<br>$CD14^+$ 0.3 %<br>$CD34^+$ 0 %
Viability after thawing	F.I.O	85.7%	84.1%
Identity after thawing	F.I.O	$CD73^+$ 98.7%<br>$CD90^+$ ≥ 99.9%<br>$CD105^+$ ≥ 99.3%	$CD73^+$ ≥ 98.5%<br>$CD90^+$ ≥ 99.7%<br>$CD105^+$ ≥ 98.4%

2.1.S.4.5 Justification of the specifications

Specifications for the MSCs are provided in section 2.1.S.4.1 These proposed specifications have been established based on data collected during development.

Sterility

No contamination of aerobic and anaerobic microbial origin is allowed, test results should be negative.

Endotoxin

The product at harvest is tested for endotoxin levels and these should not exceed 7 EU/ml. The pyrogenic threshold for humans has been established as 5 endotoxin units (EU)/kg. For intravenous products, the Ph.Eur. 2.6.14 "Bacterial Endotoxins" specifies a maximum endotoxin limit of 5.0 EU/kg/hour.

Mycoplasma

No contamination with Mycoplasma is allowed in the MSC drug product as this is a safety concern. Tests should be negative.

Viability

At least 80% of the cells collected after the ex vivo expansion and right before cryopreservation should be viable. Based on historical data viability percentages below 80% have never been obtained.

Immunophenotype analysis of MSC

For the MSCs, the MSC markers CD73, CD105 and CD90 are used for release. A 90% purity for MSC markers is set as release criterion for the final product.

Furthermore, ≤ 5% expression of hematopoietic antigens should be considered as the maximum threshold for cellular impurities potentially present in the MSC product. The maximum threshold for impurity has been adapted to ≤ 5% CD14+ cells, CD34+cells and CD45+ cells. These percentages are well within the detection limit of the procedure.

Karyotype:

No genetic abnormalities are allowed, as this is a safety concern. Karyotype analysis should reveal no genetic abnormalities. However, there is no evidence for malignant transformation of MSC related to the expansion process. Furthermore karyotype analysis may reveal isolated non-clonal apparent abnormalities which are thought to be related to senescence (or dying cells) (21). In some cases, MSCs are not proliferating significantly so that karyotypic analysis is unable to detect metaphases. Therefore the adopted criteria, used by Laboratory Lanzani since 2013 is: No metaphases or no chromosomic aberrations or non-clonal chromosomic aberrations in ≤10% of metaphases

Lack of malignant transformation is confirmed by lack of clonogenic capacity (see below).

Clonogenic assay

The final product must show no clonogenic capacity, with concomitant positive growth of PDE-02 cells.

2.1.S.5 Reference standard materials

Not Applicable.

2.1.S.6 Container closure

CryoMACS® Freezing Bags (Miltenyi Biotec): EVA (ethylene-vinyl-acetate) cryobags. EC Declaration of Conformity and Certificate of Analysis are annexed (Annex 7).

The freezing bag is equipped with EVA tubing for drawing Quality Control samples out of heat-sealed tube sections. The tubing is provided with luer connectors, roller clamp and a luer-lock injection port. In addition, each bag contains two spike ports for removal of the thawed cell product: the single-component ports are secured with sealed, twist-off protective caps which reduce the risk of nitrogen ingress into these ports.

2.1.P Drug Product

The drug product is a cell suspension formulated in administration buffer consisting of 90% human AB plasma and 10% DMSO and is stored frozen in liquid nitrogen vapor phase until thaw directly prior to infusion.

The final product is presented in ethylene vinyl acetate cryopreservation bag (CryoMACS® Freezing Bag 250 - Miltenyi Biotech) in a total volume of 20-50 mL for pediatric patients and 50 mL for adults, with this volume containing the appropriate cell dose for a specific patient. Final concentration of MSC can range from 1-3 x 10^6 cells/ml.

This is a phase 1 study with two administrations within an interval of 7 days. The target concentration of the MSC product to be used in the clinical trial is 1x106 cells/kg recipient body weight.

2.1.P.1 Manufacturer

2.1.P.1.1 Manufacturer

The DP is manufactured at the following facilities:

Drug Product Manufacturing Facilities

Location	Activity
**Officina Farmaceutica-OPBG**<br>**Viale San Paolo 15**<br>**00146 Roma**	Manufacturing of MSC and cryopreservation
**Centro di Terapia Cellulare “G. Lanzani”**<br>**ASST Papa Giovanni XXIII –**<br>**Via Garibaldi, 11/13**<br>**24124 Bergamo**	Manufacturing of MSC and cryopreservation

Drug Product Testing Facilities

Location	Activity
**Officina Farmaceutica-OPBG**<br>**Viale San Paolo 15**<br>**00146 Roma**	Viability, cell count, Flow Cytometry, Clonogenic Assay, Sterility, endotoxin, mycoplasma, release testing, stability
**Centro di Terapia Cellulare "G. Lanzani"**<br>**Azienda Ospedaliera Papa Giovanni XXIII**<br>**Via Garibaldi, 11/13**<br>**24124 Bergamo**	Viability, cell count, flow cytometry, clonogenic assay, endotoxin, release testing, stability
**Laboratorio di Genetica Medica OPBG**<br>**Viale San Paolo 15**<br>**00146 Roma**	Karyology testing
**Laboratorio di Citogenetica e Genetica Medica,**<br>**ASST Papa Giovanni XXIII**<br>**Piazza OMS, 1**<br>**24127 Bergamo**	Karyology testing
**Eurofins Biolab Srl**<br>**Via Bruno Buozzi, 2**<br>**20090 Vimodrone (MI)**<br>**Italy**	Sterility and mycoplasma testing of final product for Bergamo

2.1.P.1.2 Batch formula

The investigational medicinal product consists of autologous MSC, cultured for a maximum of 2 passages and cryopreserved at a final concentration of 1-3 x 10^6 cells /ml in administration buffer consisting of human AB plasma and 10% clinical grade DMSO.

Active substance	20 ml dose<br>Amount per dose<br>(ml)	50 ml dose<br>Amount per dose<br>(ml)
**MSC**	1x10e6 cells/kg	1x10e6 cells/kg

Excipients	20 ml dose<br>Amount per dose<br>(ml)	50 ml dose<br>Amount per dose<br>(ml)
Human AB plasma	18	45
Dimethylsulfoxide DMSO	2	5

2.1.P.1.3 Description of manufacturing process and process controls

A flow diagram of the manufacturing process for the MSC drug product from drug substance is provided in Figure 2.

Figure 2. Flow chart Drug Product

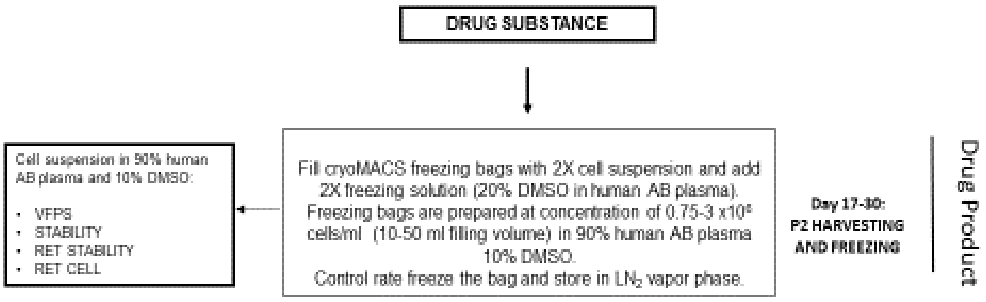

After centrifugation, cells are resuspended in human AB plasma at concentration 2X (2-6 $x10^6$ cells/ml) with respect to the established target concentration (1-3 $x10^6$ cells/ml).

CryoMACS freezing bags (50 and 250 ml formats) are filled with 2X cell suspension and 2X freezing solution (20% DMSO in human AB plasma) is added to obtain a 0.75-3 $x10^6$ cells/ml final concentration in 90% human AB plasma and 10% DMSO (20-50 ml filling volume).

Container Filling:

Cryobags are filled as outlined in *2.1.P.1.2. Batch Formula* and sealed. All labels are verified for label accuracy, legibility and integrity.

Storage: The MSC product bag are frozen using a controlled rate freezing of -1°C per minute to -130°C.

The cryopreserved MSC product bag is removed from the controlled rate freezer, placed on dry ice and quickly transferred to a designated rack space in the vapor phase of a liquid nitrogen product storage freezer. Product is cryopreserved in nitrogen vapor until all tests for release have been completed and it is shipped to the clinical site for administration to the patient.

A sentinel vial is frozen with the cryobag. The sentinel vial is used for realease testing of viability and immunophenotype anlysis after thawing.

For shipment, the final product bag is placed in vapor phase of liquid nitrogen in a validated cryoshipper with a data logger (temperature monitor). Transit times and internal temperatures will be retrieved from the data logger and the information reviewed by QA for qualification of the shipping procedures.

2.1.P.1.4 Controls of critical steps and intermediates

Please be referred to section 2.1.S.2.4

2.1.P.1.5 Process validation and/or evaluation

Successful qualification runs have been processed in the cleanroom at Officina Farmaceutica OPBG and Laboratorio Lanzani. Please refer to section 2.1.S.2.5.

Process monitoring during sterile filtration and aseptic filling comprises air samples, monitoring of surfaces, monitoring for presence of viables by settle plates and monitoring of non-viable particles. Routine monitoring (at rest) is also performed by testing samples for viable microorganisms and particles.

For a complete description of the manufacturing area and controls on particulate and microbiological contaminants, please refer to Section 2.1.A.

2.1.P.1.5.1 Validation of aseptic manufacturing process

The aseptic processing of parenterals is validated by performing media fills. To this purpose, the nutrient medium TSB is manipulated and exposed to operators, equipment, surfaces and environmental conditions to closely simulate the same exposure, which a product itself will undergo. Microbial growth in the filled vials is monitored.

The initial validation is performed on three consecutive media fills. Annual revalidation runs are conducted to evaluate the state of control of the aseptic process.

Media fill performance is regulated by internal policies in compliance with GMP regulation.

2.1.P.2 Control of excipients

2.1.P.2.1 Specifications

The resuspension medium is human AB plasma purchased from Octapharma and normally used for transfusion purposes.

It is screened to be negative for viral markers (HBsAg, Anti-HBc, HCV, Anti-HCV, HIV-1, Anti-HIV-1 and -2) and syphilis.

10% DMSO purchased from WAK- Chemie Medical GmbH is added to final product. It is sterile, pyrogen free, clinical grade material with endotoxin levels < 0.1 EU/ml, and no detectable mycoplasma contamination. Purity is 99.9%.

2.1.P.2.2 Excipients of human or animal origin

There is one human-derived excipient, human AB plasma.

2.1.P.3 Control of the Investigational Medicinal Product

2.1.P.3.1 Specifications

The test methods and acceptance criteria for MSC drug product are listed in table 12.

Table 12 Test methods and acceptance criteria

**TEST**	Specification
Sterility<br>(anaerobic microorganisms, EP 2.6.27)	Sterile
Sterility<br>(aerobic microorganisms, EP 2.6.27)	Sterile
Bacterial Endotoxins (EP 2.6.14)	≤ 5 E.U./mL
Mycoplasma (EP 2.6.7)	NEGATIVE
Karyotype	No metaphases or no chromosomic aberrations or non-clonal chromosomic aberrations in ≤10% of metaphases
Clonogenic assay	Negative
Viability	≥ 80%
Identity	$CD73^+ \geq 90\%$<br>$CD90^+ \geq 90\%$<br>$CD105^+ \geq 90\%$<br>$CD45^+ \leq 5\%$<br>$CD14^+ \leq 5\%$<br>$CD34^+ \leq 5\%$

2.1.P.3.2 Analytical procedures

See Section 2.1.S.4.2.1

2.1.P.3.3 Validation of Analytical procedures

The analytical procedures Sterility, Mycoplasma, Endotoxin used are all Ph.Eur. methods verified for the intended use.

The enumeration of CD73+, CD90+, CD105+, CD14+, CD34+, CD45+ using flow cytometry analysis is performed according to local SOPs.

2.1.P.3.4 Batch analyses of the final product

See Section 2.1.S.4.4

2.1.P.3.5 Characterization of impurities

See Section 2.1.S.3.2

2.1.P.3.6 Justification of the specifications

See Section 2.1.S.4.5 and 2.1.P.3.1

Viability:

At least 80% of the cells at harvest (pre-freeze) should be viable. A sentinel sample frozen with the product serves to establish the viability after storage and thawing of the product prior to shipment to a clinical site. The minimal threshold for viability of MSC after storage and thawing in the sentinel sample is set at 80%. This is based on historical data and will be re-evaluated once more batch release data will become available.

2.1.P.4 Reference standard

Not applicable

2.1.P.5 Container closure system

CryoMACS® Freezing Bags (Miltenyi Biotec): EVA (ethylene-vinyl-acetate) cryobags. EC Declaration of Conformity and Certificate of Analysis are annexed.

The freezing bag is equipped with EVA tubing for drawing Quality Control samples out of heat-sealed tube sections. The tubing is provided with luer connectors, roller clamp and a luer-lock injection port. In addition, each bag contains two spike ports for removal of the thawed cell product: the single-component ports are secured with sealed, twist-off protective caps which reduce the risk of nitrogen ingress into these ports.

2.1.P.6 Stability

A stability test of frozen BM-MSC maintained in liquid nitrogen for at least 5 years was performed in the Bergamo Facility. These studies have shown that the cells retain their viability (≥ 80%) and MSC markers expression (CD73, CD90, CD105≥90%). On the base of this stability study, the expiration date of the MSC is therefore currently established at 5 years from the time of freezing, if kept below -130°C.

Final product stability studies are ongoing in the facility in Rome to determine cell viability and cell recovery at different times from freezing. Vials of the validation batches produced by the manufacturing

process are set aside to monitor final product stability according to the stability plan reported in table 13.

Table 13 MSC stability evaluation after thawing

	Specification	T0	3M + 15 gg	6M + 15 gg	18M +1 M	36M +1 M
**VIABILITY**	≥ 80%	95.3	x	x	x	x
**CD73 Expression**	≥ 90%	99.4	x	x	x	x
**CD90 Expression**	≥ 90%	99.6	x	x	x	x
**CD105 Expression**	≥ 90%	99.8	x	x	x	x
**STERILITY**	sterile	NEG	x	-	x	x

Shipping does not affect the expiration timing, as the frozen storage and the dry shipper conditions are both nitrogen vapor phase.

A temperature profile is generated for each shipment that shows whether any excursions were experienced.

2.1.A Appendices

2.1.A.1 Facilities and equipment

The Officina Farmaceutica Ospedale Pediatrico Bambino Gesù in Rome (Italy) is an academic GMP facility devoted exclusively to production of investigational ATMPs for human therapeutic trials. The design, construction, and validation of the GMP facility (Production and Quality Control areas) was reviewed by the Italian medicine agency AIFA to assure full compliance with all current GMPs. The Officina Farmaceutica Ospedale Pediatrico Bambino Gesù has been certified by the Italian medicine agency AIFA, according to D.L.vo 211/2003, for the production of sterile medicinal products, of cell based and gene therapy medicinal products, and for secondary packaging operations all in compliance with GMP regulations.

L'officina farmaceutica Laboratorio Lanzani is an academic GMP facility authorized to produce investigational ATMPs for human therapeutic trials since 2008. The design, construction, and validation of the GMP production facility has been reviewed by AIFA in 6 separate inspections to assure full compliance with all current GMPs. The Laboratorio Lanzani has been certified by the Italian medicine agency AIFA, according to D.L.vo 211/2003 and D.L.vo 200/2007, for the production of sterile cell based medicinal products of small volume, for secondary packaging operations and biological quality control tests, all in compliance with GMP regulations.

The Production Laboratory for MSC in Laboratorio Lanzani is a dedicated class B laboratory complete with class A laminar cabinet, CO2 incubators and a centrifuge for processing by dedicated and trained production operators. The quality controls are performed in dedicated and separate areas, by specific and separate QC operators. A separate storage area for materials is avalaible as well as specific storage area for frozen ATMP (liquid nitrogen tank dedicated to storage of ATMPs in nitrogen vapor). Spaces, equipment, personnel, environmental controls etc are performed in accordance to GMP regulation. The Cell Factory in Bergamo has been repeatedly visited by AIFA inspectors and has not been structurally modified in the last 9 years. In addition is has produced MSCs for several clinical trials (see Ref). The details of the Begamo Cell Factory will therefore not be described in further detail below.

More specifically, the classified areas include the two laboratories TC1 and TC2, in class B enviroment for the production of ATMPs in open systems, stained in dark green in Fig 3, one laboratory in class C (TC3) for the development and production of ATMPs in closed systems (bioreactors) indicated in light green in Fig 3 and one laboratory in class C (TC4) for the manipulation of haematopoietic stem cells (HSC) for the transplant program (laboratory TC4 in light blue in Fig 3).

Inside each room there are sterile hoods reaching class A enviroment under vertical flow as well as refrigerated centrifuges, refrigerators and CO2 incubators at 37°C. All equipment has been installed according to IQ, OQ, PQ specifications, is alarmed and is periodically controlled according to validation plans.

Furthermore, the Laboratory contains one non classified area, including the rooms for the quality controls indicated in purple in Fig 6A, passages and storage rooms (yellow), freezing and storage of ATMPs (indicated in pink) and haematopoietic stem cells in distinct liquid nitrogen tanks (white in Fig 3).

In a separated floor, there are several officies and meeting rooms as indicated in Fig 4.

The different levels of atmospheric pressure are indicated schematically in figure 5 as well as the position of the absolute filters located in the ceilings of different rooms as schematically indicated in figure 6.

Biological materials, reagents and other materials follow the fluxes as indicated in Fig 7, also showing of the exit passages for the ATMPs produced. In particular there are indicated five separated pass boxes with air flow and UV sterilization for the passages of material between different compartments of the structure.

Similarly, the pathways for proper entering and dressing as well as leaving of the operators is indicated in figure 8; the opening of the doors is controlled by automatic system to monitor each operator via Wingaep software and data are directly registered in the batch record files.

Figure 3 Annex 6A

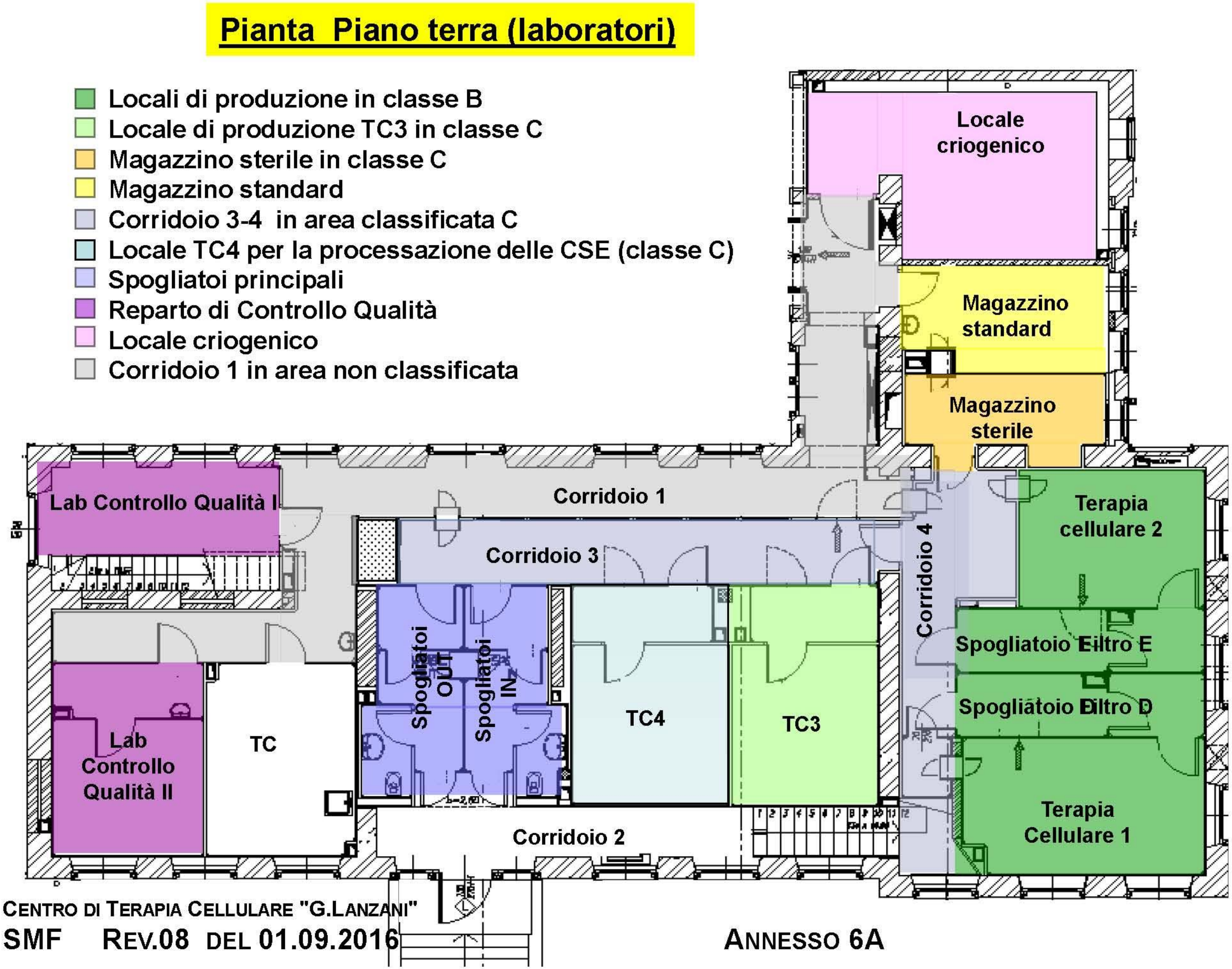

Figure 4 Annex 6B

Pianta Zona uffici primo piano

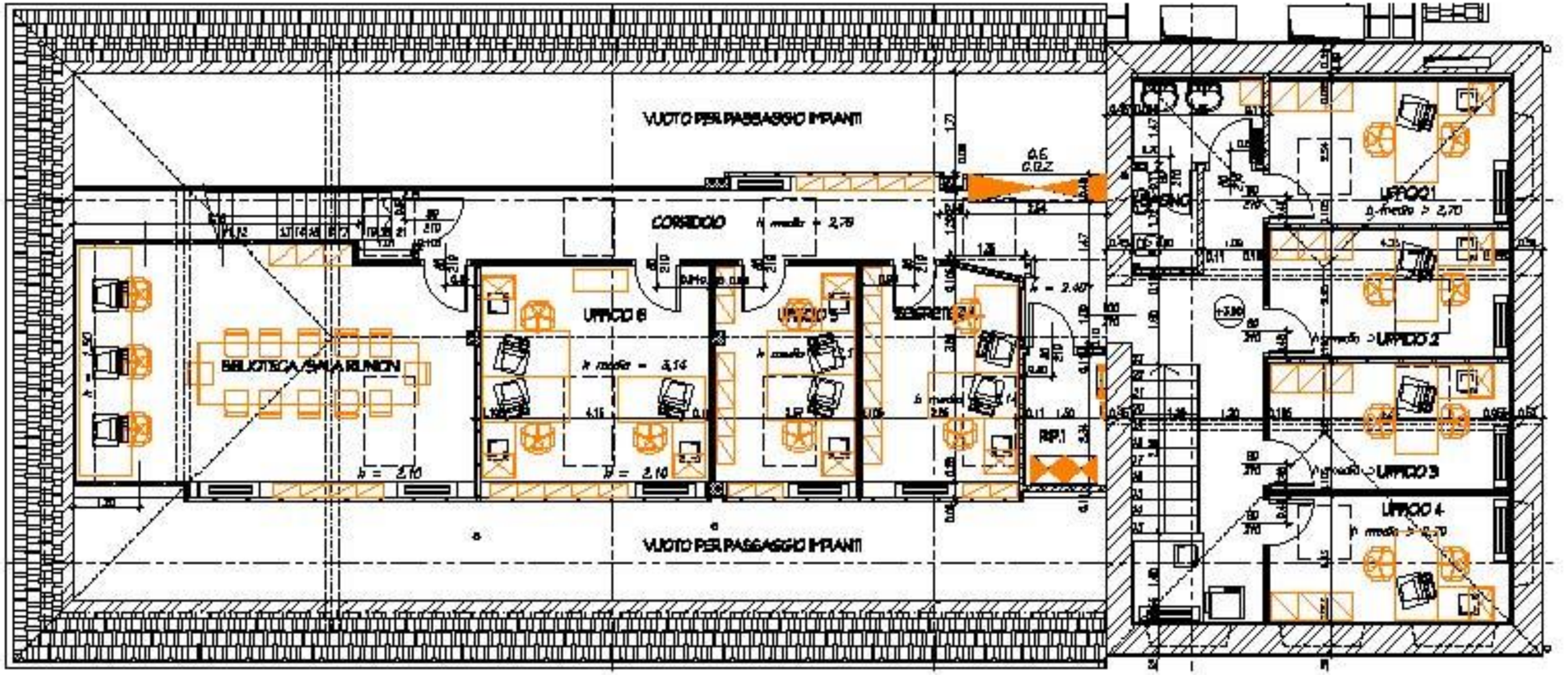

CENTRO DI TERAPIA CELLULARE "G.LANZANI"

SMF REV.08 DEL 01.09.2016 **ANNESSO 6B**

Figure 5 Annex 6C

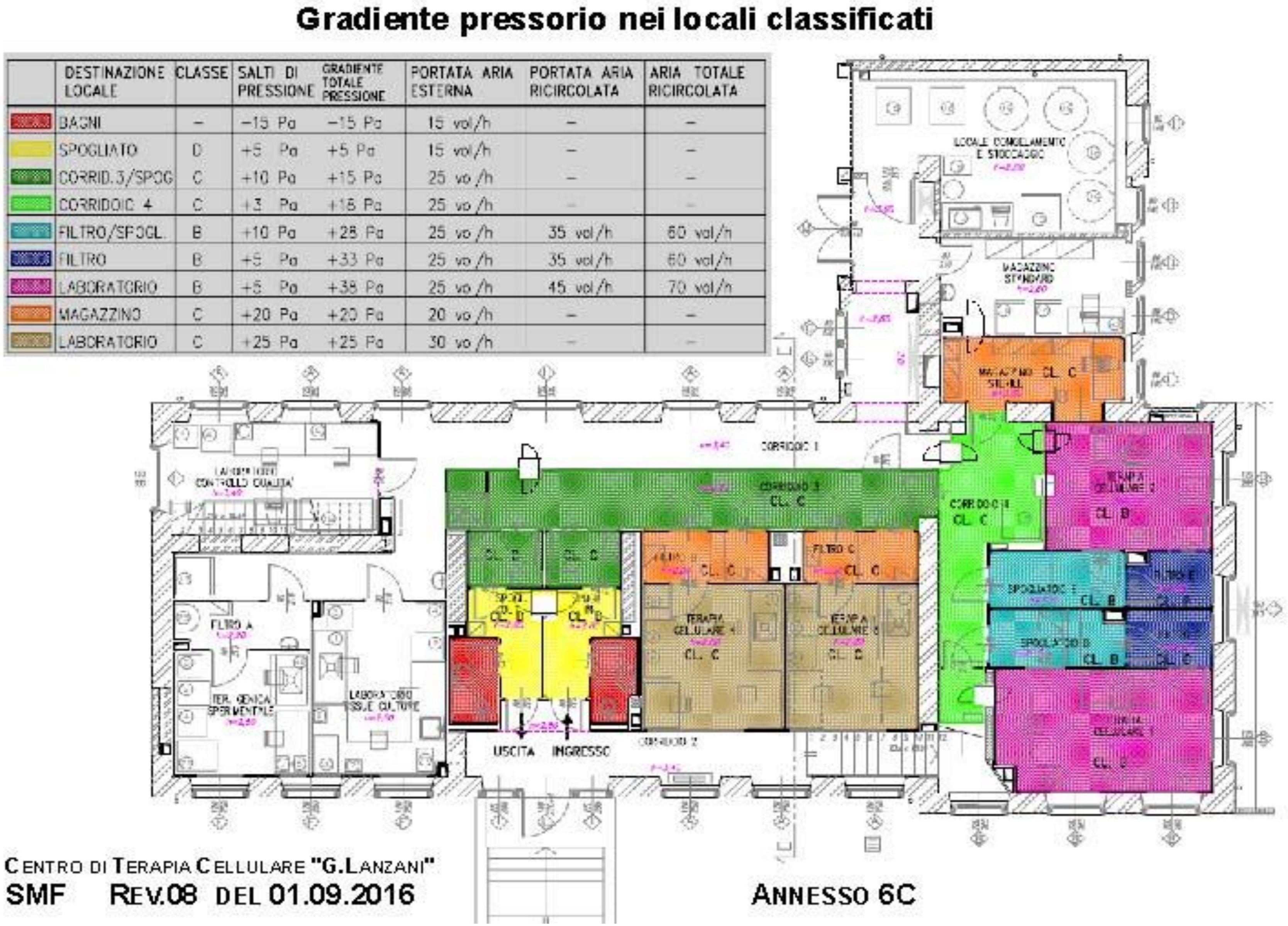

	DESTINAZIONE LOCALE	CLASSE	SALTI DI PRESSIONE	GRADIENTE TOTALE PRESSIONE	PORTATA ARIA ESTERNA	PORTATA ARIA RICIRCOLATA	ARIA TOTALE RICIRCOLATA
(red)	BAGNI	–	−15 Pa	−15 Pa	15 vol/h	–	–
(yellow)	SPOGLIATO	D	+5 Pa	+5 Pa	15 vol/h	–	–
(dark green)	CORRID.3/SPOG	C	+10 Pa	+15 Pa	25 vol/h	–	–
(green)	CORRIDOIO 4	C	+3 Pa	+18 Pa	25 vol/h	–	–
(cyan)	FILTRO/SPOGL.	B	+10 Pa	+28 Pa	25 vol/h	35 vol/h	60 vol/h
(blue)	FILTRO	B	+5 Pa	+33 Pa	25 vol/h	35 vol/h	60 vol/h
(magenta)	LABORATORIO	B	+5 Pa	+38 Pa	25 vol/h	45 vol/h	70 vol/h
(orange)	MAGAZZINO	C	+20 Pa	+20 Pa	20 vol/h	–	–
(brown)	LABORATORIO	C	+25 Pa	+25 Pa	30 vol/h	–	–

Figure 6 Annex 6D

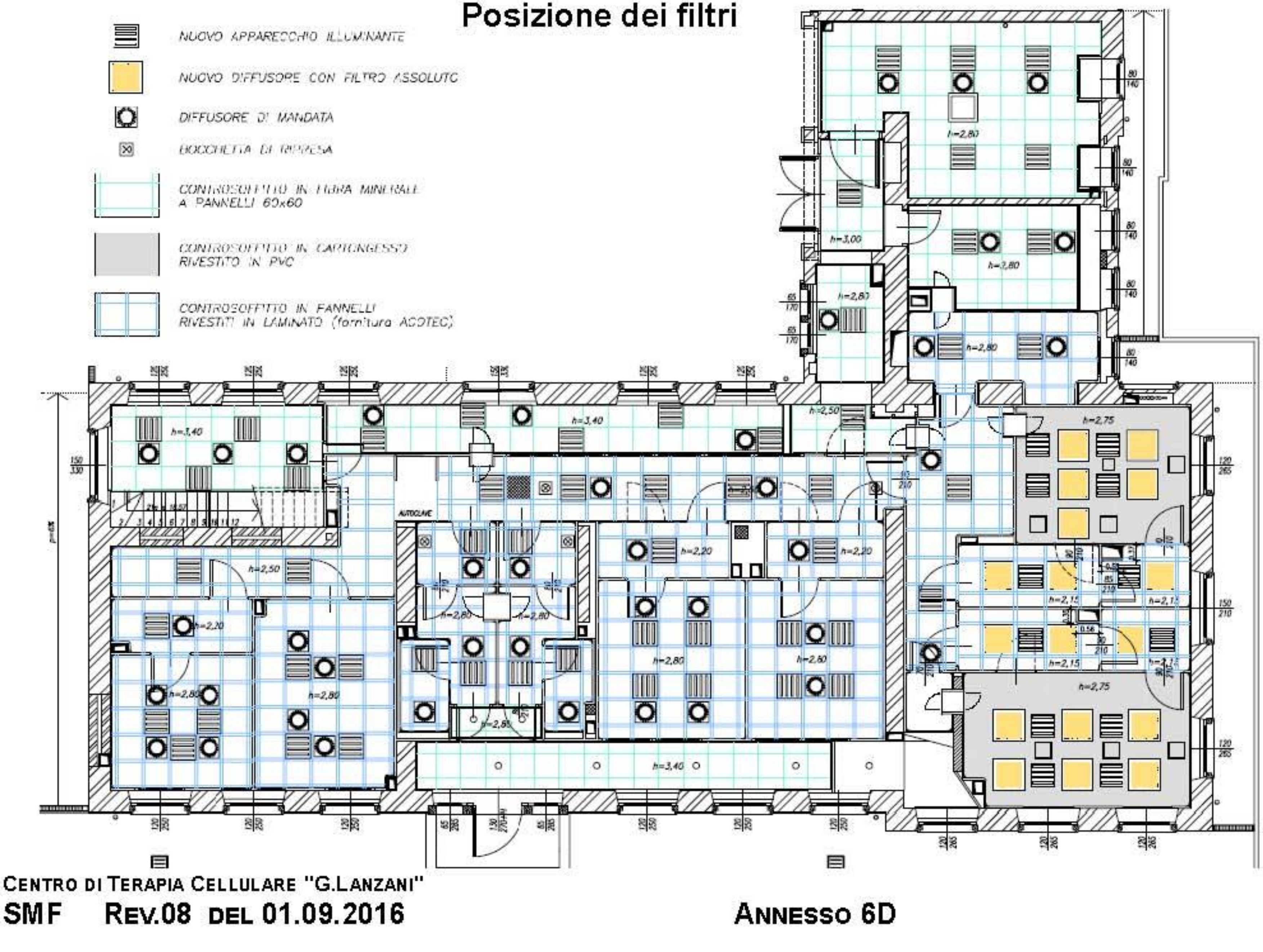

Figure 7 Annex 6E

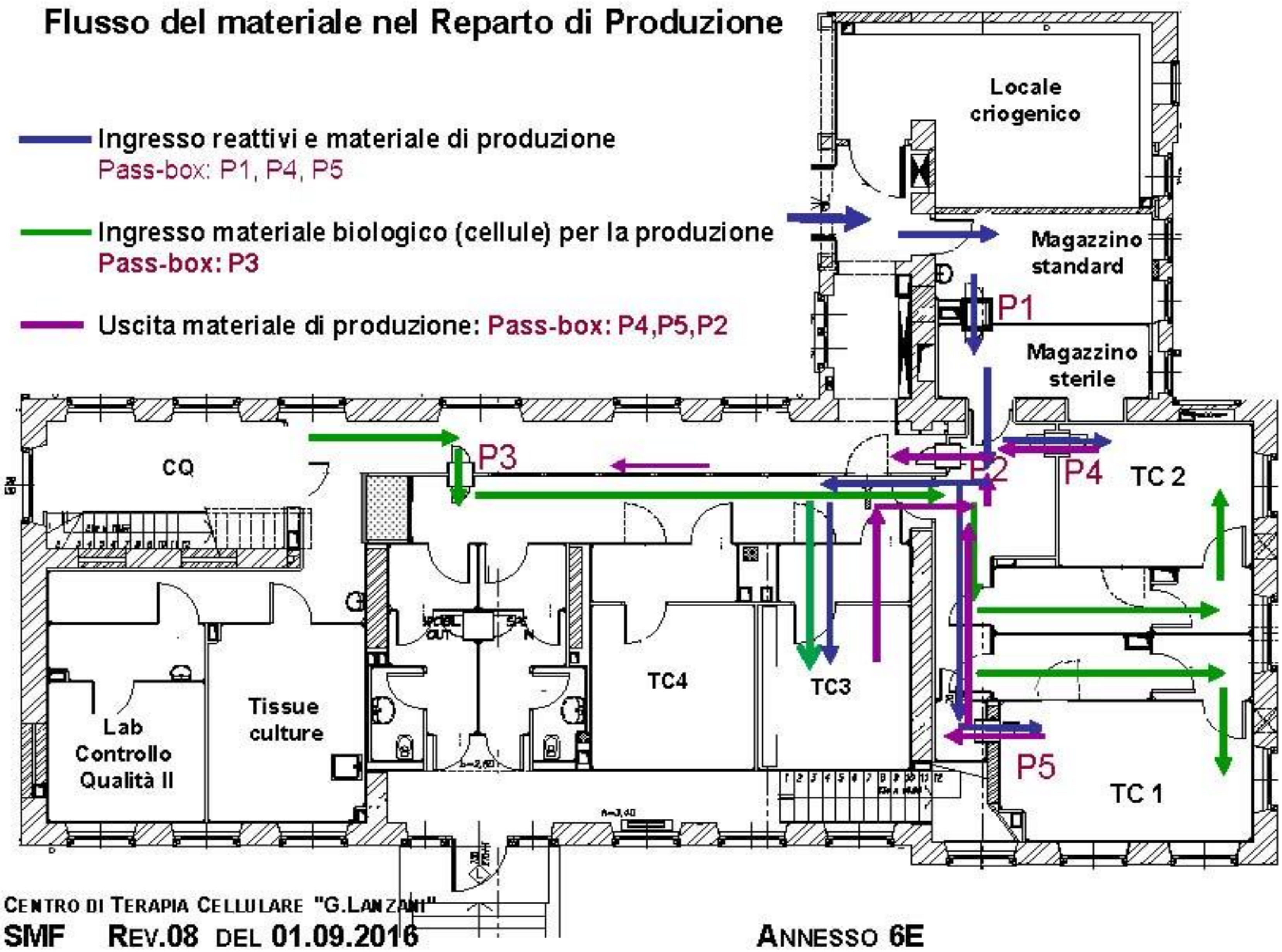

Figure 8 Annex 6F

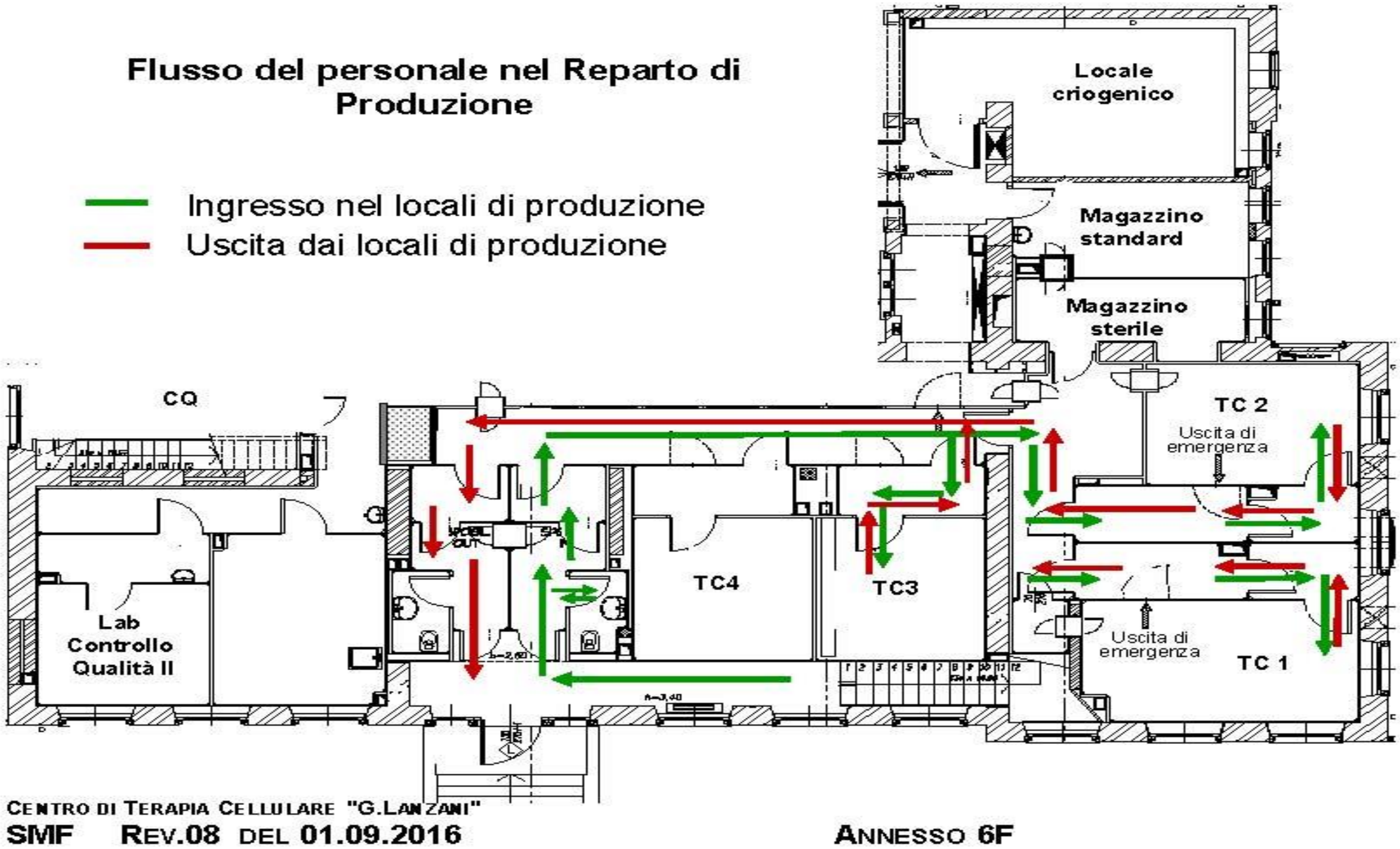

2.1.A.1.1 Description of the facilities and equipment

OPBG GMP Facility is located on the ground floor of the San Paolo Research Facility of the Ospedale Pediatrico Bambino Gesù. The structure covers an area of about 980 sqm composed of 4 different areas categorized as following:

1. production area according to GMP standards;
2. Quality Control laboratories;
3. area dedicated to receiving goods and warehouse;
4. staff offices.

In particular, production area of about 400 square meters consists of D, C and B grade lockers or dressing rooms, B and C grade production areas and Grade C shared areas (corridors and supporting rooms). The areas dedicated to the production of gene and cell therapy are separated.

The GMP facility is built with suitable materials (PVC, melamine resin, stainless steel, glass).

Floors and walls are smooth and without split, they are continuously connected to make cleanliness operations easy and avoid dust sediment. The coating materials (for example, PVC) are resistant to the action of chemical disinfectants.

Access to the classified area is controlled by means of a badge reader and is done by appropriate dressing, according to gowning procedure.

The HVAC (Heating, Ventilation and Air-Conditioning) system is designed to provide air class A, B, C and D, according to EU GMP Annex I and, in order to avoid cross-contamination, is based on the MAKE-UP + UTA principle.

Air enters the manufacturing area and is extracted through HEPA filters. In particular, each room has a terminal HEPA filter (H14) on false ceiling.

The make-up unit supplies air to the 14 independent sub-systems (UTAs). Each production unit (manufacturing lab, changing room, pre- changing room and material pass through) is managed by a single UTA that treats both fresh and recirculated air (about 70% of each unit). There is not recirculating air from biohazard cabinet and pass through extraction.

For handling of the product, several biohazard laminar flow generating unidirectional air flow cabinets, complying with class A, are involved.

The HVAC system layout is reported in Figure 9

Figure 9: HVAC system layout

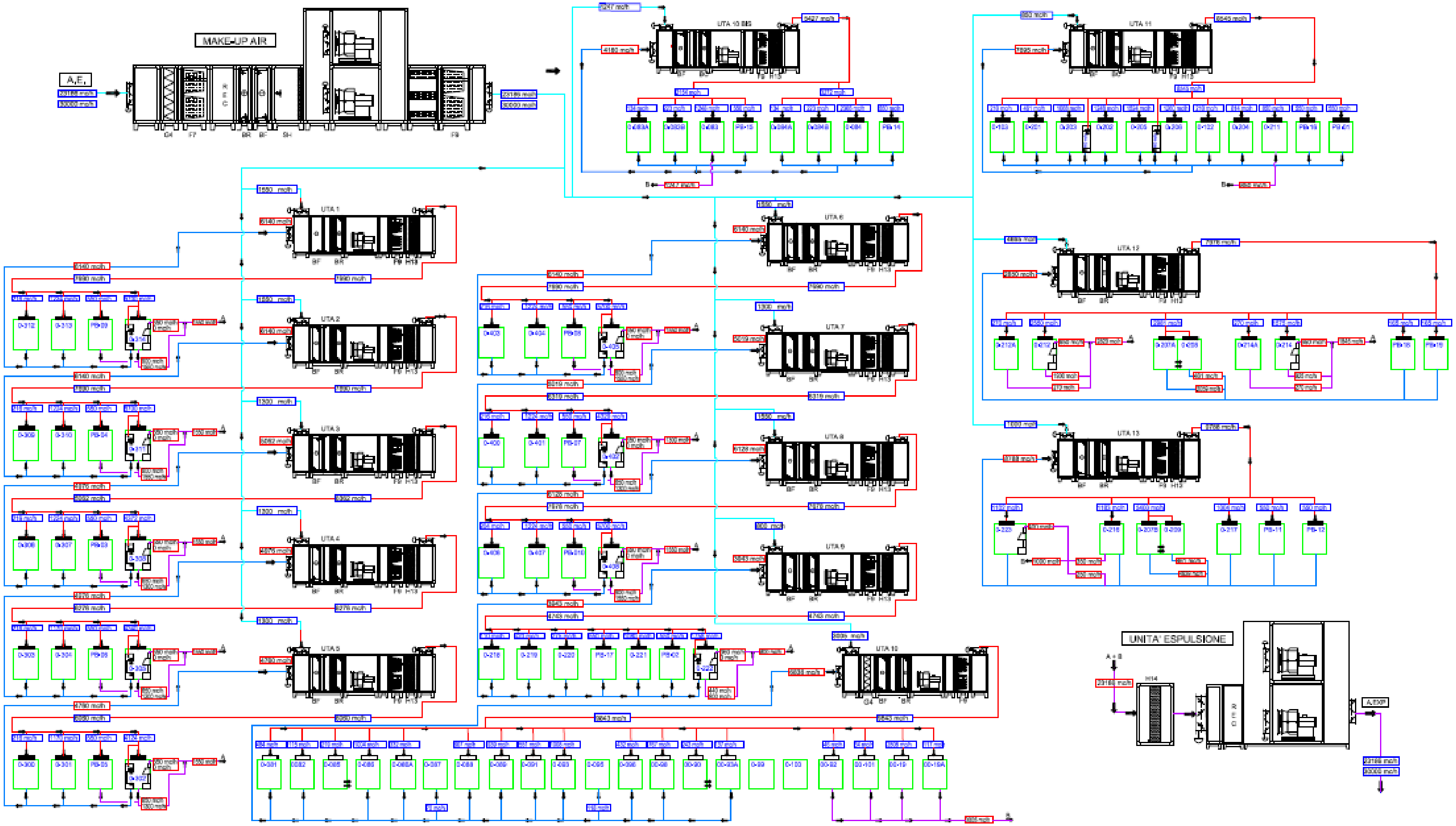

2.1.A.1.1.1 Description of the production area

Manufacturing area for OPBG is composed of gene and cell therapy separated zones, set up with the appropriate manufacturing equipment according to the manufacturing process to produce the requested products.

All the manufacturing operations are defined into approved Batch Records and handled by trained personnel following pre-defined instructions. The received products and materials are inspected, to verify their identity, quantity and agreement against Batch Record instructions. All the process controls are registered in the Batch Record and, at the end of production, after sampling for analysis, the products are stored inside GMP product store area, according appropriate storage conditions (freezers -80°C and vapor phase nitrogen).

All the people working in GMP production areas are specifically trained, must behave properly and must give special care to personal hygiene in accordance to specific SOPs. In addition, GMP production operators are periodically retrained (according to annual training master plan) in order to ensure the proper execution of planned manufacturing activities.

At the end of every manufacturing working session in sterile areas, operators are monitored for microbial contamination by means of contact plates taken from different parts of the body, according to written SOPs in compliance with GMPs requirements.

All the requirements in terms of cleaning, hygiene and aseptic conditions for the manufacturing of sterile products are satisfied and in compliance with the GMP requirements for the specific classification of the rooms.

Production unit is also responsible for the transfer of new developed processes and for the qualification/ validation of manufacturing processes also in terms of sterility assurance (media fill runs).

The production area is also authorized for the MOGM treatment in accordance to the Law Decree 206/01.

The production area dedicated to cell therapy process consists of:

- N.3 Grade B labs (0-302, 0-305, 0-402) of approximately 15 sqm with accessories rooms (Class C pre-B dressing and Class B dressing) of approximately 5 sqm;
- N.2 Grade B labs (0-405, 0-408) of approximately 21 sqm with accessories rooms (Class C pre-B dressing and Class B dressing) of about 5 sqm;
- N. 1 Grade C lab dedicated to the processing of oncoematological transplant products under laminar flow hood space.

The production area dedicated to gene therapy process consists of:

- N.1 Grade B lab (0-308) of approximately 15 sqm with accessories rooms (Class C pre-B dressing and Class B dressing) of approximately 5 sqm;
- N.2 Grade B labs (0-311, 0-314) of approximately 21 sqm with accessories rooms (Class C pre-B dressing and Class B dressing) of about 5 sqm;
- N.1 Grade C lab lab (upstream lab) dedicated to vectors production under laminar flow hood space. Also one grade C room is present for personnel gowning;
- N.1 Grade C lab (downstream lab) dedicated to vectors purification. Also one grade C room is present for personnel gowning.

Every grade B production lab is equipped with class A biosafety laminar air flow cabinet and also includes one grade B pass through, one grade C and one grade B gowning premises.

Personnel enters and leaves the manufacturing area through a locker system.

There are separate material/personnel entry air lock. Only sterile materials are handled in manufacturing labs and all materials enter and leave the area by using dedicated pass through.

All the production areas are qualified both for viable and non viable particles in "at rest" and "in operation" conditions.

Clean zones within the manufacturing area also include supporting area as:

- N. 1 Grade C picking warehouse for the temporary storage of reagent/media at +5°C, -20° C and -80° C;
- N. 1 Grade C room used to manage material for sterilization by autoclave;
- N. 2 Grade C rooms (one for the cell therapy area and one for the gene therapy area) to store "in use" cleaning material;
- N.1 room dedicated to freezing and thawing of cellular material;
- N.2 grade C dressers (one for male and one for female staff) for access to common areas of grade C (corridors);
- N.2 grade C (one for male and one for female staff) for the exit from the common areas of grade C (corridors);
- N.2 grade D lockers for access / exit to / from C grade lockers (one for male and one for female staff).

The GMP production areas are qualified and monitored in accordance to Annex I of GMP. In Table 1 are summarizes the classified areas and their function in the GMP OPBG facility.

Table 14 Function of different class areas

Clas s	Description and function of the area
A	Laminar flow hood used for the sterile manipulation of products and intermediates placed in class B Area
B	Class A background area; third step dressing rooms, laminar air flow located in the C class rooms (in terms of microbiological limits)
C	Class B background area, area used for the production of vector- Supernatant and common rooms/corridors; purification area; second step dressing rooms
D	First step dressing rooms

The access to the production areas is controlled by airlocks that allow the separation of the different classified areas through over pressure systems that guarantee the classification of the areas.

Regarding cell therapy area, over pressure system consists of:

- Grade B lab with a ΔP value of 50 ± 5Pa;
- adjacent Grade B lockers with a ΔP value of 45 ± 5Pa;
- Grade C pre dresses with a ΔP value of 30 ± 5Pa;
- Grade C cell therapy corridor in front of the pre-dresser with a ΔP value of 30 ± 5Pa.

For the restriction of MOGMs, gene therapy area differs from those of cellular therapy in terms of □P between the production room, the adjacent grade B dressing room, the C grade pre-dressing room and gene therapy corridor.

The over pressure system implemented on gene therapy area consist of

- Grade B lab with a □P value of 35 ± 5Pa;
- adjacent Grade B locker with a □P value of 50 ± 5Pa;
- Grade C pre dressing room with a □P value of 25 ± 5Pa;
- Grade C gene therapy corridor in front of the pre-dressing room with a □P value of 30 ± 5Pa

Such differential pressures guarantee classifications of premises related to the conditions set out in Annex 1, vol. 4 GMP.

An overview of classified manufacturing area, of the differential pressures, of the materials and personnel flow are given in Figure 8, Figure 9, Figure 10 and Figure 11.

Figure 10 Rooms classification

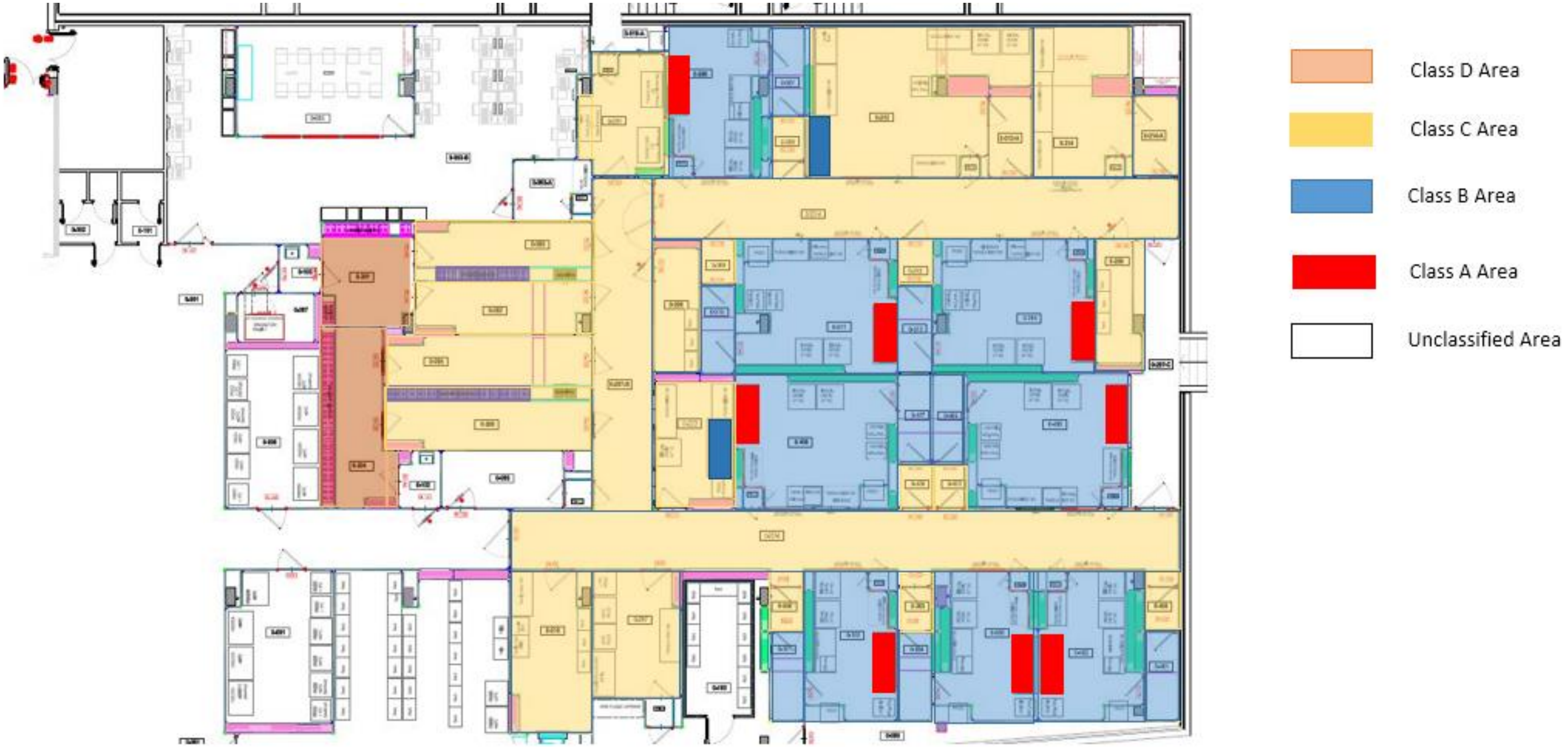

Figure 11 Over pressure systems

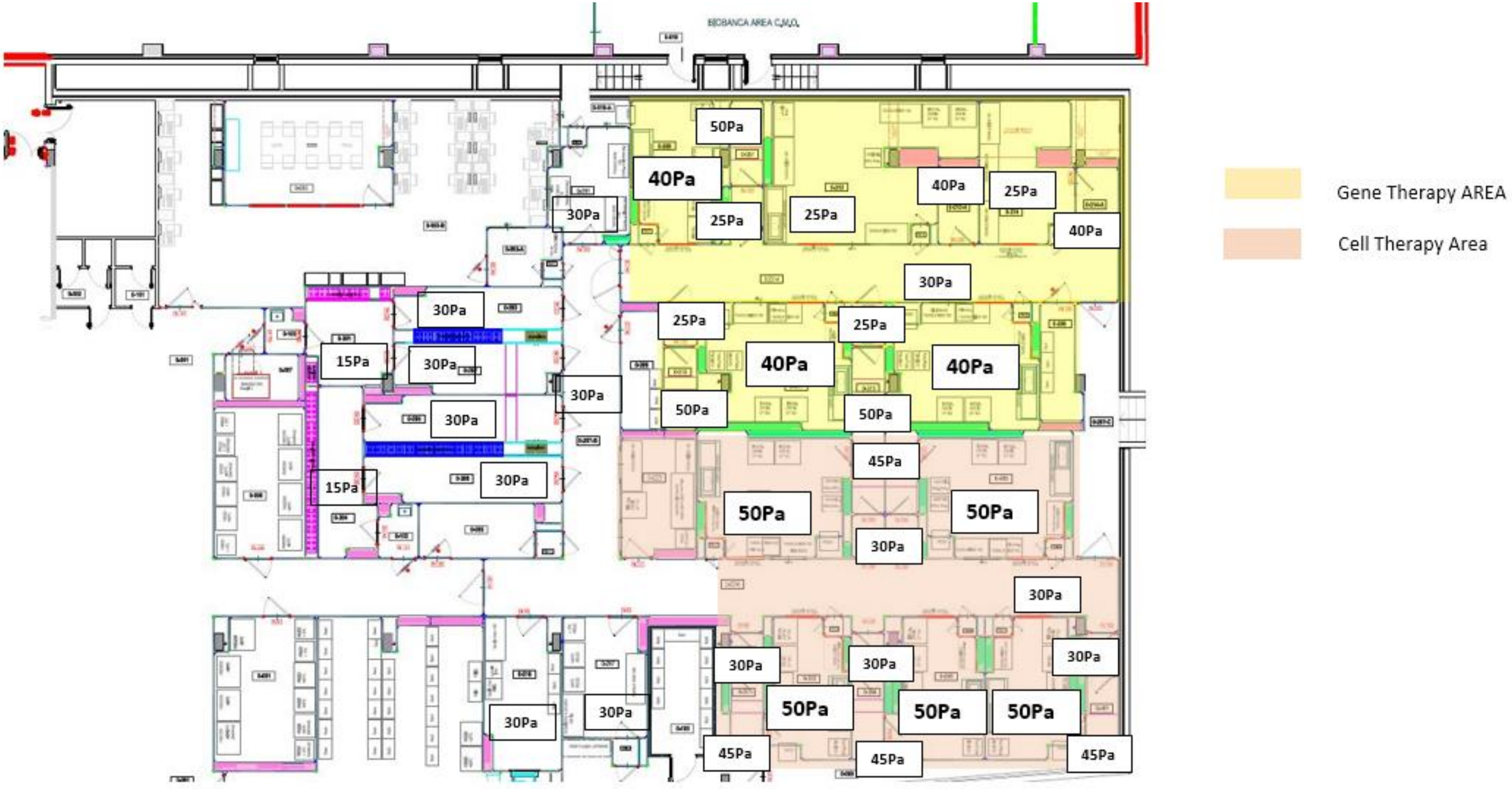

Figure 12 Material flow diagram

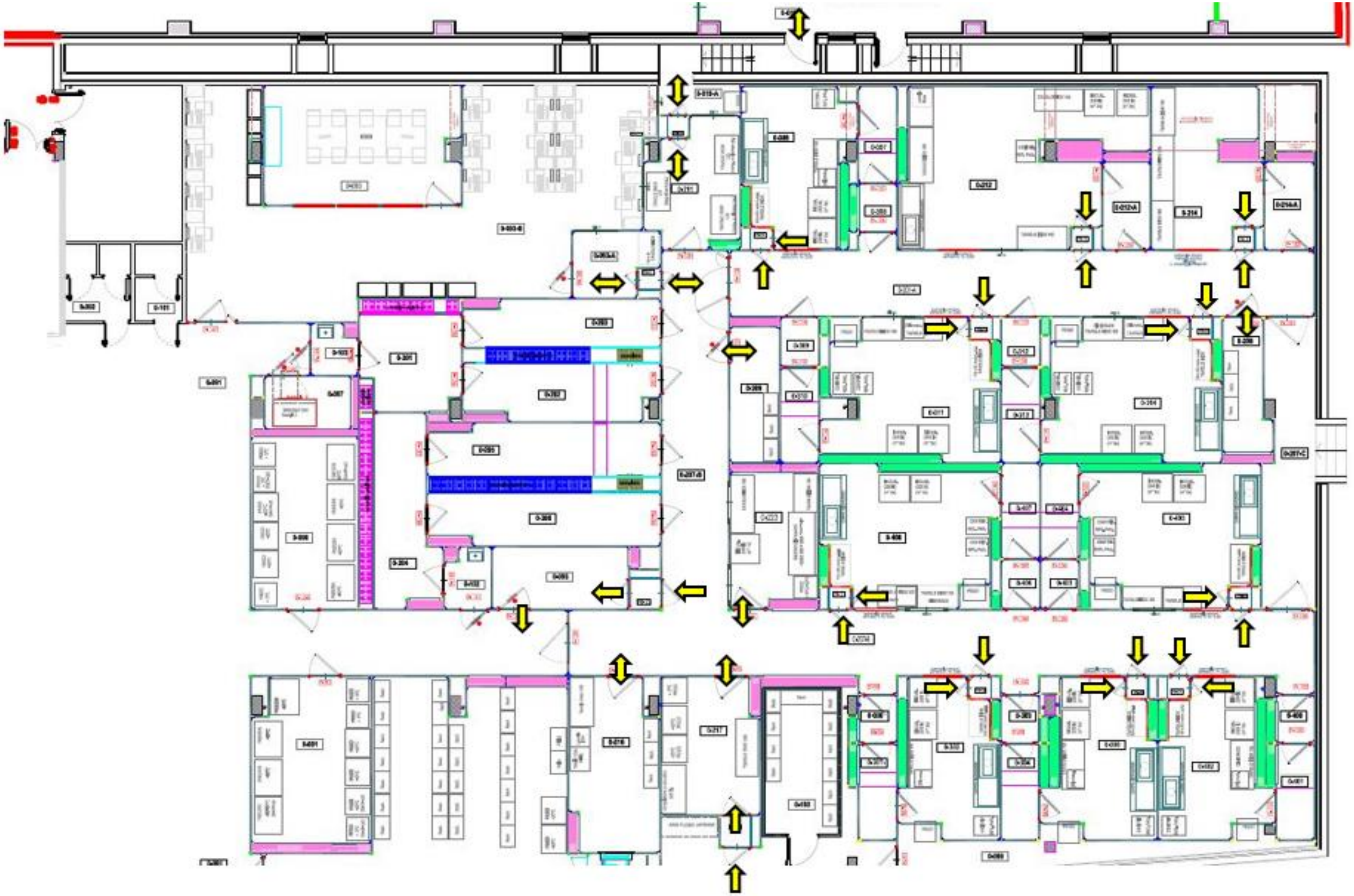

Figure 13 Personnel Flow diagram

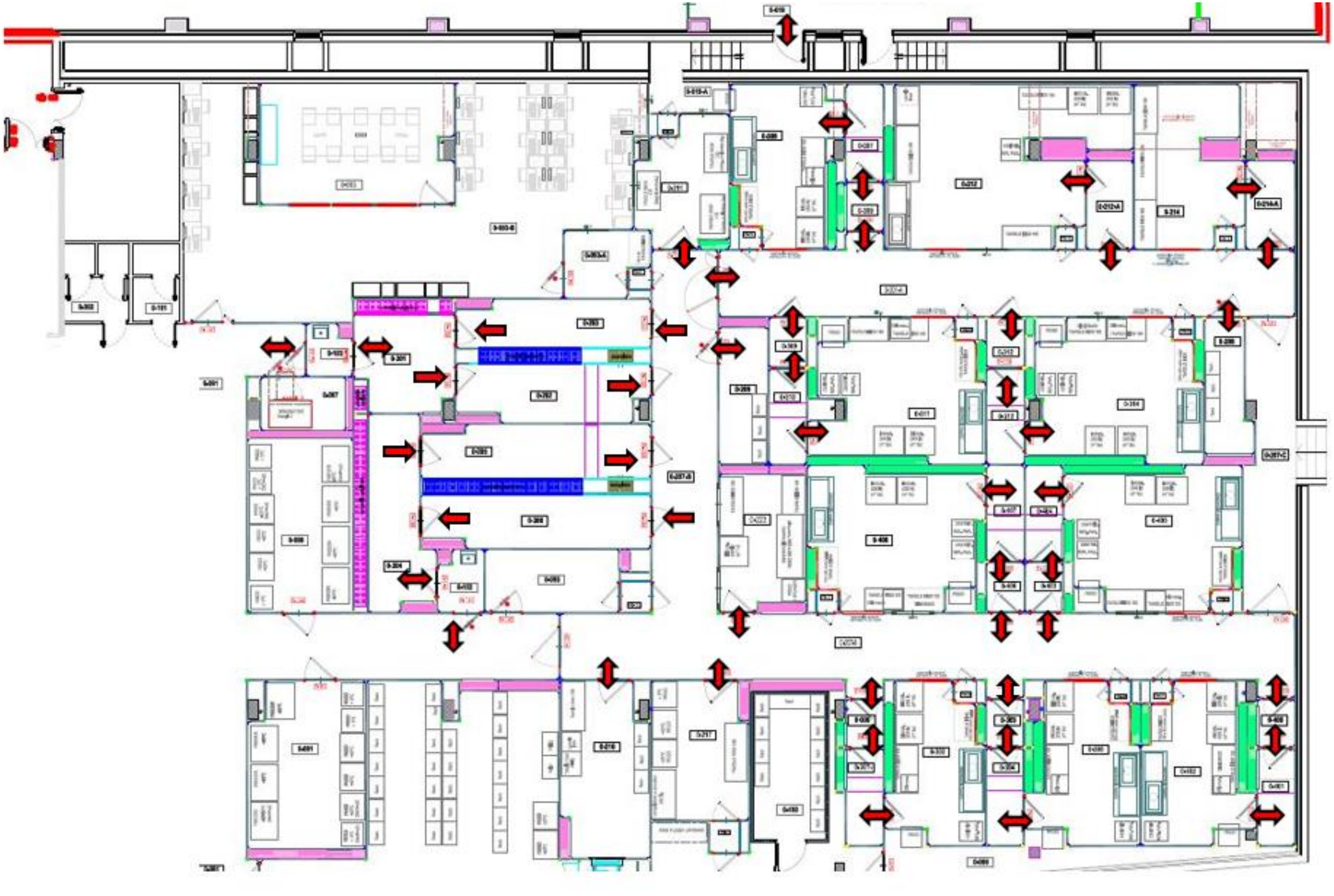

The cleaning activities for the rooms and the working stations are regulated by specific procedures that define how to perform the activities, the chemical agents to be used and the timings.

The cleaning system has been validated from a microbiological point of view.

Viable and non viable particles periodical monitoring plans have been defined for the production areas in order to ensure the maintenance of the classification.

All the production equipment are fixed and dedicated to a room.

Equipment/ systems available for use in the GMP processing includes the following:

- LAF biohazard hoods located in each Grade C and Grade B manufacturing room;
- Centrifuges located in each Grade C and Grade B manufacturing room;
- Incubators located in each Grade C and Grade B manufacturing room;
- Microscopes located in each Grade C and Grade B manufacturing room;
- Pipet-Aid located in each Grade C and Grade B manufacturing room;
- Clinimacs Plus located in 2 Grade B manufacturing room;
- Clinimacs Prodigy located in 1 Grade B and 1 Grade C manufacturing room;
- Refrigerators +5°C located in each Grade B manufacturing room;
- Refrigerators +5° located in Grade C picking warehouse for the temporary storage of reagent/ media;
- Freezers -20°C located in Grade C picking warehouse for the temporary storage of reagent/ media;
- Freezers -80°C located in Grade C picking warehouse for the temporary storage of reagent/ media;
- Autoclave;
- Controlled rate freezer, planer;
- Vapor phase nitrogen containers used for the storage of intermediates/ products;
- All equipment involved in cGMP activities, has been validated through the IQ, OQ e PQ phases.

All equipment involved in cGMP activities, has been validated through the IQ, OQ and PQ phases.

The validation protocols and reports are approved by the Quality Assurance manager.

For each instruments, a preventive maintenance and calibration program has been established and recorded in proper logbooks.

2.1.A.1.1.2 Description of the quality control area

QC laboratories for OPBG are dedicated to perform testing on intermediates, products and IPC in accordance to the product specification file in which the details of all required samples, quantity, specifications, sub-contractors, labels and retains are reported. Also testing on raw materials are performed in accordance to specific raw material specification documents which specify the sampling and testing requirements and specifications. QC Units carriers out the enviromental controls (both viable and non viable particles) of the classified areas of the GMP facility.

Moreover, QC laboratories perform the following activities:

- stability studies, in accordance to specific protocols and procedures. The data are trended and reviewed on a regular basis and OOS formally investigated in accordance to the procedure for NCA and OOS management;
- incubation of TSB used for the validation of the aseptic process by media fill runs and of the TSA plates used for the monitoring of the manufacturing facility;
- development, transfer and validation of analytical methods in accordance to specific protocols and procedures. The Quality Control area is approximately 280 square meters, is physically segregated from the production area and includes classified and unclassified rooms.

QC unclassified area is subdivided in the following 5 areas, all of them involved in the testing of raw materials, IPC, intermediates and finished DP and also in the development and validation of the analytical assays to be used:

Molecular Biology, used for safety test (as VCN and Mycoplasma tests) of intermediates, DS and DP by molecular biology analytical techniques such as RT-PCR.

Cell Biology: for the safety, identity and efficacy testing of raw materials, intermediates, DS and DP by cellular biology analytical techniques (such as cell cultures to test viability, co-colture to test product potency and functionality, transduction efficiency of viral vectors).

Microbiology: for the safety testing of raw materials, intermediates, DS and DP by microbiological analytical techniques (such as sterility by using Bact/Alert) and for the incubation of TSB used during the media fill runs and of the plates collected during the viable environmental monitoring.

Biochemistry: for investigation of phisical parameters such as pH.

Cytofluorimetry: for the identity and efficacy (functionality) testing of intermediates, DS and DP by cytofluorimetric analytical techniques.

In accordance to EU Ph 9, Molecular Biology area consist of subdivided comparments as:

- Master Mix area (area where exclusively template free material is handled);
- Pre-PCR (area where reagents, samples and controls are handled);
- PCR amplification;
- Post-PCR detection (area where amplified material is handled).

Segregated classified QC area is consistent with the activities carried out and consist of :

- Class B lab equipped with class A biosafety cabinet, dedicated exclusively to perform sterility test, adjacent Grade B locker, Grade C and D dressing rooms;
- Class D lab dedicated to microbial strain storage and handling;
- Class D lab dedicated to incubate TSB medium and TSA plates.

Equipment used in the QC laboratories is controlled, qualified, maintained and calibrated in accordance to internal specific procedures. The type of QC equipment is below reported:

- Biohazard LAF hood;
- Molecular biology hood;
- Incubators;

- Thermostat;
- Centrifuges;
- Microscope;
- Cytofluorimeter;
- PCR instrument;
- Real-Time-PCR;
- ELISA Reader;
- ChemiDoc;
- BactAlert;
- Portable non viable counters;
- Portable viable counters;
- Scales;
- Refrigerators +5°C;
- Freezers -20°C;
- Freezers -80°C

Personnel involved of QC laboratories are trained in techniques, analytical methods and systems. Analytical results are checked and reported either on laboratory notebooks and in certificates of analysis.

The QC samples are stored in freezers and refrigerators continuously monitored using calibrated probes connected to Desigo. An alarm is set off if the temperature falls outside the ranges and it is connected to a centralized control system (Desigo), which is supervised 24 hours a day, 7/7.

The access to the QC classified and sample store areas is restricted and controlled by swipe card access system.

2.1.A.1.1.3 Description of the warehouse and GMP product storage area

Warehouse Unit in OPBG is responsible for the management of incoming materials and their storage in appropriate conditions (such as room temperature, +5°C, -20°C or -80°C). The temperature of the cold room and freezers is continuously monitored using calibrated probes connected to the monitoring system Desigo.

All incoming batches of raw materials (media/reagents) and packaging materials are received in a dedicated area (receiving goods area) where they are identified by a OPBG batch number and taken in charge by the warehouse. In the warehouse there are dedicated areas for the materials in quarantine status and release status.

Rejected materials are stored in a segregated area of the warehouse until they are returned to the supplier or destroyed.

Once stored, the status of material are clearly identified by the use of appropriate labels that allow the immediate identification of the relevant status (yellow for quarantine status, green for released status and red for rejected status).

All materials received at the facility are logged into quarantine status until the conformity verification to detailed raw material release specifications. Once all the test performed for raw materials release have come successfully, the material is accepted for release, the manufacturer’s certificate of analysis is attached to release documentation and the item are relabeled with a green sticker reporting materials information.

The access to the warehouse areas is via personnel doors. Entry is restricted and controlled by keys that are managed only by the Logistic manager and the warehouse operator.

Desigo also monitors physical room conditions of warehouse and product GMP storage.

The GMP product store is located in the areas represented in the layout in Figure 12

Figure 14 warehouse and GMP product storage area

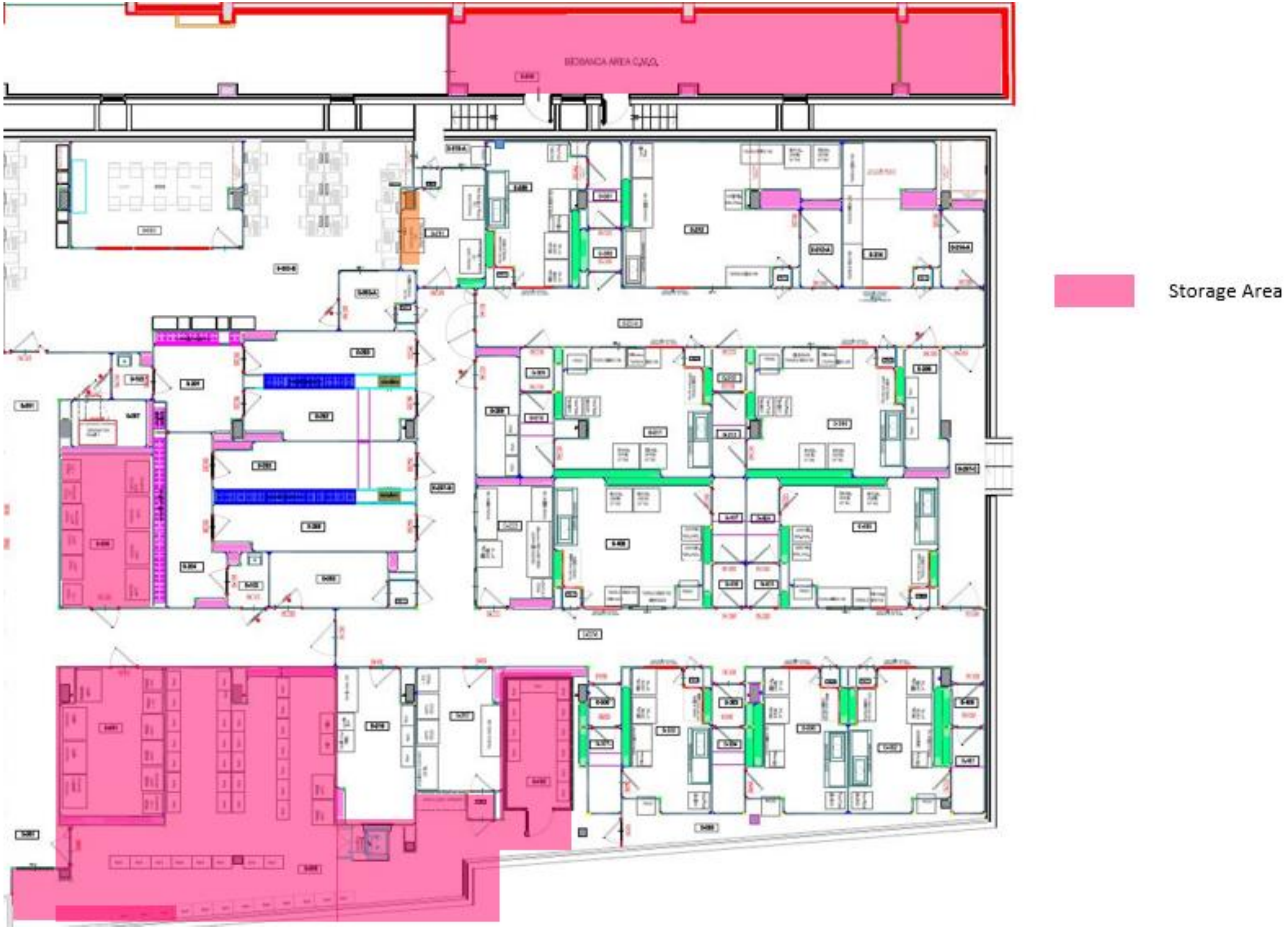

The storage conditions required for the different type of products are freezer al -80°C (vectors) or vapor phase nitrogen (viable cells) where the product is introduced after a first step freezing by using controlled rate freezer, planer.

The temperature of the freezers and vapor phase nitrogen containers is continuously monitored using calibrated probes connected to Desigo monitoring system which is supervised H24 7/7.

The access to the product storage areas is restricted and controlled by keys.

Warehouse Unit is also responsible for the shipment of products interacting directly with the couriers, accordingly to specific SOP. In order to verify the compliance to product specifications in terms of temperature, data logger is employed during the shipping to record temperature itself.

2.1.A.1.1.4 Environmental cleaning and monitoring

Routine cleaning of the classified areas of OPBG by means of suitable disinfectants is detailed described in internal SOPs. This procedure described the kind of chemical agents to be used and frequency of cleaning. Anti microbial efficiency of the cleaning products and of the cleaning protocol has been formally validated.

Daily working cleaning of aseptic rooms with hydrogen peroxide 6% involves wiping of benches, tables and internal pass-through shelves. Moreover, once a week also wiping of external surfaces of the equipment and mopping of floor were executed. At the end of daily working session, also internal work surface of cabinet laminar flow was sanitized.

At the end of a batch production, a full and complete clean is carried out using before chlorine and then sterile IPA 70/30. Full clean includes walls, floor, false ceiling, external and internal equipment surfaces, benches and tables.

Classified areas (GMP production and Quality Control) are periodically monitored in terms of viable and non-viable particles in accordance to EU guideline Annex I.

Routine monitoring (at rest and in operation) is performed by measuring and by taking samples for both viable and non-viable particles.

Environmental monitoring comprises both air samples and settle plates for microbiological monitoring and aerotrack particle counter for non-viable particles determination. Microbiological monitoring of surfaces is performed using contact plates. Environmental monitoring is performed under SOP at defined location and frequency. Moreover, environmental monitoring data are periodically trended.

In particular, the classified areas are monitored during all steps of the production of the pharmaceutical product for viable particles using settle plates, active air sampling and contact plates sampling. Operators' gloves and lab coats are also monitored at the end of the process by contact plates. The frequency and the sampling points are based on the room classification and are defined in specific internal procedures.

For all classified areas, also non-viable particles monitoring is performed by particle count air samples as part of the qualification of the facility air system and is carried out routinely in the different classified areas. For the Grade A areas (LAF biohazard hoods) and Grade B background lab, the continuous non-viable monitoring is performed.

The action levels are defined in accordance to EU GMP Guideline Annex I and are reported in Table 27 and in Table 28. Any excursion over the action level is formally recorded as deviation and investigated with the identification of microorganisms for Grade A and Grade B areas in order to establish preventive actions.

Table 15 Limits for non viable particles.

	At rest		In operation	
Grade	Max. permitted number of particles/m^3 equal to or above			
	0.5 μm	5.0 μm	0.5 μm	5.0 μm
A	3,520	20	3,520	20
B	3,520	29	352,000	2,900
C	352,000	2,900	3,520,000	29,000
D	3,520,000	29,000	not defined	not defined

Table 16 Limits for viable particles

Class	Air sample cfu/m^3	Settle Plate (∅ 90 mm) cfu/4 ore	Contact plate (∅ 55 mm) cfu/plate	Glove (5 fingers) cfu/Glove
A	< 1	< 1	< 1	< 1
B	10	5	5	5
C	100	50	25	NA
D	200	100	50	NA

2.1.A.1.1.5 GMP declaration

cGMP declarations and certifications after inspection by the competent authorities (Italian Medicines Agency– AIFA) are provided in Annex 1 and Annex 2.

2.1.A.1.2 Transportation, storage and traceability of the DS and the DP and starting relevant material

2.1.A.1.2.1 Transportation of the Drug Substance

Since the manufacturing process is conceived as a single intervention from cell source collection to DP, no transportation of the DS is foreseen.

2.1.A.1.2.2 Transportation of the Drug Product

The DP is thawed directly prior to infusion. Frozen cells in EVA bag are transported from the Officina Farmaceutica OPBG to the hospital in vapor phase nitrogen dry shipper (with logistics).

The LN2 dry shippers are targeted to maintain a temperature of ≤-130°C.

In Bergamo, due to the fact that Cell Factory and hospital are distant only 3 km, frozen cells in EVA bag are transported by an internal transporter from the Laboratorio Lanzani to the hospital in validated container containing dry ice. This transport method is targeted to maintain temperature of bad to ≤-70°C, has been validated and is controlled by a data logger.

2.1.A.1.2.3 Transportation of relevant starting material

Autologous patient's BM aspirate is delivered to the manufacturing facility as a bag containing, accompanied by appropriate documentation.

2.1.A.1.2.4 Storage of the Drug Substance

Not applicable

2.1.A.1.2.5 Storage of the Drug Product

The MSCs are stored in liquid nitrogen vapor at Officina Farmaceutica OPBG and at Centro di Terapia Cellulare "G. Lanzani" under GMP conditions. Storage conditions are controlled and recorded.

2.1.A.1.2.6 Storage of relevant starting materials

The starting material is stored at room temperature (15-25°C) in dedicated compartment until processing, which normally initiates within 2-6 hours.

2.1.A.1.2.7 Traceability of the DS and the DP and relevant materials

Traceability of the DS, DP and relevant starting materials will be carried out according to hospital and manufacturing facility policies in accordance to internal SOPs: the patient code is related to the batch number both in the batches register and in the batch record. The Drug Product and Drug Substance are managed in the same batch record. The system in place will assure traceability of the product throughout the entire manufacturing process.

2.1.A.1.2.7.1 Labelling and traceability of the Drug Substance

Not applicable.

2.1.A.1.2.7.2 Labelling and traceability of the Drug Product

An example of DP labelling is reported in Figure 13. The label applies for both primary and secondary package.

Figure 15a Example of Drug Product label

Ref.	ETICHETTA PRIMARIO E SECONDARIO
Cellule Mesenchimali Stromali derivate da Midollo Osseo	**MESNEPH – EudraCT n.**<br><br>**Promotore**: Ospedale Pediatrico "Bambino Gesù" piazza S. Onofrio 4 – 00165 Roma Tel. +39 06 68592393 email: marina.vivarelli@opbg.net<br><br>**Sperimentatore**: Dr. Marina Vivarelli; Ospedale Pediatrico Bambino Gesù-Roma Tel. +39 06 68592393 email: marina.vivarelli@opbg.net<br><br>**Centro di Produzione**: Officina Farmaceutica OPBG Viale di San Paolo 15, 00146 Roma Tel. +39 06 68593613 email: franca.fassio@opbg<br><br>Prodotto: **MSC Mesenchymal stromal cells**<br><br>ID Paziente___________<br><br>N° Lotto:__________n° sacca_______ Id OF_______<br><br>Volume: ______ml N° cellule:_____ x 10^6<br><br>Data di congelamento_______<br><br>Data di scadenza_________<br><br>Criopreservato in 90% plasma + 10% DMSO<br><br>Conservare a ≤ -150°C<br><br>Sospensione cellulare per infusione endovenosa. Contiene cellule di orìgine umana. Da somministrare secondo le istruzioni del promotore. Medicinale esclusivamente per sperimentazione clinica. Vietata la vendita al pubblico. **PER USO AUTOLOGO**. NON IRRADIARE. NON UTILIZZARE FILTRI DI LEUCORIDUZIONE.

Figure 15b Example of Drug Product label

Ref.	ETICHETTA PRIMARIO E SECONDARIO
Cellule Mesenchimali Stromali derivate da Midollo Osseo	**MESNEPH – EudraCT n.**<br><br>**Promotore**: Ospedale Pediatrico "Bambino Gesù" piazza S. Onofrio 4 - 00165 Roma Tel. +39 06 68592393 email: marina.vivarelli@opbg<br><br>**Sperimentatore**: Dr. Marina Vivarelli; Ospedale Pediatrico Bambino Gesù-Roma Tel. +39 06 68592393 email: marina.vivarelli@opbg.net<br><br>**Centro di Produzione:** Centro di Terapia Cellulare "G.Lanzani" ASST Papa Giovanni XXIII, Via Garibaldi 11/13 - 24124 Bergamo (BG) Tel. +39035227.8684 email: lablanzani@asst-pg23.it<br><br>Prodotto: **MSC Mesenchymal stromal cells**<br><br>ID Paziente___________<br><br>N° Lotto:__________ n° sacca_______ Id OF_______<br><br>Volume: ______ml N° cellule:_____ x 10^6.<br><br>Data di congelamento_______<br><br>Data di scadenza_________<br><br>Criopreservato in 90% plasma + 10% DMSO<br><br>Conservare a ≤ -150°C<br><br>PER USO AUTOLOGO<br><br>Sospensione cellulare per infusione endovenosa. Contiene cellule di origine umana. Da somministrare secondo le istruzioni del promotore. Medicinale esclusivamente per sperimentazione clinica. Vietata la vendita al pubblico. **PER USO AUTOLOGO**. NON IRRADIARE. NON UTILIZZARE FILTRI DI LEUCORIDUZIONE.

2.1.A.1.2.7.3 Labelling and traceability of relevant starting material

Example of label for autologous donor cells is reported below. Each patient is identified with RM or BG depending on the hospital where is enrolled in the study and sequential number

Figure 16 Labels for autologous donor cells

ID paziente:_________<br>Data del prelievo:_________<br>Aspirato Midollo Osseo<br>Conservazione: 15-25°CC

2.1.A.2 Adventitious agents safety evaluation

MSC is an autologous cell based medicinal product. The MSC product is manufactured by expansion of bone marrow derived mesenchymal stem cells without any hold step. Due to the nature of the product (i.e. cells) there is no possibility to introduce viral removal and inactivation steps. Therefore, adequate precautions to prevent introduction of viral adventitious and endogenous agents and to ensure microbial safety of MSC drug product, are taken as detailed below:

• Control of the human derived raw material entering the manufacturing process through certificate of origin and suitability. In particular for human piastrinic lysate, this information can be obtained from the vendor.

• Donor testing for relevant human viruses as part of the donor eligibility assessment;

• Control of the MSC drug product manufacturing process. For open steps, the process is performed under environmentally controlled conditions;

• Routine testing for microbial contaminants performed at various points during MSC drug product manufacturing;

• Environmental controls;

• Cleaning and decontamination of work surfaces and equipment;

• Aseptic verification is conducted, simulating all process steps and interventions to verify that the process is capable of maintaining sterility;

• Microbial contaminants testing as part of drug product release;

• Bacterial endotoxin;

• Sterility;

• Mycoplasma.

2.2 Section 2.2 – Non clinical pharmacology and toxicology data

Please refer to the Investigator's Brochure and clinical protocol.

REFERENCES

1. Vivarelli, M, Massella L, Ruggiero B, Emma F. Minimal change disease. Clin J Am Soc Nephrol 2017;12(2):332-345
2. Niaudet P, Boyer, O. Idiopathic Nephrotic Syndrome in Children: Clinical Aspects. In: Pediatric Nephrology-7th edition. Springer-Verlag 2016;839-82.
3. Colucci M, Corpetti G, Emma F, Vivarelli M. Immunology of idiopathic nephrotic syndrome. Pediatr Nephrol 2017 in press
4. Bertelli R, Bonanni A, Di Donato A, et al. Regulatory T cells and minimal change nephropathy: in the midst of a complex network. Clin Exp Immunology 2016;183(2):166-74
5. Ravani P, Bonanni A, Rossi R, et al. Anti-CD20 Antibodies for Idiopathic Nephrotic Syndrome in Children. CJASN 2016;11(4):710-20.
6. Ruggenenti P, Ruggiero B, Cravedi P, et al for the Rituximab in Nephrotic Syndrome of Steroid-Dependent or Frequently Relapsing Minimal Change Disease Or Focal Segmental Glomerulosclerosis (NEMO) Study Group: Rituximab in steroid-dependent or frequently relapsing idiopathic nephrotic syndrome. JASN 2014;25(4): 850-63.
7. Nauta AJ, Fibbe WE. Immunomodulatory properties of mesenchymal stromal cells. Blood. 2007;110(10):3499-506.
8. Bernardo ME, Fibbe WE. Mesenchymal stromal cells: sensors and switchers of inflammation, Cell Stem Cell 2013; 13: 392-402.
9. Keating A. Mesenchymal stromal cells: new directions. Cell Stem Cell 2012; 709-716.
10. Le Blanc K, Mougiakakos D. Multipotent mesenchymal stromal cells and the innate immune system. Nat Rev Immunology 2012; 12:383.396
11. Perico N, Casiraghi F, Remuzzi G. Clinical Translation of Mesenchymal Stromal Cell Therapies in Nephrology. Journal American Society Nephrol 2017; in press
12. Peired AJ, Sisti A, Romagnani P. Mesenchymal Stem Cell-Based Therapy for Kidney Disease: A Review of Clinical Evidence. Stem Cells International 2016;4798639
13. Starc N, Li M, Algeri M, et al. Phenotypic and functional characterization of mesenchymal stromal cells isolated from pediatric patients with severe idiopathic nephrotic syndrome Cytotherapy. 2017 (17)30775-2
14. Introna M, Lucchini G, Dander E et al. Treatment of graft versus host disease with mesenchymal stromal cells: a phase I study on 40 adult and pediatric patients. Biol Blood Marrow Transplant. 2014 20(3):375-81.
15. Perico N, Casiraghi F, Gotti E.et al. Mesenchymal stromal cells and kidney transplantation: pretransplant infusion protects from graft dysfunction while fostering immunoregulation. Transpl Int. 2013 (9):867-78.
16. Perico N, Casiraghi F. Introna M. et al. Autologous mesenchymal stromal cells and kidney transplantation: a pilot study of safety and clinical feasibility. Clin J Am Soc Nephrol. 2011 6(2):412-22
17. Perico N. et al. Frontiers in Immunology in press
18. Golay J, Pedrini O, Capelli C, et al. Utility of routine evaluation of sterility of cellular therapy products with or without extensive manipulation: Best practices and clinical significance. Cytotherapy. 2018 20(2):262-270.
19. Strunk D Lozano M, Marks DC, et al. International Forum on GMP-grade human platelet lysate for cell propagation: summary. Vox Sang. 2018 113(1):80-87.

20. Capelli C, Pedrini O, Valgardsdottir Ret al. Clinical grade expansion of MSCs. Immunol Lett. 2015 168(2):222-7
21. Capelli C, Pedrini O, Cassina G, et al. Frequent occurrence of non-malignant genetic alterations in clinical grade mesenchymal stromal cells expanded for cell therapy protocols. Haematologica. 2014 99(6):e94-7.
